# Molecular Ruler Variation in Insect Dicer-2 Suggests a Structural Basis for Species-Dependent siRNA Length and Antiviral Defense Diversity

**DOI:** 10.3390/v18030285

**Published:** 2026-02-27

**Authors:** Moises Joao Zotti, Juliana Wegner, Bruno Freitas Farias, Guy Smagghe

**Affiliations:** 1 Laboratory of Molecular Entomology and Applied Bioinformatics, Department of Crop Protection, Federal University of Pelotas, Pelotas 96010-610, Brazil; juliana.wegner1@gmail.com (J.W.); bruno.f.far@gmail.com (B.F.F.); 2 Institute of Entomology, Guizhou University, Guiyang 550025, China; 3 Department of Biology, Vrije Universiteit Brussel (VUB), 1050 Brussels, Belgium

**Keywords:** Dicer-2, molecular ruler hypothesis, modeling, RNase III, PAZ, virus interactions, siRNA, insects, evolutionary relationship

## Abstract

Understanding species-dependent siRNA length generation provides both fundamental and applied insights. At the basic level, it highlights an underappreciated dimension of RNAi diversity in insects, emphasizing that antiviral immunity cannot be fully understood from *Drosophila melanogaster* alone. At the applied level, these findings have direct implications for the design of dsRNA-based pest management strategies, where tailoring siRNA production to the target insect order could enhance both efficacy and specificity. Previously studies demonstrated that Dicer-2-generated siRNAs exhibit distinct, species-dependent length distributions: dipterans (*D. melanogaster*) and coleopterans (*Tribolium castaneum*) mainly produce 21-nt siRNAs; and hymenopterans (*Bombus terrestris*) and orthopterans (*Locusta migratoria*) generate siRNAs enriched at 22 nt; whereas lepidopterans such as *Spodoptera exigua* and *Trichoplusia ni* predominantly produce 20-nt siRNAs. The central biological question motivating this study was whether structural divergence in Dicer-2 may explain these lineage-specific differences in siRNA length profiles and antiviral RNAi efficiency. To address this, we interpreted observed structural variations in the context of the “molecular ruler” hypothesis and integrated them with previous experimental data on siRNA length variation across insect taxa. Future studies that combine high-resolution structural biology, comparative genomics, and functional assays will be essential to experimentally test whether the structural correlations proposed here determine Dicer-2 cleavage length in vivo and in vitro, and to leverage this knowledge for both agricultural and biomedical applications.

## 1. Introduction

RNA interference (RNAi) is an evolutionarily conserved gene silencing mechanism that plays a central role in antiviral immunity in insects [1,2]. Triggered by the presence of double-stranded RNA (dsRNA), RNAi provides a sequence-specific means of degrading viral transcripts, thereby limiting infection and controlling viral replication. The key of this process is the Ribonuclease III (RNase III) enzyme Dicer-2, which cleaves long dsRNA into small interfering RNAs (siRNAs). These siRNAs, typically 18–24 nucleotides in length, are incorporated into the RNA-induced silencing complex (RISC), where they guide Argonaute-2 (Ago-2)-mediated cleavage of complementary viral RNAs. In this way, Dicer-2-generated siRNAs serve as molecular signatures of invading pathogens, determining the specificity and efficiency of the insect antiviral response [3].

Dicer-2 belongs to the RNase III family of endonucleases and is specialized for recognizing and processing long dsRNA molecules [4,5]. Its modular structure includes a helicase domain, a Piwi-Argonaute-Zwille domain (PAZ) for dsRNA end recognition, two RNase III catalytic domains that form a dimeric cleavage center, and a dsRNA-binding domain. During siRNA biogenesis, Dicer-2 measures a defined distance from the bound end of the dsRNA duplex to the catalytic site, producing siRNA duplexes of characteristic length. The guide strand is loaded into Ago-2 within RISC, whereas the passenger strand is degraded. Because RISC function depends on precise base-pairing between the guide strand and its target RNA, the size and integrity of Dicer-2-generated siRNAs are critical for effective silencing. In *Drosophila melanogaster*, the canonical dipteran model, Dicer-2 generates siRNAs almost exclusively 21 nucleotides in length [6,7]. This uniform size underpins an efficient antiviral response against a broad spectrum of RNA viruses, including Flock House Virus (FHV), Drosophila C Virus (DCV), and Cricket Paralysis Virus (CrPV) [8,9,10]. While studies in dipterans have elucidated fundamental mechanisms of RNAi, extrapolating these findings to other insect orders risks overlooking evolutionary and mechanistic diversity in antiviral RNAi.

Recent studies reveal substantial interspecies variation in siRNA structural features. Notably, the predominant length of siRNAs produced by Dicer-2 is not uniform across insects but shows order-specific distributions [7]. Dipterans such as *D. melanogaster* produce mainly 21-nt siRNAs, hymenopterans such as *Bombus terrestris* and orthopterans like *Locusta migratoria* generate siRNAs enriched at 22 nucleotides, lepidopterans including *Spodoptera exigua* and *Trichoplusia ni* produce primarily 20-nt siRNAs, and coleopterans like *Tribolium castaneum* maintain the canonical 21-nt length. These patterns are consistent across diverse viruses and synthetic dsRNA triggers, suggesting that siRNA length is dictated by host RNAi machinery rather than viral properties. Furthermore, closely related species tend to share similar siRNA length profiles, indicating that Dicer-2 cleavage preferences are phylogenetically conserved. Collectively, these observations raise fundamental biological questions about the molecular determinants and evolutionary significance of Dicer-2-mediated siRNA length variation.

Structural studies in plants and mammals indicate that siRNA length is determined by the distance between the PAZ domain, which anchors dsRNA termini, and the RNase III catalytic center, which mediates cleavage. Small alterations in these domains could shift the length of siRNAs produced [11]. Supporting this, Dicer-2 and Ago-2 are among the most rapidly evolving components of insect antiviral immunity, reflecting ongoing arms races with viruses. It is therefore plausible that structural divergence in Dicer-2 underlies observed interspecies differences in siRNA length, potentially reinforced by Argonaute binding preferences [11,12].

Testing this hypothesis could illuminate whether Dicer-2 cleavage specificity and Argonaute binding constraints co-evolve to optimize antiviral defense. Understanding species-dependent siRNA length also has practical implications for RNAi-based pest control. The efficacy of dsRNA-based insecticides varies widely across orders: coleopterans such as *T. castaneum* are highly responsive to ingested dsRNA, whereas many lepidopterans are refractory under field conditions. One determinant of this variability may be the efficiency with which Dicer-2 processes dsRNA into siRNAs of optimal length for RISC loading. Hence, elucidating how Dicer-2 generates siRNAs across insect lineages is critical for understanding the evolution of antiviral defense and designing effective, species-specific RNAi-based pest management strategies. Ensuring that delivered dsRNA is processed into siRNAs of the correct length may enhance RNAi efficacy across diverse insects.

In the present study, we focus on evidence that Dicer-2 generates siRNAs of distinct predominant lengths in different insect species, particularly under viral challenge [6]. Comparative analyses across Diptera, Coleoptera, Hymenoptera, Orthoptera, and Lepidoptera indicate that siRNA length variation reflects both mechanistic properties of Dicer-2 and evolutionary adaptation. We hypothesize that interspecies differences in siRNA length are determined by structural variation in Dicer-2’s cleavage site architecture. Specifically, the spatial configuration of the PAZ domain, RNase III catalytic domains, and the intervening enzyme cavity may define the measurement “ruler” by which dsRNA substrates are processed. Variation in this geometry may explain why some species produce predominantly 20-nt siRNAs, others 21-nt, and others 22-nt. Indeed our previous work [6] demonstrated species-dependent siRNA length distributions: coleopterans (*T. castaneum*) and dipterans (*D. melanogaster*) produced mostly 21-nt siRNAs; hymenopterans (*B. terrestris*) and orthopterans (*L. migratoria*) generated siRNAs enriched at 22 nt; while lepidopterans such as *S. exigua* and *T. ni* predominantly produced 20-nt siRNAs. These findings suggested that siRNA length is an intrinsic property of the host RNAi machinery rather than determined by the infecting virus. The prevailing mechanistic model posits that the PAZ domain anchors the dsRNA terminus, while the distance to the RNase III catalytic center dictates cleavage length [13]. Subtle structural differences in this “molecular caliper” could shift the distance by one or two nucleotides, producing species-specific siRNA length profiles. To test this, we modeled three-dimensional (3D) structures of Dicer-2 from representative species across Diptera, Coleoptera, Hymenoptera, Orthoptera, and Lepidoptera. We analyzed the enzyme cavity and docking space to simulate dsRNA substrate binding and predict siRNA length outputs. By comparing structural predictions with in vivo siRNA length data, we aim to determine whether Dicer-2 architecture correlates with observed siRNA length distributions. This integrative approach, combining comparative structural modeling with empirical data, addresses a central question in insect virology: Does the architecture of Dicer-2 explain the evolutionary diversification of siRNA length? Confirming this relationship would provide mechanistic insight into species-specific RNAi responses, deepen our understanding of insect antiviral immunity, and guide the design of RNAi-based pest control strategies tailored to specific insect orders.

## 2. Materials and Methods

### 2.1. Sequence Retrieval and Selection of Dicer-2 Orthologs

To investigate structural variation in Dicer-2 across insect species, representative taxa were selected from four major insect orders with diverse evolutionary histories and RNAi responses: *T. castaneum* (Coleoptera), *B. terrestris* (Hymenoptera), *L. migratoria* (Orthoptera), and *T. ni* (Lepidoptera). The Dicer-2 protein sequence from *D. melanogaster* (Diptera) was included as a reference. Full-length coding sequences (CDS) or translated amino acid sequences for Dicer-2 orthologs were retrieved from the NCBI RefSeq and UniProt databases using BLASTp (v2.17.0) searches with *D. melanogaster* Dicer-2 (UniProt ID: Q9V3W0) as the query. All sequences were verified for completeness by confirming the presence of canonical Dicer-2 domains (helicase, PAZ, RNase III A and B, and dsRNA-binding domains) using InterProScan (v107.0) and Pfam (v38.1) domain annotations.

### 2.2. Multiple Sequence Alignment and Domain Annotation

To confirm orthology and identify conserved regions, all retrieved Dicer-2 sequences were aligned using Clustal Omega (v1.2.4) with default parameters. Manual curation was performed to refine alignment boundaries in highly variable regions. Conserved functional domains, including the helicase DExD/H-box domain, DUF283, PAZ domain, tandem RNase III catalytic domains (RNase III A and B), and the C-terminal dsRNA-binding domain, were annotated based on alignment with *D. melanogaster* Dicer-2 and published references [4,5]. These annotations were subsequently mapped onto the modeled structures to guide structural interpretation and identification of active sites.

### 2.3. Homology Modeling of Dicer-2 Structures

Homology modeling was performed using SWISS-MODEL (https://swissmodel.expasy.org/ (accessed on 21 August 2024)), which constructs 3D protein structures from known templates. The *D. melanogaster* Dicer-2 crystal structure (PDB ID: 7v6c) [5] was selected as the primary template because of its high resolution and domain completeness. Although this structure includes dsRNA bound to the protein, the substrate is positioned in a pre-cleavage state. To model the active conformation, a secondary template (PDB ID: 7w0e) [4], in which dsRNA is positioned within the PAZ and RNase III domains, was also employed.

For each insect species, full-length Dicer-2 sequences were submitted to SWISS-MODEL. Template-target alignments were automatically computed, and model quality was assessed using Global Model Quality Estimation (GMQE) and Qualitative Model Energy ANalysis scores (QMEAN). Only models with GMQE > 0.6 and QMEAN Z-scores within ±1.5 of the expected range for experimentally determined structures were retained. Where necessary, iterative modeling was performed to improve the alignment of flexible loop regions, particularly within the PAZ domain.

### 2.4. Structural Superimposition and Comparative Analysis

To assess structural conservation and divergence, all modeled Dicer-2 structures were superimposed onto the *D. melanogaster* templates (7v6c and 7w0e) using the PyMOL Molecular Graphics System (v2.5, Schrödinger LLC). Superimpositions were based on Cα atoms, and root mean square deviation (RMSD) values were calculated to quantify overall and domain-level structural similarity. RMSD values were computed separately for the helicase, PAZ, and RNase III regions.

**Figure 1:**
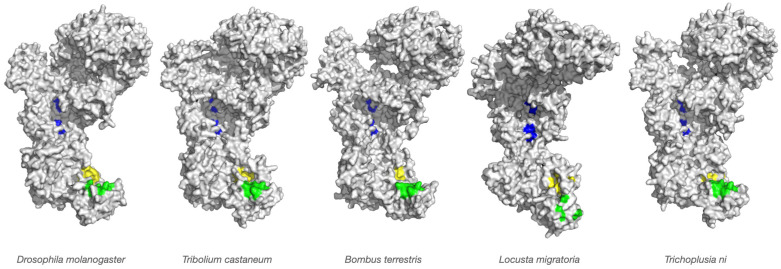
Structural conservation of Dicer-2 across insect species. Superimposed homology models of Dicer-2 from *Tribolium castaneum* (Coleoptera), *Bombus terrestris* (Hymenoptera), *Locusta migratoria* (Orthoptera) and *Trichoplusia ni* (Lepidoptera) aligned to the *Drosophila melanogaster* Dicer-2 crystal structure (PDB: 7v6c). Low RMSD values (0.200–0.245 Å) indicate strong conservation of global architecture, encompassing the helicase, PAZ, and dual RNase III domains. The two blue regions represent the RNase III domains (A and B), which form the catalytic site for dsRNA cleavage, while the green and yellow regions in the PAZ domain correspond to the 3′ and 5′ nucleotide-binding pockets that anchor dsRNA termini.

**Figure 2:**
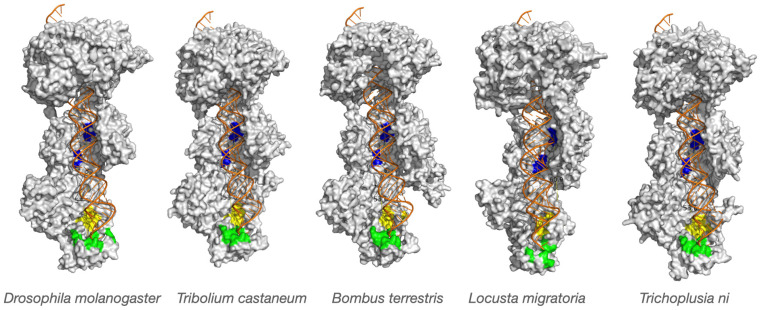
Structural variation in functional domains of insect Dicer-2. Close-up views of the PAZ domain (3′ and 5′ nucleotide-binding pockets shown in green and yellow, respectively) and the RNase III catalytic centers (domains A and B, shown in blue). Homology models of Dicer-2 from *T. castaneum*, *B. terrestris*, *L. migratoria* and *T. ni* are superimposed and aligned to the *D. melanogaster* Dicer-2 crystal structure (PDB: 7w0e), representing the active dicing conformation with dsRNA docked. Subtle but consistent interspecies shifts in pocket geometry and catalytic site positioning are evident, suggesting that structural divergence may influence dsRNA anchoring and cleavage distance, which are key determinants of siRNA length and antiviral RNAi efficiency.

**Figure 3:**
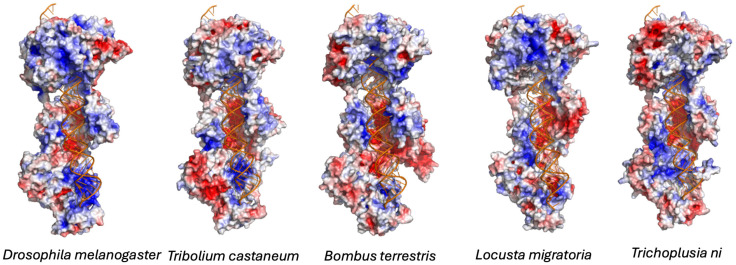
Electrostatic potential surface of insect Dicer-2. Electrostatic potential maps of Dicer-2 from *T. castaneum*, *B. terrestris*, *L. migratoria* and *T. ni* modeled and aligned to *D. melanogaster* Dicer-2 (PDB: 7w0e). Electrostatic surfaces were calculated in PyMOL using the Adaptive Poisson-Boltzmann Solver (APBS) plugin. Positively charged regions (blue) and negatively charged regions (red) highlight differences in the electrostatic landscape surrounding the PAZ domain and RNase III catalytic cleft. Species-specific charge distributions may influence dsRNA substrate binding, positioning, and cleavage efficiency, contributing to observed variation in siRNA length profiles.

Visual inspection and manual comparisons were used to identify differences in key functional regions, including: (i) the 3′ and 5′ binding pockets of the PAZ domain (substrate anchoring sites); (ii) the RNase III catalytic cleft (slicing site); and (iii) the relative positioning of the PAZ and RNase III domains (the “molecular ruler” distance determining siRNA length). Color-coded overlays were generated to visualize species-specific variations (Figure 1 and Figure 2). Electrostatic potentials were calculated using the Adaptive Poisson–Boltzmann Solver (APBS) plugin in PyMOL with default parameters (Figure 3).

### 2.5. Identification of Catalytic Residues and Active Sites

Catalytic residues were mapped using previously characterized Dicer-2 active sites from *D. melanogaster* as references (Table 1). Conserved RNase III catalytic residues, namely acidic amino acids responsible for metal ion coordination and cleavage catalysis, were identified through literature review and multiple sequence alignment. These residues were mapped onto modeled structures using PyMOL and confirmed via structural alignment with known RNase III active sites from related Dicer-family enzymes. The distance from the PAZ anchoring site to the RNase III catalytic residues was then measured using PyMOL’s internal measurement tools, providing an estimate of the siRNA product length determined by each modeled enzyme.

### 2.6. Measurement of PAZ-Catalytic Site Distances (“Molecular Ruler” Analysis)

Because siRNA length is dictated by the spatial separation between the dsRNA anchoring site (PAZ domain) and the catalytic RNase III center, we measured this distance in each modeled structure as a proxy for expected siRNA product size. Measurements were taken between the centers of the 3′/5′ terminal nucleotide-binding pockets in the PAZ domain and the centers of the catalytic residues (typically DDXE/D motifs) in the RNase III A and B domains (See the Supplementary Figure S1 for more details). Variations of ±1–2 Å were considered potentially significant, as they correspond to approximately 1–2 nucleotide shifts in siRNA cleavage position.

### 2.7. Structural Visualization and Figure Generation

All structural figures were generated in PyMOL. Global superimpositions were rendered for each species (*Tribolium*, *Bombus*, *Locusta*, and *Trichoplusia*, and *Drosophila* as reference). Functional domains were color-coded as follows: blue regions represent the RNase III domains (A and B, the dsRNA slicing site), while green and yellow regions in the PAZ domain represent the 3′ and 5′ pockets that anchor the dsRNA termini, respectively.

### 2.8. Statistical Analysis

All RMSD calculations, distance measurements, and pocket geometry analyses were repeated in three independent modeling runs to ensure reproducibility. Descriptive statistics (mean ± standard deviation) were calculated using built-in PyMOL functions and custom Python scripts. Structural variations exceeding 0.5 Å in active site spacing were considered biologically relevant, consistent with prior estimates of the molecular ruler mechanism [11].

## 3. Results

### 3.1. Comparative Structural Modeling of Dicer-2 Across Insect Species

To investigate whether structural variation in the antiviral RNAi enzyme Dicer-2 underlies species-specific differences in siRNA biogenesis, we performed comparative homology modeling of Dicer-2 orthologs from four evolutionarily distant insect species, namely *T. castaneum* (Coleoptera), *B. terrestris* (Hymenoptera), *L. migratoria* (Orthoptera) and *T. ni* (Lepidoptera), and compared them with the well-characterized *D. melanogaster* Dicer-2 (Diptera). These taxa represent distinct evolutionary lineages, ecological niches, and viral exposure histories, providing an ideal framework to explore structural and functional diversity in antiviral RNAi.

As shown in Figure 1, homology models were generated using SWISS-MODEL, with *Drosophila* Dicer-2 crystal structures (PDB: 7v6c and 7w0e) as templates. We refer here to alignments in the supplementary materials for more details. Template 7v6c represents a pre-cleavage conformation (dsRNA bound but not actively cleaving), whereas 7w0e captures an active conformation with dsRNA engaged in both PAZ and RNase III domains. Superimposition of the modeled structures revealed strong conservation of the overall architecture across all species, reflected in low RMSD values: 0.240 Å (6243 atoms) for *Tribolium*, 0.200 Å (6147 atoms) for *Bombus*, 0.233 Å (5619 atoms) for *Locusta* and 0.245 Å (6568 atoms) for *Trichoplusia*. These data demonstrate that the global domain organization, including the helicase, PAZ, and dual RNase III domains, is highly conserved across insect taxa. This conservation is consistent with the central role of Dicer-2 in antiviral defense and transposon silencing. The preservation of its modular structure suggests that evolutionary diversification likely targets regions that fine-tune enzyme performance rather than altering its core catalytic capacity.

It is important to mention that these alignment distances correspond to the alignments between the alpha carbons of the protein backbone. Homology modeling involves using a known 3D structure of a related protein, referred to as the template, to construct a 3D model of a target protein based on its amino acid sequence. In brief, the backbone coordinates (N, Cα, C) are copied from the template to the target protein; this process is oriented by the alignment between them. Finally, the side chains of the target protein are added guided by specific libraries of rotamers. Long dsRNA anchoring in Dicer-2 domains (3′ and 5′ pockets, Platform and RNase III A/B), occur in grooves shaped by amino acid side chains via electrostatic interactions rather than direct atomic contact. While homologous modeling closely matches the template, the amino acids and their side chains can rotate freely, altering the shape and depth of contact points. These subtle differences may shorten or lengthen the distances between 3′ and 5′ pockets, Platform and RNase III A/B domains, resulting in different siRNA lengths.

### 3.2. Domain-Level Variation and Functional Implications

Despite overall structural conservation, close inspection revealed subtle yet potentially significant interspecies variations in key functional domains, particularly within the PAZ and RNase III regions that anchor and cleave dsRNA substrates, respectively (Table 1; Figure 2).

The RNase III catalytic domains showed strong structural conservation, consistent with their indispensable role in dsRNA cleavage. The Dicer-2 enzyme processes long dsRNA molecules into siRNA duplexes with characteristic two-nucleotide 3′ overhangs and 5′ phosphates, retaining the antiparallel orientation of the original strands. For each modeled enzyme, we measured the distance between the PAZ anchoring pocket and the RNase III catalytic residues, corresponding to the enzyme’s “molecular ruler” mechanism (Table 2).

In the case of *L. migratoria*, the superimposed model may visually suggest that RNase III catalytic centers engage distinct dsRNA grooves. However, distance measurements and residue alignment confirm that both RNase III domains act on opposite strands of the same duplex, consistent with canonical RNase III architecture. The apparent divergence reflects minor rotational differences in homology modeling rather than a mechanistic deviation.

Average distances correlated with known siRNA length profiles: for *D. melanogaster* this is 60.3 Å (~21 nt), for *T. castaneum* 59.8 Å (~21 nt), for *B. terrestris* 62.2 Å (~22 nt), for *T. ni* 58.3 Å (~20 nt) and 62.3 Å (~22 nt) for *L. migratoria*. Small but consistent differences of 1–2 Å were observed between species, translating to shifts in one, two or more nucleotides in siRNA product length. According to the molecular ruler model, even minor adjustments in the spatial relationship between PAZ and RNase III centers can alter cleavage site selection. These subtle yet reproducible shifts are consistent with the hypothesis that species-specific siRNA lengths may reflect intrinsic properties of Dicer-2 geometry.

It is also important to highlight the potential role of accessory proteins in modulating Dicer-2, which in turn affects the molecular ruler mechanism used to process dsRNA substrates. In *Drosophila*, the double-stranded RNA-binding proteins R2D2 and Loquacious (Loqs-PD) specifically regulate Dicer-2 activity [14]. Loqs-PD modulates Dicer-2’s cleavage specificity by interacting with the dsRNA termini, thereby influencing where Dicer-2 slices the substrate. R2D2, on the other hand, binds to the helicase domain at the N-terminus of Dicer-2, restricting its activity [5,15]. This restriction prevents Dicer-2 from processing pre-miRNAs and instead directs it to convert long dsRNA into siRNAs [16]. Similar accessory protein-mediated mechanisms have been observed in other insects and organisms, suggesting that protein partners play a crucial role in fine-tuning RNA interference pathways across diverse species [17]. These interactions not only impact the efficiency and specificity of siRNA production but also contribute to the evolutionary adaptability of antiviral responses in insects and other eukaryotes.

We also detected variations in the shape and electrostatic potential of the PAZ domain’s 3′ and 5′ binding pockets (Figure 2 and Figure 3). Differences in pocket depth and charge distribution may affect how dsRNA termini are recognized and anchored, potentially influencing substrate orientation and cleavage precision. The electrostatic potential surface is essential for Dicer-2 to bind dsRNA and cofactors such as R2D2 and Loqs-PD. It acts as a docking platform for key RNA substrates and proteins, and changes in its charge distribution directly affect complex formation with cofactors [4]. Together, these data indicate that although the catalytic mechanism of Dicer-2 is conserved, minor adaptive changes in domain spacing and binding-site architecture may modulate siRNA length and processing efficiency in a species-dependent manner. We think that this structural flexibility could represent an evolutionary tuning mechanism, enabling each insect lineage to optimize antiviral RNAi to its ecological and virological environment.

### 3.3. Biological Implications: siRNA Length Diversity and Antiviral Function

Our structural findings (Figure 2 and Table 2) align closely with empirical observations of siRNA length variation among insects [7]. In *D. melanogaster*, Dicer-2 produces predominantly 21-nt siRNAs, whereas in other insects siRNAs may differ in size. Good examples are as follows: *Aedes aegypti* often produces 21–22 nt species [17], and lepidopterans such as *S. exigua* and *T. ni* tend toward 20-nt products [7]. These modest differences have potentially large biological consequences.

siRNA length influences RISC loading efficiency, Argonaute binding affinity, and the precision of target recognition. Variations in siRNA size can therefore affect both the potency and the selectivity of antiviral RNAi responses. Furthermore, viruses encode viral suppressors of RNAi (VSRs) that inhibit Dicer-2 or sequester siRNAs. Thus, the observed species-specific structural adaptations in Dicer-2 (Figure 1, Figure 2 and Figure 3) may represent evolutionary countermeasures to viral antagonism.

From an evolutionary standpoint, these adaptations exemplify an ongoing molecular arms race between host and virus. Dicer-2 diversification likely reflects selective pressures imposed by viral diversity, infection intensity, and host ecology. For example, eusocial hymenopterans such as *B. terrestris* may experience high pathogen exposure, favoring diversification in RNAi components to counter a broader viral repertoire. Conversely, lineages with narrower ecological niches may retain more conserved RNAi machinery. The data indicate that minor changes in Dicer-2’s structure can adjust antiviral RNAi, affecting species-specific immunity and vulnerability to viruses.

### 3.4. Evolutionary Considerations and Structural Flexibility

The structural patterns observed here illuminate how Dicer-2 balances rigidity and flexibility in its antiviral role. Precise molecular measurement requires a rigid core, yet substrate engagement and cleavage demand conformational adaptability. Our homology models, while static, likely capture biologically relevant configurations that correspond with species-specific siRNA lengths (Figure 2 and Table 2).

Evolutionary pressures appear to act asymmetrically on different Dicer-2 domains: the RNase III catalytic center is under strong purifying selection, maintaining enzymatic integrity, while peripheral domains, especially the PAZ and its interface with the RNase III domains, show greater divergence. This suggests that natural selection preserves catalytic precision but allows adaptive remodeling of the measurement apparatus. We believe that such a pattern mirrors that observed in other antiviral RNAi components, notably Ago-2, which exhibits signatures of positive selection in insects. Both Dicer-2 and Ago-2 may co-evolve to maintain compatibility within the RNAi machinery while responding to viral selective pressures. This modular evolution could help maintain overall functionality while introducing subtle innovations that confer lineage-specific advantages in antiviral defense.

Importantly to note here is that the present work establishes structural correlations but does not experimentally demonstrate causation. Functional validation through recombinant Dicer-2 cleavage assays, mutagenesis of PAZ-RNase III spacing, and comparative small RNA sequencing remains necessary to confirm whether the measured geometric differences directly determine siRNA length in vivo.

### 3.5. Future Directions and Research Needs

While our comparative modeling provides mechanistic insights and generates testable hypotheses, we believe that several important gaps remain that must be addressed through further integrated experimental and computational approaches. Functional validation of the structural predictions is a key priority. Experimental confirmation of siRNA length differences through comparative small RNA sequencing during viral infections in diverse insect species would directly link structural variation in Dicer-2 to the siRNA size distributions observed in vivo. Complementary in vitro cleavage assays using recombinant Dicer-2 proteins will be essential to determine how specific structural differences influence substrate recognition, cleavage site selection, and catalytic efficiency. In parallel, high-resolution structural elucidation using cryo-electron microscopy (cryo-EM) or X-ray crystallography of Dicer-2 orthologs complexed with dsRNA substrates will be critical to confirm the predicted spatial relationships between the PAZ and RNase III domains and to capture conformational transitions during the catalytic cycle. Dynamic modeling and molecular dynamics (MD) simulations represent another promising avenue, enabling detailed exploration of domain flexibility, electrostatic interactions, and substrate motion that contribute to catalytic precision. Comparative MD analyses among orthologs could help identify dynamic determinants of species-specific siRNA lengths and reveal how Dicer-2 responds to structural perturbations or mutations. Functional assays in vivo, including experimental infection models in insects expressing divergent Dicer-2 variants, are also needed to assess how siRNA length impacts viral replication dynamics, antiviral efficiency, and host survival outcomes, thereby establishing a direct link between structural variation and immune efficacy. Finally, we think that exploring the coevolutionary relationship between Dicer-2 and VSRs remains a compelling future direction. Comparative genomic analyses assessing whether viral VSR diversity correlates with Dicer-2 structural diversification could reveal reciprocal adaptation between host defense and viral countermeasures. Collectively, these research directions will bridge the gap between computational structure and biological function, advancing our understanding of how Dicer-2 diversification shapes antiviral RNAi across insect taxa. Moreover, by further testing the structural principles that may influence siRNA production, the present insights may guide the rational design of dsRNA-based pest control strategies optimized for specific insect groups and contribute to the broader application of RNAi in both agriculture and biomedicine.

## 4. Conclusions

Our comparative analysis reveals a remarkable balance between conservation and innovation in insect Dicer-2 evolution. While the enzyme’s global fold and catalytic architecture are highly conserved, subtle differences in substrate anchoring and domain spacing appear to modulate siRNA length and processing dynamics. These fine-tuned structural adjustments provide a plausible structural framework that may contribute to species-specific siRNA length profiles observed across insects. From a broader perspective, these findings highlight Dicer-2 as a key player in the molecular arms race between insects and their viral pathogens. Its modular adaptability allows preservation of essential catalytic function while accommodating lineage-specific optimization of antiviral RNAi. We think that future integrative studies, combining structural biology, comparative genomics, and functional virology, will allow us to confirm these predictions and to determine how Dicer-2 diversity contributes to viral susceptibility, immune plasticity, and RNAi-based applications in pest and vector management.

## Figures and Tables

**Table 1:** Comparative alignment of key amino acid residues in Dicer-2 catalytic and substrate-binding regions across insect species. Amino acid residues corresponding to the RNase III domains (RIII-A and RIII-B) and the 3′ and 5′ nucleotide-binding pockets (PAZ domain) are listed as identified in the *Drosophila melanogaster* Dicer-2 crystal structure (PDB: 7V6C; https://www.rcsb.org/structure/7V6C (accessed on 14 September 2024)) and compared to the corresponding residues in the homology models of *Tribolium castaneum*, *Bombus terrestris*, *Locusta migratoria* and *Trichoplusia ni*. These conserved and variant residues were used to map catalytic and anchoring sites within the modeled structures and to assess structural conservation and divergence across species. The question mark indicates that the amino acid was not found in the alignment or was inconsistent, due to exclusion.

	RIII-A				RIII-B				
*D. melanogaster*	E1213	D1217	D1368	E1371	E1472	D1476	D1614	E1617	
*T. castaneum*	E1165	D1169	D1308	E1311	E1410	D1414	D1515	E1518	
*B. terrestris*	E1016	D1020	D1160	E1163	E1258	D1262	D1364	E1367	
*L. migratoria*	E1032	D1036	D1167	E1170	E1270	D1274	D1377	E1380	
*T. ni*	E1615	D1619	D1766	E1769	E1869	D1873	D1975	E1978	
	**Pocket 3′**								
*D. melanogaster*	Y886	A887	N888	K910	F920	T921	K924	Y925	V971
*T. castaneum*	Y865	R866	S867	?	Y898	Y899	K902	H903	E947
*B. terrestris*	Y746	R747	A748	THR769?	Y778	Y779	K782	H783	E819
*L. migratoria*	R858	K859	F860	?	Q889	N890	I893	D894	I934
*T. ni*	Y1290	R1291	V1292	?	Y1323	Y1324	K1327	Y1328	E1386
	**Pocket 5′**								
*D. melanogaster*	Y740	H743	M744	N773	Q774	R943	D944	L945	T946
*T. castaneum*	N721	T724	I725	S754	N755	K918	G919	L920	S921
*B. terrestris*	R611	T614	F615	R644	V645	K798	S799	I800	S801
*L. migratoria*	D729	I732	L733	A762	E763	I910	I911	I912	S913
*T. ni*	R1150	A1153	L1154	T1183	V1184	R1344	N1345	I1346	S1347

**Table 2:** Measured distances (Å) between the RNase III catalytic domains (A and B) and the 3′/5′ nucleotide-binding pockets in insect Dicer-2 homologs. Distances were measured in PyMOL using the *Measurement Wizard* tool. These values represent the spatial separation between the PAZ domain anchoring sites (3′ and 5′ pockets) and the RNase III catalytic centers, which together define the “molecular ruler” determining siRNA product length. Comparative distances among species correspond to predicted siRNA lengths of ~20, 21, or 22 nucleotides, consistent with experimentally observed distributions.

	RIII-A < > 3′ Pocket	RIII-B < > 5′ Pocket	Mean	Length of siRNA (nt)
D. melanogaster	61.0	59.6	60.3	~21 nt
T. castaneum	58.8	61.2	59.8	~21 nt
B. terrestris	61.7	62.7	62.2	~22 nt
L. migratoria	63.7	60.9	62.3	~22 nt
T. ni	53.6	63.1	58.3	~20 nt

## Data Availability

All sequence data used in this study are publicly available from NCBI GenBank and UniProt. Homology models, PyMOL session files, and measurement data are available from the corresponding author upon request.

## Supplementary Materials

The following supporting information can be downloaded at: https://www.mdpi.com/article/10.3390/v18030285/s1, we provide the results of homology modeling submitted to SWISS-MODEL workspace during the Dicer-2 molecular ruler project, in which the Dicer-2 from *Tribolium castaneum*, *Bombus terrestris*, *Locusta migratoria* and *Trichoplusia ni* were modeled. This file contains 8 documents with .html extension. Four files represent the reports of sequences modeled using Drosophila melanogaster Dicer-2 crystal structure (PDB: 7V6C) and Four files represent the reports of sequences modeled using Drosophila melanogaster Dicer-2 crystal structure (PDB: 7w0e). Figure S1: Alignments.

## Abbreviations

The following abbreviations are used in this manuscript:

APBS	Adaptive Poisson-Boltzmann Solver
Ago-2	Argonaute-2
dsRNA	Double-stranded RNA
MD	Molecular dynamics
RMSD	root mean square deviation
RISC	RNA-induced silencing complex
RNAi	RNA interference
siRNA	Small interfering RNA
Loqs-PD	Loquacious
VSR	viral suppressor of RNAi
PAZ	Piwi-Argonaute-Zwille
RNase III	Ribonuclease III
GMQE	Global Model Quality Estimation
QMEAN	Qualitative Model Energy ANalysis
