# Supplementary material for: Molecular Ruler Variation in Insect Dicer-2 Suggests a Structural Basis for Species-Dependent siRNA Length and Antiviral Defense Diversity
Source: Viruses. 2026 Feb 27;18(3):285. doi: 10.3390/v18030285 (PMC13030778; doi:10.3390/v18030285)
Supplement: Supplementary file 1 [file viruses-18-00285-s001.zip › Locusta_report_7V6C.html]

Locusta\_dicer\_2\_7v6c | Report


Homology Modelling Report

## Model Building Report

This document lists the results for the homology modelling project "Locusta\_dicer\_2\_7v6c" submitted to SWISS-MODEL workspace
on Dec. 28, 2025, 12:17 p.m..The submitted primary amino acid sequence is given in Table T1.

If you use any results in your research, please cite the relevant publications:

- Waterhouse A, Bertoni M, Bienert S, Studer G, Tauriello G, Gumienny R, Heer FT, de Beer TAP, Rempfer C, Bordoli L, Lepore R, Schwede T

  SWISS-MODEL: homology modelling of protein structures and complexes.

  Nucleic Acids Res 46, W296-W303. (2018) 2978835510.1093/nar/gky427
- Bienert S, Waterhouse A, de Beer TAP, Tauriello G, Studer G, Bordoli L, Schwede T

  The SWISS-MODEL Repository - new features and functionality.

  Nucleic Acids Res 45, D313-D319. (2017) 2789967210.1093/nar/gkw1132
- Studer G, Tauriello G, Bienert S, Biasini M, Johner N, Schwede T

  ProMod3 - A versatile homology modelling toolbox.

  PLOS Comp Biol 17(1), e1008667. (2021) 3350798010.1371/journal.pcbi.1008667
- Studer G, Rempfer C, Waterhouse AM, Gumienny R, Haas J, Schwede T

  QMEANDisCo - distance constraints applied on model quality estimation.

  Bioinformatics 36, 1765-1771. (2020) 3169731210.1093/bioinformatics/btz828
- Bertoni M, Kiefer F, Biasini M, Bordoli L, Schwede T

  Modeling protein quaternary structure of homo- and hetero-oligomers beyond binary interactions by homology.

  Scientific Reports 7. (2017) 2887468910.1038/s41598-017-09654-8

## Results

The SWISS-MODEL template library (SMTL version 2025-12-24, PDB release 2025-12-19) was searched with
BLAST (Camacho et al.) and HHblits (Steinegger et al.)
for evolutionary related structures matching the target sequence in Table T1. For details on the template search, see Materials and Methods. Overall 10851 templates were found (Table T2).

## Models

The following model was built (see Materials and Methods "Model Building"):

### Warning! The model had the following issues:

- Failed to close gap (A.VAL984-(LGHEISENG)-A.ASN994).
- Failed to close gap (A.LEU999-(NSDF)-A.SER1004).

  

| Model #01 | File | Built with | Oligo-State | Ligands | GMQE | QMEANDisCo Global |
| --- | --- | --- | --- | --- | --- | --- |
|  | PDB | ProMod3 3.6.0 | monomer | None | 0.60 | 0.58 ± 0.05 |

|  |  |  |
| --- | --- | --- |
|  |  |  |

| Template | Seq Identity | Oligo-state | QSQE | Found by | Method | Resolution | Seq Similarity | Range | Coverage | Description |
| --- | --- | --- | --- | --- | --- | --- | --- | --- | --- | --- |
| 7v6c.1.A | 28.25 | monomer | 0.00 | HHblits | EM | 3.30Å | 0.34 | 12 - 1413 | 0.98 | Dicer-2, isoform A |

  

### The template contained no ligands.

  

```
Target    MDTAPEKKQIAPRRYQEELLKRCLNENTILYLPTGSGKTFIAVMFIKKIMKECCVKYGRGKKLAVFAVNQVALAQQQTDY  
7v6c.1.A  ----MEDVEIKPRGYQLRLVDHLTKSNGIVYLPTGSGKTFVAILVLKRFSQDFDKPIESGGKRALFMCNTVELARQQAMA  
  
Target    ISRHVEMNVGCYIGIMNVDYWDKETWHKEFEKNQVLVMTAQILCNIIVHNILDVNRICVAVFDECHAATGNHPMKQAADN  
7v6c.1.A  VRRCTNFKVGFYVGEQGVDDWTRGMWSDEIKKNQVLVGTAQVFLDMVTQTYVALSSLSVVIIDECHHGTGHHPFREFMRL  
  
Target    ILKL--HINPRLLGLSGSLINGDCKVSRVVRCLKDLEDTFKCKIATAEET-LLPEVRRYSTNPEEEIHHYEGPVSD-TFT  
7v6c.1.A  FTIANQTKLPRVVGLTGVLIKGNE-ITNVATKLKELEITYRGNIITVSDTKELENVMLYATKPTEVMVSFPHQEQVLTVT  
  
Target    ENIQRLLTQCQNVIMASKVTTSILEKTKVPFGLIPMSDDMKQNKELKNIVENVKYQIDDLGLYGGYVATKMYIAALEKLA  
7v6c.1.A  RLISAEIEKFYVSLDLMNIGVQPIRRS----KSLQCLRDPSKKSFVKQLFNDFLYQMKEYGIYAASIAIISLIVEFDIKR  
  
Target    RRTDTVDALDLITVVKENLTEVRNGMEGQMMV--L------RNPHEQIRRFSSNKFMGFVSLIEEIFRGVHKTDQSELGN  
7v6c.1.A  RQAETLSVKLMHRTALTLCEKIRHLLVQKLQDMTYDDDDDNVNTEEVIMNFSTPKVQRFLMSLKVSFADK------DPKD  
  
Target    NNVLVFVKRRASARTLSALLKILSESDIRL-HTMKPDCVVGYGAQQSNEATEMHRKTNEDAILRFRKKETNLLVATDVLE  
7v6c.1.A  ICCLVFVERRYTCKCIYGLLLNYIQSTPELRNVLTPQFMVGRNNISPDFESVLERKWQKSAIQQFRDGNANLMICSSVLE  
  
Target    EGIDIPICNTVIMFDPPNSCRSYIQSKGRARHKTSSYHIFVCKSDT-KFLEKFNMYKAVGHEISKLLRPGAINVDVDMTD  
7v6c.1.A  EGIDVQACNHVFILDPVKTFNMYVQSKGRARTTEAKFVLFTADKEREKTIQQIYQYRKAHNDIAEYLKDRVLEKTEPELY  
  
Target    G--ESEDECLEWHTPFGSHAILKGPYAIQLVNMYSCKLPHDRFTHLAPLWYLLNR-----------DGKCICFVQLPVIS  
7v6c.1.A  EIKGHFQDDIDPFTN-ENGAVLLPNNALAILHRYCQTIPTDAFGFVIPWFHVLQEDERDRIFGVSAKGKHVISINMPVNC  
  
Target    VLKWTIEGEPQKNKRLAKQSAAIEAVKQLHACGELDDDMIPRPSEYIIY--HEKLFPYLTKEPIE-GSSPQQGSRQRKQP  
7v6c.1.A  MLRDTIYSDPMDNVKTAKISAAFKACKVLYSLGELNERFVPKTLKERVASIADVHFEHWNKYGDSVTATVNKADKSKDRT  
  
Target    YVKRCPLMYSDCRPQELQTTYLHVIRIEVDYPEPPVDSDDRILYEMFNEEEYFGMISSKEIPQEFSFPLYPAEGKLQVIL  
7v6c.1.A  YKTECPLEFYDALPRVGEICYAYEIFLEPQFES---CEYTEHMYLNLQTPRNYAILLRNKLPRLAEMPLFSNQGKLHVRV  
  
Target    MKTQ-KLYSLSKEEIRHCEEFHQFIFSKVLKSVKPFMSVDQERRDHSYLIVPVNKNENGAMQIDWNTMISTRNVSTNENI  
7v6c.1.A  ANAPLEVIIQNSEQLELLHQFHGMVFRDILKIWHPFFVLDRRSKENSYLVVPLILGAGEQKCFDWELMTNFRRLPQSHGS  
  
Target    K--M---QMNGQNRKFSAFSVFVHHSSSGKHKFYFVEEGSGGSNQNIEIDG----------------VHV--NRSPTASL  
7v6c.1.A  NVQQREQQPAPRPEDFEGKIVTQWYA--NYDKPMLVTKVHRELTPLSYMEKNQQDKTYYEFTMSKYGNRIGDVVHKDKFM  
  
Target    KEAIIISSDLNCLNQSRQL--QKNSQKRKIFFNPEVCDIVPLSASLCVKVHLLPSILHKLTLMATSYEIFQQIPVSKGSV  
7v6c.1.A  IEVRDLTEQLTFYVHNRGKFNAKSKAKMKVILIPELCFNFNFPGDLWLKLIFLPSILNRMYFLLHAEALRKRFNTYLNLH  
  
Target    -------------L--GH-----------EISE---------------N------------G-----------NDI--QL  
7v6c.1.A  LLPFNGTDYMPRPLEIDYSLKRNVDPLGNVIPTEDIEEPKSLLEPMPTKSIEASVANLEITEFENPWQKYMEPVDLSRNL  
  
Target    L---------------------N-SDF-----------------------------------------------------  
7v6c.1.A  LSTYPVELDYYYHFSVGNVCEMNEMDFEDKEYWAKNQFHMPTGNIYGNRTPAKTNANVPALMPSKPTVRGKVKPLLILQK  
  
Target    -----SGCGLTLNDIEQALTAASCDNIFNSETLETLGDSFLKFAVSLFLFIHYRNHHEGTLSTIKMKVVSNWHFYNVAKA  
7v6c.1.A  TVSKEHITPAEQGEFLAAITASSAADVFDMERLEILGDSFLKLSATLYLASKYSDWNEGTLTEVKSKLVSNRNLLFCLID  
  
Target    KDIGSKLQIHRFLPDETWVPPGFTVPQAVRRKFLS-----EHISPEYLKNLNLSGGKHLLDQL----------AGLEN--  
7v6c.1.A  ADIPKTLNTIQFTPRYTWLPPGISLPHNVLALWRENPEFAKIIGPHNLRDLALGDEESLVKGNCSDINYNRFVEGCRANG  
  
Target    -SWNLTK--LNNSELLIGVQTVSDKALADALEALTGVYLKAYGLYGATTLLNSLGVLPAKKASPHILFEK-PPESPLLQE  
7v6c.1.A  QSFYAGADFSSEVNFCVGLVTIPNKVIADTLEALLGVIVKNYGLQHAFKMLEYFKICRADIDKPLTQLLNLELGGKKMRA  
  
Target    SLPFQCLDFHLGDTDELEDTLNYKFKDRGFLLQALTHPSWSDNRITDCYQRLEFLGDAVLDFLVTSYIYDKCQKLSPGKI  
7v6c.1.A  NVNTTEIDGFLINHYYLEKNLGYTFKDRRYLLQALTHPSYPTNRITGSYQELEFIGDAILDFLISAYIFENNTKMNPGAL  
  
Target    TILRAALVNNGTFAAFAVRIGLQKYCKYRSSELFYNIDAFVKYQQENNHEIIDEVTC-----------------------  
7v6c.1.A  TDLRSALVNNTTLACICVRHRLHFFILAENAKLSEIISKFVNFQESQGHRVTNYVRILLEEADVQPTPLDLDDELDMTEL  
  
Target    ------------FGDDSDEENRILNSVPAPKVLGDLFESVAAAIYLDSGKCLKTVWSTYFKIMEKELARYCL  
7v6c.1.A  PHANKCISQEAEKGVPPKGEFNMSTNVDVPKALGDVLEALIAAVYLDCR-DLQRTWEVIFNLFEPELQEFT-
```

  


---

  

## Materials and Methods

## Template Search

Template search with BLAST and HHblits
has been performed against the SWISS-MODEL template library (SMTL, last update: 2025-12-24, last included PDB release: 2025-12-19).

The target sequence was searched with BLAST against the primary amino acid sequence contained in the SMTL.
A total of 255 templates were found.

An initial HHblits profile has been built using the procedure outlined in (Steinegger et al.), followed by 1 iteration of HHblits against Uniclust30 (Mirdita, von den Driesch et al.). The obtained profile has then be searched against all profiles of the SMTL. A total of 11018 templates were found.

## Model Building

Models are built based on the target-template alignment using ProMod3 (Studer et al.). Coordinates which are conserved between the target and the template are copied from the template to the model. Insertions and deletions are remodelled using a fragment library. Side chains are then rebuilt. Finally, the geometry of the resulting model is regularized by using a force field.

## Model Quality Estimation

The global and per-residue model quality has been assessed using the QMEAN scoring function (Studer et al.).

## Ligand Modelling

Ligands present in the template structure are transferred by homology to the model when the following criteria are met: (a) The ligands are annotated as biologically relevant in the template library, (b) the ligand is in contact with the model, (c) the ligand is not clashing with the protein, (d) the residues in contact with the ligand are conserved between the target and the template. If any of these four criteria is not satisfied, a certain ligand will not be included in the model. The model summary includes information on why and which ligand has not been included.

## Oligomeric State Conservation

The quaternary structure annotation of the template is used to model the target sequence in its oligomeric form. The method (Bertoni et al.) is based on a supervised machine learning algorithm, Support Vector Machines (SVM), which combines interface conservation, structural clustering, and other template features to provide a quaternary structure quality estimate (QSQE). The QSQE score is a number between 0 and 1, reflecting the expected accuracy of the interchain contacts for a model built based a given alignment and template. Higher numbers indicate higher reliability. This complements the GMQE score which estimates the accuracy of the tertiary structure of the resulting model.

## References

- Camacho C, Coulouris G, Avagyan V, Ma N, Papadopoulos J, Bealer K, Madden TL

  BLAST+: architecture and applications.

  BMC Bioinformatics, 10, 421-430. (2009) 2000350010.1186/1471-2105-10-421
- Steinegger M, Meier M, Mirdita M, Vöhringer H, Haunsberger SJ, Söding J

  HH-suite3 for fast remote homology detection and deep protein annotation.

  BMC Bioinformatics 20, 473. (2019) 3152111010.1186/s12859-019-3019-7
- Mirdita M, von den Driesch L, Galiez C, Martin MJ, Söding J, Steinegger M

  Uniclust databases of clustered and deeply annotated protein sequences and alignments.

  Nucleic Acids Res, 45, D170–D176. (2016) 2789957410.1093/nar/gkw1081

## Table T1:

Primary amino acid sequence for which templates were searched and models were built.

MDTAPEKKQIAPRRYQEELLKRCLNENTILYLPTGSGKTFIAVMFIKKIMKECCVKYGRGKKLAVFAVNQVALAQQQTDYISRHVEMNVGCYIGIMNVDY  
WDKETWHKEFEKNQVLVMTAQILCNIIVHNILDVNRICVAVFDECHAATGNHPMKQAADNILKLHINPRLLGLSGSLINGDCKVSRVVRCLKDLEDTFKC  
KIATAEETLLPEVRRYSTNPEEEIHHYEGPVSDTFTENIQRLLTQCQNVIMASKVTTSILEKTKVPFGLIPMSDDMKQNKELKNIVENVKYQIDDLGLYG  
GYVATKMYIAALEKLARRTDTVDALDLITVVKENLTEVRNGMEGQMMVLRNPHEQIRRFSSNKFMGFVSLIEEIFRGVHKTDQSELGNNNVLVFVKRRAS  
ARTLSALLKILSESDIRLHTMKPDCVVGYGAQQSNEATEMHRKTNEDAILRFRKKETNLLVATDVLEEGIDIPICNTVIMFDPPNSCRSYIQSKGRARHK  
TSSYHIFVCKSDTKFLEKFNMYKAVGHEISKLLRPGAINVDVDMTDGESEDECLEWHTPFGSHAILKGPYAIQLVNMYSCKLPHDRFTHLAPLWYLLNRD  
GKCICFVQLPVISVLKWTIEGEPQKNKRLAKQSAAIEAVKQLHACGELDDDMIPRPSEYIIYHEKLFPYLTKEPIEGSSPQQGSRQRKQPYVKRCPLMYS  
DCRPQELQTTYLHVIRIEVDYPEPPVDSDDRILYEMFNEEEYFGMISSKEIPQEFSFPLYPAEGKLQVILMKTQKLYSLSKEEIRHCEEFHQFIFSKVLK  
SVKPFMSVDQERRDHSYLIVPVNKNENGAMQIDWNTMISTRNVSTNENIKMQMNGQNRKFSAFSVFVHHSSSGKHKFYFVEEGSGGSNQNIEIDGVHVNR  
SPTASLKEAIIISSDLNCLNQSRQLQKNSQKRKIFFNPEVCDIVPLSASLCVKVHLLPSILHKLTLMATSYEIFQQIPVSKGSVLGHEISENGNDIQLLN  
SDFSGCGLTLNDIEQALTAASCDNIFNSETLETLGDSFLKFAVSLFLFIHYRNHHEGTLSTIKMKVVSNWHFYNVAKAKDIGSKLQIHRFLPDETWVPPG  
FTVPQAVRRKFLSEHISPEYLKNLNLSGGKHLLDQLAGLENSWNLTKLNNSELLIGVQTVSDKALADALEALTGVYLKAYGLYGATTLLNSLGVLPAKKA  
SPHILFEKPPESPLLQESLPFQCLDFHLGDTDELEDTLNYKFKDRGFLLQALTHPSWSDNRITDCYQRLEFLGDAVLDFLVTSYIYDKCQKLSPGKITIL  
RAALVNNGTFAAFAVRIGLQKYCKYRSSELFYNIDAFVKYQQENNHEIIDEVTCFGDDSDEENRILNSVPAPKVLGDLFESVAAAIYLDSGKCLKTVWST  
YFKIMEKELARYCL

## Table T2:

| Template | Seq Identity | Oligo-state | QSQE | Found by | Method | Resolution | Seq Similarity | Coverage | Description |
| --- | --- | --- | --- | --- | --- | --- | --- | --- | --- |
| 8yig.1.E | 28.15 | homo-dimer | 0.21 | HHblits | EM | NA | 0.34 | 0.98 | Dicer-2, isoform A |
| 8hf1.1.A | 28.15 | homo-trimer | 0.24 | HHblits | EM | NA | 0.34 | 0.98 | Dicer-2, isoform A |
| 7w0f.1.A | 28.22 | monomer | - | HHblits | EM | NA | 0.34 | 0.98 | Dicer-2, isoform A |
| 7xw3.1.A | 31.24 | monomer | - | HHblits | EM | NA | 0.35 | 0.98 | Endoribonuclease Dicer |
| 8hf1.1.F | 28.15 | homo-trimer | 0.24 | HHblits | EM | NA | 0.34 | 0.98 | Dicer-2, isoform A |
| 7v6c.1.A | 28.25 | monomer | - | HHblits | EM | 3.30Å | 0.34 | 0.98 | Dicer-2, isoform A |
| 7zpj.1.A | 30.97 | monomer | - | HHblits | EM | NA | 0.35 | 0.98 | Endoribonuclease Dicer |
| 7v6b.1.A | 28.42 | monomer | - | HHblits | EM | 3.30Å | 0.34 | 0.98 | Dicer-2, isoform A |
| 7yym.1.A | 30.82 | monomer | - | HHblits | EM | NA | 0.35 | 0.98 | Endoribonuclease Dicer |
| 8dga.1.A | 28.72 | monomer | - | HHblits | EM | NA | 0.34 | 0.98 | Endoribonuclease Dcr-1 |
| 7yz4.1.A | 30.82 | monomer | - | HHblits | EM | NA | 0.35 | 0.98 | Endoribonuclease Dicer |
| 8dfv.1.A | 28.72 | monomer | - | HHblits | EM | NA | 0.34 | 0.98 | Endoribonuclease Dcr-1 |
| 8dgi.1.A | 28.72 | monomer | - | HHblits | EM | NA | 0.34 | 0.98 | Endoribonuclease Dcr-1 |
| 8dg7.1.A | 28.72 | monomer | - | HHblits | EM | NA | 0.34 | 0.98 | Endoribonuclease Dcr-1 |
| 8dg5.1.A | 28.72 | monomer | - | HHblits | EM | NA | 0.34 | 0.98 | Endoribonuclease Dcr-1 |
| 8dgj.1.A | 28.72 | monomer | - | HHblits | EM | NA | 0.34 | 0.98 | Endoribonuclease Dcr-1 |
| 5zam.1.A | 31.24 | monomer | - | HHblits | EM | NA | 0.35 | 0.98 | Endoribonuclease Dicer |
| 5zak.1.A | 31.24 | monomer | - | HHblits | EM | NA | 0.35 | 0.98 | Endoribonuclease Dicer |
| 5zal.1.A | 31.24 | monomer | - | HHblits | EM | NA | 0.35 | 0.98 | Endoribonuclease Dicer |
| 7zpi.1.A | 30.97 | monomer | - | HHblits | EM | NA | 0.35 | 0.98 | Endoribonuclease Dicer |
| 7eld.1.A | 27.55 | monomer | - | HHblits | EM | NA | 0.33 | 0.92 | Endoribonuclease Dicer homolog 1 |
| 7ele.1.A | 27.55 | monomer | - | HHblits | EM | NA | 0.33 | 0.92 | Endoribonuclease Dicer homolog 1 |
| 6bua.1.A | 28.13 | monomer | - | HHblits | EM | NA | 0.34 | 0.98 | Dicer-2, isoform A |
| 7xw2.1.A | 31.24 | monomer | - | HHblits | EM | NA | 0.35 | 0.98 | Endoribonuclease Dicer |
| 7yyn.1.B | 30.58 | monomer | - | HHblits | EM | NA | 0.35 | 0.80 | Isoform 2 of Endoribonuclease Dicer |
| 7vg2.1.A | 23.23 | monomer | - | HHblits | EM | NA | 0.32 | 0.86 | Dicer-like 3 |
| 8hf1.1.D | 29.80 | homo-trimer | - | BLAST | EM | NA | 0.36 | 0.57 | Dicer-2, isoform A |
| 8yii.1.A | 29.80 | monomer | - | BLAST | EM | NA | 0.36 | 0.57 | Dicer-2, isoform A |
| 8yig.1.E | 29.80 | homo-dimer | - | BLAST | EM | NA | 0.36 | 0.57 | Dicer-2, isoform A |
| 8hf1.1.A | 29.80 | homo-trimer | - | BLAST | EM | NA | 0.36 | 0.57 | Dicer-2, isoform A |
| 8yih.1.B | 29.80 | monomer | - | BLAST | EM | NA | 0.36 | 0.57 | Dicer-2, isoform A |
| 8hf1.1.F | 29.80 | homo-trimer | - | BLAST | EM | NA | 0.36 | 0.57 | Dicer-2, isoform A |
| 7vg3.1.A | 23.23 | monomer | - | HHblits | EM | NA | 0.32 | 0.86 | Dicer-like 3 |
| 7v6c.1.A | 29.93 | monomer | - | BLAST | EM | 3.30Å | 0.36 | 0.57 | Dicer-2, isoform A |
| 9mx5.1.A | 35.50 | monomer | - | HHblits | EM | NA | 0.37 | 0.45 | AncD1D2 |
| 9mw8.1.A | 35.50 | monomer | - | HHblits | EM | NA | 0.37 | 0.45 | AncD1D2 |
| 7v6b.1.A | 29.93 | monomer | - | BLAST | EM | 3.30Å | 0.36 | 0.57 | Dicer-2, isoform A |
| 9mx3.1.A | 35.50 | monomer | - | HHblits | EM | NA | 0.37 | 0.45 | AncD1D2 |
| 9mx3.1.A | 40.78 | monomer | - | BLAST | EM | NA | 0.40 | 0.44 | AncD1D2 |
| 9mw6.1.A | 35.50 | monomer | - | HHblits | EM | 3.40Å | 0.37 | 0.45 | AncD1D2 |
| 9mx5.1.A | 40.78 | monomer | - | BLAST | EM | NA | 0.40 | 0.44 | AncD1D2 |
| 9mw8.1.A | 40.78 | monomer | - | BLAST | EM | NA | 0.40 | 0.44 | AncD1D2 |
| 9mw7.1.C | 35.50 | monomer | - | HHblits | EM | NA | 0.37 | 0.45 | AncD1D2 |
| 9mw6.1.A | 40.78 | monomer | - | BLAST | EM | 3.40Å | 0.40 | 0.44 | AncD1D2 |
| 9mw7.1.C | 40.78 | monomer | - | BLAST | EM | NA | 0.40 | 0.44 | AncD1D2 |
| 7eld.1.A | 30.77 | monomer | - | BLAST | EM | NA | 0.35 | 0.53 | Endoribonuclease Dicer homolog 1 |
| 7ele.1.A | 30.77 | monomer | - | BLAST | EM | NA | 0.35 | 0.53 | Endoribonuclease Dicer homolog 1 |
| 6bu9.1.A | 28.13 | monomer | - | HHblits | EM | NA | 0.34 | 0.98 | Dicer-2, isoform A |
| 9f1u.1.A | 20.83 | homo-dimer | - | HHblits | EM | NA | 0.29 | 0.43 | Interferon-induced helicase C domain-containing protein 1 |
| 9f0j.1.A | 20.83 | homo-dimer | - | HHblits | EM | NA | 0.29 | 0.43 | Interferon-induced helicase C domain-containing protein 1 |

  
The table above shows the top 50 filtered templates. A further 8,907 templates were found which were considered to be less suitable for modelling than the filtered list.  
1a1v.1.A, 1a5t.1.A, 1ak2.1.A, 1b7t.1.A, 1br1.1.A, 1br2.1.A, 1br4.1.A, 1c4o.1.A, 1cr0.1.A, 1cu1.1.A, 1cu1.1.B, 1d0x.1.A, 1d2m.1.A, 1d6j.1.A, 1d6j.1.B, 1d9x.1.A, 1d9z.1.A, 1dfk.1.A, 1dfl.1.A, 1di2.1.C, 1di2.1.D, 1do0.1.A, 1do0.1.B, 1do0.1.C, 1do0.1.F, 1do2.1.C, 1do2.3.B, 1dvr.1.A, 1dvr.1.B, 1e0j.1.A, 1e0j.1.B, 1e0j.2.A, 1e1q.1.A, 1e1r.1.A, 1e1r.1.B, 1e1r.1.C, 1e2d.1.A, 1e32.1.B, 1e6c.1.A, 1e94.3.D, 1e98.1.A, 1e9c.1.A, 1efr.1.A, 1efr.1.B, 1efr.1.C, 1efr.1.D, 1efr.1.E, 1ffh.1.A, 1fl9.1.A, 1fl9.2.A, 1fl9.3.A, 1fmv.1.A, 1fnn.1.A, 1fts.1.A, 1fuk.1.A, 1fuu.1.A, 1fx0.1.C, 1g3i.1.B, 1g3i.1.S, 1g3i.1.V, 1g41.1.C, 1g4a.1.H, 1g4a.1.M, 1g4b.1.B, 1g5t.1.B, 1g64.1.A, 1g8p.1.A, 1g8y.1.D, 1gki.1.D, 1gku.1.A, 1gl6.1.A, 1gl7.1.C, 1gl9.1.A, 1gm5.1.A, 1h65.1.A, 1h65.2.B, 1h8e.1.E, 1hei.1.A, 1hei.1.B, 1hqc.1.A, 1hqc.2.A, 1htw.1.A, 1htw.2.A, 1htw.3.A, 1hv8.1.A, 1hv8.1.B, 1i4s.1.A, 1i4s.1.B, 1im2.1.F, 1in4.1.A, 1in5.1.A, 1in6.1.A, 1in7.1.A, 1in8.1.A, 1iqp.1.A, 1iqp.1.B, 1iqp.1.C, 1iqp.1.D, 1iqp.1.E, 1iqp.1.F, 1ixr.1.C, 1ixs.1.B, 1ixz.1.A, 1iy2.1.A, 1j7k.1.A, 1j8m.1.A, 1j8y.1.A, 1j90.1.A, 1j90.1.B, 1jah.1.A, 1jb1.1.D, 1jbk.1.A, 1jfz.1.A, 1jfz.1.B, 1jfz.2.A, 1jfz.2.B, 1jpj.1.A, 1jpn.1.A, 1jpn.2.A, 1jr3.1.A, 1jr3.1.B, 1jr3.1.C, 1jr3.1.E, 1jr6.1.A, 1kag.1.A, 1kag.2.A, 1kao.1.A, 1kgd.1.A, 1ki9.2.A, 1ki9.2.E, 1ki9.2.F, 1kk7.1.A, 1kk8.1.A, 1kkl.1.C, 1kkl.1.H, 1knq.1.B, 1ko1.1.B, 1ko5.1.B, 1ko8.1.A, 1ko8.1.B, 1kqm.1.A, 1ksf.1.A, 1kwo.1.A, 1kyi.1.C, 1l2o.1.A, 1l8q.1.A, 1lkx.1.A, 1lkx.3.A, 1ls1.1.A, 1lv7.1.A, 1lvk.1.A, 1m6n.1.A, 1m7g.2.B, 1m7g.4.A, 1m7h.3.C, 1m8q.1.A, 1m8q.1.D, 1m8q.1.G, 1m8q.1.J, 1mab.1.A, 1mab.1.D, 1mma.1.A, 1mmd.1.A, 1mmg.1.A, 1mmn.1.A, 1mnd.1.A, 1mvw.1.M, 1mvw.1.P, 1n0w.1.A, 1n25.1.A, 1nbm.1.D, 1nbm.1.E, 1njf.1.A, 1njf.2.A, 1njf.3.A, 1njf.4.A, 1njg.1.A, 1njg.2.A, 1nks.1.A, 1nks.1.B, 1nks.1.C, 1nkt.1.A, 1nl3.1.B, 1nlf.1.B, 1nly.1.E, 1nlz.1.B, 1nlz.1.C, 1nlz.1.E, 1np6.1.A, 1np6.1.B, 1nsf.1.A, 1ny6.1.A, 1ny6.1.B, 1ny6.1.C, 1ny6.1.E, 1ny6.1.F, 1ny6.1.G, 1ny6.2.A, 1ny6.2.B, 1ny6.2.E, 1ny6.2.F, 1ny6.2.G, 1o0w.1.A, 1o18.1.K, 1o18.1.N, 1o19.1.M, 1o19.1.P, 1o1a.1.M, 1o1a.1.P, 1o1b.1.G, 1o1b.1.J, 1o1c.1.J, 1o1c.1.M, 1o1d.1.M, 1o1d.1.P, 1o1e.1.M, 1o1f.1.D, 1o1g.1.M, 1o1g.1.P, 1o87.1.A, 1o87.2.A, 1oe0.1.B, 1oe9.1.A, 1ofh.1.A, 1ofh.2.A, 1ofh.2.B, 1ofi.1.G, 1ohh.1.C, 1ohh.1.D, 1ojl.1.A, 1ojl.1.C, 1ojl.2.A, 1ojl.2.B, 1okk.1.A, 1olo.1.A, 1olo.1.D, 1onb.1.A, 1oyy.1.A, 1p3j.1.A, 1p60.3.B, 1p9n.1.A, 1p9n.1.B, 1p9r.1.A, 1plk.1.B, 1pvo.1.A, 1pvo.1.C, 1pvo.1.D, 1pvo.1.E, 1pvo.1.F, 1pzn.1.A, 1pzn.1.D, 1q0u.1.A, 1q0u.2.A, 1q3t.1.A, 1q57.1.A, 1q57.1.B, 1q57.1.C, 1q57.1.D, 1q57.1.E, 1q57.1.F, 1q57.1.G, 1qde.1.A, 1qhg.1.A, 1qhh.1.A, 1qhn.1.D, 1qhy.1.D, 1qu6.1.A, 1qva.1.A, 1qvi.1.A, 1qvr.1.A, 1qvr.1.B, 1qvr.1.C, 1qzw.1.B, 1qzx.1.A, 1r6b.1.A, 1r7r.1.A, 1rc7.1.E, 1rif.1.A, 1rif.2.A, 1rj9.1.B, 1rkb.1.A, 1rz3.1.A, 1s2m.1.A, 1s3g.1.A, 1s3s.1.A, 1s3s.1.B, 1s3s.1.C, 1s3s.1.D, 1s3s.1.E, 1s3s.1.F, 1s9h.1.A, 1s9h.2.A, 1s9h.3.A, 1shk.3.A, 1shk.5.B, 1sky.1.A, 1sky.1.B, 1sr6.1.A, 1stu.1.A, 1svl.1.A, 1svl.1.B, 1svl.1.F, 1svm.1.A, 1svm.1.B, 1svo.1.A, 1sxj.1.A, 1sxj.1.B, 1sxj.1.C, 1sxj.1.D, 1sxj.1.E, 1szp.1.A, 1szp.1.B, 1t4l.1.B, 1t4n.1.A, 1t4o.1.A, 1t4o.2.A, 1t5i.1.A, 1t5l.1.A, 1t5l.2.A, 1t6n.1.A, 1t6n.1.B, 1tf2.1.A, 1tf7.1.A, 1tf7.1.F, 1tue.1.A, 1tue.2.A, 1tue.5.A, 1u0j.1.A, 1u61.1.A, 1u94.1.A, 1u98.1.A, 1uaa.1.B, 1uaa.1.C, 1ubc.1.A, 1ubf.1.A, 1ubg.1.A, 1uei.1.A, 1ufq.1.A, 1ufq.1.B, 1uil.1.A, 1uj2.1.C, 1um8.1.A, 1v5w.1.A, 1vec.1.A, 1vec.2.A, 1via.1.A, 1vma.1.A, 1vom.1.A, 1w2h.1.A, 1w2h.1.B, 1w36.1.C, 1w4r.1.A, 1w4r.1.B, 1w5s.1.A, 1w5s.2.A, 1w5t.1.A, 1w5t.3.A, 1w7j.1.A, 1w8j.1.A, 1w8j.3.A, 1w8j.4.A, 1w9i.1.A, 1w9j.1.A, 1w9k.1.A, 1w9l.1.A, 1we2.1.A, 1whn.1.A, 1whq.1.A, 1wp9.1.A, 1wp9.2.A, 1wrb.1.A, 1wrb.2.A, 1x49.1.A, 1xew.1.A, 1xex.1.C, 1xjc.1.A, 1xmv.1.A, 1xpo.1.A, 1xpo.2.A, 1xpo.3.A, 1xpo.4.A, 1xpo.5.A, 1xpo.6.A, 1xpr.1.A, 1xpr.2.A, 1xpr.3.A, 1xpr.4.A, 1xpr.5.A, 1xpr.6.A, 1xrj.1.A, 1xti.1.A, 1xtj.1.A, 1xtk.1.A, 1xu4.1.A, 1xwi.1.A, 1xx6.1.A, 1xxh.1.B, 1xxh.1.C, 1xxh.1.D, 1xxh.1.E, 1xxh.2.B, 1xxh.2.C, 1xxh.2.D, 1xxi.1.B, 1xxi.1.C, 1xxi.1.D, 1xxi.1.E, 1xxi.2.B, 1y63.1.A, 1ye8.1.A, 1yks.1.A, 1yv3.1.A, 1yyf.1.B, 1yyf.1.U, 1yyk.1.E, 1yyk.1.F, 1yyo.1.E, 1yyo.1.F, 1yyw.1.E, 1yyw.1.F, 1yyw.2.E, 1yyw.2.F, 1yz9.1.E, 1yz9.1.F, 1yzk.1.A, 1z3i.1.A, 1z5z.1.A, 1z5z.2.A, 1z63.2.C, 1z6a.3.A, 1z6g.1.A, 1zd8.1.A, 1zm7.1.A, 1zmx.1.A, 1ztd.1.A, 1ztd.1.B, 1zu4.1.A, 1zu5.1.A, 1zuh.1.A, 1zui.1.A, 1zyu.1.A, 2a11.1.A, 2a2z.1.A, 2a7q.1.B, 2aka.1.A, 2ar7.1.A, 2ar7.2.A, 2ax4.1.A, 2b21.1.A, 2b7t.1.A, 2b7v.1.A, 2b8t.1.A, 2b8t.1.C, 2b8t.1.D, 2bbw.1.A, 2bbw.2.A, 2bhr.1.A, 2bjw.1.A, 2bkh.1.A, 2bki.1.A, 2bmf.1.A, 2bmf.2.A, 2c03.1.A, 2c03.2.A, 2c04.1.A, 2c04.2.A, 2c9c.1.A, 2c9o.1.D, 2c9o.2.A, 2c9o.2.F, 2c9y.1.A, 2ccg.1.A, 2cdn.1.A, 2ce7.1.A, 2ce7.1.B, 2ce7.1.C, 2ce7.2.A, 2ce7.2.B, 2ce7.2.C, 2chg.1.A, 2chq.1.A, 2chq.1.B, 2chq.1.C, 2chv.1.C, 2chv.1.E, 2ck3.1.B, 2ck3.1.C, 2cnw.1.A, 2cnw.3.A, 2cvf.1.A, 2cvf.2.A, 2cvh.1.A, 2cvh.2.A, 2d7d.1.A, 2db2.1.A, 2db3.1.B, 2dfl.1.A, 2dfn.1.A, 2dft.1.A, 2dft.1.B, 2dft.1.C, 2dhr.1.A, 2dhr.1.B, 2dmy.1.A, 2dr3.1.A, 2dr3.1.B, 2dr3.1.C, 2dr3.1.D, 2dr3.1.E, 2dr3.1.F, 2eb1.1.A, 2eb1.1.B, 2ec6.1.A, 2eu8.1.A, 2eu8.2.A, 2ewv.1.E, 2eyq.1.A, 2eyq.2.A, 2eyu.1.A, 2eyu.2.A, 2ez6.1.C, 2f1h.1.A, 2f1i.1.A, 2f1r.1.A, 2f1r.1.B, 2f43.1.A, 2f55.1.B, 2f55.1.C, 2f55.2.A, 2fdc.1.B, 2fdc.2.B, 2ffh.1.A, 2fna.1.A, 2fsh.1.A, 2fsh.1.B, 2fwr.1.A, 2fwr.2.A, 2fwr.3.A, 2fwr.4.A, 2fz4.1.A, 2fzl.1.A, 2g1j.1.A, 2g1j.2.A, 2g1k.1.A, 2g88.1.A, 2g9n.1.A, 2g9n.2.A, 2gaa.1.A, 2gbl.1.C, 2gbl.1.F, 2gdj.1.A, 2gjk.1.A, 2gk6.1.A, 2gk7.1.A, 2gks.1.A, 2gks.1.B, 2gno.1.A, 2grj.1.A, 2gsl.1.A, 2gsl.2.A, 2gsl.2.B, 2gsl.3.A, 2gsl.3.B, 2gsz.1.A, 2gsz.1.B, 2gxa.1.B, 2gxa.1.C, 2gxa.1.F, 2gxa.1.G, 2gxa.2.D, 2gxa.2.G, 2gxq.1.A, 2gxs.1.A, 2gxs.1.B, 2gza.1.A, 2gza.1.C, 2h1l.1.A, 2h1l.1.B, 2h1l.1.C, 2h92.1.A, 2hcb.1.A, 2hcb.1.B, 2hcb.1.C, 2hcb.1.D, 2hf9.2.B, 2hjv.1.A, 2hjv.2.A, 2hld.1.C, 2hld.1.D, 2hld.3.A, 2hld.3.B, 2hld.3.E, 2hld.3.F, 2ht1.1.A, 2ht1.1.B, 2hxy.1.A, 2hxy.3.A, 2hyi.1.C, 2i4i.1.A, 2ibm.3.C, 2ibm.3.D, 2ipc.1.A, 2ipc.1.B, 2is1.1.C, 2is1.2.C, 2is2.1.C, 2is4.1.C, 2is4.1.D, 2is6.1.D, 2ius.1.A, 2iut.1.A, 2iuu.1.B, 2iyq.1.A, 2iyr.1.A, 2iyr.2.A, 2iys.1.A, 2iyv.1.A, 2iyx.1.A, 2j0q.1.A, 2j0u.1.A, 2j0u.2.A, 2j28.1.H, 2j37.1.G, 2j3e.1.A, 2j41.1.A, 2j41.1.B, 2j45.1.A, 2j45.2.A, 2j46.1.A, 2j46.2.A, 2j7p.2.A, 2j87.1.A, 2j87.1.B, 2j9r.1.A, 2ja1.1.A, 2jas.1.A, 2jat.1.A, 2jcs.1.A, 2jdi.1.F, 2jeo.1.A, 2jgn.1.A, 2jgn.2.A, 2jiz.1.A, 2jj1.1.B, 2jj2.2.C, 2jj8.1.A, 2jj9.1.A, 2jlq.1.A, 2jlr.1.A, 2jls.1.A, 2jlu.1.A, 2jlv.2.A, 2jly.1.A, 2kbe.1.A, 2kbf.1.A, 2khx.1.A, 2kjq.1.A, 2kou.1.A, 2l2k.1.A, 2l2n.1.A, 2l3c.1.A, 2l3j.1.A, 2l8b.1.A, 2lbs.1.B, 2ljh.1.A, 2lrs.1.A, 2ltr.1.A, 2lts.1.A, 2lup.1.B, 2luq.1.A, 2mys.1.A, 2n3f.1.A, 2n3g.1.A, 2n3h.1.A, 2na2.1.A, 2ng1.1.A, 2npi.1.A, 2nue.1.B, 2nue.1.C, 2nuf.1.C, 2nuf.1.D, 2nug.1.E, 2o0j.1.A, 2o0k.1.A, 2oaq.1.B, 2oaq.1.E, 2oca.1.A, 2odn.1.A, 2oe2.1.A, 2oep.1.A, 2ofo.1.A, 2ofw.1.A, 2ofw.1.B, 2ofx.1.A, 2og2.1.A, 2oo7.1.A, 2ori.1.A, 2ori.1.B, 2orv.1.A, 2orw.1.A, 2os8.1.A, 2otg.1.A, 2oxc.1.A, 2p3s.1.A, 2p65.1.A, 2p67.1.A, 2p6n.1.A, 2p6r.1.C, 2p6u.1.A, 2pey.1.A, 2pey.1.B, 2pjr.1.A, 2pjr.1.C, 2pl3.1.A, 2pt5.1.A, 2pt5.2.A, 2pt5.3.A, 2pt5.4.A, 2pt7.1.B, 2pt7.1.D, 2pt7.1.F, 2px0.1.A, 2px0.3.B, 2px0.4.A, 2px0.4.B, 2px3.1.A, 2q6t.1.A, 2q6t.2.A, 2q6t.3.A, 2q6t.4.A, 2qa5.1.A, 2qag.1.C, 2qag.1.E, 2qaj.1.A, 2qby.1.C, 2qby.1.D, 2qe7.1.B, 2qe7.1.D, 2qeq.1.A, 2qeq.2.A, 2qgn.1.A, 2qgz.1.A, 2ql6.8.A, 2qnr.1.A, 2qor.1.A, 2qp9.1.A, 2qpa.1.A, 2qpa.2.A, 2qpa.3.A, 2qpo.1.A, 2qpo.1.B, 2qpo.1.D, 2qsy.1.A, 2qt0.1.A, 2qvw.1.A, 2qvw.3.A, 2qz4.1.A, 2r2a.1.A, 2r44.1.A, 2r62.1.A, 2r62.2.A, 2r65.1.A, 2r65.2.A, 2r65.3.A, 2r65.4.A, 2r65.5.A, 2r6a.1.B, 2r6a.1.D, 2r6c.1.A, 2r6c.1.B, 2r6c.1.C, 2r6c.1.D, 2r6c.1.F, 2r6c.1.N, 2r6d.1.A, 2r6d.1.B, 2r6d.1.C, 2r6d.1.D, 2r6d.1.E, 2r6d.1.F, 2r6e.1.A, 2r6e.1.F, 2r8r.1.A, 2r8r.2.A, 2r9v.1.A, 2rap.1.A, 2rb4.1.A, 2reb.1.A, 2rko.1.A, 2rs6.1.A, 2rs7.1.A, 2v1u.1.A, 2v1x.1.A, 2v26.1.A, 2v3c.1.B, 2v3c.2.B, 2v6i.1.A, 2v6j.1.A, 2v7q.1.A, 2v7q.1.B, 2v7q.1.C, 2v7q.1.E, 2v8o.1.A, 2v9p.1.A, 2v9p.1.D, 2v9p.2.E, 2v9p.2.F, 2va8.1.A, 2va8.2.A, 2vas.1.A, 2vb6.1.A, 2vbc.1.A, 2vda.1.A, 2vii.1.A, 2vl7.1.A, 2vpp.1.B, 2vsf.1.A, 2vso.1.A, 2vye.1.A, 2vye.1.D, 2vyf.1.B, 2vyf.1.C, 2w00.2.A, 2w0m.1.A, 2w4a.3.A, 2w4g.1.C, 2w4h.1.C, 2w4t.1.A, 2w4v.1.A, 2w4w.1.A, 2w58.1.A, 2w58.1.B, 2w6e.1.A, 2w6e.1.B, 2w6e.1.C, 2w6e.1.D, 2w6e.1.E, 2w6j.1.A, 2w6j.1.B, 2w6j.1.C, 2w6j.1.F, 2wax.1.A, 2wax.2.A, 2way.1.A, 2whx.1.A, 2wjv.1.A, 2wjy.1.A, 2wpd.1.B, 2wsm.1.A, 2wss.1.A, 2wss.1.B, 2wss.1.C, 2wv9.1.A, 2www.2.B, 2wwy.1.B, 2wzq.1.A, 2x31.1.G, 2x31.1.H, 2x51.1.A, 2x8a.1.A, 2x9h.1.A, 2xau.1.A, 2xb2.2.A, 2xb4.1.A, 2xel.1.A, 2xfm.1.A, 2xgj.1.A, 2xgj.2.A, 2xnd.1.A, 2xnd.1.B, 2xnd.1.C, 2xnd.1.D, 2xnd.1.E, 2xnd.1.F, 2xo8.1.A, 2xok.1.A, 2xok.1.B, 2xok.1.D, 2xok.1.E, 2xok.1.F, 2xsz.1.A, 2xsz.1.D, 2xx3.1.A, 2xxa.1.A, 2xxa.2.A, 2xzl.1.A, 2xzo.1.A, 2xzp.1.A, 2y0r.1.A, 2y8i.1.A, 2y9e.1.A, 2yjt.1.D, 2ykg.1.A, 2yof.1.A, 2yof.3.A, 2yoh.1.A, 2yoh.2.A, 2yt4.1.A, 2z0m.1.A, 2z43.1.A, 2z43.2.A, 2z4r.1.A, 2z83.1.A, 2zam.1.A, 2zan.1.A, 2zao.1.A, 2ze5.1.A, 2zej.1.A, 2zej.1.B, 2zj5.2.B, 2zj8.2.A, 2zjb.1.A, 2zjo.1.A, 2zpa.1.A, 2zpa.2.A, 2zr0.1.A, 2zra.1.A, 2zrb.1.A, 2zrc.1.A, 2zrg.1.A, 2zrh.1.A, 2zrl.1.A, 2zrm.1.A, 2zro.1.A, 2zts.1.A, 2zu6.1.C, 2zu6.2.C, 2zub.1.A, 2zub.1.B, 2zuc.1.A, 2zuc.1.B, 2zud.1.A, 2zud.1.B, 2zxu.3.A, 2zxu.3.B, 3a8t.1.A, 3adb.1.A, 3adb.1.B, 3adc.1.A, 3adc.1.B, 3add.1.B, 3adg.1.A, 3adi.1.A, 3adi.2.A, 3adi.3.A, 3adk.1.A, 3adl.1.A, 3asy.1.A, 3asy.1.B, 3b6e.1.A, 3b85.1.A, 3b9p.1.A, 3b9q.1.A, 3baf.1.A, 3bb3.1.A, 3be4.1.A, 3ber.1.A, 3bgw.1.A, 3bgw.1.B, 3bgw.1.C, 3bgw.1.D, 3bgw.1.E, 3bgw.1.F, 3bh0.1.A, 3bor.1.A, 3bos.1.A, 3bos.1.B, 3bs4.1.A, 3bxz.1.A, 3c4b.1.A, 3c4t.1.A, 3cf0.1.A, 3cf1.1.C, 3cf1.1.D, 3cf2.2.D, 3cf3.1.A, 3cf3.1.F, 3ch4.1.A, 3cmt.1.B, 3cmt.2.B, 3co5.1.A, 3cpe.1.A, 3cr7.1.A, 3cr7.1.B, 3crm.1.A, 3crv.1.A, 3crw.1.A, 3d3q.1.A, 3d3q.2.A, 3d8b.1.A, 3d8b.1.B, 3def.1.A, 3din.1.A, 3dkp.1.A, 3dl0.1.A, 3dl8.1.A, 3dm5.1.A, 3dmd.1.A, 3dmd.1.D, 3dmd.3.A, 3e1s.1.A, 3e2i.1.A, 3e70.1.A, 3eaq.1.A, 3eaq.1.B, 3ear.1.A, 3eas.1.A, 3eas.1.B, 3ec2.1.A, 3ecc.1.A, 3eie.1.A, 3eih.1.A, 3eih.2.A, 3eih.3.A, 3eiq.1.A, 3eiq.1.C, 3eph.1.A, 3epl.1.A, 3epl.1.C, 3etl.1.A, 3ews.1.A, 3ews.2.A, 3ex7.2.C, 3exa.1.A, 3exa.2.B, 3f9v.1.A, 3fb4.1.A, 3fe2.1.A, 3fhc.1.B, 3fho.1.A, 3fht.1.A, 3fks.1.C, 3fks.1.D, 3fks.1.E, 3fks.1.F, 3fks.2.B, 3fks.2.D, 3fks.3.A, 3fks.3.D, 3fmo.1.B, 3fmp.1.B, 3foz.1.B, 3ftq.1.A, 3ftq.2.A, 3ftq.3.A, 3ftq.4.A, 3g0h.1.A, 3gfp.1.A, 3glf.1.B, 3glf.1.C, 3glf.1.D, 3glg.1.B, 3glg.1.C, 3glg.1.D, 3glh.1.B, 3glh.1.C, 3glh.1.D, 3glh.2.B, 3glh.3.B, 3gli.1.B, 3gli.1.E, 3gp8.1.A, 3gpl.1.A, 3h1t.1.A, 3h4m.1.A, 3h4m.2.A, 3h4m.3.A, 3h86.1.A, 3h86.1.B, 3h86.1.C, 3h86.2.A, 3hdt.1.A, 3hdt.1.B, 3hgt.1.B, 3hr7.1.A, 3hr7.1.B, 3hr8.1.A, 3hte.1.A, 3hte.1.B, 3hte.1.C, 3hte.1.D, 3hu1.1.A, 3hu2.1.A, 3hu3.1.F, 3hws.1.C, 3hws.1.F, 3hws.2.B, 3hws.3.A, 3hws.3.C, 3i32.1.A, 3i5f.1.A, 3i5g.1.A, 3i62.1.A, 3ice.1.A, 3ice.1.B, 3ice.1.C, 3ice.1.D, 3ice.1.E, 3ice.1.F, 3iij.1.A, 3io5.1.A, 3iqm.1.A, 3iqy.1.A, 3iuy.1.A, 3j04.1.A, 3j04.1.D, 3j3r.1.G, 3j3r.1.H, 3j3r.1.I, 3j3r.1.J, 3j3r.1.K, 3j3r.1.L, 3j3s.1.G, 3j3s.1.H, 3j3s.1.I, 3j3s.1.J, 3j3s.1.K, 3j3s.1.L, 3j3t.1.G, 3j3t.1.H, 3j3t.1.I, 3j3t.1.J, 3j3t.1.K, 3j3t.1.L, 3j3u.1.G, 3j3u.1.H, 3j3u.1.I, 3j3u.1.J, 3j3u.1.K, 3j3u.1.L, 3j67.1.A, 3j6b.1.5, 3j6b.1.9, 3j7y.1.a, 3j94.1.A, 3j94.1.B, 3j94.1.C, 3j94.1.D, 3j94.1.E, 3j94.1.F, 3j95.1.A, 3j95.1.B, 3j95.1.C, 3j95.1.D, 3j95.1.E, 3j95.1.F, 3j96.1.A, 3j96.1.E, 3j96.1.F, 3j97.1.A, 3j97.1.B, 3j97.1.C, 3j97.1.D, 3j97.1.E, 3j97.1.F, 3j98.1.A, 3j98.1.B, 3j98.1.D, 3j98.1.E, 3j98.1.F, 3j99.1.A, 3j99.1.D, 3j99.1.E, 3j99.1.F, 3jb9.1.5, 3jbl.1.A, 3jcm.1.I, 3jco.1.Q, 3jco.1.R, 3jco.1.S, 3jco.1.T, 3jco.1.U, 3jco.1.V, 3jcp.1.Q, 3jcp.1.R, 3jcp.1.S, 3jcp.1.T, 3jcp.1.U, 3jcp.1.V, 3jd5.1.W, 3jux.1.A, 3jv2.1.A, 3jv2.2.A, 3jvu.1.A, 3jvu.1.B, 3jvu.1.C, 3jvv.1.A, 3jvv.1.B, 3jvv.1.C, 3jzm.1.A, 3jzm.1.F, 3k09.1.A, 3k09.1.B, 3k09.1.E, 3k09.1.F, 3k0a.1.A, 3k0a.1.B, 3k0a.1.C, 3k0a.1.F, 3k0c.1.A, 3k0c.1.B, 3k0c.1.D, 3k0c.1.F, 3k0e.1.A, 3k0e.1.B, 3k0e.1.C, 3k0e.1.D, 3k0e.1.E, 3k0e.1.F, 3k0f.1.A, 3k0f.1.B, 3k0f.1.E, 3k0f.1.F, 3k1j.1.A, 3k1j.1.B, 3k70.1.A, 3k70.2.A, 3k70.2.C, 3kds.1.A, 3kl4.1.A, 3kqh.1.A, 3kql.1.A, 3kqu.1.B, 3kta.1.A, 3kta.2.A, 3kx2.2.A, 3l0o.1.A, 3l0o.1.B, 3l0p.1.A, 3l9i.1.A, 3ld9.1.A, 3lda.1.A, 3lfu.1.A, 3llh.1.A, 3llh.2.A, 3llm.1.A, 3llm.1.B, 3lnc.1.A, 3ly5.1.A, 3m0e.1.A, 3m6a.1.A, 3m6a.1.D, 3m6a.1.E, 3m6a.1.F, 3mkd.1.A, 3mle.3.A, 3mle.3.B, 3mnq.1.A, 3mrs.1.A, 3muf.1.A, 3mwj.1.A, 3mwj.1.B, 3mwy.1.A, 3myk.1.A, 3myl.1.A, 3n3w.1.A, 3n70.1.A, 3n70.2.A, 3n70.2.B, 3n70.3.A, 3n70.4.A, 3n70.5.A, 3n70.5.B, 3n70.7.A, 3nbf.1.A, 3nbf.2.A, 3nbx.1.A, 3ndb.1.B, 3ney.1.A, 3ney.2.A, 3ng1.1.A, 3ntu.1.A, 3nwj.1.A, 3nwj.2.A, 3o2r.1.A, 3o2r.2.B, 3o7x.1.A, 3o7x.2.B, 3o8b.1.A, 3o8b.2.A, 3o8c.1.A, 3o8r.1.A, 3o8r.1.B, 3oaa.2.A, 3oaa.4.B, 3oaa.4.C, 3oe7.1.D, 3oee.1.A, 3oee.1.B, 3oee.1.C, 3oee.3.F, 3oeh.1.B, 3oeh.1.D, 3oeh.1.E, 3oeh.2.B, 3oeh.2.F, 3oeh.3.C, 3ofn.1.A, 3ofn.1.B, 3ofn.2.F, 3ofn.3.C, 3oiy.1.A, 3oiy.2.A, 3p4x.1.A, 3p4x.2.A, 3p4y.1.A, 3pev.2.B, 3pew.1.A, 3pey.1.A, 3pfi.1.A, 3pfi.1.B, 3pjr.1.C, 3pqc.1.A, 3pqc.2.A, 3pr1.1.A, 3pvs.1.A, 3pvs.1.B, 3pvs.1.C, 3pvs.1.D, 3pxg.1.B, 3pxg.1.D, 3pxg.1.F, 3pxg.1.J, 3pxi.1.B, 3pxi.1.D, 3pxi.1.F, 3qir.1.A, 3qir.2.B, 3qir.3.A, 3qkr.1.A, 3qks.1.A, 3qmz.1.A, 3r8f.1.A, 3rap.1.A, 3rap.1.B, 3rc3.1.A, 3rc8.1.A, 3rrm.1.A, 3rrn.1.A, 3rv0.1.A, 3rv0.1.B, 3rv0.2.A, 3rv0.2.B, 3rv1.1.A, 3rv1.1.B, 3rvb.1.A, 3s1a.1.A, 3s1a.1.B, 3s1a.1.E, 3s1a.1.F, 3sc3.1.A, 3sc3.1.B, 3sfz.1.A, 3sja.2.B, 3sjb.1.A, 3sjb.1.B, 3sjc.2.A, 3sjd.2.A, 3sop.1.A, 3sqw.1.A, 3sqx.1.A, 3syl.2.A, 3syl.2.B, 3syn.5.E, 3t15.1.A, 3t5d.1.A, 3tau.1.A, 3tau.1.B, 3tbk.1.A, 3te6.1.A, 3te6.2.A, 3tk1.1.A, 3tlx.1.A, 3tlx.3.A, 3tlx.4.A, 3tqf.1.A, 3trf.1.A, 3tw4.1.A, 3u44.1.A, 3u4q.1.B, 3u5z.1.A, 3u5z.2.A, 3u5z.2.B, 3u5z.2.D, 3u60.1.A, 3u60.1.B, 3u60.1.C, 3u60.1.D, 3u61.1.A, 3u61.1.B, 3u61.1.C, 3u61.1.D, 3ua2.1.A, 3ua2.1.B, 3ua2.1.C, 3ua2.1.D, 3ua2.1.E, 3ua2.1.F, 3uk6.1.A, 3umf.1.A, 3upu.1.A, 3upu.3.A, 3uwk.1.A, 3uwx.1.B, 3v4r.1.A, 3v70.1.A, 3v9p.1.A, 3vaa.1.A, 3vaa.2.A, 3vaa.3.A, 3vfd.1.A, 3vkg.1.A, 3vkg.1.B, 3vkh.1.A, 3vkh.2.A, 3vyx.1.A, 3vyy.1.A, 3vyy.2.A, 3w34.1.A, 3w8r.1.A, 3whk.1.A, 3zd6.1.A, 3zd7.1.A, 3zia.2.C, 3zuh.1.A, 3zw6.1.A, 3zw6.1.B, 3zw6.1.F, 4a15.1.A, 4a1f.1.A, 4a1f.1.B, 4a2p.1.A, 4a2q.1.A, 4a2q.3.A, 4a2w.1.A, 4a2w.2.A, 4a36.2.A, 4a4d.1.A, 4a4z.1.A, 4a7f.1.C, 4a7h.1.C, 4a7l.1.C, 4a92.1.A, 4ae3.1.A, 4ag5.1.B, 4ag5.2.A, 4ag5.2.B, 4ag6.2.B, 4ai6.1.A, 4ai6.1.B, 4akg.1.A, 4akg.1.B, 4akh.1.A, 4akh.2.A, 4aki.1.B, 4anj.1.A, 4asu.1.A, 4asu.1.C, 4asu.1.D, 4asu.1.E, 4asu.1.F, 4ay2.1.A, 4b2i.1.A, 4b2p.1.A, 4b2q.1.2, 4b2q.1.B, 4b2q.1.C, 4b2q.1.D, 4b2q.1.E, 4b3c.1.A, 4b3d.2.A, 4b3f.1.A, 4b3g.1.A, 4b3g.2.A, 4b6e.1.A, 4b75.1.A, 4be7.1.A, 4beb.1.A, 4bec.1.A, 4bgd.1.A, 4bij.1.A, 4bil.1.A, 4blp.1.A, 4blp.1.D, 4blp.1.F, 4bpb.1.A, 4bqs.3.A, 4bru.1.A, 4brw.1.A, 4bs1.1.B, 4buj.1.A, 4buj.2.A, 4byf.1.A, 4bzp.1.A, 4c0b.1.A, 4c0h.1.A, 4c2t.1.A, 4c2t.2.A, 4c2u.1.A, 4c30.1.A, 4c30.2.B, 4c7o.1.A, 4c7o.1.B, 4c7o.2.A, 4c9b.1.A, 4cbg.2.A, 4cbh.2.A, 4cbh.4.A, 4cbi.2.A, 4cbi.4.A, 4cbl.1.A, 4cbl.3.A, 4cbm.1.A, 4cbm.3.A, 4cbm.4.A, 4cdg.1.A, 4ce4.1.3, 4ceh.1.A, 4cei.1.A, 4cej.1.A, 4cej.1.B, 4cgz.1.A, 4ciu.1.A, 4cr2.1.O, 4cr2.1.P, 4cr2.1.Q, 4cr2.1.R, 4cr2.1.S, 4cr2.1.T, 4cr3.1.O, 4cr3.1.P, 4cr3.1.Q, 4cr3.1.R, 4cr3.1.S, 4cr3.1.T, 4cr4.1.O, 4cr4.1.P, 4cr4.1.Q, 4cr4.1.R, 4cr4.1.S, 4cr4.1.T, 4crw.1.B, 4ct4.1.B, 4ct4.2.B, 4ct5.1.A, 4cvn.1.A, 4d25.1.A, 4d2i.1.A, 4d2i.1.B, 4d2q.1.F, 4d2u.1.F, 4d2x.1.E, 4d6p.1.A, 4d80.1.A, 4d80.1.B, 4d80.1.C, 4d80.1.D, 4d80.1.F, 4d81.1.A, 4d82.1.A, 4db1.1.A, 4db1.2.A, 4db2.1.A, 4db4.1.A, 4dbp.1.A, 4dbq.1.A, 4dbr.1.A, 4dc9.2.D, 4ddt.1.A, 4ddu.1.A, 4ddv.1.A, 4ddw.1.A, 4ddx.1.A, 4dkk.1.A, 4dwj.1.A, 4dxa.1.A, 4e2i.1.A, 4e5u.1.A, 4e5u.1.B, 4e7s.1.A, 4e7s.2.A, 4e7z.1.A, 4edh.1.B, 4eiw.1.A, 4eiw.1.B, 4ern.1.A, 4esv.1.B, 4esv.1.C, 4esv.1.D, 4esv.1.E, 4esv.1.F, 4esv.1.G, 4esv.2.B, 4esv.2.C, 4esv.2.D, 4esv.2.E, 4esv.2.F, 4esv.2.G, 4eun.1.A, 4f4i.1.B, 4f91.1.A, 4f92.1.A, 4f93.1.A, 4fct.1.A, 4fcv.3.A, 4fcw.1.A, 4fcw.2.A, 4fcw.3.A, 4fd2.1.A, 4gdf.1.A, 4gdf.1.B, 4gl2.1.A, 4gl2.2.A, 4gmd.2.B, 4hse.1.A, 4hut.1.A, 4hut.1.B, 4hyy.1.A, 4i1s.1.A, 4i34.1.A, 4i34.1.B, 4i34.1.C, 4i34.1.D, 4i4l.1.D, 4i4l.1.E, 4i4l.1.F, 4i5o.1.A, 4i5o.1.C, 4i5o.1.E, 4i63.1.C, 4i63.1.F, 4i81.1.B, 4i81.1.C, 4i81.1.D, 4i81.1.E, 4i81.1.F, 4iee.1.A, 4ife.1.A, 4ihq.1.A, 4ihq.1.B, 4ihq.1.C, 4ii7.1.A, 4ii7.1.B, 4ii7.2.B, 4ijm.1.A, 4ijm.1.C, 4ijm.1.F, 4kbf.1.A, 4kbf.2.A, 4kbg.2.A, 4kfs.1.A, 4kfs.2.A, 4kft.3.A, 4kft.4.A, 4kit.1.A, 4kln.1.A, 4kod.1.A, 4kod.2.E, 4ksr.1.A, 4ksr.1.B, 4ksr.1.C, 4kss.1.A, 4kss.1.B, 4kss.1.C, 4kss.1.D, 4kss.1.E, 4kss.1.F, 4kv9.1.A, 4kva.1.B, 4kxf.10.D, 4kxf.11.D, 4kxf.2.A, 4kxf.3.A, 4kxf.4.A, 4kxf.5.A, 4kxf.9.B, 4kxf.9.C, 4l15.1.A, 4l16.1.A, 4l79.1.A, 4lcb.1.A, 4lgm.1.A, 4lj5.1.A, 4lj6.1.A, 4lj7.1.A, 4lj7.2.A, 4lj7.3.A, 4lj8.1.A, 4ljy.1.A, 4lk2.1.A, 4lk2.2.A, 4lwz.1.A, 4lx0.1.A, 4ly6.1.A, 4ly6.1.B, 4ly6.1.C, 4ly6.1.D, 4ly6.1.E, 4ly6.1.F, 4ly6.2.F, 4lya.1.A, 4lzz.1.A, 4lzz.1.B, 4lzz.1.C, 4lzz.1.D, 4lzz.1.E, 4lzz.1.F, 4lzz.3.F, 4m30.1.A, 4m30.1.B, 4m4w.1.A, 4m4w.1.B, 4m4w.1.C, 4m4w.1.D, 4m4w.1.E, 4m4w.1.F, 4m4w.1.J, 4m4w.1.K, 4m4w.1.L, 4m4w.1.M, 4m4w.1.N, 4m4w.1.O, 4mkf.1.A, 4mkf.2.A, 4mkh.1.A, 4n0n.1.A, 4n0o.1.A, 4n0o.2.A, 4n0o.3.A, 4n0o.4.A, 4n1a.1.A, 4n1a.2.A, 4n1a.4.A, 4ngb.1.B, 4ngf.4.A, 4ngf.4.B, 4ngg.1.B, 4nh0.1.A, 4nh0.2.A, 4nha.2.A, 4nho.1.A, 4nkr.1.A, 4nkr.2.A, 4nkr.2.B, 4nkr.3.A, 4nkr.3.B, 4nl4.1.A, 4nl8.2.B, 4nmn.1.A, 4nmn.1.B, 4np6.1.A, 4np6.1.B, 4ntz.1.A, 4nu0.1.A, 4nu0.2.A, 4o0m.1.A, 4o0m.1.B, 4o0m.1.C, 4o3m.1.A, 4oi0.1.A, 4oi4.1.A, 4ojk.1.A, 4ojq.1.A, 4ojq.1.B, 4ok3.1.A, 4ok3.1.B, 4oks.1.A, 4on9.1.A, 4on9.2.A, 4oog.1.C, 4oqf.1.A, 4oun.1.A, 4oyh.1.A, 4oyh.2.A, 4oyh.2.B, 4oyh.3.A, 4oyh.3.B, 4p7h.1.A, 4p7h.2.A, 4pa0.1.A, 4pa0.2.A, 4pfo.1.A, 4pht.1.B, 4pj3.1.A, 4pjj.1.A, 4pjk.1.A, 4pjl.1.A, 4pk4.1.A, 4po1.1.A, 4px9.1.A, 4pxa.1.A, 4pzl.1.A, 4pzl.1.B, 4q2c.1.A, 4q47.1.A, 4q47.2.A, 4q48.1.A, 4q48.2.A, 4qnm.1.A, 4qqw.6.A, 4qqx.1.A, 4qqx.4.A, 4qrh.1.A, 4qrh.1.B, 4qu4.1.A, 4r2h.1.A, 4r7z.1.A, 4rh7.1.A, 4s20.1.F, 4s20.2.F, 4tl6.1.A, 4tl6.1.E, 4tl8.1.A, 4tl8.1.C, 4tl8.1.D, 4tl9.1.A, 4tl9.1.B, 4tl9.1.D, 4tl9.1.E, 4tla.1.C, 4tla.1.E, 4tla.1.F, 4tlb.1.A, 4tlb.1.B, 4tlb.1.D, 4tlb.1.E, 4tlc.1.A, 4tlc.1.B, 4tlc.1.D, 4tlc.1.E, 4tld.1.A, 4tld.1.C, 4tld.1.D, 4tle.1.A, 4tle.1.B, 4tle.1.D, 4tle.1.F, 4tmu.1.A, 4tsf.1.B, 4tsf.1.E, 4tt3.1.A, 4tt3.1.C, 4twz.1.A, 4tyn.1.A, 4tyw.1.A, 4tyy.1.A, 4tz0.1.A, 4u4c.1.A, 4u7d.1.A, 4uaq.1.A, 4ue5.1.D, 4uj3.1.A, 4uj4.1.A, 4uj5.1.A, 4uj5.2.A, 4unn.1.A, 4unp.1.A, 4unq.1.A, 4unq.2.A, 4uxh.1.A, 4uxj.1.A, 4uxj.1.B, 4uxj.1.C, 4v1a.1.H, 4w5h.1.A, 4w5j.1.A, 4w5j.3.A, 4w5w.1.A, 4w7s.1.A, 4w7s.1.B, 4w8f.1.A, 4w8f.3.B, 4wft.2.A, 4wia.1.A, 4wop.1.A, 4wvy.1.B, 4wvy.1.E, 4ww0.1.A, 4ww0.1.E, 4ww0.1.F, 4ww4.1.B, 4ww4.2.E, 4wxp.1.A, 4wxr.1.A, 4wyq.1.A, 4wyq.2.A, 4xd7.1.A, 4xd7.1.B, 4xd7.1.C, 4xd7.1.D, 4xd7.1.E, 4xd7.1.F, 4xgc.1.C, 4xgc.1.D, 4xgc.1.G, 4xgt.1.A, 4xgu.1.A, 4xgu.1.B, 4xgu.1.C, 4xgu.1.D, 4xgu.1.E, 4xgu.1.F, 4xjx.1.A, 4xjx.1.B, 4xqk.1.A, 4xqk.2.A, 4y0a.1.A, 4yds.1.A, 4ypl.1.A, 4ypl.1.B, 4ypl.1.C, 4ypl.1.D, 4ypl.1.E, 4ypl.1.F, 4ypn.1.A, 4yqf.1.A, 4yqf.2.A, 4ys0.1.A, 4yxw.1.A, 4yxw.1.B, 4yxw.1.D, 4yxw.1.E, 4z1m.1.C, 4z51.1.A, 4z54.1.A, 4z54.1.B, 4z8q.1.A, 4z8u.1.C, 4z8x.1.A, 4zc0.1.A, 4zc0.1.B, 4zcf.1.C, 4zg4.1.A, 4zlk.1.A, 4zni.1.A, 4znj.1.A, 4znk.1.A, 4znl.2.A, 4znl.3.A, 4zpx.1.A, 5a9f.1.D, 5a9j.1.A, 5a9j.3.A, 5anr.2.B, 5aor.1.A, 5aor.2.A, 5ara.1.A, 5ara.1.C, 5ara.1.D, 5ara.1.F, 5are.1.A, 5are.1.B, 5are.1.C, 5are.1.D, 5are.1.E, 5arh.1.A, 5arh.1.C, 5arh.1.D, 5arh.1.E, 5arh.1.F, 5ari.1.A, 5ari.1.C, 5ari.1.D, 5ari.1.E, 5b16.1.A, 5b7i.1.A, 5bn3.1.B, 5bo5.1.A, 5bq5.1.A, 5c18.1.A, 5c18.1.B, 5c18.1.C, 5c18.1.D, 5c18.1.E, 5c18.1.F, 5c19.1.A, 5c19.1.B, 5c19.1.C, 5c19.1.D, 5c19.1.E, 5c19.1.F, 5c1a.1.A, 5c1a.1.B, 5c1a.1.C, 5c1a.1.D, 5c1a.1.E, 5c1a.1.F, 5c1a.2.A, 5c1a.2.B, 5c1a.2.C, 5c1a.2.D, 5c1a.2.E, 5c1a.2.F, 5c1b.1.A, 5c1b.1.B, 5c1b.1.C, 5c1b.1.E, 5c1b.1.F, 5c3c.1.A, 5c3c.1.B, 5ck4.1.A, 5ck5.1.A, 5ck5.2.A, 5ck5.3.A, 5ck5.4.A, 5cyo.1.A, 5cyo.2.A, 5d0u.1.A, 5d4w.1.A, 5dca.1.A, 5dtu.1.A, 5dv7.1.C, 5dyg.1.E, 5dyi.1.A, 5dzr.1.A, 5e02.1.A, 5e3h.1.A, 5e4f.1.A, 5e4f.2.A, 5e7i.1.A, 5e7i.2.A, 5e7i.3.A, 5e7j.1.A, 5e7m.1.A, 5e7p.1.A, 5ean.1.A, 5eaw.1.A, 5eax.1.A, 5elx.1.A, 5ep0.1.A, 5ep1.1.A, 5ep2.1.A, 5ep4.1.A, 5eqt.1.A, 5eul.1.A, 5ext.1.D, 5exx.1.A, 5f3o.1.A, 5f3o.1.B, 5f3q.1.A, 5f4h.1.A, 5f4h.1.B, 5f4h.1.C, 5f4h.1.D, 5f4h.1.E, 5f4h.1.F, 5f98.1.C, 5f9f.1.A, 5f9f.1.E, 5f9h.1.E, 5ffj.1.A, 5ffj.2.A, 5fhd.2.B, 5fhe.1.A, 5fhf.1.A, 5fhg.2.A, 5fhh.1.A, 5fhh.2.A, 5fij.1.A, 5fij.1.B, 5fij.1.C, 5fij.1.E, 5fij.1.F, 5fik.1.A, 5fik.1.B, 5fik.1.D, 5fik.1.E, 5fik.1.F, 5fil.1.A, 5fil.1.C, 5fil.1.F, 5fl3.1.A, 5fl7.1.A, 5fl7.1.B, 5fl7.1.C, 5fl7.1.D, 5fl7.1.E, 5fl7.1.F, 5fm7.1.A, 5fm7.1.B, 5fmf.1.0, 5fmf.1.A, 5fox.1.A, 5ftb.1.A, 5ftc.1.A, 5ftd.1.A, 5fte.1.A, 5ftf.1.A, 5ftj.1.B, 5ftk.1.E, 5ftl.1.B, 5ftm.1.A, 5ftn.1.A, 5fuw.1.A, 5fuw.1.D, 5fuy.1.A, 5fv0.1.A, 5fv0.2.A, 5g4f.1.A, 5g4f.1.B, 5g4f.1.C, 5g4f.1.D, 5g4f.1.E, 5g4f.1.F, 5g4g.1.A, 5gad.1.7, 5gaf.1.7, 5gan.1.I, 5gao.1.I, 5gi4.1.A, 5gi4.1.B, 5gjb.1.A, 5gjc.1.A, 5gjq.1.N, 5gjq.1.P, 5gjq.1.R, 5gjq.1.T, 5gjq.1.V, 5gjq.1.X, 5gju.1.A, 5gm6.1.B, 5gm6.1.W, 5gn1.1.A, 5gn1.4.A, 5gq1.1.A, 5gq1.1.B, 5gq1.1.C, 5gq1.1.D, 5gq1.1.E, 5gq1.1.F, 5gqh.1.A, 5grb.1.A, 5grb.1.B, 5grb.1.C, 5grb.1.D, 5grb.2.A, 5gvr.1.A, 5gvs.1.A, 5gvs.3.A, 5gvu.1.A, 5gvu.2.A, 5gvu.3.A, 5h1b.1.A, 5h1y.1.A, 5h1y.2.A, 5h53.1.A, 5h8c.1.A, 5h8w.1.A, 5hci.1.A, 5hci.2.A, 5hcn.1.A, 5hd9.1.A, 5he8.1.A, 5he8.1.B, 5he8.2.A, 5he8.2.B, 5he8.3.A, 5he8.3.B, 5he8.4.A, 5he8.4.B, 5he8.5.A, 5he8.5.B, 5he8.6.A, 5he8.6.B, 5he9.1.A, 5hkk.2.A, 5hkk.2.B, 5hkk.2.C, 5hkk.2.D, 5hkk.2.E, 5hmp.1.A, 5hmp.2.A, 5hzr.1.A, 5i0h.1.A, 5i0h.2.A, 5i0i.1.A, 5i8q.1.A, 5ifs.1.D, 5ifw.1.B, 5ik2.1.A, 5ik2.1.B, 5ik2.1.C, 5ikn.1.D, 5ikn.1.E, 5ikn.1.F, 5ikn.1.G, 5ikn.1.H, 5ikn.1.I, 5ikn.1.J, 5irr.1.A, 5it5.1.A, 5it5.1.B, 5it5.1.C, 5it5.1.D, 5it5.1.E, 5it5.1.F, 5ivl.1.A, 5ivl.1.B, 5ivw.1.A, 5ivw.1.B, 5j1s.1.A, 5j1t.1.A, 5jaj.1.A, 5jb2.1.A, 5jbj.1.A, 5jc3.1.A, 5jc7.1.A, 5jcs.1.7, 5jed.1.A, 5jee.1.A, 5ji2.3.A, 5ji2.3.B, 5ji3.1.R, 5jji.1.A, 5jji.1.D, 5jji.1.E, 5jji.1.F, 5jjl.1.C, 5jmt.1.A, 5jpq.1.8, 5jps.1.A, 5jpt.1.A, 5jpt.2.A, 5jrj.1.A, 5jrz.1.A, 5jul.1.A, 5jwh.1.A, 5jwo.1.A, 5jwq.1.A, 5jwq.1.C, 5jwr.1.A, 5jwr.2.A, 5jxr.1.A, 5jxt.1.A, 5jxt.1.B, 5jxt.1.E, 5jxt.1.F, 5jxt.1.L, 5jxt.1.O, 5jxt.1.R, 5jxt.1.S, 5jzc.1.A, 5jzv.1.A, 5k8l.1.A, 5k8t.1.A, 5k9t.1.A, 5kdd.1.A, 5kg8.1.A, 5kiw.1.A, 5kiw.1.B, 5kiy.1.A, 5kne.1.A, 5kne.1.B, 5kne.1.C, 5kne.1.D, 5kne.1.E, 5kne.1.F, 5kwa.1.A, 5kzf.1.A, 5kzf.1.B, 5kzf.1.C, 5kzf.1.D, 5kzf.1.E, 5kzf.1.F, 5kzf.2.A, 5kzf.2.B, 5kzf.2.C, 5kzf.2.D, 5kzf.2.E, 5kzf.2.F, 5l3q.1.A, 5l3r.1.A, 5l3r.1.B, 5l3v.1.A, 5l3v.2.A, 5l4g.1.2, 5l4g.1.3, 5l4g.1.4, 5l4g.1.5, 5l4g.1.6, 5l4g.1.7, 5l8v.1.A, 5lb2.1.A, 5lb3.1.A, 5lb3.2.A, 5lb4.1.A, 5lb5.1.A, 5lb5.2.A, 5lb5.3.A, 5lb8.1.A, 5lba.2.A, 5lba.4.A, 5lbi.1.A, 5ld2.1.A, 5ld2.1.C, 5lj5.1.G, 5lj5.1.c, 5ln3.1.P, 5ln3.1.Q, 5ln3.1.R, 5ln3.1.S, 5ln3.1.T, 5ln3.1.U, 5lqx.1.E, 5lqx.1.F, 5lqx.1.G, 5lqx.1.H, 5lqx.1.I, 5lqy.1.G, 5lqz.1.F, 5lst.1.A, 5lta.1.A, 5ltj.1.A, 5ltk.1.A, 5m05.1.A, 5m32.1.2, 5m32.1.3, 5m32.1.4, 5m32.1.5, 5m32.1.6, 5m32.1.7, 5m52.1.A, 5m52.2.A, 5m59.1.B, 5m59.2.B, 5m59.3.B, 5m59.4.B, 5m5p.1.A, 5m5p.2.A, 5mbv.1.A, 5mbv.1.C, 5mc6.34.A, 5mfx.1.A, 5mla.1.A, 5mp9.1.2, 5mp9.1.3, 5mp9.1.4, 5mp9.1.5, 5mp9.1.6, 5mp9.1.7, 5mpa.1.2, 5mpa.1.3, 5mpa.1.4, 5mpa.1.5, 5mpa.1.6, 5mpa.1.7, 5mpb.1.2, 5mpb.1.3, 5mpb.1.4, 5mpb.1.5, 5mpb.1.6, 5mpb.1.7, 5mpc.1.2, 5mpc.1.3, 5mpc.1.4, 5mpc.1.5, 5mpc.1.6, 5mpc.1.7, 5mq0.1.4, 5mre.35.A, 5mrf.31.A, 5mrf.62.A, 5mvr.1.A, 5mz4.1.A, 5mz4.1.B, 5n69.1.A, 5n69.1.B, 5n6a.1.A, 5n8l.1.A, 5n8m.1.A, 5n8o.1.A, 5n8s.1.A, 5n8s.2.A, 5n8u.2.A, 5n8y.1.B, 5n90.2.A, 5n94.1.A, 5n9e.2.A, 5n9f.1.A, 5n9f.2.A, 5nco.1.9, 5nco.1.c, 5np7.1.D, 5np9.1.A, 5npa.1.A, 5npg.1.A, 5nq5.1.A, 5nrn.1.A, 5nrn.1.B, 5nss.1.F, 5nss.1.H, 5nss.1.I, 5nss.1.J, 5nss.1.K, 5nt7.1.A, 5nug.1.A, 5nwl.1.A, 5nwl.1.G, 5o2l.1.A, 5o2s.2.A, 5o31.1.N, 5o6b.1.A, 5o6b.1.B, 5o6d.1.A, 5o6d.2.A, 5o6e.1.A, 5o6e.2.A, 5o9g.1.K, 5o9z.1.C, 5oaf.1.A, 5oaf.1.B, 5oaf.1.C, 5oaf.1.D, 5oaf.1.E, 5oaf.1.F, 5oc4.1.A, 5oc5.1.A, 5oc6.1.A, 5oe8.1.A, 5oe8.2.A, 5oe8.3.A, 5oea.1.A, 5oea.4.A, 5oea.6.A, 5of4.1.A, 5of4.1.B, 5ofo.1.A, 5ofo.1.B, 5ofo.1.C, 5ofo.1.D, 5ofo.1.E, 5ofo.1.F, 5og1.1.A, 5og1.1.B, 5og1.1.C, 5og1.1.D, 5og1.1.E, 5og1.1.F, 5oiu.1.A, 5oiu.1.C, 5oiu.1.E, 5ooq.1.A, 5oqj.1.3, 5oqj.1.W, 5oql.1.9, 5qub.1.A, 5qul.1.A, 5qum.1.A, 5qum.2.A, 5qun.1.A, 5rob.1.A, 5rob.2.A, 5sup.1.A, 5sup.2.A, 5sup.3.A, 5suq.1.A, 5suq.1.C, 5sva.1.Y, 5sva.1.Z, 5swj.1.A, 5swl.1.A, 5syp.1.A, 5syp.2.A, 5syr.1.A, 5t0g.1.O, 5t0g.1.P, 5t0g.1.Q, 5t0g.1.R, 5t0g.1.S, 5t0g.1.T, 5t0h.1.A, 5t0h.1.B, 5t0h.1.C, 5t0h.1.D, 5t0h.1.E, 5t0h.1.F, 5t0i.1.A, 5t0i.1.B, 5t0i.1.C, 5t0i.1.D, 5t0i.1.E, 5t0i.1.F, 5t0j.1.P, 5t0j.1.Q, 5t0j.1.R, 5t0j.1.S, 5t0j.1.T, 5t0j.1.U, 5t16.1.A, 5t16.1.B, 5t45.1.A, 5t4o.1.A, 5t4o.1.B, 5t4o.1.C, 5t4p.1.A, 5t4p.1.B, 5t4p.1.C, 5t4q.1.A, 5t4q.1.B, 5t4q.1.C, 5tnu.1.A, 5tnu.2.A, 5tsg.1.A, 5tsg.1.C, 5tsh.1.A, 5tsh.1.B, 5tsh.1.E, 5txg.1.A, 5txv.3.D, 5u32.1.A, 5ubv.1.A, 5ubv.2.A, 5uie.1.A, 5uie.1.B, 5uie.1.D, 5uie.1.E, 5uie.1.F, 5uiv.1.A, 5uj7.1.A, 5uj7.1.B, 5uj7.1.C, 5uj7.2.A, 5uj7.2.B, 5uj7.2.C, 5ujm.1.A, 5ujm.1.E, 5urj.1.A, 5urm.1.A, 5urm.2.A, 5v7x.1.A, 5v8f.1.H, 5v8f.1.I, 5v8f.1.L, 5v8f.1.M, 5v9x.1.A, 5vc7.1.C, 5vca.1.A, 5vca.1.B, 5vca.1.C, 5vca.1.D, 5vca.1.E, 5vca.1.F, 5vfp.1.d, 5vfp.1.e, 5vfp.1.f, 5vfp.1.g, 5vfp.1.h, 5vfp.1.i, 5vfq.1.d, 5vfq.1.e, 5vfq.1.f, 5vfq.1.g, 5vfq.1.h, 5vfq.1.i, 5vfr.1.d, 5vfr.1.e, 5vfr.1.f, 5vfr.1.g, 5vfr.1.h, 5vfr.1.i, 5vfs.1.d, 5vfs.1.e, 5vfs.1.f, 5vfs.1.g, 5vfs.1.h, 5vfs.1.i, 5vft.1.L, 5vft.1.M, 5vft.1.N, 5vft.1.O, 5vft.1.P, 5vft.1.Q, 5vfu.1.L, 5vfu.1.M, 5vfu.1.N, 5vfu.1.O, 5vfu.1.P, 5vfu.1.Q, 5vh9.1.A, 5vha.1.A, 5vhc.1.A, 5vhe.1.A, 5vhf.1.B, 5vhf.1.C, 5vhf.1.D, 5vhf.1.E, 5vhf.1.F, 5vhf.1.G, 5vhi.1.A, 5vhi.1.B, 5vhi.1.C, 5vhi.1.D, 5vhi.1.E, 5vhi.1.F, 5vhj.1.B, 5vhj.1.C, 5vhj.1.D, 5vhj.1.E, 5vhj.1.F, 5vhj.1.G, 5vhm.1.B, 5vhm.1.C, 5vhm.1.D, 5vhm.1.E, 5vhm.1.F, 5vhm.1.G, 5vhn.1.B, 5vhn.1.C, 5vhn.1.D, 5vhn.1.E, 5vhn.1.F, 5vhn.1.G, 5vho.1.B, 5vho.1.C, 5vho.1.D, 5vho.1.E, 5vho.1.F, 5vho.1.G, 5vhp.1.B, 5vhp.1.C, 5vhp.1.D, 5vhp.1.E, 5vhp.1.F, 5vhp.1.G, 5vhq.1.B, 5vhq.1.C, 5vhq.1.D, 5vhq.1.E, 5vhq.1.F, 5vhq.1.G, 5vhr.1.B, 5vhr.1.C, 5vhr.1.D, 5vhr.1.E, 5vhr.1.F, 5vhr.1.G, 5vhs.1.A, 5vhs.1.B, 5vhs.1.C, 5vhs.1.D, 5vhs.1.E, 5vhs.1.F, 5vi7.1.A, 5vjh.1.A, 5vjh.1.B, 5vjh.1.C, 5vjh.1.D, 5vjh.1.E, 5vjh.1.F, 5vlj.1.A, 5vp7.1.A, 5vpi.2.A, 5vqa.1.A, 5vvr.1.M, 5vy8.1.A, 5vy8.1.B, 5vy8.1.C, 5vy8.1.D, 5vy8.1.E, 5vy8.1.F, 5vy9.1.A, 5vy9.1.B, 5vy9.1.C, 5vy9.1.D, 5vy9.1.E, 5vy9.1.F, 5vya.1.A, 5vya.1.B, 5vya.1.C, 5vya.1.D, 5vya.1.E, 5vya.1.F, 5w0t.1.A, 5w1a.1.A, 5w1a.2.A, 5wbw.1.A, 5wbw.2.A, 5wbw.3.A, 5wc0.1.A, 5wc0.1.D, 5wc0.1.E, 5wc0.1.F, 5wc1.1.A, 5wc2.1.A, 5wcb.1.A, 5wcb.1.B, 5wcb.1.C, 5wcb.1.D, 5wcb.1.E, 5wcb.1.F, 5wdx.1.A, 5wsg.1.h, 5wvi.1.A, 5wvi.1.B, 5wvi.1.P, 5wvi.1.Q, 5wvi.1.R, 5wvi.1.W, 5wvk.1.2, 5wvk.1.3, 5wvk.1.4, 5wvk.1.5, 5wvk.1.6, 5wvk.1.7, 5wwp.1.A, 5wwp.2.A, 5wx1.1.A, 5x06.1.E, 5x06.1.G, 5x0x.1.K, 5x0y.1.K, 5x6i.1.A, 5x6j.1.A, 5x7j.1.A, 5x7j.1.B, 5x86.1.A, 5x86.1.B, 5x8k.1.A, 5x8k.1.B, 5xc6.1.A, 5xc7.1.A, 5xdr.1.A, 5xjc.1.Y, 5xmi.1.A, 5xmi.1.B, 5xmi.1.C, 5xmi.1.D, 5xmi.1.E, 5xmi.1.F, 5xmk.1.A, 5xmk.1.B, 5xmk.1.C, 5xmk.1.D, 5xmk.1.E, 5xmk.1.F, 5y4z.1.A, 5y6m.1.A, 5y6n.1.A, 5y88.1.W, 5ybh.1.A, 5ybh.2.A, 5ybi.2.A, 5ylz.1.W, 5yvj.1.A, 5yvu.1.A, 5yvv.1.A, 5yvw.1.A, 5yvy.1.B, 5yw1.1.A, 5yww.1.A, 5yz8.1.A, 5yzg.1.0, 5z3g.1.Y, 5z3l.1.K, 5z3o.1.K, 5z3q.1.A, 5z3q.1.B, 5z3q.1.C, 5z3q.1.D, 5z3q.2.A, 5z3u.1.A, 5z3v.1.A, 5z6q.1.A, 5z6r.1.A, 5zak.1.A, 5zak.1.B, 5zal.1.A, 5zal.1.B, 5zam.1.A, 5zam.1.B, 5zbz.1.A, 5zc9.1.A, 5zfq.1.A, 5zfr.1.A, 5zfr.1.B, 5zfr.1.C, 5zql.1.A, 5zql.2.A, 5zqm.1.A, 5zr1.1.A, 5zr1.1.D, 5zr1.1.E, 5zt1.1.A, 5zt1.2.A, 5ztm.1.A, 5ztm.1.B, 5zui.1.D, 5zwo.1.0, 6ac6.2.A, 6ac6.3.A, 6ac8.1.B, 6ac8.2.A, 6aca.1.A, 6acx.1.B, 6adw.1.A, 6ah0.1.B, 6ah0.1.s, 6ahf.1.A, 6ahf.1.B, 6ahf.1.C, 6ahf.1.D, 6ahf.1.E, 6ahf.1.F, 6aib.1.A, 6aic.1.A, 6amn.1.A, 6ap1.1.A, 6ap1.1.B, 6ap1.1.E, 6ap1.1.G, 6az0.1.A, 6az0.1.B, 6az0.1.C, 6az0.1.D, 6az0.1.E, 6az0.1.F, 6azy.1.A, 6b4i.1.C, 6b4j.2.C, 6b4k.1.A, 6b4k.2.A, 6b5b.1.C, 6b5c.1.A, 6b5d.1.A, 6bb8.1.A, 6bbm.1.A, 6bbm.1.B, 6bbm.1.C, 6bbm.1.D, 6bbm.1.E, 6bbm.1.F, 6bge.1.A, 6bih.1.A, 6bk8.1.S, 6blb.1.A, 6bmf.1.A, 6bmf.1.D, 6bmf.1.E, 6bnp.1.A, 6bnp.1.B, 6bnp.1.C, 6bnp.1.D, 6bnp.1.E, 6bnp.1.F, 6bnq.1.A, 6bnq.1.B, 6bnq.1.C, 6bnq.1.D, 6bnq.1.E, 6bnq.1.F, 6bnv.1.A, 6bnw.1.A, 6bog.1.A, 6bog.2.A, 6bu9.1.A, 6bua.1.A, 6c1d.1.F, 6c1g.1.A, 6c1h.1.F, 6c2u.1.A, 6c2v.1.A, 6c66.1.A, 6c90.1.A, 6cb1.1.9, 6chs.1.B, 6cp3.1.L, 6cp3.1.M, 6cp3.1.O, 6cp3.1.P, 6crm.1.A, 6cy1.1.A, 6cy1.1.B, 6cy5.1.A, 6cz5.1.A, 6d00.1.A, 6d00.1.B, 6d00.1.C, 6d00.1.D, 6d00.1.E, 6d00.1.F, 6d6r.1.M, 6dcr.1.A, 6dcr.2.A, 6dgd.1.A, 6dgd.2.A, 6dju.1.C, 6dju.1.D, 6dju.1.F, 6dju.1.G, 6djv.1.A, 6djv.1.B, 6djv.1.F, 6djv.1.G, 6due.1.A, 6duq.2.C, 6e10.1.A, 6e10.1.B, 6e10.1.C, 6e10.1.D, 6e10.1.E, 6e10.1.F, 6e11.1.D, 6e11.1.E, 6e11.1.F, 6e11.1.G, 6e11.1.H, 6e11.1.J, 6ed3.1.C, 6ed3.1.E, 6ed3.1.F, 6ef0.1.H, 6ef0.1.I, 6ef0.1.J, 6ef0.1.K, 6ef0.1.L, 6ef0.1.M, 6ef1.1.H, 6ef1.1.I, 6ef1.1.J, 6ef1.1.K, 6ef1.1.L, 6ef1.1.M, 6ef2.1.H, 6ef2.1.I, 6ef2.1.J, 6ef2.1.K, 6ef2.1.L, 6ef2.1.M, 6ef3.1.O, 6ef3.1.P, 6ef3.1.Q, 6ef3.1.R, 6ef3.1.S, 6ef3.1.T, 6eg2.1.A, 6eg3.1.A, 6ei8.1.A, 6ej5.1.A, 6ejf.1.M, 6ejf.1.N, 6ejf.1.O, 6ejf.1.P, 6ejf.1.Q, 6ejf.1.R, 6em5.1.6, 6em8.1.A, 6em8.1.B, 6em8.1.C, 6em8.1.D, 6em8.1.E, 6em8.1.F, 6em8.1.G, 6em8.1.H, 6em8.1.I, 6em8.1.J, 6em9.1.A, 6em9.1.B, 6em9.1.C, 6em9.1.D, 6em9.1.E, 6em9.1.F, 6em9.1.G, 6em9.1.H, 6em9.1.I, 6em9.1.J, 6emw.1.B, 6emw.1.E, 6epc.1.0, 6epc.1.1, 6epc.1.2, 6epc.1.3, 6epc.1.4, 6epc.1.5, 6epd.1.0, 6epd.1.1, 6epd.1.2, 6epd.1.3, 6epd.1.4, 6epd.1.5, 6epe.1.0, 6epe.1.1, 6epe.1.2, 6epe.1.3, 6epe.1.4, 6epe.1.5, 6epf.1.0, 6epf.1.1, 6epf.1.2, 6epf.1.3, 6epf.1.4, 6epf.1.5, 6eud.1.A, 6exn.1.T, 6f00.1.A, 6f0x.1.A, 6f0x.1.B, 6f0x.1.C, 6f0x.1.D, 6f0x.1.E, 6f0x.1.F, 6f4a.1.B, 6f8l.1.M, 6f8l.1.N, 6f8l.1.O, 6f8l.1.P, 6f8l.1.Q, 6f8l.1.R, 6f9s.1.A, 6fa5.1.A, 6fa9.1.A, 6faa.1.A, 6fac.1.A, 6ff7.1.c, 6ff7.1.v, 6fkf.1.A, 6fkf.1.C, 6fkf.1.E, 6fkh.1.B, 6fkh.1.D, 6fkh.1.F, 6fki.1.U, 6fki.1.W, 6fki.1.Y, 6fml.1.A, 6fml.1.B, 6fml.1.C, 6fml.1.D, 6fml.1.E, 6fml.1.F, 6fml.1.G, 6fo1.1.C, 6fo1.1.D, 6fo1.1.E, 6foc.1.A, 6foc.1.B, 6foc.1.C, 6foc.1.E, 6foc.1.F, 6fqd.1.A, 6frk.1.t, 6fsz.1.N, 6ft6.1.t, 6ftx.1.M, 6fvt.1.f, 6fvt.1.g, 6fvt.1.h, 6fvt.1.i, 6fvt.1.j, 6fvt.1.k, 6fvu.1.f, 6fvu.1.g, 6fvu.1.h, 6fvu.1.i, 6fvu.1.j, 6fvu.1.k, 6fvv.1.f, 6fvv.1.g, 6fvv.1.h, 6fvv.1.i, 6fvv.1.j, 6fvv.1.k, 6fvw.1.f, 6fvw.1.g, 6fvw.1.h, 6fvw.1.i, 6fvw.1.j, 6fvw.1.k, 6fvx.1.f, 6fvx.1.g, 6fvx.1.h, 6fvx.1.i, 6fvx.1.j, 6fvx.1.k, 6fvy.1.f, 6fvy.1.g, 6fvy.1.h, 6fvy.1.i, 6fvy.1.j, 6fvy.1.k, 6fwr.1.A, 6fws.1.A, 6fws.1.C, 6g0l.1.L, 6g19.1.A, 6g1s.1.A, 6g1x.1.A, 6g2v.1.A, 6g2w.1.A, 6g2x.1.A, 6g2y.1.A, 6g2z.1.A, 6g30.1.A, 6g7e.1.A, 6gb2.1.j, 6gcn.2.D, 6gcn.2.E, 6gco.1.A, 6gco.1.F, 6geb.1.A, 6geb.1.B, 6geb.1.C, 6geb.1.D, 6geb.1.E, 6geb.1.F, 6gef.1.A, 6gef.1.B, 6gef.1.C, 6gef.1.D, 6gef.1.E, 6gef.1.F, 6gej.1.P, 6gej.1.S, 6gej.1.T, 6gen.1.L, 6gen.1.P, 6gen.1.S, 6gjz.1.A, 6gkh.1.A, 6goe.1.A, 6gox.1.A, 6gpg.1.C, 6gqt.1.A, 6gqt.2.A, 6gqw.5.A, 6gqy.2.A, 6gye.1.A, 6gyf.2.A, 6gzo.1.A, 6h46.1.A, 6h47.1.A, 6h57.1.A, 6h61.1.A, 6h66.1.A, 6h7g.1.A, 6h7g.1.B, 6h7x.1.A, 6hd0.1.G, 6hd3.1.A, 6he4.1.A, 6he4.1.B, 6he4.1.C, 6he4.1.D, 6he4.1.E, 6he4.1.F, 6he8.1.2, 6he8.1.3, 6he8.1.4, 6he8.1.5, 6he8.1.6, 6he8.1.7, 6he9.1.2, 6he9.1.3, 6he9.1.4, 6he9.1.5, 6he9.1.6, 6he9.1.7, 6hea.1.2, 6hea.1.3, 6hea.1.4, 6hea.1.5, 6hea.1.6, 6hea.1.7, 6hec.1.2, 6hec.1.3, 6hec.1.4, 6hec.1.5, 6hec.1.6, 6hec.1.7, 6hed.1.2, 6hed.1.3, 6hed.1.4, 6hed.1.5, 6hed.1.6, 6hed.1.7, 6heg.1.A, 6hf7.1.A, 6hiz.1.W, 6hph.1.A, 6hpt.1.A, 6hpu.2.A, 6hqu.1.A, 6hqu.2.A, 6hqu.3.A, 6hqu.8.A, 6hts.1.A, 6hts.1.B, 6hts.1.C, 6hts.1.D, 6hts.1.E, 6hts.1.F, 6hts.1.G, 6htu.1.C, 6htu.1.D, 6htu.1.E, 6hyp.1.A, 6hys.2.A, 6hyt.3.A, 6hyu.1.A, 6hyu.2.A, 6hz4.1.A, 6hz4.1.B, 6hz4.1.C, 6hz4.1.D, 6hz4.1.E, 6hz4.1.F, 6i26.1.A, 6i27.1.A, 6i3o.1.A, 6i3o.2.A, 6i3p.1.A, 6i3r.1.A, 6i59.1.A, 6i7d.2.A, 6i7d.3.A, 6i7d.4.A, 6i7e.1.A, 6icz.1.b, 6id1.1.5, 6id1.1.g, 6ieg.1.A, 6ieg.2.A, 6ieh.1.A, 6igm.1.A, 6igm.1.B, 6igm.1.C, 6igm.1.D, 6igm.1.E, 6igm.1.F, 6igm.1.H, 6ip2.1.A, 6ip2.1.B, 6ip2.1.C, 6ip2.1.D, 6ip2.1.E, 6ip2.1.F, 6iro.1.A, 6itc.1.A, 6iy1.1.A, 6iy1.4.A, 6iy1.5.A, 6iy1.6.A, 6iy2.1.K, 6iy3.1.K, 6iy8.1.A, 6iy8.1.B, 6iy8.1.C, 6iy8.1.D, 6j17.1.A, 6j19.1.A, 6j2c.1.2, 6j2c.1.3, 6j2c.1.4, 6j2c.1.5, 6j2c.1.6, 6j2c.1.7, 6j2n.1.2, 6j2n.1.3, 6j2n.1.4, 6j2n.1.5, 6j2n.1.6, 6j2n.1.7, 6j2q.1.3, 6j2q.1.4, 6j2q.1.5, 6j2q.1.6, 6j2q.1.7, 6j2x.1.2, 6j2x.1.3, 6j2x.1.4, 6j2x.1.5, 6j2x.1.6, 6j2x.1.7, 6j30.1.3, 6j30.1.4, 6j30.1.5, 6j30.1.6, 6j30.1.7, 6j5i.1.A, 6j5i.1.B, 6j5i.1.C, 6j5i.1.D, 6j5i.1.E, 6j5i.1.F, 6j5j.1.A, 6j5j.1.C, 6j5j.1.D, 6j5j.1.E, 6j5j.1.F, 6j5k.65.A, 6j7e.1.A, 6jd4.1.A, 6jd5.1.A, 6jde.1.A, 6jde.2.A, 6jdi.1.A, 6jdl.1.A, 6jdr.1.A, 6jds.1.A, 6jdu.1.A, 6jim.1.A, 6jpq.1.A, 6jpu.1.A, 6jq0.1.A, 6jq0.1.B, 6jq0.1.C, 6jq0.1.D, 6jq0.1.E, 6jq0.1.F, 6jtz.1.A, 6jyl.1.K, 6jyt.1.A, 6jyt.2.A, 6k0r.1.A, 6k0r.1.D, 6k0r.1.F, 6k1p.1.K, 6kez.1.E, 6kez.1.F, 6kez.2.E, 6kez.2.F, 6kw3.1.1, 6kw4.1.1, 6kw5.1.1, 6kww.1.A, 6kww.11.A, 6kww.13.A, 6kww.16.A, 6kww.2.A, 6kww.24.A, 6kww.5.A, 6kww.6.A, 6kww.7.A, 6kww.8.A, 6kww.9.A, 6kyv.1.B, 6kza.1.A, 6kza.2.A, 6l1q.1.A, 6l3g.1.E, 6l5l.1.A, 6l5m.2.A, 6l5m.3.A, 6l5m.4.A, 6l5n.1.A, 6l5n.2.A, 6l5o.1.A, 6l8d.1.A, 6l8d.1.D, 6l8d.1.E, 6l8o.1.A, 6lk0.1.A, 6ln3.1.A, 6lqp.59.A, 6lqq.58.A, 6lqs.75.A, 6lsy.1.A, 6lt4.1.A, 6ltj.1.I, 6lua.1.A, 6luf.1.A, 6lxd.1.A, 6lxe.1.A, 6m40.1.A, 6m5r.1.A, 6m5s.1.A, 6m5u.1.A, 6m5v.1.A, 6m6a.1.F, 6m6b.1.F, 6mat.1.A, 6mat.1.B, 6mat.1.D, 6mat.1.E, 6mat.1.F, 6mck.1.A, 6mdm.1.A, 6mdm.1.B, 6mdm.1.C, 6mdm.1.D, 6mdm.1.E, 6mdm.1.F, 6mdn.1.A, 6mdn.1.B, 6mdn.1.C, 6mdn.1.D, 6mdn.1.E, 6mdn.1.F, 6mdp.1.A, 6mdp.1.B, 6mdp.1.C, 6mdp.1.D, 6mdp.1.E, 6mdp.1.F, 6mfu.1.A, 6mh3.1.A, 6mii.1.A, 6mii.1.B, 6mii.1.D, 6mii.1.E, 6mii.1.F, 6mq9.1.A, 6mq9.2.A, 6mq9.3.A, 6mqb.1.A, 6mqk.4.A, 6mql.1.A, 6msb.1.M, 6msb.1.N, 6msb.1.P, 6msd.1.O, 6msd.1.P, 6msd.1.Q, 6msd.1.R, 6mse.1.M, 6mse.1.N, 6mse.1.O, 6mse.1.P, 6mse.1.Q, 6mse.1.R, 6msg.1.M, 6msg.1.O, 6msg.1.P, 6msg.1.Q, 6msg.1.R, 6msh.1.M, 6msh.1.N, 6msh.1.O, 6msh.1.P, 6msh.1.Q, 6msh.1.R, 6msj.1.M, 6msj.1.N, 6msj.1.O, 6msj.1.P, 6msj.1.Q, 6msj.1.R, 6msk.1.M, 6msk.1.N, 6msk.1.O, 6msk.1.P, 6msk.1.Q, 6msk.1.R, 6n2i.1.A, 6n2y.1.D, 6n2y.1.E, 6n2y.1.F, 6n2z.1.O, 6n2z.1.P, 6n30.1.N, 6n30.1.O, 6n30.1.P, 6n53.1.A, 6n53.1.B, 6n55.1.A, 6n55.1.B, 6n55.2.A, 6n6l.1.A, 6n6l.2.A, 6n6m.1.A, 6n6m.2.A, 6n6z.1.A, 6n6z.2.A, 6n70.1.A, 6n70.2.A, 6n71.1.A, 6n71.2.A, 6n72.1.A, 6n72.2.A, 6n73.1.A, 6n73.2.A, 6n74.1.A, 6n74.2.A, 6n75.1.A, 6n75.2.A, 6n76.1.A, 6n76.2.A, 6n7n.1.A, 6n7n.1.F, 6n7s.1.A, 6n7s.1.B, 6n7s.1.C, 6n7s.1.D, 6n7s.1.E, 6n7s.1.F, 6n7t.1.A, 6n7t.1.F, 6n7v.1.C, 6n7v.1.D, 6n7v.1.F, 6n8t.1.A, 6n8t.1.B, 6n8t.1.C, 6n8t.1.D, 6n8t.1.E, 6n8t.1.F, 6n8v.1.A, 6n8v.1.B, 6n8v.1.C, 6n8v.1.D, 6n8v.1.E, 6n8v.1.F, 6n8z.1.A, 6n8z.1.B, 6n8z.1.C, 6n8z.1.D, 6n8z.1.E, 6n8z.1.F, 6n9w.1.B, 6n9x.1.B, 6nak.1.C, 6nak.1.F, 6ne3.1.K, 6nmi.1.A, 6nmi.1.B, 6nyv.1.A, 6nyw.1.A, 6nyy.1.A, 6nyy.1.B, 6nyy.1.C, 6nyy.1.D, 6nyy.1.E, 6nyy.1.F, 6o16.1.A, 6o16.2.A, 6o1w.1.A, 6o1w.2.A, 6o1x.1.A, 6o1x.2.A, 6o1y.1.A, 6o1y.2.A, 6o1z.1.A, 6o5f.1.A, 6o8e.1.A, 6o8e.2.A, 6o8f.1.A, 6o8g.1.A, 6o8g.2.A, 6o8g.3.A, 6o8h.1.A, 6o9l.1.2, 6o9l.1.V, 6o9m.1.A, 6o9m.1.H, 6oa9.1.A, 6oa9.1.B, 6oa9.1.C, 6oa9.1.D, 6oa9.1.E, 6oa9.1.F, 6oaa.1.A, 6oaa.1.B, 6oaa.1.C, 6oaa.1.D, 6oab.1.A, 6oab.1.B, 6oab.1.C, 6oab.1.E, 6oax.1.A, 6oax.1.C, 6oax.1.D, 6oax.1.E, 6oay.1.A, 6oay.1.C, 6oay.1.D, 6oay.1.E, 6og1.1.A, 6og1.1.B, 6og2.1.A, 6og2.1.B, 6oif.1.A, 6oje.1.A, 6oje.1.B, 6ojx.1.F, 6ojy.1.A, 6ojy.1.E, 6ojy.1.F, 6ojz.1.D, 6ok2.1.A, 6ok2.1.B, 6ok2.1.C, 6ok2.1.E, 6ok2.1.F, 6okv.1.B, 6olj.1.A, 6olj.1.B, 6olj.1.C, 6olj.1.E, 6olk.1.D, 6oll.1.B, 6oll.1.D, 6olm.1.D, 6omb.1.A, 6omb.1.B, 6omb.1.C, 6omb.1.D, 6omb.1.E, 6on2.1.A, 6on2.1.B, 6on2.1.C, 6on2.1.D, 6on2.1.E, 6on2.1.F, 6oo2.1.A, 6oo2.1.B, 6oo2.1.D, 6oo2.1.E, 6oo2.1.G, 6opc.1.F, 6oqs.1.C, 6oqt.1.B, 6oqu.1.C, 6oqv.1.B, 6oqv.1.C, 6oqv.1.D, 6oqw.1.B, 6oqw.1.C, 6oqw.1.D, 6or5.1.A, 6orb.1.A, 6p07.1.A, 6p07.1.B, 6p07.1.C, 6p07.1.E, 6p07.1.F, 6p11.1.A, 6p12.1.A, 6p14.1.A, 6p4f.1.A, 6p4o.1.A, 6p4o.2.A, 6p4o.3.A, 6p4w.1.A, 6p66.1.A, 6p66.2.A, 6p8v.1.A, 6p8v.1.B, 6p8v.1.C, 6p8v.1.D, 6p8v.1.E, 6p8v.1.F, 6pb3.1.A, 6pdw.1.A, 6pdw.1.B, 6pdw.1.C, 6pdw.1.D, 6pdw.1.E, 6pdy.1.A, 6pdy.1.B, 6pdy.1.C, 6pdy.1.D, 6pdy.1.E, 6pdy.1.F, 6pe0.1.A, 6pe0.1.B, 6pe0.1.C, 6pe0.1.D, 6pe0.1.E, 6pe0.1.F, 6pen.1.A, 6pen.1.B, 6pen.1.C, 6pen.1.D, 6pen.1.E, 6pen.1.G, 6pjw.1.A, 6pjw.1.C, 6pk5.1.A, 6pk5.1.B, 6pk5.1.C, 6pk5.2.B, 6po1.1.A, 6po1.1.B, 6po1.1.C, 6po3.1.A, 6po3.1.D, 6po3.1.E, 6pod.1.E, 6pod.1.F, 6pos.1.A, 6pos.1.C, 6pp5.1.D, 6pp5.1.E, 6pp5.1.F, 6pp6.1.B, 6pp6.1.C, 6pp6.1.F, 6pp7.1.A, 6pp7.1.C, 6pp7.1.D, 6pp8.1.B, 6pp8.1.D, 6pp8.1.E, 6pp8.1.F, 6ppj.1.A, 6ppj.1.B, 6ppr.1.A, 6ppr.1.B, 6ppu.1.A, 6pqv.1.D, 6psp.1.A, 6pwf.1.K, 6pwz.1.D, 6pwz.2.A, 6pwz.2.D, 6pxi.1.E, 6pxi.1.F, 6pxk.1.A, 6pxk.1.F, 6pxk.2.C, 6pxl.1.A, 6pxl.1.B, 6pxl.1.D, 6pxl.1.E, 6pxl.1.F, 6pxl.2.A, 6pxl.2.D, 6pxl.2.E, 6q45.1.A, 6q45.1.B, 6q45.1.C, 6q45.1.D, 6q45.1.E, 6q45.1.F, 6q7l.1.U, 6q7l.1.V, 6q7l.1.W, 6q7l.1.X, 6q7l.1.Y, 6q7l.1.Z, 6q7m.1.U, 6q7m.1.W, 6q7m.1.X, 6q7m.1.Y, 6qdj.1.A, 6qdv.1.0, 6qdv.1.D, 6qdv.1.H, 6qel.1.A, 6qel.1.B, 6qel.1.C, 6qel.1.D, 6qel.1.E, 6qel.1.F, 6qel.1.G, 6qel.1.H, 6qel.1.I, 6qel.1.J, 6qel.1.L, 6qem.1.A, 6qem.1.B, 6qem.1.C, 6qem.1.D, 6qem.1.E, 6qem.1.F, 6qem.1.G, 6qem.1.H, 6qem.1.J, 6qem.1.K, 6qem.1.L, 6qi8.1.A, 6qi8.1.B, 6qi8.1.C, 6qi8.1.D, 6qi8.1.E, 6qi8.1.F, 6qi9.1.A, 6qi9.1.B, 6qi9.1.C, 6qi9.1.D, 6qi9.1.E, 6qi9.1.F, 6qic.1.A, 6qic.2.A, 6qic.4.A, 6qid.1.A, 6qie.1.A, 6qp0.1.A, 6qpw.1.A, 6qs4.1.A, 6qs4.1.B, 6qs4.1.C, 6qs4.1.E, 6qs4.1.F, 6qs6.1.A, 6qs6.1.E, 6qs6.1.F, 6qs7.1.A, 6qs7.1.D, 6qs7.1.E, 6qs7.1.F, 6qs8.1.F, 6qv3.1.A, 6qv4.1.A, 6qw6.1.X, 6qws.1.A, 6r3p.1.A, 6rad.1.A, 6rae.1.A, 6raw.1.D, 6raw.1.F, 6rax.1.D, 6rax.1.F, 6ray.1.C, 6ray.1.E, 6raz.1.H, 6raz.1.L, 6rd4.1.0, 6rd4.1.1, 6rd4.1.2, 6rd4.1.3, 6rd4.1.4, 6rd4.1.Z, 6rdc.1.0, 6rdc.1.2, 6rdc.1.3, 6rdc.1.4, 6rdc.1.Z, 6rdg.1.O, 6rdg.1.P, 6rdg.1.Q, 6rdg.1.R, 6rdg.1.S, 6rdg.1.T, 6rdh.1.4, 6rdk.1.3, 6rdk.1.4, 6rdq.1.2, 6rdq.1.3, 6rdq.1.4, 6rdq.1.Z, 6rdt.1.Z, 6rdw.1.3, 6rdw.1.4, 6rdw.1.Z, 6rdz.1.Z, 6re5.1.Z, 6re8.1.1, 6re8.1.2, 6re8.1.4, 6re8.1.Z, 6res.1.0, 6res.1.1, 6res.1.2, 6res.1.3, 6res.1.4, 6res.1.Z, 6rm8.1.A, 6rm9.1.A, 6rma.1.A, 6rmb.1.A, 6rmc.1.A, 6rmc.2.A, 6rn2.1.A, 6rn2.1.B, 6rn2.1.C, 6rn2.1.D, 6rn2.1.E, 6rn2.1.F, 6rn3.1.A, 6rn3.1.B, 6rn3.1.C, 6rn3.1.D, 6rn3.1.E, 6rn3.1.F, 6rn4.1.A, 6rn4.1.E, 6rn4.1.F, 6ro1.1.A, 6rqc.1.A, 6rqc.1.D, 6rqc.1.E, 6rwz.1.A, 6rxu.36.A, 6rxx.37.A, 6rxy.1.8, 6rxz.35.A, 6ryr.1.K, 6ryu.1.K, 6rze.1.A, 6s0k.1.6, 6s2p.1.A, 6s3a.1.A, 6s3e.1.A, 6s3e.2.A, 6s3h.1.A, 6s3h.2.A, 6s3i.1.A, 6s3m.2.A, 6s3n.1.A, 6s3n.2.A, 6s3o.1.A, 6s3p.1.A, 6s84.1.B, 6s84.1.F, 6s8o.1.A, 6s8q.1.A, 6s8r.1.A, 6s8s.2.A, 6s9i.1.A, 6sdw.1.A, 6sdx.1.A, 6sdy.1.A, 6sfw.1.A, 6sfw.1.B, 6sfw.1.C, 6sfw.1.D, 6sfw.1.E, 6sfw.1.F, 6sh3.1.A, 6sh4.1.A, 6sh5.1.A, 6sh6.1.A, 6sh7.1.A, 6sjb.1.A, 6sje.1.A, 6sjg.1.C, 6so5.1.A, 6so5.1.B, 6sxa.1.A, 6sxb.1.A, 6sxh.1.A, 6sz9.1.A, 6sza.1.A, 6sza.1.C, 6sza.1.D, 6szb.1.C, 6szb.1.F, 6t2u.1.A, 6t4h.1.A, 6t66.1.A, 6t66.1.C, 6t66.1.D, 6t66.1.F, 6t8b.1.A, 6t8b.1.B, 6t8b.1.C, 6t8b.1.D, 6t8b.1.E, 6t8b.1.F, 6t8g.1.A, 6t8g.1.B, 6t8g.1.C, 6t8g.1.D, 6t8g.1.E, 6t8g.1.F, 6t8o.1.A, 6t8o.1.B, 6t8o.1.D, 6t8o.1.F, 6tda.1.S, 6tdy.1.D, 6tdy.1.E, 6tdy.1.F, 6tmh.1.B, 6tmh.1.C, 6tmh.1.D, 6tnn.1.B, 6tnn.1.C, 6tt7.1.A, 6tt7.1.B, 6tt7.1.C, 6tt7.1.D, 6tt7.1.E, 6tt7.1.F, 6tu7.1.A, 6tuu.1.A, 6tuu.4.A, 6tw4.1.A, 6tw9.1.A, 6tzx.1.A, 6u03.1.A, 6u0m.1.J, 6u1y.1.A, 6u5z.1.A, 6ugd.1.A, 6ugd.1.B, 6ugd.1.C, 6ugd.1.E, 6ugd.1.F, 6uge.1.A, 6ugf.1.A, 6ugf.1.B, 6ugf.1.D, 6ugf.1.F, 6ui4.1.A, 6uko.1.A, 6uko.1.B, 6uko.1.C, 6uko.1.D, 6uko.1.E, 6uko.1.F, 6uko.1.G, 6ukp.1.F, 6uks.1.E, 6up2.1.A, 6up3.1.A, 6up4.1.A, 6upa.1.A, 6upa.1.B, 6upq.1.A, 6upq.1.B, 6upr.1.A, 6upr.1.B, 6uqe.1.A, 6uqe.1.B, 6uqe.1.C, 6uqe.1.D, 6uqe.1.E, 6uqe.1.F, 6uqo.1.A, 6uqo.1.B, 6uqo.1.C, 6uqo.1.D, 6uqo.1.E, 6uqo.1.F, 6uqq.1.B, 6ut3.1.A, 6ut3.1.B, 6ut3.1.C, 6ut3.1.D, 6ut3.1.E, 6ut3.1.F, 6ut4.1.A, 6ut4.1.B, 6ut4.1.C, 6ut4.1.E, 6ut4.1.F, 6ut5.1.A, 6ut5.1.B, 6ut5.1.C, 6ut5.1.D, 6ut5.1.E, 6ut5.1.F, 6ut6.1.A, 6ut6.1.B, 6ut6.1.C, 6ut6.1.D, 6ut6.1.E, 6ut6.1.F, 6ut7.1.A, 6ut7.1.B, 6ut7.1.C, 6ut7.1.D, 6ut7.1.E, 6ut7.1.F, 6uv0.1.A, 6uv0.2.A, 6uv1.1.A, 6uv2.1.A, 6uv4.1.A, 6uxw.1.N, 6v11.1.A, 6v11.1.B, 6v11.1.C, 6v11.1.D, 6v11.1.E, 6v11.1.F, 6v5b.1.A, 6v5c.1.A, 6vff.1.B, 6vfs.1.A, 6vfs.1.B, 6vfs.1.C, 6vfs.1.D, 6vfs.1.E, 6vfs.1.F, 6vfx.1.A, 6vfx.1.B, 6vfx.1.C, 6vfx.1.D, 6vfx.1.F, 6vlz.14.A, 6vlz.72.A, 6vm1.1.A, 6vm1.1.B, 6vm1.1.C, 6vm4.1.A, 6vm4.1.B, 6vm4.1.C, 6vmb.1.A, 6vmb.1.B, 6vmb.1.C, 6vmd.1.A, 6vmd.1.B, 6vmd.1.C, 6vmg.1.H, 6vmg.1.J, 6vmg.1.L, 6vof.1.A, 6vof.1.B, 6vof.1.C, 6voi.1.A, 6voi.1.B, 6voi.1.C, 6voj.1.B, 6vok.1.A, 6vok.1.C, 6vol.1.B, 6vol.1.C, 6vom.1.A, 6von.1.A, 6von.1.B, 6von.1.C, 6vvo.1.A, 6vvo.1.B, 6vvo.1.C, 6vvo.1.D, 6vvo.1.E, 6vz4.1.K, 6w1z.1.O, 6w1z.1.P, 6w1z.1.Q, 6w1z.1.R, 6w1z.1.S, 6w1z.1.T, 6w20.1.O, 6w20.1.P, 6w20.1.Q, 6w20.1.R, 6w20.1.S, 6w20.1.T, 6w21.1.A, 6w21.1.B, 6w21.1.C, 6w21.1.D, 6w21.1.E, 6w21.1.F, 6w6e.1.C, 6w6g.1.E, 6w6g.1.F, 6w6h.1.A, 6w6h.1.B, 6w6h.1.C, 6w6h.1.D, 6w6h.1.E, 6w6h.1.F, 6w6i.1.E, 6w6j.1.F, 6wa8.1.A, 6wa8.1.B, 6wa8.1.C, 6wa8.1.D, 6wa8.1.E, 6wa8.1.F, 6wct.1.A, 6wct.1.B, 6wct.2.B, 6wgc.1.A, 6wgc.1.E, 6wgg.1.C, 6wgh.1.A, 6wgh.2.A, 6wgi.1.H, 6wjd.1.M, 6wjd.1.N, 6wjd.1.O, 6wjd.1.P, 6wjd.1.Q, 6wjd.1.R, 6wjn.1.d, 6wjn.1.e, 6wjn.1.f, 6wjn.1.g, 6wjn.1.h, 6wjn.1.i, 6wqh.1.A, 6wqh.1.B, 6wqh.1.C, 6wqh.1.D, 6wqh.1.E, 6wqh.1.F, 6wrf.1.A, 6wrf.1.B, 6wrf.1.C, 6wrf.1.D, 6wrf.1.E, 6wrf.1.N, 6wsg.1.A, 6wsg.1.B, 6wsg.1.C, 6wsg.1.D, 6wsg.1.E, 6wsg.1.F, 6wvk.1.G, 6x26.1.A, 6x2f.1.A, 6x2n.1.A, 6x43.1.A, 6x4w.1.A, 6x4y.1.A, 6x50.1.A, 6x5z.1.C, 6x61.1.A, 6x61.2.A, 6x61.3.A, 6x61.4.A, 6x61.6.A, 6xaf.1.A, 6xaf.1.B, 6xas.1.L, 6xeo.1.A, 6xez.1.E, 6xez.1.F, 6xki.1.A, 6xtw.1.A, 6xtw.2.A, 6xzt.1.A, 6xzt.2.A, 6y2z.1.A, 6y2z.2.A, 6y30.1.A, 6y30.2.A, 6y31.1.A, 6y31.2.A, 6y53.1.D, 6y5q.1.F, 6ybh.1.A, 6ybu.2.B, 6ycx.1.A, 6ycx.1.B, 6ycz.1.A, 6yhr.1.A, 6ylf.1.A, 6ynz.125.A, 6ynz.147.A, 6ynz.149.A, 6yt1.1.B, 6yvh.1.F, 6yw5.1.X, 6yws.1.c, 6ywv.1.5, 6yxu.1.F, 6yy0.1.C, 6yys.1.F, 6z1e.1.B, 6z1e.1.E, 6z1f.1.A, 6z1f.1.B, 6z1f.1.C, 6z1f.1.E, 6z1f.1.F, 6z1r.1.C, 6z1r.1.E, 6z1u.1.A, 6z1u.1.B, 6z1u.1.C, 6z1u.1.E, 6z2s.1.A, 6z6f.1.D, 6z6h.1.G, 6z6o.1.K, 6z6p.1.D, 6z7t.1.A, 6z7t.2.A, 6z7u.1.A, 6z9p.1.F, 6z9q.1.B, 6z9r.1.C, 6z9s.1.B, 6z9t.1.F, 6zca.1.G, 6zdw.1.A, 6zdw.1.B, 6zfb.1.N, 6zio.1.A, 6zir.1.A, 6zjb.2.A, 6zjb.5.A, 6zm2.1.A, 6zm5.35.A, 6zmw.1.t, 6znp.2.A, 6znq.1.A, 6znq.2.A, 6zns.1.A, 6zpo.1.B, 6zpo.1.C, 6zpo.1.D, 6zpo.1.F, 6zpo.1.G, 6zqb.38.A, 6zqb.39.A, 6zqc.3.A, 6zqc.6.A, 6zqd.37.A, 6zqf.1.O, 6zqg.1.8, 6zqm.1.A, 6zqm.1.B, 6zqn.1.D, 6zsa.38.A, 6zsa.65.A, 6zsb.65.A, 6zsc.65.A, 6zse.65.A, 6zsl.1.A, 6zsl.2.A, 6zww.1.A, 6zww.2.A, 6zww.3.A, 6zww.4.A, 6zwx.1.A, 6zyw.1.A, 6zz6.1.A, 6zz6.1.B, 7a5h.1.7, 7a5i.36.A, 7a5i.76.A, 7a5k.36.A, 7a5k.75.A, 7a5p.1.T, 7a5p.1.d, 7a5p.1.e, 7a5p.1.k, 7a8r.1.A, 7a8r.2.A, 7abg.1.H, 7abr.1.A, 7abr.1.B, 7abr.1.C, 7abr.1.D, 7abr.1.E, 7abr.1.F, 7ad8.1.C, 7ad8.1.E, 7ada.1.A, 7ada.2.A, 7adb.1.F, 7adc.1.B, 7aho.1.A, 7aho.1.B, 7aho.1.C, 7aho.1.D, 7aho.1.E, 7aho.1.F, 7ajf.1.2, 7ajf.1.3, 7ajf.1.4, 7ajf.1.5, 7ajf.1.6, 7ajf.1.7, 7ajf.1.A, 7ajf.1.B, 7ajf.1.C, 7ajf.1.D, 7ajf.1.E, 7ajf.1.F, 7ajt.3.A, 7ajt.79.A, 7aju.74.A, 7akp.1.A, 7aln.1.F, 7alv.1.A, 7am2.64.A, 7amv.1.M, 7aoh.1.M, 7aoi.59.A, 7aor.1.C, 7apd.1.B, 7apd.1.C, 7apd.1.D, 7apd.1.E, 7apd.1.F, 7apd.1.G, 7apk.1.G, 7apx.1.F, 7ase.1.A, 7ask.1.A, 7auc.1.A, 7aud.1.A, 7aud.2.A, 7aud.3.A, 7aud.4.A, 7aud.6.A, 7b19.1.A, 7b1a.1.A, 7b9v.1.E, 7b9v.1.T, 7bbb.1.A, 7bdi.1.A, 7bdj.1.A, 7bdk.1.A, 7bdl.1.A, 7bil.1.A, 7bkp.1.A, 7bkq.1.A, 7blv.1.A, 7bm0.1.A, 7bp8.1.B, 7bp9.1.C, 7bpa.1.D, 7bpb.1.C, 7bst.1.A, 7bst.1.B, 7bto.1.F, 7btp.1.B, 7btq.1.F, 7btr.1.B, 7c98.1.C, 7c99.1.A, 7c9a.1.A, 7c9c.1.A, 7cg3.1.A, 7cg3.1.B, 7cg3.1.C, 7cg3.1.D, 7cg3.1.E, 7cg3.1.F, 7cgy.1.A, 7clg.1.A, 7clg.1.B, 7coq.1.D, 7coq.1.E, 7coq.1.F, 7crc.1.A, 7crc.1.B, 7ctf.1.D, 7ctg.1.D, 7ctg.1.E, 7cxm.1.G, 7cxm.1.H, 7cxn.1.G, 7cxn.1.H, 7cyq.1.H, 7d4i.78.A, 7d5t.1.r, 7dco.1.D, 7dcp.1.A, 7dcq.1.A, 7dcr.1.A, 7dd3.1.A, 7ddx.1.B, 7dey.1.A, 7dey.1.B, 7dey.2.A, 7dey.2.B, 7dfv.1.A, 7dfv.1.B, 7dgr.27.A, 7dgz.1.Y, 7dhw.1.A, 7dtj.1.A, 7dtk.1.A, 7dtk.1.B, 7dvq.1.D, 7dxq.1.D, 7dy1.1.A, 7dye.1.A, 7dyi.1.A, 7dyj.1.A, 7dyk.1.A, 7e6v.1.A, 7e6v.2.A, 7e9v.1.A, 7eey.1.A, 7eey.1.B, 7egb.1.G, 7egc.1.G, 7egc.1.H, 7egp.1.H, 7egq.1.E, 7egq.1.M, 7egq.1.Q, 7egq.1.R, 7egt.1.A, 7eiz.1.J, 7eiz.1.K, 7ej6.1.A, 7ej7.1.A, 7ejc.1.C, 7eje.1.B, 7ejw.1.C, 7ejw.1.D, 7eld.1.A, 7ele.1.A, 7enn.1.A, 7epk.1.A, 7epu.1.B, 7es4.1.A, 7evn.1.E, 7evo.1.L, 7f6j.1.A, 7f6j.1.B, 7fd4.1.A, 7fd4.1.B, 7fd4.1.C, 7fd4.1.D, 7fd4.1.E, 7fd4.1.F, 7fd5.1.A, 7fd5.1.B, 7fd5.1.C, 7fd5.1.D, 7fd5.1.E, 7fd5.1.F, 7fgq.1.A, 7fid.1.A, 7fid.1.B, 7fid.1.C, 7fid.1.D, 7fid.1.E, 7fid.1.F, 7fie.1.A, 7fie.1.B, 7fie.1.C, 7fie.1.D, 7fie.1.E, 7fie.1.F, 7fiz.1.A, 7fiz.1.B, 7fiz.1.C, 7fiz.1.D, 7fiz.1.E, 7fiz.1.F, 7fse.1.A, 7fsf.1.A, 7gqs.1.A, 7gqt.1.A, 7gqu.1.A, 7jg5.1.A, 7jg5.1.B, 7jg5.1.C, 7jg5.1.F, 7jg6.1.A, 7jg6.1.B, 7jg6.1.C, 7jg7.1.A, 7jg7.1.B, 7jg7.1.C, 7jg8.1.B, 7jg8.1.C, 7jg9.1.C, 7jg9.1.F, 7jga.1.A, 7jga.1.B, 7jga.1.C, 7jga.1.F, 7jgr.1.C, 7jgr.1.F, 7jgr.1.G, 7jk2.1.D, 7jk3.1.D, 7jk3.1.G, 7jk5.1.C, 7jk5.1.D, 7jk5.1.F, 7jk6.1.B, 7jk6.1.C, 7jl0.1.C, 7jl3.1.A, 7jlv.1.A, 7jno.1.A, 7jpo.1.A, 7jpo.1.D, 7jpo.1.E, 7jpp.1.D, 7jpp.1.E, 7jpq.1.C, 7jpq.1.D, 7jpr.1.A, 7jpr.1.D, 7jpr.1.E, 7jps.1.A, 7jps.1.D, 7jps.1.E, 7jsf.1.A, 7jsg.1.A, 7jsg.1.B, 7jsg.1.C, 7jsg.1.D, 7jsg.1.E, 7jsg.1.F, 7jsg.1.G, 7jsh.1.A, 7jsh.1.B, 7jsh.1.C, 7jsh.1.D, 7jsh.1.E, 7jsh.1.F, 7jsh.1.G, 7jsi.1.A, 7jsi.1.B, 7jsi.1.C, 7jsi.1.D, 7jsi.1.E, 7jsi.1.F, 7jy5.1.B, 7jy6.1.A, 7jy6.1.B, 7jy6.1.H, 7jy6.1.I, 7jy7.1.A, 7jy7.1.H, 7jy8.1.A, 7jy9.1.C, 7jy9.1.D, 7k01.1.C, 7k04.1.J, 7k56.1.F, 7k57.1.A, 7kch.1.B, 7kek.1.B, 7krn.1.E, 7kro.1.F, 7krz.1.A, 7krz.1.B, 7krz.1.C, 7krz.1.D, 7krz.1.E, 7krz.1.F, 7ksl.1.A, 7ksl.1.B, 7ksl.1.C, 7ksl.1.D, 7ksl.1.E, 7ksm.1.A, 7ksm.1.B, 7ksm.1.C, 7ksm.1.D, 7ksm.1.E, 7ksm.1.F, 7l1q.1.A, 7l1q.1.B, 7l1q.1.C, 7l1q.1.D, 7l1q.1.E, 7l1q.1.F, 7l1r.1.A, 7l1r.1.B, 7l1r.1.C, 7l1r.1.D, 7l1r.1.E, 7l1r.1.F, 7l5w.1.L, 7l5x.1.D, 7l6n.1.A, 7l6n.1.B, 7l6n.1.D, 7l6n.1.F, 7l9p.1.A, 7l9p.1.B, 7l9p.1.C, 7l9p.1.D, 7l9p.1.E, 7l9p.1.F, 7l9x.1.A, 7lar.1.A, 7lar.1.B, 7lar.1.C, 7lar.1.D, 7lar.1.E, 7lar.1.F, 7lbm.1.0, 7lbm.1.1, 7lcc.1.A, 7liu.1.A, 7ljf.1.A, 7ljf.1.B, 7ljf.1.C, 7ljf.1.F, 7lmy.1.A, 7lmz.1.A, 7lmz.1.B, 7lmz.1.C, 7lmz.1.D, 7lmz.1.E, 7lmz.1.F, 7ln0.1.A, 7ln0.1.B, 7ln0.1.F, 7ln1.1.A, 7ln1.1.B, 7ln1.1.C, 7ln1.1.D, 7ln1.1.E, 7ln1.1.F, 7ln2.1.A, 7ln2.1.B, 7ln2.1.C, 7ln2.1.D, 7ln2.1.E, 7ln2.1.F, 7ln3.1.A, 7ln3.1.B, 7ln3.1.F, 7ln4.1.A, 7ln4.1.B, 7ln4.1.C, 7ln4.1.D, 7ln4.1.E, 7ln4.1.F, 7ln5.1.A, 7ln5.1.B, 7ln5.1.C, 7ln5.1.E, 7ln5.1.F, 7ln6.1.A, 7ln6.1.B, 7ln6.1.C, 7ln6.1.D, 7ln6.1.E, 7ln6.1.F, 7luv.1.F, 7luy.1.A, 7luy.1.B, 7luy.2.B, 7m6j.1.A, 7m6j.1.B, 7m6j.1.C, 7m6j.1.D, 7m6j.1.E, 7m6j.1.F, 7m8e.1.F, 7m99.1.E, 7m9a.1.C, 7m9a.1.L, 7m9b.1.A, 7m9b.1.D, 7m9b.1.K, 7m9c.1.B, 7m9c.1.E, 7m9c.1.F, 7m9c.1.G, 7m9c.1.H, 7m9c.1.I, 7m9c.1.J, 7mbw.1.A, 7mbw.1.B, 7mbw.2.A, 7mbw.2.B, 7mca.1.A, 7mca.1.D, 7mca.1.E, 7mca.1.I, 7mcs.1.A, 7mcs.1.B, 7mcs.1.C, 7mcs.1.D, 7mcs.1.E, 7mcs.1.F, 7mcs.1.G, 7md3.1.A, 7md3.1.B, 7md3.1.C, 7md3.1.D, 7md3.1.E, 7md3.1.F, 7mdm.1.A, 7mdm.1.B, 7mdm.1.C, 7mdm.1.D, 7mdm.1.E, 7mdm.1.F, 7mdo.1.C, 7mgm.1.A, 7mhb.1.A, 7mhs.1.A, 7mhs.1.B, 7mhs.1.C, 7mhs.1.D, 7mhs.1.E, 7mi1.1.A, 7mi3.1.A, 7mkn.1.F, 7mkq.1.F, 7ml0.1.1, 7ml0.1.U, 7ml1.1.C, 7ml1.1.G, 7ml2.1.2, 7ml2.1.Y, 7ml3.1.C, 7ml3.1.H, 7ml4.1.Q, 7ml4.1.U, 7mq8.38.A, 7mq9.33.A, 7mq9.67.A, 7mqj.1.A, 7mr0.1.A, 7mr1.1.A, 7mr1.1.C, 7mr2.1.A, 7mr3.1.A, 7mr3.1.C, 7mr4.1.A, 7nac.1.v, 7nep.1.P, 7nep.1.S, 7nfx.1.q, 7nfy.1.A, 7nfy.1.B, 7nfy.1.C, 7nfy.1.D, 7nfy.1.E, 7nfy.1.F, 7ng4.1.A, 7ng4.1.B, 7ng4.1.C, 7ng4.1.D, 7ng4.1.E, 7ng4.1.F, 7ng5.1.A, 7ng5.1.B, 7ng5.1.C, 7ng5.1.D, 7ng5.1.E, 7ng5.1.F, 7nga.1.C, 7ngc.1.A, 7ngc.1.B, 7ngc.1.C, 7ngc.1.D, 7ngc.1.E, 7ngc.1.F, 7ngf.1.A, 7ngf.1.B, 7ngf.1.C, 7ngf.1.D, 7ngf.1.E, 7ngf.1.F, 7ngl.1.A, 7ngl.1.B, 7ngl.1.C, 7ngl.1.D, 7ngl.1.E, 7ngl.1.F, 7ngp.1.A, 7ngp.1.C, 7ngp.1.D, 7ngp.1.E, 7ngp.1.F, 7ngq.1.A, 7ngq.1.B, 7ngq.1.C, 7ngq.1.D, 7ngq.1.E, 7ngq.1.F, 7nic.1.A, 7nio.1.A, 7nio.1.B, 7niq.1.A, 7njl.1.A, 7njl.1.B, 7njl.1.C, 7njm.1.C, 7njn.1.C, 7njo.1.C, 7njq.1.C, 7nku.1.E, 7nkx.1.U, 7nn0.1.A, 7nn0.2.A, 7nn0.3.A, 7nn0.4.A, 7nsh.1.K, 7nsj.72.A, 7nvv.1.C, 7o41.1.C, 7o41.1.Z, 7o42.1.A, 7o42.1.B, 7o42.1.C, 7o42.1.D, 7o42.1.E, 7o42.1.F, 7o43.1.A, 7o43.1.B, 7o4i.1.H, 7o4j.1.H, 7o4l.1.H, 7o5b.1.C, 7o72.1.A, 7o72.1.H, 7o73.1.H, 7o75.1.H, 7o9g.1.A, 7o9i.1.A, 7o9k.40.A, 7oar.1.A, 7oar.1.B, 7oat.1.B, 7obq.1.F, 7obr.1.p, 7of2.1.b, 7ohp.1.F, 7ohr.1.G, 7ohs.1.J, 7ohv.1.G, 7ohw.1.J, 7ohx.1.I, 7oi6.1.e, 7oi7.1.7, 7oi9.1.7, 7oib.1.7, 7oic.1.a, 7oid.1.8, 7oie.1.8, 7ole.1.A, 7ole.1.B, 7ole.1.C, 7ole.1.D, 7ole.1.E, 7ole.1.F, 7oo3.1.Q, 7oob.1.M, 7oop.1.X, 7opc.1.X, 7oqb.1.T, 7oqh.1.A, 7oqh.1.B, 7oqh.1.C, 7oqh.1.D, 7oqh.1.E, 7oqh.1.F, 7os1.1.A, 7os2.1.A, 7otj.1.A, 7otj.2.A, 7otq.1.A, 7ovb.1.A, 7owe.1.A, 7owe.1.B, 7owe.1.C, 7owe.2.A, 7owe.2.B, 7owe.2.C, 7owh.1.A, 7owh.1.B, 7owh.1.C, 7owh.2.A, 7owh.2.C, 7owj.1.B, 7owj.1.C, 7owj.2.B, 7owk.1.A, 7owk.1.B, 7owk.1.C, 7owk.2.A, 7owk.2.B, 7owk.2.C, 7owl.1.C, 7owl.2.A, 7oxo.1.A, 7oxo.1.B, 7oxo.1.C, 7oxo.1.D, 7oxo.1.E, 7oxo.1.F, 7p09.1.A, 7p09.1.B, 7p09.1.C, 7p09.1.D, 7p09.1.E, 7p09.1.F, 7p0b.1.A, 7p0b.1.B, 7p0b.1.C, 7p0b.1.D, 7p0b.1.E, 7p0b.1.F, 7p0m.1.A, 7p0m.1.B, 7p0m.1.C, 7p0m.1.D, 7p0m.1.E, 7p0m.1.F, 7p2y.1.B, 7p2y.1.C, 7p2y.1.D, 7p2y.1.E, 7p2y.1.F, 7p3n.1.A, 7p3n.1.B, 7p3n.1.C, 7p3n.1.D, 7p3n.1.E, 7p3n.1.F, 7p3w.1.A, 7p3w.1.B, 7p3w.1.C, 7p3w.1.D, 7p3w.1.E, 7p3w.1.F, 7p6u.1.A, 7p6u.1.B, 7p6u.1.C, 7p6u.1.D, 7p6u.1.E, 7p6u.1.F, 7p6x.1.A, 7p6x.1.B, 7p6x.1.C, 7p6x.1.D, 7p6x.1.E, 7p6x.1.F, 7pbl.1.A, 7pbl.1.B, 7pbl.1.C, 7pbl.1.D, 7pbl.1.E, 7pbl.1.F, 7pbm.1.E, 7pbo.1.D, 7pbo.1.E, 7pbp.1.D, 7pbp.1.F, 7pbq.1.D, 7pbq.1.E, 7pbr.1.D, 7pbt.1.C, 7pbt.1.E, 7pd3.1.b, 7pkq.1.f, 7plh.1.A, 7pli.1.C, 7pli.2.A, 7pli.2.C, 7plt.1.A, 7plw.1.B, 7plx.1.B, 7pm0.1.B, 7pm1.1.B, 7pm2.1.B, 7pm5.1.B, 7pm7.1.B, 7pm8.1.A, 7pm9.1.B, 7pma.1.B, 7pmb.1.B, 7pmc.1.B, 7pmf.1.B, 7pmg.1.A, 7pmi.1.A, 7pmj.1.B, 7pml.1.B, 7pmm.1.B, 7pmq.1.A, 7pmq.2.B, 7ppj.1.A, 7ppz.1.B, 7pq0.1.B, 7pux.1.F, 7px3.1.A, 7px9.1.A, 7px9.1.C, 7px9.1.D, 7px9.1.E, 7px9.1.F, 7pxb.1.A, 7pxb.1.B, 7pxb.1.C, 7pxb.1.D, 7pxc.1.G, 7pxc.1.H, 7pxc.1.I, 7pxc.1.K, 7pxd.1.K, 7pxd.1.L, 7qdr.1.A, 7qdz.1.A, 7qe0.1.A, 7qh6.1.4, 7qh7.1.5, 7qin.1.L, 7qo4.1.B, 7qo4.1.C, 7qo4.1.D, 7qo4.1.E, 7qo4.1.F, 7qo4.1.G, 7qo5.1.f, 7qo5.1.g, 7qo5.1.h, 7qo5.1.i, 7qo5.1.j, 7qo5.1.k, 7qo6.1.f, 7qo6.1.g, 7qo6.1.h, 7qo6.1.i, 7qo6.1.j, 7qsd.1.O, 7qso.1.O, 7qtt.1.K, 7qv8.1.A, 7qv9.1.H, 7qv9.1.I, 7qv9.1.J, 7qv9.1.K, 7qv9.1.L, 7qv9.1.M, 7qwq.1.F, 7qxm.1.A, 7qxm.1.B, 7qxm.1.E, 7qxm.1.F, 7qxn.1.O, 7qxn.1.R, 7qxp.1.N, 7qxu.1.N, 7qxu.1.O, 7qxu.1.P, 7qxw.1.L, 7qxw.1.M, 7qxw.1.N, 7qxx.1.Q, 7qy7.1.M, 7qy7.1.N, 7qy7.1.O, 7qy7.1.P, 7qy7.1.Q, 7qy7.1.R, 7qya.1.M, 7qya.1.N, 7qya.1.O, 7qya.1.P, 7qya.1.Q, 7qya.1.R, 7r2k.1.A, 7r4g.1.O, 7r6q.1.B, 7r76.1.A, 7r77.1.A, 7r78.1.A, 7r7j.1.A, 7r7j.2.A, 7r7s.1.A, 7r7s.1.B, 7r7s.1.C, 7r7s.1.D, 7r7s.1.E, 7r7s.1.F, 7r7t.1.A, 7r7t.1.B, 7r7t.1.C, 7r7t.1.D, 7r7t.1.E, 7r7t.1.F, 7r7u.1.A, 7r7u.1.B, 7r7u.1.C, 7r7u.1.D, 7r7u.1.E, 7r7u.1.F, 7r91.1.D, 7r97.1.A, 7rb8.1.B, 7rb9.1.B, 7rdx.1.E, 7rdx.1.F, 7rdy.1.E, 7rdy.1.F, 7rdz.1.E, 7rdz.1.F, 7re0.1.E, 7re0.1.F, 7re1.1.E, 7re1.1.F, 7re2.1.E, 7re3.1.F, 7re3.1.M, 7rl6.1.A, 7rl7.1.A, 7rl9.1.A, 7rla.1.A, 7rlb.1.A, 7rlc.1.A, 7rld.1.A, 7rlf.1.A, 7rlg.1.A, 7rlh.1.A, 7rli.1.A, 7rlj.1.L, 7rzy.1.A, 7s65.1.A, 7s65.1.B, 7s65.1.C, 7s66.1.A, 7s67.1.E, 7s7b.1.A, 7s7c.1.A, 7s9v.1.A, 7s9w.1.A, 7sgz.1.A, 7sgz.1.B, 7sgz.1.C, 7sgz.1.D, 7sgz.1.E, 7sh2.1.A, 7sh2.1.B, 7sh2.1.C, 7sh2.1.E, 7sjr.1.A, 7sjr.1.B, 7sql.1.A, 7sql.1.B, 7sql.1.C, 7sql.1.D, 7ssg.1.A, 7stb.1.A, 7stb.1.B, 7stb.1.C, 7stb.1.D, 7stb.1.E, 7ste.1.A, 7ste.1.B, 7ste.1.C, 7ste.1.D, 7ste.1.E, 7suk.62.A, 7suk.63.A, 7svu.1.R, 7svv.1.K, 7swl.1.A, 7swl.1.B, 7swl.1.D, 7swl.1.E, 7swl.1.F, 7sxo.1.A, 7sxo.1.B, 7sxo.1.C, 7sxo.1.D, 7sxo.1.E, 7sxo.1.F, 7t02.1.A, 7t0v.1.A, 7t0v.1.B, 7t0v.1.C, 7t0v.1.D, 7t0v.1.E, 7t0v.1.F, 7t20.1.A, 7t20.1.B, 7t20.1.C, 7t20.1.D, 7t20.1.E, 7t20.1.F, 7t21.1.A, 7t21.1.B, 7t21.1.C, 7t21.1.D, 7t21.1.E, 7t21.1.F, 7t22.1.A, 7t22.1.B, 7t22.1.C, 7t22.1.D, 7t22.1.E, 7t22.1.F, 7t3i.1.A, 7t3i.1.B, 7t3i.1.C, 7t3i.1.D, 7t3i.1.E, 7t3i.1.F, 7tdo.1.A, 7tfh.1.A, 7tfh.1.E, 7tfi.1.A, 7tfi.1.B, 7tfi.1.C, 7tfi.1.D, 7tfi.1.E, 7tfj.1.A, 7tfj.1.B, 7tfj.1.C, 7tfj.1.E, 7tfk.1.A, 7tfk.1.B, 7tfk.1.C, 7tfk.1.D, 7tfk.1.E, 7tfl.1.A, 7tfl.1.B, 7tfl.1.C, 7tfl.1.D, 7tfl.1.E, 7thj.1.A, 7thj.1.C, 7thj.1.D, 7thj.1.E, 7thv.1.A, 7thv.1.C, 7thv.1.D, 7thv.1.E, 7thy.1.A, 7thz.1.A, 7ti8.1.A, 7ti8.1.E, 7tib.1.A, 7tib.1.C, 7tic.1.A, 7tic.1.B, 7tic.1.C, 7tic.1.D, 7tic.1.E, 7tid.1.A, 7tid.1.B, 7tjf.1.A, 7tjh.1.E, 7tjh.1.I, 7tji.1.A, 7tji.1.D, 7tji.1.E, 7tji.1.I, 7tjj.1.A, 7tjj.1.I, 7tju.1.A, 7tju.1.B, 7tju.1.D, 7tjv.1.E, 7tjw.1.C, 7tjw.1.E, 7tjx.1.B, 7tjx.1.E, 7tk0.1.K, 7tk1.1.K, 7tk1.1.N, 7tk1.1.O, 7tk2.1.K, 7tk2.1.L, 7tk5.1.K, 7tk5.1.L, 7tk5.1.M, 7tk5.1.N, 7tk5.1.O, 7tk5.1.P, 7tk7.1.N, 7tk7.1.O, 7tk9.1.L, 7tka.1.N, 7tkb.1.K, 7tkb.1.M, 7tkc.1.N, 7tkd.1.K, 7tkd.1.L, 7tkd.1.M, 7tkd.1.N, 7tkd.1.O, 7tkd.1.P, 7tke.1.L, 7tke.1.N, 7tke.1.O, 7tkf.1.K, 7tkf.1.M, 7tkg.1.K, 7tki.1.K, 7tki.1.L, 7tki.1.M, 7tki.1.O, 7tki.1.P, 7tkj.1.K, 7tkj.1.L, 7tkj.1.M, 7tkj.1.N, 7tkj.1.O, 7tkj.1.P, 7tkk.1.K, 7tkk.1.L, 7tkk.1.M, 7tkk.1.N, 7tkk.1.O, 7tkk.1.P, 7tkl.1.L, 7tkl.1.M, 7tkm.1.P, 7tkn.1.K, 7tkn.1.L, 7tkn.1.M, 7tkn.1.N, 7tkn.1.O, 7tkn.1.P, 7tkp.1.K, 7tkp.1.L, 7tkr.1.K, 7tkr.1.L, 7tkr.1.N, 7tkr.1.O, 7tkr.1.P, 7tks.1.K, 7tks.1.L, 7tks.1.M, 7tks.1.N, 7tks.1.O, 7tks.1.P, 7tku.1.A, 7tku.1.B, 7tku.1.C, 7tku.1.E, 7tle.1.A, 7tlk.1.A, 7tlk.2.A, 7tn2.1.K, 7tnx.1.A, 7tny.1.A, 7tnz.1.A, 7to0.1.A, 7to1.1.A, 7to2.1.A, 7tr8.1.A, 7tr9.1.P, 7tra.1.A, 7ttr.1.A, 7ttr.1.B, 7ttr.1.C, 7ttr.1.D, 7ttr.1.E, 7ttr.1.F, 7u19.1.A, 7u19.1.D, 7u19.1.E, 7u1a.1.A, 7u1a.1.C, 7u1a.1.D, 7u1a.1.E, 7u1p.1.A, 7u1p.1.B, 7u1p.1.C, 7udt.1.E, 7udu.1.D, 7ufi.1.C, 7ufm.1.A, 7uiv.1.A, 7uiv.1.B, 7uiv.1.C, 7uiv.1.D, 7uiv.1.E, 7uiv.1.F, 7uiw.1.A, 7uiw.1.B, 7uiw.1.C, 7uiw.1.D, 7uiw.1.E, 7uiw.1.F, 7uix.1.A, 7uix.1.B, 7uix.1.C, 7uix.1.D, 7uix.1.E, 7uix.1.F, 7uiy.1.A, 7uiy.1.B, 7uiy.1.C, 7uiy.1.D, 7uiy.1.E, 7uiy.1.F, 7uiz.1.A, 7uiz.1.B, 7uiz.1.C, 7uiz.1.D, 7uiz.1.E, 7uiz.1.F, 7uj0.1.A, 7uj0.1.B, 7uj0.1.C, 7uj0.1.D, 7uj0.1.E, 7uj0.1.F, 7ujb.1.A, 7upr.1.A, 7upr.1.B, 7upr.1.C, 7upr.1.E, 7upr.1.F, 7upt.1.A, 7upt.1.B, 7upt.1.C, 7upt.1.D, 7upt.1.E, 7upt.1.F, 7uqi.1.A, 7uqi.1.B, 7uqi.1.C, 7uqi.1.D, 7uqi.1.E, 7uqi.1.F, 7uqj.1.A, 7uqj.1.B, 7uqj.1.C, 7uqj.1.D, 7uqj.1.E, 7uqj.1.F, 7ux9.1.I, 7v2b.1.A, 7v2b.1.B, 7v2c.1.i, 7v2d.1.i, 7v2v.1.A, 7v2v.1.B, 7v2y.1.F, 7v2z.1.A, 7v3w.1.A, 7v3w.1.B, 7v4e.1.A, 7v4e.1.B, 7v4q.1.A, 7v4r.1.A, 7v6b.1.A, 7v6b.1.B, 7v6c.1.A, 7vbs.1.A, 7vcs.1.A, 7vct.1.A, 7vcu.1.A, 7vcv.1.A, 7vcx.1.A, 7vdt.1.A, 7vdv.1.J, 7vg2.1.A, 7vg3.1.A, 7vsr.1.A, 7vsr.1.B, 7vsr.1.C, 7vsr.1.D, 7vsr.1.E, 7vsr.1.F, 7w0a.1.A, 7w0b.1.A, 7w0c.1.A, 7w0d.1.D, 7w0d.1.E, 7w0e.1.C, 7w0f.1.A, 7w1r.1.A, 7w37.1.A, 7w37.1.B, 7w37.1.C, 7w37.1.D, 7w37.1.E, 7w37.1.F, 7w38.1.C, 7w38.1.D, 7w38.1.E, 7w38.1.j, 7w39.1.A, 7w39.1.B, 7w39.1.C, 7w39.1.D, 7w39.1.E, 7w39.1.F, 7w3a.1.A, 7w3a.1.B, 7w3a.1.C, 7w3a.1.D, 7w3a.1.E, 7w3a.1.F, 7w3b.1.A, 7w3b.1.B, 7w3b.1.C, 7w3b.1.D, 7w3b.1.E, 7w3b.1.F, 7w3c.1.A, 7w3c.1.B, 7w3c.1.C, 7w3c.1.D, 7w3c.1.E, 7w3c.1.F, 7w3f.1.A, 7w3f.1.B, 7w3f.1.C, 7w3f.1.D, 7w3f.1.E, 7w3f.1.F, 7w3g.1.A, 7w3g.1.B, 7w3g.1.C, 7w3g.1.D, 7w3g.1.E, 7w3g.1.F, 7w3h.1.A, 7w3h.1.B, 7w3h.1.C, 7w3h.1.D, 7w3h.1.E, 7w3h.1.F, 7w3i.1.B, 7w3i.1.C, 7w3i.1.D, 7w3i.1.E, 7w3i.1.F, 7w3i.1.G, 7w3j.1.6, 7w3j.1.7, 7w3j.1.8, 7w3j.1.9, 7w3j.1.A, 7w3j.1.B, 7w3k.1.8, 7w3k.1.9, 7w3k.1.A, 7w3k.1.B, 7w3k.1.C, 7w3k.1.D, 7w3m.1.A, 7w3m.1.B, 7w3m.1.C, 7w3m.1.D, 7w3m.1.E, 7w3m.1.F, 7w42.1.B, 7w46.1.C, 7w46.1.D, 7w59.1.Y, 7w5a.1.Y, 7w5b.1.Y, 7wbb.1.A, 7wbb.1.B, 7wbb.1.C, 7wbb.1.D, 7wbb.1.E, 7wbb.1.G, 7wbt.1.A, 7wbt.1.B, 7wbu.1.A, 7wd3.1.A, 7wd3.1.B, 7wd3.1.C, 7wd3.1.E, 7wd3.1.F, 7wd4.1.A, 7wdc.1.A, 7wi4.1.A, 7wi4.1.B, 7wi4.1.C, 7wi4.1.D, 7wi4.1.E, 7wi4.1.F, 7wub.1.B, 7wub.1.C, 7wub.1.D, 7wub.1.J, 7x1y.1.A, 7x1z.1.A, 7x2r.1.A, 7x2r.1.B, 7x2r.1.C, 7x2r.1.D, 7x2r.1.E, 7x2r.1.F, 7x3w.1.K, 7x5b.1.A, 7x7p.1.C, 7x7p.1.E, 7x7p.1.G, 7x7p.1.I, 7x7q.1.I, 7x7q.1.J, 7x7q.1.K, 7x7q.1.L, 7x7s.1.A, 7xe0.1.E, 7xex.1.A, 7xex.2.A, 7xex.3.A, 7xf0.1.A, 7xf0.2.A, 7xf0.3.A, 7xf1.1.A, 7xg3.1.L, 7xha.1.A, 7xhb.1.A, 7xkh.1.A, 7xkh.1.B, 7xkh.1.C, 7xkh.1.D, 7xkh.1.E, 7xkh.1.F, 7xko.1.B, 7xko.1.C, 7xkp.1.A, 7xkp.1.B, 7xkp.1.C, 7xkp.1.D, 7xkp.1.E, 7xkq.1.A, 7xkq.1.B, 7xkq.1.C, 7xkq.1.D, 7xkq.1.E, 7xkr.1.A, 7xkr.1.C, 7xkr.1.D, 7xt0.1.A, 7xt3.1.A, 7xw2.1.A, 7xw3.1.A, 7xwy.1.A, 7xx2.1.A, 7xx2.1.C, 7xx2.1.D, 7xx2.1.E, 7xx2.1.F, 7xxe.1.A, 7xxe.2.A, 7xyf.1.G, 7xyg.1.G, 7y38.1.N, 7y4w.1.E, 7y4w.1.F, 7y4w.1.G, 7y4w.1.H, 7y4w.1.I, 7y4w.1.J, 7y53.1.E, 7y53.1.F, 7y53.1.G, 7y53.1.H, 7y53.1.I, 7y53.1.J, 7y59.1.E, 7y59.1.F, 7y59.1.G, 7y59.1.H, 7y59.1.I, 7y59.1.J, 7y5a.1.A, 7y5a.1.B, 7y5a.1.C, 7y5a.1.D, 7y5a.1.E, 7y5a.1.F, 7y5b.1.A, 7y5b.1.B, 7y5b.1.C, 7y5b.1.D, 7y5b.1.E, 7y5b.1.F, 7y5c.1.A, 7y5c.1.B, 7y5c.1.C, 7y5d.1.M, 7y5d.1.N, 7y5d.1.O, 7y8r.1.J, 7ykk.1.A, 7ykk.1.B, 7ykk.1.C, 7ykk.1.D, 7ykk.1.E, 7ykk.1.F, 7ykl.1.A, 7ykl.1.B, 7ykl.1.C, 7ykl.1.D, 7ykl.1.E, 7ykl.1.F, 7ykt.1.A, 7ykt.1.B, 7ykt.1.C, 7ykt.1.D, 7ykt.1.E, 7ykt.1.F, 7ykz.1.A, 7ykz.1.B, 7ykz.1.C, 7ykz.1.D, 7ykz.1.E, 7ykz.1.F, 7ymf.1.A, 7ymf.1.B, 7yph.1.A, 7yph.1.B, 7yph.1.C, 7yph.1.D, 7yph.1.E, 7ypi.1.A, 7ypi.1.B, 7ypi.1.C, 7ypi.1.D, 7ypi.1.E, 7ypi.1.F, 7ypj.1.A, 7ypj.1.B, 7ypj.1.C, 7ypj.1.D, 7ypj.1.E, 7ypk.1.B, 7ypk.1.C, 7ypk.1.D, 7ypk.1.E, 7ypk.1.F, 7ypk.1.G, 7yry.1.A, 7yry.1.B, 7yry.1.C, 7yry.1.D, 7yry.1.E, 7yry.1.F, 7yuh.1.A, 7yuh.1.B, 7yuh.1.C, 7yum.1.A, 7yum.1.B, 7yum.1.C, 7yum.1.D, 7yup.1.A, 7yup.1.B, 7yup.1.C, 7yup.1.D, 7yup.1.E, 7yut.1.A, 7yut.1.B, 7yut.1.C, 7yut.1.D, 7yut.1.E, 7yut.1.F, 7yuu.1.A, 7yuu.1.B, 7yuu.1.C, 7yuv.1.A, 7yuv.1.B, 7yuv.1.C, 7yuv.1.D, 7yuw.1.A, 7yuw.1.B, 7yuw.1.C, 7yuw.1.D, 7yuw.1.E, 7yux.1.A, 7yux.1.B, 7yux.1.C, 7yux.1.D, 7yux.1.E, 7yux.1.F, 7ywa.1.B, 7ywa.1.C, 7yym.1.A, 7yyn.1.B, 7yz4.1.A, 7z11.1.A, 7z11.1.B, 7z11.1.C, 7z11.1.D, 7z11.1.E, 7z11.1.F, 7z34.1.A, 7z34.1.h, 7z34.1.i, 7z34.1.n, 7z34.1.q, 7z34.1.r, 7z3m.1.A, 7z4y.1.B, 7z4y.1.D, 7z52.1.A, 7z67.1.A, 7z6h.1.D, 7z6h.1.E, 7z6h.1.F, 7z6h.1.G, 7z6h.1.H, 7z7n.1.D, 7z8f.1.0, 7z8f.1.8, 7z8f.1.Z, 7z8g.1.A, 7z8s.1.D, 7zb5.1.D, 7zbh.1.A, 7zel.1.A, 7zep.1.A, 7zi4.1.A, 7zi4.1.B, 7zi4.1.C, 7zi4.1.D, 7zi4.1.E, 7zi4.1.F, 7zi4.1.G, 7zke.1.D, 7zml.1.A, 7zml.2.A, 7zmm.4.A, 7zmn.1.A, 7zmn.2.A, 7zmo.2.A, 7zmp.1.A, 7zmp.2.A, 7zmq.1.A, 7zmq.2.A, 7zms.1.A, 7zmt.1.A, 7znj.1.A, 7znk.1.G, 7zpi.1.A, 7zpj.1.A, 7zpj.1.C, 7zpk.1.C, 7zpq.75.A, 7zrs.75.A, 7zsa.1.3, 7zsb.1.3, 7zuw.75.A, 8a3v.1.A, 8a3v.1.H, 8a8u.1.A, 8a8u.1.B, 8a8u.1.C, 8a8u.1.D, 8a8u.1.E, 8a8u.1.F, 8a8v.1.A, 8a8v.1.B, 8a8v.1.C, 8a8v.1.D, 8a8v.1.E, 8a8v.1.F, 8a8w.1.A, 8a8w.1.B, 8a8w.1.C, 8a8w.1.D, 8a8w.1.E, 8a8w.1.F, 8act.1.A, 8act.1.B, 8alz.1.B, 8amd.1.D, 8amf.1.D, 8amz.1.A, 8amz.1.B, 8amz.1.C, 8amz.1.D, 8amz.1.E, 8amz.1.F, 8ark.1.A, 8ark.2.A, 8ark.3.A, 8arp.1.A, 8arp.1.B, 8arp.1.C, 8arp.1.D, 8arp.1.E, 8arp.1.F, 8atf.1.A, 8av6.1.G, 8b0a.1.A, 8b1r.1.A, 8b1r.1.C, 8b1t.1.A, 8b1t.1.C, 8b3d.1.R, 8b3f.1.R, 8b5r.1.A, 8b5r.1.B, 8b5r.1.C, 8b5r.1.D, 8b5r.1.E, 8b5r.1.F, 8b9g.1.A, 8b9i.1.A, 8b9j.1.A, 8b9k.1.A, 8b9l.1.A, 8b9z.1.O, 8ba0.1.O, 8bc8.1.A, 8bc9.1.A, 8bca.1.A, 8bcb.1.A, 8bcc.1.A, 8bcd.1.A, 8bce.1.A, 8bcf.1.A, 8bcg.1.A, 8bch.1.A, 8bd4.1.B, 8bd5.1.E, 8bd5.1.F, 8bd5.1.G, 8bd5.1.H, 8bd5.1.I, 8bd5.1.J, 8bns.1.A, 8bns.1.B, 8bns.2.A, 8bns.2.B, 8bnv.1.A, 8bnx.1.A, 8bnx.2.A, 8bob.1.C, 8bq2.1.H, 8bqf.1.A, 8br9.1.A, 8bsc.1.G, 8btg.1.A, 8btg.1.B, 8btg.1.C, 8btg.1.D, 8btg.1.E, 8btg.1.F, 8btg.1.G, 8bv0.1.A, 8bv3.1.A, 8bvw.1.A, 8bvw.1.B, 8bwy.1.A, 8bx8.1.C, 8byq.1.A, 8byq.1.B, 8c0v.1.A, 8c0v.1.B, 8c0v.1.C, 8c0v.1.D, 8c0v.1.E, 8c0v.1.F, 8c0w.1.A, 8c0w.1.B, 8c0w.1.C, 8c0w.1.D, 8c0w.1.E, 8c0w.1.F, 8c3j.2.A, 8c3n.1.A, 8c8h.1.L, 8cen.1.H, 8ceo.1.A, 8ch6.1.9, 8ch6.1.U, 8ch6.1.v, 8cnt.1.A, 8cqz.1.A, 8cr1.1.C, 8cr2.1.A, 8cr2.1.B, 8cst.1.3, 8cvt.1.A, 8cvt.1.B, 8cvt.1.C, 8cvt.1.D, 8cvt.1.E, 8cvt.1.F, 8d6y.1.A, 8d6y.1.B, 8d6y.1.C, 8d6y.1.D, 8d6y.1.E, 8d6y.1.F, 8dar.1.A, 8dar.1.B, 8dar.1.C, 8dar.1.D, 8dar.1.E, 8dar.1.F, 8dav.1.B, 8dav.1.D, 8daw.1.A, 8daw.1.C, 8daw.1.E, 8daw.1.F, 8db3.1.A, 8db3.1.B, 8db3.1.C, 8dba.1.A, 8dba.1.B, 8dba.1.C, 8dba.1.D, 8dba.1.E, 8dba.1.F, 8dba.1.G, 8dba.1.H, 8dba.1.I, 8dba.1.J, 8dba.1.K, 8dba.1.L, 8dbq.1.A, 8dbq.1.C, 8dbq.1.D, 8dbq.1.E, 8dbq.1.F, 8dbr.1.E, 8dbs.1.A, 8dbv.1.D, 8dbw.1.A, 8dbw.1.B, 8dbw.1.C, 8dfv.1.A, 8dfv.1.C, 8dg5.1.A, 8dg5.1.C, 8dg7.1.A, 8dg7.1.D, 8dga.1.A, 8dga.1.D, 8dgc.1.E, 8dgi.1.A, 8dgi.1.B, 8dgj.1.A, 8dgj.1.B, 8dkt.1.A, 8dol.1.A, 8dol.1.B, 8dol.1.C, 8dol.1.D, 8dol.1.E, 8dol.1.F, 8dpb.1.A, 8dpb.1.B, 8dpe.1.A, 8dqw.1.A, 8dqw.1.B, 8dqw.1.D, 8dqw.1.H, 8dqx.1.A, 8dqx.1.B, 8dqx.1.E, 8dqz.1.A, 8dqz.1.B, 8dr0.1.A, 8dr0.1.C, 8dr0.1.D, 8dr1.1.A, 8dr1.1.B, 8dr3.1.B, 8dr3.1.C, 8dr4.1.A, 8dr4.1.B, 8dr4.1.C, 8dr6.1.E, 8dr7.1.A, 8dr7.1.B, 8dr7.1.E, 8dtp.1.A, 8dtp.1.C, 8dtp.1.D, 8dtp.1.E, 8dtp.1.F, 8dtp.1.G, 8due.1.A, 8due.1.B, 8due.1.C, 8due.1.D, 8due.1.E, 8due.1.F, 8duo.1.A, 8duo.1.B, 8duo.1.C, 8duo.1.D, 8duo.1.E, 8duo.1.F, 8dvs.1.A, 8dyu.1.A, 8dyv.1.A, 8dze.1.A, 8dzf.1.A, 8dzg.1.A, 8dzz.1.A, 8e0f.1.B, 8e2w.1.A, 8e7v.1.C, 8e7v.1.E, 8e7v.1.F, 8e8q.1.A, 8e8q.1.C, 8e91.1.A, 8e91.1.B, 8e91.1.C, 8e91.1.D, 8e91.1.E, 8e91.1.F, 8ea4.1.C, 8ea4.1.I, 8ea4.1.J, 8ea4.1.M, 8eaf.1.A, 8eaf.1.D, 8eag.1.B, 8eag.1.E, 8eag.1.F, 8eah.1.E, 8eai.1.C, 8eai.1.F, 8eal.1.B, 8eal.1.F, 8eam.1.D, 8ebs.1.B, 8ebt.1.A, 8ebt.1.B, 8ebu.1.A, 8ebu.1.B, 8ebw.1.A, 8efd.1.A, 8efe.1.A, 8efh.1.A, 8efv.1.A, 8efv.1.F, 8efy.1.A, 8efy.1.B, 8efy.1.D, 8efy.1.F, 8efy.1.K, 8efy.1.M, 8ejm.1.A, 8emc.1.A, 8emc.1.H, 8emc.1.I, 8emc.1.J, 8emc.1.K, 8emc.1.L, 8emc.1.M, 8emc.1.N, 8emh.1.A, 8emh.1.B, 8emh.1.C, 8emh.1.D, 8emh.1.E, 8emh.1.F, 8emh.1.G, 8emh.1.H, 8emh.1.I, 8emh.1.J, 8emh.1.K, 8emh.1.L, 8enk.1.A, 8enk.1.B, 8eog.1.C, 8esq.1.J, 8et3.1.A, 8et3.1.B, 8et3.1.C, 8et3.1.D, 8et3.1.E, 8et3.1.F, 8etr.1.A, 8ets.1.A, 8ets.1.D, 8ets.1.E, 8ets.1.F, 8ets.1.G, 8ets.1.H, 8ets.1.I, 8etw.1.A, 8eu9.1.A, 8eu9.1.D, 8eu9.1.E, 8eu9.1.F, 8eu9.1.G, 8eu9.1.H, 8eu9.1.I, 8euf.1.D, 8euf.1.E, 8euf.1.F, 8euf.1.G, 8euf.1.H, 8euf.1.I, 8eup.1.J, 8exw.1.A, 8f29.1.V, 8f29.1.W, 8f29.1.X, 8f29.1.Y, 8f29.1.Z, 8f2k.1.A, 8f2k.1.C, 8f2k.1.D, 8f2k.1.E, 8f39.1.T, 8f39.1.U, 8f39.1.V, 8f39.1.W, 8f39.1.X, 8f39.1.Y, 8fak.1.D, 8faz.1.B, 8faz.1.C, 8faz.1.D, 8fcl.1.A, 8fcl.1.B, 8fcl.1.C, 8fcl.1.D, 8fcl.1.E, 8fcm.1.D, 8fcm.1.E, 8fcm.1.G, 8fcn.1.L, 8fco.1.D, 8fco.1.E, 8fco.1.G, 8fcp.1.A, 8fcp.1.C, 8fcp.1.F, 8fcq.1.B, 8fcq.1.C, 8fcr.1.D, 8fcr.1.E, 8fct.1.A, 8fct.1.B, 8fct.1.D, 8fct.1.F, 8fcu.1.Q, 8fcv.1.E, 8fcv.1.F, 8fcv.1.G, 8fcv.1.I, 8fcv.1.J, 8fcw.1.D, 8fd6.1.A, 8fdt.1.A, 8fkj.1.B, 8fkj.1.C, 8fkj.1.D, 8fkj.1.E, 8fkj.1.F, 8fkj.1.G, 8fks.1.j, 8fkt.1.0, 8fl8.1.D, 8fl8.1.E, 8fl8.1.F, 8fl8.1.G, 8fl8.1.H, 8fl8.1.I, 8flj.1.M, 8flj.1.N, 8fs3.1.A, 8fs3.1.C, 8fs3.1.E, 8fs4.1.A, 8fs4.1.C, 8fs5.1.A, 8fs5.1.E, 8fs6.1.A, 8fs6.1.B, 8fs6.1.E, 8fs7.1.A, 8fs7.1.B, 8fs7.1.E, 8fs8.1.B, 8fs8.1.C, 8fs8.1.D, 8fs8.1.E, 8fth.1.A, 8ftk.1.A, 8ftm.1.A, 8ftm.2.A, 8fwi.1.C, 8fwj.1.A, 8g09.1.J, 8g0z.1.A, 8g0z.1.B, 8g0z.1.C, 8g0z.1.D, 8g0z.1.E, 8g0z.1.F, 8g4f.1.B, 8g7t.1.A, 8g7t.1.C, 8g7u.1.A, 8g7u.1.C, 8g7v.1.A, 8g7v.1.C, 8g9u.1.A, 8gbj.1.B, 8gbj.1.C, 8gbj.1.D, 8giy.1.B, 8giy.1.C, 8giy.1.D, 8giy.1.E, 8giz.1.B, 8giz.1.C, 8giz.1.D, 8giz.1.E, 8gj0.1.B, 8gj0.1.C, 8gj0.1.D, 8gj0.1.E, 8gj1.1.B, 8gj1.1.C, 8gj1.1.D, 8gj1.1.E, 8gj2.1.B, 8gj2.1.C, 8gj2.1.D, 8gj2.1.E, 8gj3.1.B, 8gj3.1.C, 8gj3.1.D, 8gj3.1.E, 8gj8.1.A, 8gja.1.A, 8gja.1.B, 8gja.2.B, 8gja.3.A, 8gja.3.B, 8gju.1.B, 8gju.2.B, 8gju.2.C, 8glu.1.A, 8glu.1.C, 8glu.1.D, 8glw.1.A, 8glw.1.B, 8glw.1.C, 8glw.1.E, 8glw.1.F, 8glw.1.G, 8glw.1.H, 8glx.1.A, 8glx.1.E, 8glx.1.F, 8glx.1.G, 8glx.1.I, 8glx.1.J, 8gme.1.B, 8gme.2.B, 8gms.1.C, 8gms.1.D, 8gmu.1.B, 8gw1.1.G, 8gw1.1.H, 8gwb.1.E, 8gwb.1.F, 8gwg.1.E, 8gwg.1.F, 8gwi.1.G, 8gwi.1.H, 8gwk.1.G, 8gwk.1.H, 8gwm.1.E, 8gwm.1.F, 8gwn.1.G, 8gwn.1.H, 8gwo.1.G, 8gwo.1.H, 8gyk.1.A, 8gyk.1.B, 8gyk.1.C, 8gyk.1.E, 8gyk.1.F, 8gyk.1.H, 8gzq.1.B, 8gzr.1.B, 8h3h.1.A, 8h3h.1.B, 8h3h.1.C, 8h3h.1.D, 8h3h.1.E, 8h3h.1.F, 8h5y.2.A, 8h5z.1.A, 8h5z.2.A, 8h6j.1.5, 8h6j.1.M, 8h6k.3.A, 8h9i.1.B, 8h9i.1.C, 8h9i.1.D, 8h9i.1.E, 8h9i.1.H, 8h9p.1.A, 8h9p.1.C, 8h9p.1.F, 8h9s.1.D, 8h9s.1.E, 8h9s.1.F, 8he5.1.O, 8hf0.1.A, 8hf0.1.D, 8hf1.1.A, 8hf1.1.D, 8hf1.1.F, 8hh1.1.A, 8hh2.1.A, 8hh2.1.B, 8hh2.1.C, 8hh3.1.A, 8hh3.1.B, 8hh3.1.C, 8hh3.1.D, 8hh3.1.E, 8hh3.1.F, 8hh4.1.A, 8hh4.1.B, 8hh4.1.C, 8hh4.1.D, 8hh4.1.E, 8hh5.1.A, 8hh5.1.B, 8hh5.1.C, 8hh6.1.B, 8hh6.1.E, 8hh7.1.A, 8hh7.1.C, 8hh8.1.B, 8hh8.1.C, 8hh8.1.D, 8hh9.1.A, 8hh9.1.B, 8hh9.1.C, 8hh9.1.D, 8hh9.1.E, 8hh9.1.F, 8hhb.1.E, 8hhc.1.A, 8hhc.1.D, 8hl7.1.B, 8hmz.1.G, 8hrz.1.A, 8hrz.1.L, 8huj.1.A, 8i0r.1.R, 8i0s.1.V, 8i0t.1.P, 8i0v.1.V, 8i0w.1.0, 8i0w.1.D, 8i1m.1.A, 8i1n.1.A, 8i9j.1.B, 8i9p.1.F, 8i9r.1.F, 8i9t.1.G, 8i9t.1.Y, 8i9v.1.0, 8i9v.1.G, 8i9w.1.F, 8i9w.1.Z, 8i9x.1.0, 8i9y.1.G, 8i9z.1.G, 8i9z.1.Y, 8igd.1.A, 8igd.2.A, 8igu.1.D, 8igu.1.E, 8igu.1.F, 8igv.1.D, 8igv.1.E, 8igv.1.F, 8igw.1.D, 8igw.1.E, 8igw.1.F, 8igw.2.D, 8igw.2.E, 8igw.2.F, 8iju.1.A, 8iud.1.A, 8izn.1.A, 8j07.4.A, 8j07.844.A, 8j07.848.A, 8j07.849.A, 8j07.877.A, 8j0s.1.B, 8j0s.1.C, 8j0s.1.D, 8j0s.1.E, 8j0s.1.F, 8j4t.1.G, 8j4u.1.M, 8j4u.1.O, 8j4u.1.P, 8j4u.1.Q, 8j4u.1.R, 8j90.1.K, 8jix.1.A, 8jne.1.K, 8jon.1.A, 8jqb.1.E, 8jqc.1.E, 8jri.1.A, 8jri.1.B, 8jri.1.C, 8jri.1.D, 8jri.1.X, 8jri.1.Y, 8jrt.1.A, 8jrt.1.B, 8jrt.1.C, 8jrt.1.D, 8jrt.1.S, 8jrt.1.T, 8jti.1.A, 8jti.1.B, 8jti.1.C, 8jti.1.D, 8jti.1.E, 8jti.1.F, 8juw.1.A, 8juw.1.E, 8juw.1.F, 8juy.1.A, 8juy.1.B, 8juy.1.C, 8juy.1.D, 8juy.1.E, 8juy.1.F, 8juz.1.A, 8juz.1.B, 8juz.1.C, 8juz.1.D, 8juz.1.E, 8juz.1.F, 8jx6.1.A, 8jx6.2.A, 8k0g.1.A, 8k0g.1.B, 8k0g.1.C, 8k0g.1.D, 8k0g.1.E, 8k0g.1.U, 8k24.1.A, 8k2a.73.A, 8k3y.1.A, 8k3y.1.B, 8k3y.1.C, 8k3y.1.D, 8k3y.1.E, 8k3y.1.F, 8kca.1.A, 8kca.2.A, 8kcb.1.K, 8kcc.1.K, 8kfp.1.A, 8kg2.1.A, 8odu.1.A, 8odu.1.B, 8odv.1.A, 8of8.1.J, 8ofb.1.A, 8ohm.1.A, 8oip.1.Y, 8oiq.1.q, 8ojl.1.A, 8ojl.1.B, 8ojl.1.C, 8ojl.1.D, 8ojl.1.E, 8ojl.1.F, 8oka.1.A, 8oka.1.B, 8oka.1.C, 8oka.1.D, 8oka.1.E, 8oka.1.F, 8om2.1.W, 8om7.1.A, 8om7.1.B, 8om7.1.C, 8om7.1.D, 8om7.1.E, 8om7.1.F, 8oo7.1.C, 8oo7.1.E, 8oo7.1.G, 8ooi.1.C, 8oop.1.A, 8oop.1.G, 8oor.1.B, 8oor.1.D, 8oor.1.F, 8ork.1.A, 8oru.1.A, 8oru.1.B, 8osf.1.A, 8osf.1.B, 8osf.1.C, 8osf.1.D, 8osf.1.E, 8osf.1.F, 8osg.1.A, 8osg.1.B, 8osg.1.C, 8osg.1.D, 8osg.1.E, 8osg.1.F, 8osh.1.A, 8osh.1.B, 8osh.1.C, 8osh.1.D, 8osh.1.E, 8ouy.1.B, 8ouy.1.C, 8ouy.1.D, 8ouz.1.B, 8ouz.1.C, 8ouz.1.D, 8ovf.1.A, 8ovf.1.B, 8ovf.1.C, 8ovf.1.D, 8ovf.1.E, 8ovf.1.F, 8ovg.1.A, 8ovg.1.B, 8ovg.1.C, 8ovg.1.D, 8ovg.1.E, 8ovg.1.F, 8oz0.1.s, 8oz0.1.t, 8p53.1.C, 8p53.1.E, 8pb9.1.A, 8pb9.1.D, 8pb9.1.E, 8pbd.1.F, 8peu.1.A, 8peu.1.B, 8peu.1.C, 8peu.1.D, 8peu.1.E, 8peu.1.F, 8peu.1.G, 8peu.1.H, 8peu.1.I, 8peu.1.J, 8peu.1.K, 8peu.1.L, 8pew.1.A, 8pew.1.B, 8pew.1.C, 8pew.1.D, 8pew.1.E, 8pew.1.F, 8pew.1.G, 8pew.1.H, 8pew.1.I, 8pew.1.J, 8pew.1.K, 8pew.1.L, 8pew.1.M, 8pew.1.N, 8pew.1.O, 8pew.1.P, 8pew.1.Q, 8pew.1.R, 8pex.1.A, 8pex.1.B, 8pex.1.C, 8pex.1.E, 8pex.1.F, 8pex.1.G, 8pex.1.H, 8pex.1.I, 8pex.1.J, 8pex.1.K, 8pex.1.L, 8pey.1.A, 8pey.1.B, 8pey.1.C, 8pey.1.E, 8pey.1.F, 8pey.1.G, 8pey.1.H, 8pey.1.J, 8pey.1.L, 8pey.1.M, 8pfl.1.A, 8pfp.1.A, 8pjb.1.A, 8pjj.1.A, 8pnk.1.A, 8po6.1.A, 8po7.1.A, 8po8.1.A, 8po8.1.B, 8pqv.1.A, 8pqx.1.A, 8pqx.1.B, 8pqz.1.J, 8ptg.1.A, 8ptg.1.D, 8ptg.1.E, 8ptg.1.F, 8ptk.1.d, 8ptk.1.e, 8ptm.1.A, 8ptm.1.C, 8ptm.1.E, 8ptm.1.F, 8ptn.1.A, 8ptn.1.B, 8ptn.1.C, 8ptn.1.D, 8ptn.1.E, 8ptn.1.F, 8pto.1.A, 8pto.1.C, 8pto.1.E, 8ptp.1.A, 8ptp.1.E, 8pts.1.A, 8pts.1.B, 8puu.1.A, 8puu.1.B, 8q0j.1.O, 8q0q.1.O, 8q3i.1.G, 8q3n.1.A, 8q3n.1.E, 8q3n.1.F, 8q3o.1.A, 8q3o.1.B, 8q3o.1.E, 8q3o.1.F, 8q3p.1.A, 8q3q.1.A, 8q3w.1.A, 8q3w.1.B, 8q46.1.O, 8q4d.1.E, 8q4d.1.F, 8q4d.1.G, 8q4d.1.H, 8q4d.1.M, 8q4d.1.N, 8q67.1.A, 8q67.1.B, 8q67.1.C, 8q67.1.D, 8q67.1.E, 8q67.1.F, 8q7w.1.F, 8q7x.1.C, 8q9t.1.A, 8qcf.1.L, 8qku.1.K, 8qku.1.N, 8qku.1.O, 8qku.1.P, 8qku.1.Q, 8qku.1.R, 8qku.1.S, 8qkv.1.L, 8qkv.1.O, 8qkv.1.P, 8qkv.1.Q, 8qkv.1.R, 8qkv.1.S, 8qkv.1.T, 8qn8.1.G, 8qqe.1.A, 8qr1.1.A, 8qr1.1.H, 8qr1.1.I, 8qr1.1.J, 8qr1.1.K, 8qr1.1.L, 8qr1.1.M, 8qsj.1.a, 8qu6.1.H, 8qy7.1.A, 8qyc.1.A, 8qyp.1.A, 8qyq.1.A, 8qyq.2.A, 8qyr.1.A, 8qyu.1.A, 8qyv.1.I, 8qyv.1.M, 8qyv.1.N, 8qyv.1.O, 8qyv.1.P, 8qyv.1.Q, 8qyv.1.R, 8qz0.1.N, 8qz0.1.O, 8qz0.1.P, 8qz0.1.Q, 8qz0.1.R, 8qz0.1.T, 8qz0.1.U, 8qzs.2.A, 8r08.10.A, 8r0a.1.M, 8r0b.1.B, 8r0e.1.A, 8r64.1.A, 8r64.1.D, 8r64.1.E, 8r64.1.F, 8r7k.1.D, 8r7l.1.A, 8r9r.1.A, 8r9v.1.A, 8ran.1.A, 8rap.1.O, 8rbf.1.A, 8rbg.1.A, 8rc0.1.E, 8rcf.1.H, 8rd3.1.A, 8rdu.1.K, 8rdu.1.L, 8rdu.1.M, 8rdu.1.U, 8rdu.1.W, 8rdu.1.X, 8rdy.1.A, 8rev.1.A, 8rfj.1.K, 8rhn.1.A, 8rhn.1.B, 8rhn.1.C, 8rhn.1.D, 8rhn.1.E, 8rhn.1.F, 8ri2.1.A, 8rix.1.A, 8rl5.1.C, 8rl5.1.D, 8rl6.1.A, 8rl9.1.D, 8rla.1.B, 8rm5.1.9, 8rm5.1.k, 8ro0.1.E, 8ro1.1.G, 8ro1.1.U, 8ro2.1.E, 8ro2.1.L, 8ro6.1.A, 8roj.1.A, 8rs9.1.A, 8rsb.1.A, 8rsb.1.B, 8rsb.1.C, 8rsc.1.A, 8rsc.1.B, 8rsc.1.F, 8rtb.1.C, 8rtd.1.5, 8ruy.1.M, 8rwv.1.F, 8rwv.1.I, 8rwv.1.J, 8rwv.1.K, 8rxd.1.A, 8rxd.1.B, 8rxd.1.C, 8rxd.1.D, 8rxd.1.E, 8rxd.1.F, 8rxk.1.A, 8rxk.1.B, 8rxk.1.C, 8rxt.1.A, 8rxt.1.B, 8rxt.1.C, 8rxt.1.D, 8rxt.1.E, 8rxt.1.F, 8s0c.1.F, 8s0d.1.N, 8s0e.1.K, 8s0e.1.O, 8s0f.1.L, 8s0f.1.M, 8s35.1.L, 8s36.1.L, 8s37.1.L, 8s70.1.A, 8s7g.1.C, 8s7g.1.D, 8s7g.1.E, 8s7g.1.F, 8s7g.1.G, 8s7g.1.H, 8s7g.1.I, 8s7g.1.K, 8s7g.1.M, 8s7g.1.N, 8s9i.1.A, 8scz.1.A, 8sd0.1.A, 8sgd.1.A, 8skz.1.A, 8sm3.1.B, 8so8.1.A, 8sp4.1.A, 8spg.1.A, 8spv.1.A, 8spv.1.D, 8spw.1.B, 8spw.1.C, 8spw.1.E, 8spw.1.F, 8spx.1.A, 8spx.1.B, 8spx.1.C, 8spx.1.D, 8spx.1.E, 8spx.1.F, 8ssw.1.A, 8su9.1.M, 8su9.1.N, 8su9.1.P, 8su9.1.Q, 8su9.1.R, 8sub.1.N, 8sub.1.O, 8sub.1.P, 8suw.1.M, 8suw.1.N, 8suw.1.O, 8suw.1.P, 8syf.1.A, 8syf.1.B, 8szp.1.A, 8szp.2.A, 8szq.1.A, 8szr.1.A, 8t0l.1.F, 8t14.1.E, 8t5s.1.A, 8t5u.1.A, 8tbn.1.A, 8tbx.1.A, 8tby.1.D, 8th8.1.L, 8thb.1.A, 8thb.1.B, 8thb.1.C, 8thb.1.D, 8thc.1.A, 8thc.1.B, 8thc.1.C, 8thc.1.D, 8thc.1.E, 8thd.1.A, 8thd.1.E, 8ti0.1.D, 8tid.1.L, 8tjy.1.H, 8tpl.1.A, 8tvy.1.M, 8tw7.1.A, 8tw7.1.B, 8tw7.1.C, 8tw7.1.D, 8tw8.1.A, 8tw8.1.B, 8tw8.1.C, 8twa.1.J, 8twb.1.D, 8u0u.1.A, 8u0v.1.A, 8u0v.1.B, 8u0v.1.C, 8u0v.1.D, 8u0v.1.E, 8u0v.1.F, 8u0w.1.A, 8u1h.1.A, 8u1h.1.B, 8u1h.1.C, 8u1h.1.D, 8u1h.1.E, 8u1h.1.F, 8u3k.1.E, 8u7i.1.G, 8u7i.1.H, 8u7t.1.A, 8u7t.1.B, 8u8e.1.D, 8u8i.1.D, 8u8i.1.E, 8u9c.1.A, 8u9c.1.C, 8u9c.1.D, 8u9c.1.E, 8u9c.1.F, 8u9p.1.A, 8u9p.1.B, 8u9p.1.C, 8u9p.1.D, 8u9q.1.A, 8u9q.1.B, 8u9q.1.C, 8u9q.1.D, 8u9q.1.E, 8u9q.1.F, 8u9z.1.A, 8u9z.1.B, 8u9z.1.C, 8u9z.1.D, 8u9z.1.E, 8u9z.1.F, 8ua0.1.A, 8ua0.1.B, 8ua0.1.C, 8ua0.1.D, 8ua0.1.E, 8ua0.1.F, 8ua1.1.A, 8ua1.1.B, 8ua1.1.C, 8ua1.1.D, 8ua1.1.E, 8ua1.1.F, 8uaa.1.A, 8uaa.1.B, 8uaa.1.C, 8uaa.1.D, 8uaa.1.F, 8uae.1.M, 8uae.1.N, 8uae.1.O, 8uae.1.P, 8uae.1.Q, 8uae.1.R, 8uaf.1.M, 8uaf.1.N, 8uaf.1.O, 8uaf.1.P, 8uaf.1.Q, 8uaf.1.R, 8ub4.1.A, 8ub4.1.E, 8uh7.1.C, 8uh7.1.D, 8uh7.1.E, 8ui7.1.B, 8ui7.1.C, 8ui7.1.D, 8ui7.1.E, 8ui8.1.C, 8ui8.1.D, 8ui8.1.E, 8ui9.1.B, 8ui9.1.C, 8ui9.1.D, 8ui9.1.E, 8uii.1.B, 8uii.1.C, 8uii.1.D, 8uii.1.E, 8uk9.1.B, 8uk9.1.C, 8uk9.1.D, 8uk9.1.E, 8uk9.2.A, 8uk9.2.B, 8uk9.2.C, 8uk9.2.D, 8umh.1.V, 8umi.1.A, 8umi.1.F, 8umt.1.A, 8umt.1.B, 8umt.1.C, 8umt.1.D, 8umt.1.E, 8umu.1.A, 8umu.1.B, 8umu.1.C, 8umu.1.D, 8umu.1.E, 8umv.1.A, 8un0.1.A, 8un0.1.B, 8un0.1.C, 8un0.1.D, 8un0.1.E, 8unf.1.F, 8unf.1.G, 8unf.1.H, 8unf.1.I, 8unh.1.A, 8unh.1.B, 8unj.1.B, 8unj.1.C, 8unj.1.D, 8unj.1.E, 8uoq.1.Z, 8usb.1.B, 8usb.1.G, 8usb.1.H, 8usb.1.I, 8usb.1.J, 8usb.1.K, 8usc.1.I, 8usc.1.J, 8usc.1.K, 8usc.1.L, 8usc.1.M, 8usc.1.N, 8uv2.1.F, 8uvo.1.E, 8uvp.1.C, 8uvq.1.F, 8uvw.1.A, 8uvw.1.B, 8uxa.9.A, 8v32.1.A, 8v32.1.B, 8v32.1.C, 8v32.1.D, 8v32.1.E, 8v32.1.F, 8v32.1.G, 8v44.1.A, 8v4y.1.K, 8v83.1.I, 8v85.1.A, 8v87.1.J, 8v9r.1.A, 8v9r.1.B, 8v9r.1.C, 8v9r.1.D, 8v9r.1.E, 8v9r.1.F, 8val.1.B, 8val.1.C, 8val.1.D, 8val.1.E, 8vam.1.B, 8vam.1.C, 8vam.1.D, 8vam.1.E, 8van.1.B, 8van.1.C, 8van.1.D, 8van.1.E, 8vap.1.B, 8vap.1.C, 8vap.1.D, 8vap.1.E, 8vaq.1.B, 8vaq.1.C, 8vaq.1.D, 8vaq.1.E, 8var.1.B, 8var.1.C, 8var.1.D, 8var.1.E, 8vas.1.B, 8vas.1.C, 8vas.1.D, 8vas.1.E, 8vat.1.B, 8vat.1.C, 8vat.1.D, 8vat.1.E, 8vcj.1.A, 8vcj.1.B, 8vcj.1.C, 8vcj.1.E, 8vcj.1.F, 8vcj.1.G, 8vcj.1.H, 8vct.1.A, 8vct.1.E, 8vct.1.F, 8vct.1.G, 8vct.1.H, 8vct.1.J, 8vku.1.D, 8vls.1.E, 8vov.1.C, 8vv2.1.A, 8vvd.1.A, 8vw9.1.A, 8vx1.1.A, 8vx9.1.B, 8vxa.1.C, 8vxc.1.B, 8vxt.1.A, 8vxy.1.D, 8w0a.1.C, 8w8d.1.A, 8w8d.1.B, 8w8d.1.D, 8w8d.1.E, 8w8d.1.F, 8wap.1.G, 8wap.1.H, 8wet.1.B, 8wet.1.D, 8wet.1.G, 8wfd.1.A, 8wfd.1.B, 8wfd.1.C, 8wfd.1.D, 8wfd.1.E, 8wfd.1.F, 8wh5.1.K, 8wh8.1.K, 8wha.1.K, 8wiv.1.C, 8wj3.1.A, 8wj3.1.B, 8wj3.1.C, 8wj3.1.D, 8wk0.1.A, 8wk0.1.B, 8wk0.1.C, 8wk0.1.D, 8wk0.1.F, 8woc.1.A, 8wof.1.E, 8wof.1.F, 8wof.1.G, 8wsm.1.A, 8wtk.1.A, 8wtl.1.A, 8wv8.1.A, 8wve.1.A, 8x15.1.I, 8x15.1.Q, 8x19.1.I, 8x19.1.M, 8x19.1.O, 8x19.1.P, 8x19.1.Q, 8x19.1.R, 8x1c.1.I, 8x1c.1.M, 8x1c.1.N, 8x1c.1.O, 8x1c.1.P, 8x1c.1.Q, 8x1c.1.R, 8x1g.1.A, 8x3v.1.A, 8x3v.1.B, 8x5n.1.B, 8x5n.1.C, 8x5n.1.D, 8x5n.1.E, 8xa8.1.F, 8xak.1.A, 8xak.2.A, 8xau.1.A, 8xau.1.C, 8xau.1.D, 8xau.1.E, 8xau.1.F, 8xav.1.B, 8xav.1.C, 8xav.1.F, 8xaw.1.A, 8xaw.1.C, 8xax.1.A, 8xax.1.B, 8xax.1.D, 8xax.1.E, 8xay.1.B, 8xay.1.C, 8xay.1.D, 8xay.1.E, 8xay.1.F, 8xbt.1.K, 8xbt.1.L, 8xbt.1.M, 8xbt.1.N, 8xbt.1.O, 8xbt.1.P, 8xbt.1.Q, 8xbt.1.R, 8xbx.1.C, 8xch.1.3, 8xch.1.M, 8xch.1.N, 8xch.1.U, 8xch.1.V, 8xi2.1.5, 8xks.1.B, 8xks.1.F, 8xks.1.G, 8xku.1.A, 8xku.1.B, 8xku.1.D, 8xku.1.E, 8xku.1.F, 8xkv.1.A, 8xkv.1.B, 8xkv.1.D, 8xkv.1.E, 8xkv.1.F, 8xms.1.A, 8xon.1.A, 8xon.1.B, 8xon.1.C, 8xon.1.D, 8xon.1.E, 8xon.1.F, 8xoo.1.O, 8xoo.1.Q, 8xoo.1.R, 8xoo.1.S, 8xoo.1.T, 8xqw.1.B, 8xqw.1.C, 8xqw.1.E, 8xqw.1.F, 8xqx.1.B, 8xqx.1.C, 8xqx.1.E, 8xqx.1.F, 8xt1.1.a, 8xvb.1.G, 8xvd.1.G, 8xvg.1.B, 8xvg.1.C, 8xvg.1.D, 8xvg.1.E, 8xvg.1.I, 8xvt.1.E, 8xvt.1.F, 8xxn.1.a, 8y6o.1.E, 8y6o.1.I, 8y7w.1.A, 8y9y.1.A, 8y9z.1.A, 8ya0.1.A, 8ya2.1.A, 8ycx.1.A, 8ycx.1.B, 8ycx.1.C, 8ycx.1.D, 8ycx.1.E, 8ycx.1.F, 8yd0.1.A, 8yd0.1.B, 8yd0.1.C, 8yd0.1.D, 8yd0.1.E, 8yd0.1.F, 8yd1.1.A, 8yd1.1.B, 8yd1.1.C, 8yd1.1.D, 8yd1.1.E, 8yd1.1.F, 8yep.1.A, 8yes.1.A, 8ygv.1.D, 8ygv.1.F, 8yh8.1.D, 8yh8.1.E, 8yh8.1.F, 8yhx.1.A, 8yhx.1.B, 8yhx.1.C, 8yhx.1.D, 8yhx.1.E, 8yhx.1.F, 8yig.1.B, 8yig.1.C, 8yig.1.E, 8yih.1.B, 8yii.1.A, 8yk8.1.A, 8yka.1.A, 8yka.1.B, 8yka.1.E, 8ykc.1.A, 8ykc.1.C, 8yle.1.A, 8ynj.1.A, 8yrs.1.A, 8yrs.1.B, 8z49.1.A, 8zb7.1.A, 8zbk.1.A, 8zbm.1.H, 8zbn.1.K, 8zef.1.A, 8zem.1.A, 8zeo.1.C, 8zep.1.B, 8zep.1.C, 8zi0.1.B, 8zi0.1.D, 8zi0.1.E, 8zi0.1.F, 8zi1.1.B, 8zi1.1.D, 8zi1.1.E, 8zi1.1.F, 8zi1.1.G, 8zi2.1.A, 8zi2.1.D, 8zi2.1.E, 8zi2.1.F, 8zi3.1.B, 8zi3.1.C, 8zi3.1.D, 8zi3.1.G, 8ziq.1.M, 8ziq.1.N, 8ziq.1.O, 8ziq.1.P, 8ziq.1.Q, 8ziq.1.R, 8zir.1.M, 8zir.1.N, 8zir.1.O, 8zir.1.P, 8zir.1.Q, 8zir.1.R, 8zis.1.A, 8zis.1.B, 8zis.1.C, 8zis.1.D, 8zis.1.E, 8zis.1.F, 8zit.1.M, 8zit.1.N, 8zit.1.O, 8zit.1.P, 8zit.1.Q, 8zit.1.R, 8zn5.1.A, 8zn5.1.B, 8zn5.1.C, 8zn5.1.D, 8zn5.1.E, 8zn5.1.F, 8zn5.2.A, 8zn5.2.B, 8zn5.2.C, 8zn5.2.D, 8zn6.1.A, 8zn7.1.A, 8zns.1.A, 8zou.21.A, 8zp4.1.A, 8zp4.1.D, 8zp4.1.G, 8zp5.1.B, 8zp5.1.F, 8zpk.1.B, 8zpk.1.F, 8zpk.1.H, 8zrs.1.A, 8zrs.1.B, 8zrs.1.C, 8zrs.1.D, 8zrs.1.E, 8zrs.1.F, 8zrs.1.G, 8zrs.1.H, 8zrs.1.I, 8zrs.1.J, 8zrs.1.K, 8zrs.1.L, 8zue.1.B, 8zwo.1.A, 8zwo.1.B, 8zwo.1.C, 8zwo.1.D, 8zwo.1.E, 9asj.1.B, 9ask.1.A, 9asm.1.A, 9asn.1.C, 9aso.1.A, 9asp.1.A, 9avj.1.A, 9avj.1.B, 9avj.1.C, 9avj.1.D, 9avj.1.E, 9avj.1.F, 9avr.1.A, 9aym.1.B, 9ayq.1.A, 9b0e.1.A, 9b0i.1.B, 9b0x.1.I, 9b0x.1.J, 9b0x.1.K, 9b0x.1.L, 9b0x.1.N, 9b15.1.A, 9b15.2.A, 9b1d.1.A, 9b1d.1.E, 9b1d.1.G, 9b1d.1.I, 9b1d.1.J, 9b1e.1.A, 9b1e.1.F, 9b1e.1.H, 9b2d.1.A, 9b3j.1.J, 9b3j.1.M, 9b89.1.A, 9bc5.1.A, 9bc5.1.B, 9bc5.1.C, 9bc5.1.D, 9bc5.1.E, 9bc5.1.F, 9bc5.1.G, 9bcx.1.G, 9bcx.1.J, 9bcx.1.K, 9bcx.1.M, 9bf1.1.A, 9bf1.1.B, 9bf5.1.C, 9bf5.1.D, 9bgb.1.A, 9bgk.1.F, 9bgk.1.G, 9bh6.1.A, 9bh7.1.A, 9bh8.1.A, 9bh8.1.B, 9bh9.1.B, 9bho.1.C, 9bho.1.D, 9bhq.1.C, 9bhq.1.D, 9bht.1.B, 9bht.1.C, 9bht.1.D, 9bht.1.E, 9bhw.1.A, 9bhw.1.B, 9bhw.1.C, 9bhw.1.D, 9bln.1.f, 9bm4.1.A, 9bm5.1.A, 9bm6.1.A, 9bm7.1.A, 9bmb.1.A, 9bmd.1.A, 9bmf.1.A, 9bmg.1.A, 9bmn.1.A, 9bmo.1.A, 9bmp.1.A, 9bmt.1.A, 9bmw.1.A, 9bn1.1.A, 9bn3.1.A, 9bn4.1.A, 9boq.1.D, 9bpa.1.A, 9bqv.1.A, 9bu7.1.A, 9bu7.1.D, 9bu7.1.E, 9bu7.1.F, 9bu7.1.G, 9bui.1.N, 9bui.1.O, 9bui.1.P, 9bui.1.Q, 9bui.1.R, 9bui.1.S, 9bv1.1.L, 9bv1.1.M, 9bv1.1.N, 9bv1.1.O, 9bv1.1.P, 9bv1.1.Q, 9bv2.1.L, 9bv2.1.M, 9bv2.1.N, 9bv2.1.O, 9bv2.1.P, 9bv2.1.Q, 9bv3.1.M, 9bv3.1.N, 9bv3.1.O, 9bv3.1.P, 9bv3.1.R, 9bw1.1.L, 9bw1.1.M, 9bw1.1.N, 9bw1.1.Q, 9bw1.1.R, 9bw4.1.N, 9bw4.1.O, 9bw4.1.P, 9bw4.1.Q, 9bw4.1.R, 9bw4.1.S, 9bxu.1.0, 9bxu.1.1, 9bxu.1.2, 9byk.1.0, 9byk.1.2, 9byk.1.B, 9bym.1.A, 9bym.1.B, 9bym.1.R, 9bz0.1.R, 9c1m.1.G, 9c1m.1.H, 9c1m.1.I, 9c1m.1.J, 9c1m.1.K, 9c1m.1.L, 9c1n.1.I, 9c1o.1.A, 9c1o.1.B, 9c1o.1.C, 9c1o.1.D, 9c1o.1.E, 9c1o.1.F, 9c57.1.A, 9c57.1.D, 9c57.1.E, 9c57.1.F, 9c57.1.G, 9c57.1.H, 9c57.1.M, 9c5q.1.B, 9c5x.1.M, 9c5x.1.N, 9c5x.1.O, 9c5x.1.P, 9c62.1.A, 9c62.1.B, 9c62.1.C, 9c62.1.D, 9c62.1.E, 9c62.1.F, 9c62.1.L, 9c6q.1.B, 9c87.1.A, 9c87.1.B, 9c87.1.C, 9c87.1.D, 9c87.1.E, 9c87.1.F, 9c88.1.B, 9c88.1.C, 9c88.1.D, 9c88.1.E, 9c88.1.F, 9c88.1.G, 9c9g.1.G, 9c9g.1.K, 9c9g.1.M, 9c9g.1.N, 9c9g.1.O, 9c9s.1.A, 9c9t.1.K, 9c9t.1.O, 9c9t.1.Q, 9c9t.1.S, 9ca7.1.G, 9ca9.1.F, 9ca9.1.H, 9ca9.1.J, 9cab.1.A, 9cab.1.E, 9cab.1.I, 9cac.1.A, 9cac.1.E, 9cac.1.G, 9cac.1.H, 9cac.1.I, 9cae.1.E, 9cae.1.F, 9cae.1.G, 9cae.1.I, 9can.1.K, 9can.1.O, 9can.1.P, 9can.1.Q, 9can.1.R, 9can.1.S, 9cat.1.A, 9cat.1.E, 9cat.1.F, 9cat.1.G, 9cat.1.I, 9cb7.1.K, 9cc0.1.A, 9cc0.1.B, 9cc0.1.C, 9cc0.1.D, 9cc0.1.E, 9cc0.1.F, 9cc1.1.B, 9cc1.1.C, 9cc1.1.D, 9cc1.1.E, 9cc1.1.F, 9cc1.1.G, 9cc3.1.B, 9cc3.1.C, 9cc3.1.D, 9cc3.1.E, 9cc3.1.F, 9cc3.1.G, 9ccd.1.D, 9ccd.1.F, 9cfu.1.D, 9cfv.1.D, 9cfw.1.D, 9cgc.1.5, 9cgc.1.6, 9cgc.1.7, 9cgc.1.O, 9cgc.1.P, 9cgc.1.Q, 9cm0.1.A, 9cn3.1.a, 9cpa.1.o, 9crx.1.A, 9crx.1.B, 9crx.1.C, 9crx.1.D, 9crx.1.E, 9crx.1.F, 9d46.1.G, 9d4n.1.C, 9d5j.1.B, 9d5k.1.B, 9d8a.1.A, 9dci.1.A, 9dci.1.B, 9des.1.A, 9des.1.C, 9dfs.1.D, 9dfv.1.D, 9dgp.1.A, 9dgr.1.X, 9dgr.1.Y, 9dgy.1.A, 9dh5.1.A, 9dh6.1.A, 9dh7.1.A, 9dh8.1.A, 9dh9.1.A, 9dha.1.A, 9di3.1.A, 9dil.1.A, 9dil.1.B, 9diu.1.A, 9dj7.1.A, 9dju.1.A, 9djz.1.A, 9dk0.1.A, 9dkd.1.A, 9dke.1.A, 9dkj.1.A, 9dkx.1.A, 9dle.1.A, 9dlp.1.D, 9dls.1.A, 9dls.1.B, 9dls.1.C, 9dls.1.D, 9dls.1.E, 9dls.1.F, 9dlv.1.D, 9dn5.1.A, 9drz.1.A, 9dtr.1.U, 9dts.1.A, 9dts.2.A, 9dts.3.A, 9dts.4.A, 9dvy.1.A, 9dvy.1.B, 9dvy.1.C, 9dvy.1.D, 9dvy.1.E, 9dvy.1.F, 9dzy.1.A, 9dzy.1.D, 9e0k.1.A, 9e0u.1.A, 9e0w.1.A, 9e0x.1.A, 9e0y.1.A, 9e0z.1.B, 9e12.1.A, 9e12.1.B, 9e14.1.A, 9e1l.1.K, 9e1m.1.K, 9e1n.1.K, 9e1o.1.K, 9e1p.1.K, 9e1q.1.K, 9e1u.1.K, 9e1v.1.K, 9e1w.1.K, 9e1x.1.K, 9e22.1.A, 9e24.1.A, 9e24.1.B, 9e24.1.C, 9e25.1.A, 9e25.1.B, 9e25.1.F, 9e26.1.A, 9e27.1.A, 9e27.1.B, 9e27.1.C, 9e29.1.A, 9e29.1.B, 9e29.1.C, 9e29.1.F, 9e2c.1.A, 9e6l.1.K, 9e6n.1.F, 9e8g.1.A, 9e8g.1.C, 9e8g.1.E, 9e8j.1.A, 9e8j.1.B, 9e8j.1.V, 9e8j.1.W, 9e8j.1.X, 9e8j.1.Y, 9e8k.1.A, 9e8k.1.B, 9e8k.1.I, 9e8k.1.J, 9e8k.1.K, 9e8k.1.L, 9e8l.1.A, 9e8l.1.B, 9e8l.1.C, 9e8l.1.D, 9e8l.1.E, 9e8l.1.F, 9e8n.1.A, 9e8n.1.B, 9e8n.1.C, 9e8n.1.K, 9e8n.1.L, 9e8n.1.M, 9e8o.1.2, 9e8o.1.3, 9e8o.1.4, 9e8o.1.A, 9e8q.1.A, 9e8q.1.B, 9e8q.1.D, 9e8q.1.F, 9e9c.1.6, 9ear.1.K, 9eco.1.A, 9eco.1.B, 9eco.1.C, 9eco.1.D, 9eco.1.E, 9eco.1.F, 9ei1.1.P, 9ei3.1.P, 9ei4.1.P, 9el5.1.A, 9ema.1.F, 9emc.1.A, 9emc.1.E, 9eq2.1.A, 9eq2.1.B, 9eq2.1.C, 9eq2.1.D, 9eq2.1.E, 9eq2.1.F, 9erd.1.A, 9ere.1.B, 9esh.1.R, 9esh.1.c, 9evh.1.A, 9evh.1.B, 9evh.1.C, 9evh.1.D, 9evh.1.E, 9evh.1.F, 9evp.1.A, 9evp.1.B, 9evp.1.C, 9evp.1.D, 9evp.1.E, 9evp.1.F, 9evs.22.A, 9exd.1.A, 9ezx.1.A, 9ezy.1.A, 9f0j.1.A, 9f1u.1.A, 9f20.1.A, 9f2l.1.A, 9f2w.1.A, 9f3p.1.A, 9f3t.1.E, 9f3t.1.F, 9f5i.1.A, 9f5i.1.D, 9f5i.1.F, 9f6c.1.A, 9f73.1.A, 9f73.1.F, 9f74.1.A, 9f74.1.F, 9f75.1.B, 9f75.1.C, 9f75.1.D, 9f75.1.F, 9f7n.1.A, 9f7n.1.B, 9f7n.1.C, 9f7n.1.F, 9f9n.1.A, 9f9n.1.F, 9f9o.1.A, 9f9o.1.F, 9f9w.1.A, 9f9w.1.B, 9f9w.1.C, 9f9w.1.D, 9f9w.1.E, 9f9w.1.F, 9f9x.1.A, 9f9x.1.B, 9f9x.1.C, 9f9x.1.E, 9f9x.1.F, 9fa1.1.A, 9fa1.1.B, 9fa1.1.F, 9fa2.1.A, 9fa2.1.B, 9fa2.1.F, 9fb0.1.A, 9fb0.1.B, 9fb0.1.C, 9fb0.1.D, 9fb0.1.E, 9fb0.1.F, 9fb4.1.A, 9fb4.1.C, 9fb4.1.E, 9fb5.1.A, 9fb5.1.B, 9fb5.1.C, 9fb5.1.F, 9fb6.1.B, 9fb6.1.D, 9fb6.1.F, 9fb8.1.A, 9fbw.1.I, 9fbw.1.L, 9fbw.1.M, 9fbw.1.N, 9fbw.1.O, 9fbw.1.P, 9fbw.1.Q, 9fd2.1.U, 9ff7.1.A, 9ff7.1.B, 9ff7.1.C, 9ff7.1.D, 9ff7.1.E, 9ff7.1.F, 9ff7.1.G, 9ff7.1.H, 9ff7.1.L, 9fl7.1.C, 9fp0.1.C, 9g0f.1.A, 9g0f.1.B, 9g0f.1.C, 9g0f.1.D, 9g0f.1.E, 9g0f.1.F, 9g0f.1.G, 9g33.61.A, 9g33.62.A, 9g5b.1.1, 9g8n.1.H, 9g8o.1.J, 9ga3.1.E, 9ga4.1.C, 9ga4.1.F, 9gcg.1.H, 9gcs.1.A, 9gcs.1.B, 9gcs.1.C, 9gcs.1.D, 9gcs.1.E, 9gcs.1.F, 9gcs.1.G, 9gcs.1.H, 9gcs.1.S, 9gcs.1.T, 9gcs.1.U, 9gcs.1.V, 9gct.1.A, 9gct.1.B, 9gct.1.C, 9gct.1.D, 9gct.1.E, 9gct.1.F, 9gct.1.G, 9gct.1.H, 9gct.1.I, 9gct.1.J, 9gct.1.L, 9gct.1.M, 9gct.1.N, 9gct.1.O, 9gct.1.P, 9gcu.1.A, 9gcu.1.B, 9gcu.1.C, 9gcu.1.E, 9gcu.1.F, 9gd1.1.Q, 9gd2.1.U, 9gd3.1.L, 9ge5.1.A, 9ge5.1.B, 9ge5.1.C, 9ge5.1.D, 9ge5.1.E, 9ge5.1.F, 9ge5.1.G, 9gev.1.A, 9gev.1.N, 9gev.1.O, 9gev.1.R, 9gfb.1.D, 9gfb.1.E, 9gfb.1.F, 9gfb.1.G, 9gi1.1.P, 9gi1.1.Q, 9gi1.1.S, 9gi1.1.T, 9gi1.1.U, 9gjp.1.K, 9gjw.1.H, 9gjw.1.K, 9gm5.1.H, 9gm5.1.L, 9gmw.1.A, 9gmw.1.B, 9gs2.1.A, 9gs2.1.B, 9gs2.1.C, 9gs2.1.D, 9gs2.1.E, 9gs2.1.F, 9gs2.1.G, 9gsn.1.A, 9gsn.1.C, 9gsn.1.E, 9gsn.1.F, 9gu9.1.A, 9gy7.1.E, 9h4a.1.A, 9h4a.1.B, 9hb4.1.A, 9hb4.1.B, 9hb4.1.C, 9hb4.1.D, 9hb4.1.E, 9hb4.1.F, 9hcc.1.4, 9hcc.1.o, 9hcf.1.p, 9hcg.1.7, 9hch.1.7, 9hg4.1.A, 9hgi.1.A, 9hgi.1.B, 9hgj.1.A, 9hgj.1.B, 9hlz.46.A, 9hm0.30.A, 9hm0.44.A, 9hpo.1.A, 9hpo.1.B, 9hpo.1.C, 9hpo.1.D, 9hpo.1.E, 9hpo.1.F, 9hpo.1.G, 9hpo.1.H, 9hpo.1.I, 9hpo.1.J, 9hpo.1.K, 9hpo.1.L, 9hqv.22.A, 9htf.1.A, 9htg.1.A, 9i0s.1.A, 9i0s.1.B, 9i16.1.A, 9i16.1.B, 9i16.2.A, 9i16.2.B, 9i16.3.A, 9i16.3.B, 9i1p.1.A, 9i1p.1.B, 9i1p.2.A, 9i1p.2.B, 9i22.1.A, 9i22.2.A, 9i23.2.A, 9i4v.1.A, 9i62.1.I, 9i8p.1.A, 9if4.1.A, 9if4.1.B, 9if4.1.C, 9if4.1.D, 9if4.1.E, 9if4.1.F, 9ihl.1.A, 9ii8.1.A, 9iin.1.A, 9iin.1.D, 9ikz.1.E, 9ikz.1.F, 9io5.1.A, 9io5.1.B, 9io5.1.C, 9io5.1.D, 9io5.1.E, 9io5.1.F, 9io5.1.I, 9io5.1.J, 9io5.1.M, 9ip0.1.A, 9itj.1.A, 9itj.1.B, 9itj.1.C, 9itj.1.D, 9itj.1.E, 9itj.1.F, 9its.1.B, 9its.1.D, 9its.1.E, 9itt.1.D, 9itt.1.E, 9itu.1.D, 9itu.1.E, 9ix4.1.A, 9ix4.1.C, 9ixm.1.A, 9ixm.1.C, 9j3c.1.A, 9jao.1.A, 9jap.1.A, 9jap.1.B, 9jap.1.C, 9jap.1.D, 9jap.1.F, 9jap.1.G, 9jap.1.H, 9jc1.1.C, 9jc1.1.F, 9jc1.1.G, 9jc1.1.H, 9jix.1.A, 9jix.2.A, 9jmt.1.A, 9jn9.1.A, 9jnt.1.K, 9jnu.1.K, 9jnv.1.K, 9jnw.1.K, 9jnz.1.K, 9jo2.1.K, 9jo5.1.K, 9jr9.1.A, 9jv8.1.A, 9jvc.1.A, 9jvp.1.A, 9jvp.1.B, 9jvp.1.C, 9jvp.1.D, 9jvp.1.E, 9jvp.1.F, 9kae.1.A, 9kae.1.C, 9kae.1.E, 9kae.1.F, 9kak.1.D, 9kak.1.F, 9kbg.1.A, 9kbh.1.A, 9kbh.1.B, 9kbh.1.C, 9kbh.1.E, 9kbh.1.F, 9kbi.1.A, 9kbi.1.D, 9kbi.1.E, 9kbi.1.I, 9kbi.1.J, 9kbj.1.A, 9kbj.1.L, 9khv.1.A, 9khz.1.A, 9khz.1.B, 9ki0.1.A, 9ki0.1.B, 9kmd.1.A, 9kmd.1.B, 9kmd.1.C, 9kmd.1.D, 9kmd.1.E, 9kmd.1.F, 9kmd.1.I, 9kqh.1.A, 9ktw.1.A, 9ku4.1.A, 9kwf.1.A, 9ky7.1.A, 9ky7.1.F, 9l26.1.B, 9l26.1.D, 9l26.1.G, 9l26.1.H, 9l26.1.I, 9l3m.1.B, 9l3m.1.C, 9l3m.1.E, 9l3m.1.F, 9l3m.1.G, 9l3u.1.B, 9l3u.1.C, 9l3u.1.F, 9l3u.1.G, 9l3u.1.H, 9l5r.1.0, 9l5r.1.j, 9l5s.1.0, 9l5s.1.Y, 9l5t.1.8, 9lgo.1.A, 9lgo.1.F, 9lgo.1.G, 9lgo.1.H, 9lgo.1.I, 9lgo.1.J, 9liu.1.K, 9lj2.1.K, 9lj2.1.L, 9llk.1.B, 9llk.1.E, 9llk.1.F, 9llk.1.I, 9m2w.1.A, 9m2w.1.B, 9m2w.1.C, 9m2w.1.D, 9m2w.1.E, 9m2w.1.F, 9m3v.1.A, 9m3w.1.A, 9m3x.1.A, 9m3y.1.A, 9m3z.1.A, 9m3z.1.B, 9m3z.1.C, 9m3z.1.D, 9m3z.1.E, 9m3z.1.F, 9m4n.1.A, 9mfo.1.A, 9mfv.1.A, 9mfw.1.A, 9mfy.1.B, 9mpq.1.A, 9mpq.1.B, 9mpq.1.C, 9mpq.1.D, 9mpq.1.E, 9mpq.1.F, 9mpr.1.G, 9mpr.1.H, 9mpr.1.I, 9mps.1.B, 9mps.1.C, 9mps.1.D, 9mpt.1.B, 9mpu.1.B, 9mpv.1.D, 9mpv.1.E, 9mpv.1.F, 9mpv.1.G, 9mpv.1.H, 9mpv.1.I, 9mq6.1.A, 9mq6.1.B, 9mse.1.A, 9mse.1.F, 9msf.1.C, 9msg.1.A, 9msg.1.B, 9msg.1.D, 9msg.1.E, 9msg.1.F, 9n07.1.A, 9n11.1.A, 9n22.1.A, 9n22.1.B, 9n22.1.C, 9n22.1.D, 9n22.1.E, 9n22.1.F, 9n32.1.A, 9n32.1.B, 9n6h.1.I, 9n6k.1.I, 9n6v.29.A, 9n6z.29.A, 9n6z.30.A, 9n72.30.A, 9n72.44.A, 9n74.40.A, 9n76.38.A, 9n77.35.A, 9n79.1.W, 9ndj.1.A, 9ndj.1.B, 9ndj.1.C, 9ndj.1.D, 9ndj.1.E, 9ndj.1.F, 9ng2.1.B, 9ng2.1.C, 9ng2.1.D, 9nh8.1.L, 9njk.1.A, 9njk.1.B, 9njk.1.C, 9njk.1.D, 9njk.1.E, 9njk.1.F, 9njr.1.A, 9njr.1.B, 9njr.1.C, 9njr.1.D, 9njr.1.E, 9njr.1.F, 9nkf.1.A, 9nkf.1.B, 9nkf.1.C, 9nkf.1.D, 9nkf.1.E, 9nkf.1.i, 9nkg.1.2, 9nkg.1.3, 9nkg.1.4, 9nkg.1.5, 9nkg.1.6, 9nkg.1.7, 9nki.1.A, 9nki.1.B, 9nki.1.C, 9nki.1.D, 9nki.1.E, 9nki.1.F, 9nkj.1.A, 9nkj.1.B, 9nkj.1.C, 9nkj.1.D, 9nkj.1.E, 9nkj.1.F, 9nlu.1.B, 9nlu.1.C, 9nlu.1.D, 9nlw.1.B, 9nlw.1.C, 9nlw.1.D, 9nly.1.B, 9nly.1.C, 9nly.1.D, 9nlz.1.A, 9nlz.1.B, 9nlz.1.C, 9nlz.1.D, 9np6.1.A, 9np6.1.B, 9nud.1.A, 9nud.1.B, 9nud.1.D, 9nud.1.F, 9nue.1.A, 9nue.1.B, 9nue.1.E, 9nue.1.F, 9nv0.1.A, 9nv0.1.B, 9nv0.1.C, 9nv0.1.D, 9nv0.1.E, 9nv0.1.F, 9nv9.1.A, 9nvd.1.A, 9nvd.1.B, 9nvd.1.C, 9nvd.1.D, 9nvd.1.E, 9nvd.1.F, 9nvl.1.A, 9nvl.1.B, 9nvl.1.C, 9nvm.1.A, 9nvm.1.B, 9nvm.1.C, 9nyy.1.A, 9nyy.1.B, 9o19.1.A, 9o19.1.B, 9o19.1.C, 9oa1.1.A, 9oa1.1.B, 9oa1.1.C, 9oa1.1.D, 9oa1.1.E, 9oa1.1.F, 9oa2.1.A, 9oa2.1.B, 9oa2.1.C, 9oa2.1.D, 9oa2.1.E, 9oa2.1.F, 9ofg.1.A, 9ofh.1.A, 9ofj.1.A, 9og3.1.A, 9ojr.1.A, 9ojr.1.D, 9ojr.1.E, 9ojr.1.F, 9oju.1.C, 9oju.1.E, 9oju.1.F, 9ojz.1.D, 9ojz.1.E, 9ojz.1.F, 9ok5.1.A, 9ok5.1.B, 9ok5.1.C, 9ok5.1.D, 9ok5.1.E, 9ok5.1.F, 9okc.1.A, 9okc.1.B, 9okc.1.C, 9okc.1.D, 9okc.1.E, 9okc.1.F, 9olj.1.A, 9olj.1.B, 9olj.1.C, 9olj.1.D, 9olj.1.E, 9olj.1.F, 9olo.1.A, 9olo.1.B, 9olo.1.C, 9olo.1.D, 9olo.1.E, 9olo.1.F, 9omq.1.A, 9omq.1.B, 9omq.1.C, 9omq.1.D, 9omq.1.E, 9omq.1.F, 9op3.1.B, 9osw.1.A, 9osy.1.B, 9osy.1.C, 9ow9.1.A, 9ox9.1.A, 9p00.1.A, 9p00.1.B, 9p00.1.E, 9p00.1.F, 9p01.1.A, 9p02.1.A, 9p02.1.B, 9p02.1.C, 9p02.1.D, 9p02.1.E, 9p02.1.F, 9p07.1.A, 9p07.1.B, 9p07.1.C, 9p07.1.D, 9p07.1.E, 9p07.1.F, 9p11.1.D, 9p1d.1.A, 9p9v.1.A, 9p9v.1.B, 9p9v.1.C, 9p9v.1.D, 9p9v.1.E, 9p9v.1.F, 9paf.1.A, 9paf.1.D, 9paf.1.E, 9paf.1.F, 9pag.1.A, 9pag.1.C, 9pag.1.D, 9pag.1.E, 9pag.1.F, 9pb9.1.A, 9pb9.1.C, 9pb9.1.D, 9pb9.1.E, 9pb9.1.F, 9pba.1.A, 9pba.1.C, 9pba.1.D, 9pba.1.E, 9pba.1.F, 9pbf.1.D, 9pbf.1.E, 9pbf.1.F, 9pbf.1.G, 9pbf.1.H, 9pbf.1.I, 9pbv.1.A, 9pbv.1.B, 9pbv.1.C, 9pbv.1.D, 9pbv.1.E, 9pbv.1.F, 9pc3.1.A, 9pc3.1.C, 9pc3.1.D, 9pc3.1.E, 9pc3.1.F, 9pcx.1.A, 9pcx.1.B, 9pcx.1.C, 9pcx.1.D, 9pcx.1.E, 9pcx.1.F, 9pcz.1.A, 9pcz.1.B, 9pcz.1.C, 9pcz.1.D, 9pcz.1.E, 9pcz.1.F, 9pd1.1.A, 9pd1.1.B, 9pd1.1.C, 9pd1.1.D, 9pd1.1.E, 9pd1.1.F, 9pd8.1.A, 9pd8.1.B, 9pd8.1.C, 9pd8.1.D, 9pd8.1.E, 9pd8.1.F, 9pdb.1.A, 9pdb.1.B, 9pdb.1.C, 9pdb.1.D, 9pdb.1.E, 9pdb.1.F, 9pdd.1.A, 9pdd.1.B, 9pdd.1.C, 9pdd.1.D, 9pdd.1.E, 9pdd.1.F, 9pdi.1.A, 9pdi.1.C, 9pdi.1.L, 9pdi.1.M, 9pdi.1.N, 9pdl.1.A, 9pdl.1.K, 9pdl.1.L, 9pdl.1.M, 9pdl.1.N, 9pdn.1.B, 9pdn.1.K, 9pdn.1.L, 9pdn.1.M, 9pdn.1.N, 9peo.1.B, 9pes.1.B, 9pes.1.C, 9pes.1.E, 9pet.1.B, 9pet.1.C, 9peu.1.B, 9peu.1.C, 9peu.1.D, 9pev.1.B, 9pev.1.C, 9pf1.1.A, 9pf1.1.B, 9pf1.1.K, 9pf1.1.L, 9pf1.1.M, 9pf1.1.N, 9pf2.1.A, 9pf2.1.B, 9pf2.1.C, 9pf2.1.D, 9pf2.1.E, 9pf2.1.F, 9pfc.1.A, 9pfc.1.B, 9pfc.1.C, 9pfc.1.D, 9pfc.1.E, 9pfc.1.F, 9pff.1.I, 9pff.1.K, 9pff.1.L, 9pff.1.M, 9pff.1.N, 9pio.1.A, 9pio.1.B, 9pio.1.C, 9pio.1.D, 9pio.1.E, 9pio.1.F, 9pjd.1.A, 9pjd.1.B, 9pjd.1.C, 9pjd.1.D, 9pjd.1.E, 9pjd.1.F, 9q91.1.A, 9q91.1.B, 9q91.1.C, 9q91.1.K, 9q92.1.B, 9q92.1.C, 9q92.1.D, 9q92.1.E, 9q92.1.F, 9q92.1.G, 9q93.1.A, 9q93.1.B, 9q93.1.C, 9q93.1.D, 9q93.1.E, 9q93.1.F, 9q97.1.G, 9q97.1.H, 9q97.1.I, 9q97.1.J, 9q97.1.K, 9q97.1.L, 9q98.1.A, 9q98.1.B, 9q98.1.C, 9q98.1.D, 9q98.1.E, 9q98.1.F, 9q9z.1.A, 9qcl.1.C, 9qcl.1.F, 9qcl.1.G, 9qcl.1.K, 9qcl.1.L, 9qcl.1.M, 9qdq.1.A, 9qec.1.A, 9qed.1.B, 9qee.1.F, 9qn8.1.C, 9qn8.1.G, 9qn8.1.K, 9qna.1.F, 9qnb.1.B, 9qnb.1.D, 9qnb.1.E, 9qnb.1.F, 9qnc.1.A, 9qnc.1.K, 9qq6.1.C, 9qqr.1.A, 9qqr.1.D, 9qqr.1.E, 9qqr.1.G, 9qqr.1.H, 9qqr.1.I, 9qqr.1.J, 9qrw.1.A, 9qrw.1.G, 9qrw.1.L, 9qwn.1.A, 9r34.1.A, 9r34.1.B, 9r34.1.C, 9r34.1.D, 9r34.1.E, 9r34.1.F, 9rai.1.H, 9rai.1.M, 9rti.1.A, 9rur.1.A, 9rur.2.A, 9rus.1.A, 9rus.2.A, 9sfg.1.A, 9svx.1.B, 9svx.1.C, 9svx.1.D, 9svx.1.E, 9svx.1.F, 9svx.1.J, 9svy.1.A, 9svy.1.D, 9svy.1.E, 9svy.1.F, 9svy.1.G, 9svy.1.M, 9sw0.1.A, 9sw0.1.G, 9sw0.1.H, 9sw0.1.I, 9sw0.1.J, 9sw0.1.K, 9sw0.1.M, 9sw0.1.N, 9t4v.1.K, 9t99.1.A, 9uf8.1.B, 9uf8.1.f, 9uf8.1.h, 9uf8.1.i, 9uf8.1.j, 9uf8.1.k, 9ug9.1.F, 9ug9.1.G, 9ug9.1.K, 9ug9.1.L, 9ug9.1.O, 9ug9.1.R, 9uht.1.E, 9uht.1.F, 9ui4.1.B, 9ui4.1.C, 9ui4.1.D, 9ui4.1.E, 9ui4.1.F, 9ui4.1.G, 9ui4.1.H, 9ui4.1.I, 9ui4.1.J, 9ui5.1.A, 9ui5.1.B, 9ui5.1.C, 9ui5.1.D, 9ui5.1.E, 9ui5.1.H, 9ui5.1.I, 9ui5.1.J, 9ui5.1.K, 9ui7.1.A, 9ui7.1.B, 9ui7.1.C, 9ui7.1.D, 9ui7.1.H, 9ui7.1.I, 9ui7.1.J, 9ui8.1.A, 9ui8.1.B, 9ui8.1.F, 9ui8.1.G, 9ui8.1.H, 9ui8.1.I, 9ui8.1.J, 9ui8.1.K, 9ui8.1.L, 9ui9.1.A, 9ui9.1.B, 9ui9.1.C, 9ui9.1.F, 9ui9.1.G, 9ui9.1.I, 9ui9.1.J, 9ui9.1.K, 9ui9.1.L, 9uie.1.B, 9uie.1.C, 9vio.1.A, 9vio.1.B, 9vio.1.C, 9vit.1.A, 9y6s.1002.A, 9y6s.869.A, 9y6s.887.A, 9y6s.903.A, 9y6s.935.A, 9y6s.939.A, 9y6s.946.A, 9y6s.948.A, 9y6s.968.A, 9y6s.969.A, 9y6s.991.A, 9y6s.994.A, 9yhn.1.A, 9ykj.1.A, 9ykj.1.B, K9N600.1.A

Swiss Institute of Bioinformatics
Contact Us
