# Supplementary material for: Molecular Ruler Variation in Insect Dicer-2 Suggests a Structural Basis for Species-Dependent siRNA Length and Antiviral Defense Diversity
Source: Viruses. 2026 Feb 27;18(3):285. doi: 10.3390/v18030285 (PMC13030778; doi:10.3390/v18030285)
Supplement: Supplementary file 1 [file viruses-18-00285-s001.zip › Locusta_report_7w0e.html]

Locusta\_dicer\_2\_7W0E | Report


Homology Modelling Report

## Model Building Report

This document lists the results for the homology modelling project "Locusta\_dicer\_2\_7W0E" submitted to SWISS-MODEL workspace
on Dec. 28, 2025, 12:39 p.m..The submitted primary amino acid sequence is given in Table T1.

If you use any results in your research, please cite the relevant publications:

- Waterhouse A, Bertoni M, Bienert S, Studer G, Tauriello G, Gumienny R, Heer FT, de Beer TAP, Rempfer C, Bordoli L, Lepore R, Schwede T

  SWISS-MODEL: homology modelling of protein structures and complexes.

  Nucleic Acids Res 46, W296-W303. (2018) 2978835510.1093/nar/gky427
- Bienert S, Waterhouse A, de Beer TAP, Tauriello G, Studer G, Bordoli L, Schwede T

  The SWISS-MODEL Repository - new features and functionality.

  Nucleic Acids Res 45, D313-D319. (2017) 2789967210.1093/nar/gkw1132
- Studer G, Tauriello G, Bienert S, Biasini M, Johner N, Schwede T

  ProMod3 - A versatile homology modelling toolbox.

  PLOS Comp Biol 17(1), e1008667. (2021) 3350798010.1371/journal.pcbi.1008667
- Studer G, Rempfer C, Waterhouse AM, Gumienny R, Haas J, Schwede T

  QMEANDisCo - distance constraints applied on model quality estimation.

  Bioinformatics 36, 1765-1771. (2020) 3169731210.1093/bioinformatics/btz828
- Bertoni M, Kiefer F, Biasini M, Bordoli L, Schwede T

  Modeling protein quaternary structure of homo- and hetero-oligomers beyond binary interactions by homology.

  Scientific Reports 7. (2017) 2887468910.1038/s41598-017-09654-8

## Results

The user uploaded a template structure to use for the modelling process.

## Models

The following model was built (see Materials and Methods "Model Building"):

| Model #01 | File | Built with | Oligo-State | Ligands | GMQE | QMEANDisCo Global |
| --- | --- | --- | --- | --- | --- | --- |
|  | PDB | ProMod3 3.6.0 | monomer | None | 0.58 | 0.56 ± 0.05 |

|  |  |  |
| --- | --- | --- |
|  |  |  |

| Template | Seq Identity | Oligo-state | QSQE | Found by | Method | Resolution | Seq Similarity | Range | Coverage | Description |
| --- | --- | --- | --- | --- | --- | --- | --- | --- | --- | --- |
| template\_upload.1.C | 27.71 | monomer | 0.00 | HHblits | Unknown | - | 0.34 | 7 - 1413 | 0.97 | Polypeptide |

  

### Excluded ligands

| Ligand Name.Number | Reason for Exclusion | Description |
| --- | --- | --- |
| ADP.4 | Binding site not conserved. | ADP |
| MG.1 | Not in contact with model. | MG |
| MG.2 | Not in contact with model. | MG |
| MG.3 | Not in contact with model. | MG |

  

```
Target                   MDTAPEKKQIAPRRYQEELLKRCLNENTILYLPTGSGKTFIAVMFIKKIMKECCV  
template_upload.1.C      ------DVEIKPRGYQLRLVDHLTKSNGIVYLPTGSGKTFVAILVLKRFSQDFDK  
  
Target                   KYGRGKKLAVFAVNQVALAQQQTDYISRHVEMNVGCYIGIMNVDYWDKETWHKEF  
template_upload.1.C      PIESGGKRALFMCNTVELARQQAMAVRRCTNFKVGFYVGEQGVDDWTRGMWSDEI  
  
Target                   EKNQVLVMTAQILCNIIVHNILDVNRICVAVFDECHAATGNHPMKQAADNILKL-  
template_upload.1.C      KKNQVLVGTAQVFLDMVTQTYVALSSLSVVIIDECHHGTGHHPFREFMRLFTIAN  
  
Target                   -HINPRLLGLSGSLINGDCKVSRVVRCLKDLEDTFKCKIATAEET-LLPEVRRYS  
template_upload.1.C      QTKLPRVVGLTGVLIKGNE-ITNVATKLKELEITYRGNIITVSDTKEMENVMLYA  
  
Target                   TNPEEEIHHYEGPVSD-TFTENIQRLLTQCQNVIMASKVTTSILEKTKVPFGLIP  
template_upload.1.C      TKPTEVMVSFPHQEQVLTVTRLISAEIEKFYVSLDLMNIGVQPIRR----SKSLQ  
  
Target                   MSDDMKQNKELKNIVENVKYQIDDLGLYGGYVATKMYIAALEKLARRTDTVDALD  
template_upload.1.C      CLRDPSKKSFVKQLFNDFLYQMKEYGIYAASIAIISLIVEFDIKRRQAETLSVKL  
  
Target                   LITVVKENLTEVRNGMEGQMMV--L------RNPHEQIRRFSSNKFMGFVSLIEE  
template_upload.1.C      MHRTALTLCEKIRHLLVQKLQDMTYDDDDDNVNTEEVIMNFSTPKVQRFLMSLKV  
  
Target                   IFRGVHKTDQSELGNNNVLVFVKRRASARTLSALLKILSESDIRL-HTMKPDCVV  
template_upload.1.C      SFADK------DPKDICCLVFVERRYTCKCIYGLLLNYIQSTPELRNVLTPQFMV  
  
Target                   GYGAQQSNEATEMHRKTNEDAILRFRKKETNLLVATDVLEEGIDIPICNTVIMFD  
template_upload.1.C      GRNNISPDFESVLERKWQKSAIQQFRDGNANLMICSSVLEEGIDVQACNHVFILD  
  
Target                   PPNSCRSYIQSKGRARHKTSSYHIFVCKSD-TKFLEKFNMYKAVGHEISKLLRPG  
template_upload.1.C      PVKTFNMYVQSKGRARTTEAKFVLFTADKEREKTIQQIYQYRKAHNDIAEYLKDR  
  
Target                   AINVDVDMTDG--ESEDECLEWHTPFGSHAILKGPYAIQLVNMYSCKLPHDRFTH  
template_upload.1.C      VLEKTEPELYEIKGHFQDDIDPFT-NENGAVLLPNNALAILHRYCQTIPTDAFGF  
  
Target                   LAPLWYLLNR-----------DGKCICFVQLPVISVLKWTIEGEPQKNKRLAKQS  
template_upload.1.C      VIPWFHVLQEDERDRIFGVSAKGKHVISINMPVNCMLRDTIYSDPMDNVKTAKIS  
  
Target                   AAIEAVKQLHACGELDDDMIPRPSEYIIY--HEKLFPYLTKEPIE-GSSPQQGSR  
template_upload.1.C      AAFKACKVLYSLGELNERFVPKTLKERVASIADVHFEHWNKYGDSVTATVNKADK  
  
Target                   QRKQPYVKRCPLMYSDCRPQELQTTYLHVIRIEVDYPEPPVDSDDRILYEMFNEE  
template_upload.1.C      SKDRTYKTECPLEFYDALPRVGEICYAYEIFLEPQFESC---EYTEHMYLNLQTP  
  
Target                   EYFGMISSKEIPQEFSFPLYPAEGKLQVILMKTQ-KLYSLSKEEIRHCEEFHQFI  
template_upload.1.C      RNYAILLRNKLPRLAEMPLFSNQGKLHVRVANAPLEVIIQNSEQLELLHQFHGMV  
  
Target                   FSKVLKSVKPFMSVDQERRDHSYLIVPVNKNENGAMQIDWNTMISTRNVSTNENI  
template_upload.1.C      FRDILKIWHPFFVLDRRSKENSYLVVPLILGAGEQKCFDWELMTNFRRLPQSHGS  
  
Target                   K--MQ---MNGQNRKFSAFSVFVHHSSSGKHKFYFVEEGSGGSNQNIEIDG----  
template_upload.1.C      NVQQREQQPAPRPEDFEGKIVTQWYA--NYDKPMLVTKVHRELTPLSYMEKNQQD  
  
Target                   ------------VHV--NRSPTASLKEAIIISSDLNCLNQSRQLQ--KNSQKRKI  
template_upload.1.C      KTYYEFTMSKYGNRIGDVVHKDKFMIEVRDLTEQLTFYVHNRGKFNAKSKAKMKV  
  
Target                   FFNPEVCDIVPLSASLCVKVHLLPSILHKLTLMATSYEIFQQIPVSKGSV---LG  
template_upload.1.C      ILIPELCFNFNFPGDLWLKLIFLPSILNRMYFLLHAEALRKRFNTYLNLHLLPFN  
  
Target                   --------HEIS----ENGNDIQLLNSDF---SGCGLTLNDIEQALTAASCDNIF  
template_upload.1.C      GTDYMPRPLEIDYSLKGKVKPLLILQKTVSKEHITPAEQGEFLAAITASSAADVF  
  
Target                   NSETLETLGDSFLKFAVSLFLFIHYRNHHEGTLSTIKMKVVSNWHFYNVAKAKDI  
template_upload.1.C      DMERLEILGNSFLKLSATLYLASKYSDWNEGTLTEVKSKLVSNRNLLFCLIDADI  
  
Target                   GSKLQIHRFLPDETWVPPGFTVPQAVRRKFLS-----EHISPEYLKNLNLSGGKH  
template_upload.1.C      PKTLNTIQFTPRYTWLPPGISLPHNVLALWRENPEFAKIIGPHNLRDLALGDEES  
  
Target                   LLDQ----------LAGLEN---SWNLT-K-LNNSELLIGVQTVSDKALADALEA  
template_upload.1.C      LVKGNCSDINYNRFVEGCRANGQSFYAGADFSSEVNFCVGLVTIPNKVIADTLEA  
  
Target                   LTGVYLKAYGLYGATTLLNSLGVLPAKKASPHI-LFEKPPESPLLQESLPFQCLD  
template_upload.1.C      LLGVIVKNYGLQHAFKMLEYFKICRADIDKPLTQLLNLELGGKKMRANVNTTEID  
  
Target                   FHLGDTDELEDTLNYKFKDRGFLLQALTHPSWSDNRITDCYQRLEFLGDAVLDFL  
template_upload.1.C      GFLINHYYLEKNLGYTFKDRRYLLQALTHPSYPTNRITGSYQELEFIGNAILDFL  
  
Target                   VTSYIYDKCQKLSPGKITILRAALVNNGTFAAFAVRIGLQKYCKYRSSELFYNID  
template_upload.1.C      ISAYIFENNTKMNPGALTDLRSALVNNTTLACICVRHRLHFFILAENAKLSEIIS  
  
Target                   AFVKYQQENNHEIIDEVTCFGDDSDEENRILNSVPAPKVLGDLFESVAAAIYLDS  
template_upload.1.C      KFVNFQESQGHRV--------------TNMSTNVDVPKALGDVLEALIAAVYLDC  
  
Target                   GKCLKTVWSTYFKIMEKELARYCL  
template_upload.1.C      R-DLQRTWEVIFNLFEPELQEFT-
```

  


---

  

## Materials and Methods

## User Template Alignment

The user entered their own target sequence together with an uploaded a template structure file in PDB format.

## Model Building

Models are built based on the target-template alignment using ProMod3 (Studer et al.). Coordinates which are conserved between the target and the template are copied from the template to the model. Insertions and deletions are remodelled using a fragment library. Side chains are then rebuilt. Finally, the geometry of the resulting model is regularized by using a force field.

## Model Quality Estimation

The global and per-residue model quality has been assessed using the QMEAN scoring function (Studer et al.).

## Ligand Modelling

Ligands present in the template structure are transferred by homology to the model when the following criteria are met: (a) The ligands are annotated as biologically relevant in the template library, (b) the ligand is in contact with the model, (c) the ligand is not clashing with the protein, (d) the residues in contact with the ligand are conserved between the target and the template. If any of these four criteria is not satisfied, a certain ligand will not be included in the model. The model summary includes information on why and which ligand has not been included.

## Oligomeric State Conservation

The quaternary structure annotation of the template is used to model the target sequence in its oligomeric form. The method (Bertoni et al.) is based on a supervised machine learning algorithm, Support Vector Machines (SVM), which combines interface conservation, structural clustering, and other template features to provide a quaternary structure quality estimate (QSQE). The QSQE score is a number between 0 and 1, reflecting the expected accuracy of the interchain contacts for a model built based a given alignment and template. Higher numbers indicate higher reliability. This complements the GMQE score which estimates the accuracy of the tertiary structure of the resulting model.

## References

- Camacho C, Coulouris G, Avagyan V, Ma N, Papadopoulos J, Bealer K, Madden TL

  BLAST+: architecture and applications.

  BMC Bioinformatics, 10, 421-430. (2009) 2000350010.1186/1471-2105-10-421
- Steinegger M, Meier M, Mirdita M, Vöhringer H, Haunsberger SJ, Söding J

  HH-suite3 for fast remote homology detection and deep protein annotation.

  BMC Bioinformatics 20, 473. (2019) 3152111010.1186/s12859-019-3019-7

## Table T1:

Primary amino acid sequence for which templates were searched and models were built.

MDTAPEKKQIAPRRYQEELLKRCLNENTILYLPTGSGKTFIAVMFIKKIMKECCVKYGRGKKLAVFAVNQVALAQQQTDYISRHVEMNVGCYIGIMNVDY  
WDKETWHKEFEKNQVLVMTAQILCNIIVHNILDVNRICVAVFDECHAATGNHPMKQAADNILKLHINPRLLGLSGSLINGDCKVSRVVRCLKDLEDTFKC  
KIATAEETLLPEVRRYSTNPEEEIHHYEGPVSDTFTENIQRLLTQCQNVIMASKVTTSILEKTKVPFGLIPMSDDMKQNKELKNIVENVKYQIDDLGLYG  
GYVATKMYIAALEKLARRTDTVDALDLITVVKENLTEVRNGMEGQMMVLRNPHEQIRRFSSNKFMGFVSLIEEIFRGVHKTDQSELGNNNVLVFVKRRAS  
ARTLSALLKILSESDIRLHTMKPDCVVGYGAQQSNEATEMHRKTNEDAILRFRKKETNLLVATDVLEEGIDIPICNTVIMFDPPNSCRSYIQSKGRARHK  
TSSYHIFVCKSDTKFLEKFNMYKAVGHEISKLLRPGAINVDVDMTDGESEDECLEWHTPFGSHAILKGPYAIQLVNMYSCKLPHDRFTHLAPLWYLLNRD  
GKCICFVQLPVISVLKWTIEGEPQKNKRLAKQSAAIEAVKQLHACGELDDDMIPRPSEYIIYHEKLFPYLTKEPIEGSSPQQGSRQRKQPYVKRCPLMYS  
DCRPQELQTTYLHVIRIEVDYPEPPVDSDDRILYEMFNEEEYFGMISSKEIPQEFSFPLYPAEGKLQVILMKTQKLYSLSKEEIRHCEEFHQFIFSKVLK  
SVKPFMSVDQERRDHSYLIVPVNKNENGAMQIDWNTMISTRNVSTNENIKMQMNGQNRKFSAFSVFVHHSSSGKHKFYFVEEGSGGSNQNIEIDGVHVNR  
SPTASLKEAIIISSDLNCLNQSRQLQKNSQKRKIFFNPEVCDIVPLSASLCVKVHLLPSILHKLTLMATSYEIFQQIPVSKGSVLGHEISENGNDIQLLN  
SDFSGCGLTLNDIEQALTAASCDNIFNSETLETLGDSFLKFAVSLFLFIHYRNHHEGTLSTIKMKVVSNWHFYNVAKAKDIGSKLQIHRFLPDETWVPPG  
FTVPQAVRRKFLSEHISPEYLKNLNLSGGKHLLDQLAGLENSWNLTKLNNSELLIGVQTVSDKALADALEALTGVYLKAYGLYGATTLLNSLGVLPAKKA  
SPHILFEKPPESPLLQESLPFQCLDFHLGDTDELEDTLNYKFKDRGFLLQALTHPSWSDNRITDCYQRLEFLGDAVLDFLVTSYIYDKCQKLSPGKITIL  
RAALVNNGTFAAFAVRIGLQKYCKYRSSELFYNIDAFVKYQQENNHEIIDEVTCFGDDSDEENRILNSVPAPKVLGDLFESVAAAIYLDSGKCLKTVWST  
YFKIMEKELARYCL

## Table T2:

| Template | Seq Identity | Oligo-state | QSQE | Found by | Method | Resolution | Seq Similarity | Coverage | Description |
| --- | --- | --- | --- | --- | --- | --- | --- | --- | --- |
| template\_upload.1.C | 27.71 | monomer | - | HHblits | Unknown | NA | 0.34 | 0.97 | Polypeptide |
| template\_upload.1.C | 29.80 | monomer | - | BLAST | Unknown | NA | 0.36 | 0.57 | Polypeptide |
| template\_upload.1.C | 37.12 | monomer | - | BLAST | Unknown | NA | 0.38 | 0.33 | Polypeptide |
| template\_upload.1.C | 26.25 | monomer | - | HHblits | Unknown | NA | 0.33 | 0.06 | Polypeptide |
| template\_upload.1.C | 20.25 | monomer | - | HHblits | Unknown | NA | 0.30 | 0.06 | Polypeptide |

  
The table above shows the top 5 filtered templates. A further 1 template was found which was considered to be less suitable for modelling than the filtered list.  
template\_upload.1.C

Swiss Institute of Bioinformatics
Contact Us
