# Supplementary material for: Molecular Ruler Variation in Insect Dicer-2 Suggests a Structural Basis for Species-Dependent siRNA Length and Antiviral Defense Diversity
Source: Viruses. 2026 Feb 27;18(3):285. doi: 10.3390/v18030285 (PMC13030778; doi:10.3390/v18030285)
Supplement: Supplementary file 1 [file viruses-18-00285-s001.zip › R1-Alignments.pdf]

Alignments:

In these files we provide the results of homology modeling submitted to SWISS-MODEL workspace during the Dicer-2 molecular ruler project, in which the Dicer-2 from *Tribolium castaneum*, *Bombus terrestris*, *Locusta migratoria* and *Trichoplusia ni* were modeled. This file contains 8 documents with .html extension. Four files represent the reports of sequences modeled using *Drosophila melanogaster* Dicer-2 crystal structure (PDB: 7V6C) and Four files represent the reports of sequences modeled using *Drosophila melanogaster* Dicer-2 crystal structure (PDB: 7w0e).
