# Supplementary material for: Molecular Ruler Variation in Insect Dicer-2 Suggests a Structural Basis for Species-Dependent siRNA Length and Antiviral Defense Diversity
Source: Viruses. 2026 Feb 27;18(3):285. doi: 10.3390/v18030285 (PMC13030778; doi:10.3390/v18030285)
Supplement: Supplementary file 1 [file viruses-18-00285-s001.zip › Thricoplusia_report_7V6C.html]

Trichoplusia\_dicer2 | Report


Homology Modelling Report

## Model Building Report

This document lists the results for the homology modelling project "Trichoplusia\_dicer2" submitted to SWISS-MODEL workspace
on Aug. 22, 2024, 2:21 p.m..The submitted primary amino acid sequence is given in Table T1.

If you use any results in your research, please cite the relevant publications:

- Waterhouse A, Bertoni M, Bienert S, Studer G, Tauriello G, Gumienny R, Heer FT, de Beer TAP, Rempfer C, Bordoli L, Lepore R, Schwede T

  SWISS-MODEL: homology modelling of protein structures and complexes.

  Nucleic Acids Res 46, W296-W303. (2018) 2978835510.1093/nar/gky427
- Bienert S, Waterhouse A, de Beer TAP, Tauriello G, Studer G, Bordoli L, Schwede T

  The SWISS-MODEL Repository - new features and functionality.

  Nucleic Acids Res 45, D313-D319. (2017) 2789967210.1093/nar/gkw1132
- Studer G, Tauriello G, Bienert S, Biasini M, Johner N, Schwede T

  ProMod3 - A versatile homology modelling toolbox.

  PLOS Comp Biol 17(1), e1008667. (2021) 3350798010.1371/journal.pcbi.1008667
- Studer G, Rempfer C, Waterhouse AM, Gumienny R, Haas J, Schwede T

  QMEANDisCo - distance constraints applied on model quality estimation.

  Bioinformatics 36, 1765-1771. (2020) 3169731210.1093/bioinformatics/btz828
- Bertoni M, Kiefer F, Biasini M, Bordoli L, Schwede T

  Modeling protein quaternary structure of homo- and hetero-oligomers beyond binary interactions by homology.

  Scientific Reports 7. (2017) 2887468910.1038/s41598-017-09654-8

## Results

The SWISS-MODEL template library (SMTL version 2024-08-21, PDB release 2024-08-16) was searched with
BLAST (Camacho et al.) and HHblits (Steinegger et al.)
for evolutionary related structures matching the target sequence in Table T1. For details on the template search, see Materials and Methods. Overall 2349 templates were found (Table T2).

## Models

The following model was built (see Materials and Methods "Model Building"):

| Model #01 | File | Built with | Oligo-State | Ligands | GMQE | QMEANDisCo Global |
| --- | --- | --- | --- | --- | --- | --- |
|  | PDB | ProMod3 3.4.1 | monomer | None | 0.37 | 0.54 ± 0.05 |

|  |  |  |
| --- | --- | --- |
|  |  |  |

| Template | Seq Identity | Oligo-state | QSQE | Found by | Method | Resolution | Seq Similarity | Range | Coverage | Description |
| --- | --- | --- | --- | --- | --- | --- | --- | --- | --- | --- |
| 7v6c.1.A | 28.51 | monomer | 0.00 | HHblits | EM | 3.30Å | 0.35 | 461 - 2083 | 0.75 | Dicer-2, isoform A |

  

### The template contained no ligands.

  

```
Target    MKIGVTFKSKDVEKTIRCMSRGKSPGHDGLSIEHLRYAGAHMSRVLSMLYNICVGHSYLPSDMMRTVVVPIVKNKTGDLA  
7v6c.1.A  --------------------------------------------------------------------------------  
  
Target    DKDNYRPISLATVISKVLDSMLNTQLNKYLCLYDNQFGFRAGLSTESAILGLKHAVKYYTQRATSVYACFLDLSRAFDMV  
7v6c.1.A  --------------------------------------------------------------------------------  
  
Target    SYDYLWKKLQNIKMPTEIVNIFKYWYGNQINNVRWAGALSLPYRLECGVRQGGLSSPTLFNLYVNELIGELSGTRVGCFI  
7v6c.1.A  --------------------------------------------------------------------------------  
  
Target    DGVCVNNISYADDMVLLSASICGLRKLVSLCEEYAKSHGLVYNCKKSEIMVFETRGRTHDNIPPLILNGTALRRVFRFKY  
7v6c.1.A  --------------------------------------------------------------------------------  
  
Target    LGHVLTPSLKDDEDIERERRALSVRANMIARRFARCSLKVKLTLFRAYCTNFYTCSLWAGYTQRTYNALRVQYNNAFRVL  
7v6c.1.A  --------------------------------------------------------------------------------  
  
Target    VGLPRFCSASGMFADAQVDCFYATMRKRCASLVSRVRASSNSILNMIASRLDCVYLGRCCAISHGLLWRTQIKNVIEKLC  
7v6c.1.A  ------------------------------------------------------------FMCNTVELARQQAMAVRRCT  
  
Target    AVSGVGAYSSENGVDYWDKAKWDAELEKNQVIVMTSQILNDMLTHQYIRIEDINLLIFDECHHAVEDHPMRVIMKHFEGC  
7v6c.1.A  NF-KVGFYVGEQGVDDWTRGMWSDEIKKNQVLVGTAQVFLDMVTQTYVALSSLSVVIIDECHHGTGHHPFREFMRLFTIA  
  
Target    PKHSQPRVLGLTATLLNANVKSRKVEDTLHDLEITFHATIATVDELG---KVLNYSTNPNEMVQFYRSSPPSA-VTNEVI  
7v6c.1.A  NQTKLPRVVGLTGVLIKGN-EITNVATKLKELEITYRGNIITVSDTKELENVMLYATKPTEVMVSFPHQEQVLTVTRLIS  
  
Target    KLLSVRQELIASVKLPRSSTKQTITLKQHQEDISNNPKKIVKAVKNMISSMILFLNELGMYGGSLGILAYIILLERLRRR  
7v6c.1.A  AEI---EKFYVSLDLMNI-GVQPIRRSKSLQCLR--DPSKKSFVKQLFNDFLYQMKEYGIYAASIAIISLIVEFDIKRRQ  
  
Target    ASSKEEDILYQNVITCCIDARARLLKAMSDV---------HGYERIIKYSSEKVLLTLNILKEYNPAYQDTPGVLLKVNR  
7v6c.1.A  AETLSVKLMHRTALTLCEKIRHLLVQKLQDMTYDDDDDNVNTEEVIMNFSTPKVQRFLMSLKVSFAD------------K  
  
Target    SRKPLSAIIFTKQRFTAKVLYNLLKDVRDSNPAEFDFLKHDFVVGFNVNPLKSTREEYYIKKTGQQALLKFGNNDLNCLI  
7v6c.1.A  DPKDICCLVFVERRYTCKCIYGLLLNYIQSTPELRNVLTPQFMVGRNNISP--DFESVLERKWQKSAIQQFRDGNANLMI  
  
Target    STSVIEEGIDIPQCLLVLRYDQPLEYRSYIQSKGRARSSESSYVILVNREDEKKFMTLYKEFQETEQLIQRILVGNTDDR  
7v6c.1.A  CSSVLEEGIDVQACNHVFILDPVKTFNMYVQSKGRARTTEAKFVLFTADKEREKTIQQIYQYRKAHNDIAEYLKDRVLEK  
  
Target    DEPAQENIDKNLYQDEDVPPFISPYGGRLTATSAISLLNRYCSMLPHDHFTIITPMWIKETVTN---------KHGFDCN  
7v6c.1.A  TEPELYEIKG-HF-QDDIDPFTNENGAVLLPNNALAILHRYCQTIPTDAFGFVIPWFHVLQEDERDRIFGVSAKGKHV--  
  
Target    LVTIVLPIACPIKEEIKGMPMYNLKSAKRSAALNACVKLYEAGELDPLTMLPMRYTAVDFDDADVQSCFLNWRNDDMKRV  
7v6c.1.A  -ISINMPVNCMLRDTIYSDPMDNVKTAKISAAFKACKVLYSLGELNER-FVPKTLKERVASIAD--VHFEHWNKYGDS--  
  
Target    DDPDYPAPGTKGRVRKHRIQFPAV-LDSVPD-ESEYYLHIIKTTTAFAEPKDTREKALYDLLHRPEGFGFMTQKPLPAIC  
7v6c.1.A  -VTATVNKADKSKDRTYKTECPLEFYDALPRVGEICYAYEIFLEPQFESC--EYTEHMYLNLQTPRNYAILLRNKLPRLA  
  
Target    DFPMFMTVGEVSTSLDVNY-AVIKLDAKLLQLVKQFHFFIFEQVLAIAKKFIVFEG--KVNCLYVVPVKEDN--GYNIDW  
7v6c.1.A  EMPLFSNQGKLHVRVANAPLEVIIQNSEQLELLHQFHGMVFRDILKIWHPFFVLDRRSKENSYLVVPLILGAGEQKCFDW  
  
Target    DVMATHDQIQPVTPPPYEDR-INLKVTPENYKDCVVTPWYRVLPDRYIVSRVLEFMTPQSHFDSD-SYVTFADYYADKYK  
7v6c.1.A  ELMTNFRRLPQSHGSNVQQREQQPAPRPEDFEGKIVTQWYANYDKPMLVTKVHRELTPLSYMEKNQQDKTYYEFTMSKYG  
  
Target    LEI--IGDKSQALLEVRNISSRMNCLLPRAATINSFTDKQKKLVSASQGDDKTNRGFAEVFVAEFCIKYDFPGVLWYKAI  
7v6c.1.A  NRIGDVVHKDKFMIEVRDLTEQLTFYVHNRGKFN----------------AKSKAKMKVILIPELCFNFNFPGDLWLKLI  
  
Target    MLPSIVHRVFMLLVAHELLTEISESTKYGNPKR-RKGEEWRPVSSNMQIATLSLLAQVEEPTPITSVDRINNPTDDENPR  
7v6c.1.A  FLPSILNRMYFLLHAEALRKRFNTYLNLHLLPFNGTDYMPRPLEIDYSLKRNVDPLG----NVIPTE-DIEEPKSLLEPM  
  
Target    RPNIMSIKQSLYQLQQKKLSKDYPWDEKMEPIDIERNLSTVTVMDIECYDEFVSSPLVPIM--S--------PTRVLSPP  
7v6c.1.A  PTK--SIEASVAN--LEITEFENPWQKYMEPVDLSRNLLSTYPVELDYYYHFSVGNVCEMNEMDFEDKEYWAKNQFHMPT  
  
Target    RVVVG--------SKISAAISAPPAK--YNDKLNILKM---TATGNGPELRDILTALTTIKSHDTFNLERVETLGDAFLK  
7v6c.1.A  GNIYGNRTPAKTNANVPALMPSKPTVRGKVKPLLILQKTVSKEHITPAEQGEFLAAITASSAADVFDMERLEILGDSFLK  
  
Target    FAASLYLFHKFPKFNEGQLTNIKGRLISNRNLYYAGERFNLAGRMKVEQFSPRKDFMVPGYFAPPEVEKFIAEK-----K  
7v6c.1.A  LSATLYLASKYSDWNEGTLTEVKSKLVSNRNLLFCLIDADIPKTLNTIQFTPRYTWLPPGISLPHNVLALWRENPEFAKI  
  
Target    LRPTFLIGVYFPSSEA-FDGNLSKESMAMVRDRFADCDGTAETEPECRVQNAMQLYIHSQAVADKSVADCVEALIGTYLL  
7v6c.1.A  IGPHNLRDLALGDEESLVKGNCSDINYNRFVEGCRA-NGQS-FYAGADFSSEVNFCVGLVTIPNKVIADTLEALLGVIVK  
  
Target    SGGVLGAIKVIEWMRIIPPQDN--FATYLHTRVS-TVLSEKRATESDINFLLSHCRPDVEKILNYKFKDPSHLLEALSHP  
7v6c.1.A  NYGLQHAFKMLEYFKICRADIDKPLTQLLNLELGGKKMRA-NVNTTEIDGFL-INHYYLEKNLGYTFKDRRYLLQALTHP  
  
Target    SYIRNRLTRSYERYEFLGDAILDFLITSHVFENCGDLKPGEMTDLRSALVNNVTFASYVVKLGLHKFLCSELNPTLDKAV  
7v6c.1.A  SYPTNRITGSYQELEFIGDAILDFLISAYIFENNTKMNPGALTDLRSALVNNTTLACICVRHRLHFFILAE-NAKLSEII  
  
Target    ITFVDHQVQREHQIVEDVLYLI-------------------------------------DEEECHIAEYVEVPKVLSDIF  
7v6c.1.A  SKFVNFQESQGHRVTNYVRILLEEADVQPTPLDLDDELDMTELPHANKCISQEAEKGVPPKGEFNMSTNVDVPKALGDVL  
  
Target    EALVGAIFLDSGGDLQTVWALVYRIMCKEIHAFSSRIPQQPVKVLYEKIHACPVFDKSVVIDPDIPKIKVGVTITKNDWQ  
7v6c.1.A  EALIAAVYLDCR-DLQRTWEVIFNLFEPELQEFTRKVPINHIRQLVEHKHAKPVFSSPIVEGE-TVMVSCQFTCME--KT  
  
Target    HTVYGVGKNKSQAKRAAAKMALKVLGI  
7v6c.1.A  IKVYGFGSNKDQAKLSAAKHALQQLS-
```

  


---

  

## Materials and Methods

## Template Search

Template search with BLAST and HHblits
has been performed against the SWISS-MODEL template library (SMTL, last update: 2024-08-21, last included PDB release: 2024-08-16).

The target sequence was searched with BLAST against the primary amino acid sequence contained in the SMTL.
A total of 214 templates were found.

An initial HHblits profile has been built using the procedure outlined in (Steinegger et al.), followed by 1 iteration of HHblits against Uniclust30 (Mirdita, von den Driesch et al.). The obtained profile has then be searched against all profiles of the SMTL. A total of 2310 templates were found.

## Model Building

Models are built based on the target-template alignment using ProMod3 (Studer et al.). Coordinates which are conserved between the target and the template are copied from the template to the model. Insertions and deletions are remodelled using a fragment library. Side chains are then rebuilt. Finally, the geometry of the resulting model is regularized by using a force field.

## Model Quality Estimation

The global and per-residue model quality has been assessed using the QMEAN scoring function (Studer et al.).

## Ligand Modelling

Ligands present in the template structure are transferred by homology to the model when the following criteria are met: (a) The ligands are annotated as biologically relevant in the template library, (b) the ligand is in contact with the model, (c) the ligand is not clashing with the protein, (d) the residues in contact with the ligand are conserved between the target and the template. If any of these four criteria is not satisfied, a certain ligand will not be included in the model. The model summary includes information on why and which ligand has not been included.

## Oligomeric State Conservation

The quaternary structure annotation of the template is used to model the target sequence in its oligomeric form. The method (Bertoni et al.) is based on a supervised machine learning algorithm, Support Vector Machines (SVM), which combines interface conservation, structural clustering, and other template features to provide a quaternary structure quality estimate (QSQE). The QSQE score is a number between 0 and 1, reflecting the expected accuracy of the interchain contacts for a model built based a given alignment and template. Higher numbers indicate higher reliability. This complements the GMQE score which estimates the accuracy of the tertiary structure of the resulting model.

## References

- Camacho C, Coulouris G, Avagyan V, Ma N, Papadopoulos J, Bealer K, Madden TL

  BLAST+: architecture and applications.

  BMC Bioinformatics, 10, 421-430. (2009) 2000350010.1186/1471-2105-10-421
- Steinegger M, Meier M, Mirdita M, Vöhringer H, Haunsberger SJ, Söding J

  HH-suite3 for fast remote homology detection and deep protein annotation.

  BMC Bioinformatics 20, 473. (2019) 3152111010.1186/s12859-019-3019-7
- Mirdita M, von den Driesch L, Galiez C, Martin MJ, Söding J, Steinegger M

  Uniclust databases of clustered and deeply annotated protein sequences and alignments.

  Nucleic Acids Res, 45, D170–D176. (2016) 2789957410.1093/nar/gkw1081

## Table T1:

Primary amino acid sequence for which templates were searched and models were built.

MKIGVTFKSKDVEKTIRCMSRGKSPGHDGLSIEHLRYAGAHMSRVLSMLYNICVGHSYLPSDMMRTVVVPIVKNKTGDLADKDNYRPISLATVISKVLDS  
MLNTQLNKYLCLYDNQFGFRAGLSTESAILGLKHAVKYYTQRATSVYACFLDLSRAFDMVSYDYLWKKLQNIKMPTEIVNIFKYWYGNQINNVRWAGALS  
LPYRLECGVRQGGLSSPTLFNLYVNELIGELSGTRVGCFIDGVCVNNISYADDMVLLSASICGLRKLVSLCEEYAKSHGLVYNCKKSEIMVFETRGRTHD  
NIPPLILNGTALRRVFRFKYLGHVLTPSLKDDEDIERERRALSVRANMIARRFARCSLKVKLTLFRAYCTNFYTCSLWAGYTQRTYNALRVQYNNAFRVL  
VGLPRFCSASGMFADAQVDCFYATMRKRCASLVSRVRASSNSILNMIASRLDCVYLGRCCAISHGLLWRTQIKNVIEKLCAVSGVGAYSSENGVDYWDKA  
KWDAELEKNQVIVMTSQILNDMLTHQYIRIEDINLLIFDECHHAVEDHPMRVIMKHFEGCPKHSQPRVLGLTATLLNANVKSRKVEDTLHDLEITFHATI  
ATVDELGKVLNYSTNPNEMVQFYRSSPPSAVTNEVIKLLSVRQELIASVKLPRSSTKQTITLKQHQEDISNNPKKIVKAVKNMISSMILFLNELGMYGGS  
LGILAYIILLERLRRRASSKEEDILYQNVITCCIDARARLLKAMSDVHGYERIIKYSSEKVLLTLNILKEYNPAYQDTPGVLLKVNRSRKPLSAIIFTKQ  
RFTAKVLYNLLKDVRDSNPAEFDFLKHDFVVGFNVNPLKSTREEYYIKKTGQQALLKFGNNDLNCLISTSVIEEGIDIPQCLLVLRYDQPLEYRSYIQSK  
GRARSSESSYVILVNREDEKKFMTLYKEFQETEQLIQRILVGNTDDRDEPAQENIDKNLYQDEDVPPFISPYGGRLTATSAISLLNRYCSMLPHDHFTII  
TPMWIKETVTNKHGFDCNLVTIVLPIACPIKEEIKGMPMYNLKSAKRSAALNACVKLYEAGELDPLTMLPMRYTAVDFDDADVQSCFLNWRNDDMKRVDD  
PDYPAPGTKGRVRKHRIQFPAVLDSVPDESEYYLHIIKTTTAFAEPKDTREKALYDLLHRPEGFGFMTQKPLPAICDFPMFMTVGEVSTSLDVNYAVIKL  
DAKLLQLVKQFHFFIFEQVLAIAKKFIVFEGKVNCLYVVPVKEDNGYNIDWDVMATHDQIQPVTPPPYEDRINLKVTPENYKDCVVTPWYRVLPDRYIVS  
RVLEFMTPQSHFDSDSYVTFADYYADKYKLEIIGDKSQALLEVRNISSRMNCLLPRAATINSFTDKQKKLVSASQGDDKTNRGFAEVFVAEFCIKYDFPG  
VLWYKAIMLPSIVHRVFMLLVAHELLTEISESTKYGNPKRRKGEEWRPVSSNMQIATLSLLAQVEEPTPITSVDRINNPTDDENPRRPNIMSIKQSLYQL  
QQKKLSKDYPWDEKMEPIDIERNLSTVTVMDIECYDEFVSSPLVPIMSPTRVLSPPRVVVGSKISAAISAPPAKYNDKLNILKMTATGNGPELRDILTAL  
TTIKSHDTFNLERVETLGDAFLKFAASLYLFHKFPKFNEGQLTNIKGRLISNRNLYYAGERFNLAGRMKVEQFSPRKDFMVPGYFAPPEVEKFIAEKKLR  
PTFLIGVYFPSSEAFDGNLSKESMAMVRDRFADCDGTAETEPECRVQNAMQLYIHSQAVADKSVADCVEALIGTYLLSGGVLGAIKVIEWMRIIPPQDNF  
ATYLHTRVSTVLSEKRATESDINFLLSHCRPDVEKILNYKFKDPSHLLEALSHPSYIRNRLTRSYERYEFLGDAILDFLITSHVFENCGDLKPGEMTDLR  
SALVNNVTFASYVVKLGLHKFLCSELNPTLDKAVITFVDHQVQREHQIVEDVLYLIDEEECHIAEYVEVPKVLSDIFEALVGAIFLDSGGDLQTVWALVY  
RIMCKEIHAFSSRIPQQPVKVLYEKIHACPVFDKSVVIDPDIPKIKVGVTITKNDWQHTVYGVGKNKSQAKRAAAKMALKVLGI

## Table T2:

| Template | Seq Identity | Oligo-state | QSQE | Found by | Method | Resolution | Seq Similarity | Coverage | Description |
| --- | --- | --- | --- | --- | --- | --- | --- | --- | --- |
| A0A7E5WVU7.1.A | 100.00 | monomer | - | AFDB search | AlphaFold v2 | NA | 0.62 | 0.56 | Endoribonuclease Dcr-1-like |
| 7v6c.1.A | 28.51 | monomer | - | HHblits | EM | 3.30Å | 0.35 | 0.75 | Dicer-2, isoform A |
| 8hf0.1.A | 28.33 | homo-dimer | 0.20 | HHblits | EM | NA | 0.35 | 0.75 | Dicer-2, isoform A |
| 8dg7.1.A | 29.24 | monomer | - | HHblits | EM | NA | 0.34 | 0.76 | Endoribonuclease Dcr-1 |
| 8hf0.1.D | 28.33 | homo-dimer | 0.20 | HHblits | EM | NA | 0.35 | 0.75 | Dicer-2, isoform A |
| 8dg5.1.A | 29.24 | monomer | - | HHblits | EM | NA | 0.34 | 0.76 | Endoribonuclease Dcr-1 |
| 7v6b.1.A | 28.44 | monomer | - | HHblits | EM | 3.30Å | 0.35 | 0.75 | Dicer-2, isoform A |
| 8dga.1.A | 29.24 | monomer | - | HHblits | EM | NA | 0.34 | 0.76 | Endoribonuclease Dcr-1 |
| 7xw3.1.A | 29.87 | monomer | - | HHblits | EM | NA | 0.35 | 0.75 | Endoribonuclease Dicer |
| 8dfv.1.A | 29.24 | monomer | - | HHblits | EM | NA | 0.34 | 0.76 | Endoribonuclease Dcr-1 |
| 8dgi.1.A | 29.24 | monomer | - | HHblits | EM | NA | 0.34 | 0.76 | Endoribonuclease Dcr-1 |
| 8dgj.1.A | 29.24 | monomer | - | HHblits | EM | NA | 0.34 | 0.76 | Endoribonuclease Dcr-1 |
| 7w0f.1.A | 28.51 | monomer | - | HHblits | EM | NA | 0.35 | 0.75 | Dicer-2, isoform A |
| 7w0a.1.A | 28.33 | homo-dimer | 0.04 | HHblits | EM | NA | 0.35 | 0.75 | Dicer-2, isoform A |
| 7zpj.1.A | 30.04 | monomer | - | HHblits | EM | NA | 0.34 | 0.75 | Endoribonuclease Dicer |
| 7yz4.1.A | 30.04 | monomer | - | HHblits | EM | NA | 0.34 | 0.75 | Endoribonuclease Dicer |
| 7yym.1.A | 30.04 | monomer | - | HHblits | EM | NA | 0.34 | 0.75 | Endoribonuclease Dicer |
| 5zam.1.A | 29.87 | monomer | - | HHblits | EM | NA | 0.35 | 0.75 | Endoribonuclease Dicer |
| 5zak.1.A | 29.87 | monomer | - | HHblits | EM | NA | 0.35 | 0.75 | Endoribonuclease Dicer |
| 8hf1.1.F | 31.83 | homo-trimer | 0.06 | BLAST | EM | NA | 0.37 | 0.73 | Dicer-2, isoform A |
| 5zal.1.A | 29.87 | monomer | - | HHblits | EM | NA | 0.35 | 0.75 | Endoribonuclease Dicer |
| 7v6b.1.A | 32.03 | monomer | - | BLAST | EM | 3.30Å | 0.37 | 0.73 | Dicer-2, isoform A |
| 7v6c.1.A | 32.03 | monomer | - | BLAST | EM | 3.30Å | 0.37 | 0.73 | Dicer-2, isoform A |
| 8hf1.1.D | 31.83 | homo-trimer | 0.06 | BLAST | EM | NA | 0.37 | 0.73 | Dicer-2, isoform A |
| 8hf0.1.D | 31.83 | homo-dimer | 0.11 | BLAST | EM | NA | 0.37 | 0.73 | Dicer-2, isoform A |
| 8hf1.1.A | 31.83 | homo-trimer | 0.06 | BLAST | EM | NA | 0.37 | 0.73 | Dicer-2, isoform A |
| 7w0f.1.A | 31.97 | monomer | - | BLAST | EM | NA | 0.37 | 0.73 | Dicer-2, isoform A |
| 7w0a.1.A | 31.83 | homo-dimer | 0.04 | BLAST | EM | NA | 0.37 | 0.73 | Dicer-2, isoform A |
| 7zpi.1.A | 30.04 | monomer | - | HHblits | EM | NA | 0.34 | 0.75 | Endoribonuclease Dicer |
| 7xw2.1.A | 29.87 | monomer | - | HHblits | EM | NA | 0.35 | 0.75 | Endoribonuclease Dicer |
| 7yyn.1.B | 29.33 | monomer | - | HHblits | EM | NA | 0.34 | 0.68 | Isoform 2 of Endoribonuclease Dicer |
| 7ele.1.A | 27.36 | monomer | - | HHblits | EM | NA | 0.34 | 0.64 | Endoribonuclease Dicer homolog 1 |
| 7eld.1.A | 27.36 | monomer | - | HHblits | EM | NA | 0.34 | 0.64 | Endoribonuclease Dicer homolog 1 |
| 6bua.1.A | 28.31 | monomer | - | HHblits | EM | NA | 0.35 | 0.75 | Dicer-2, isoform A |
| 7vg3.1.A | 24.34 | monomer | - | HHblits | EM | NA | 0.32 | 0.60 | Dicer-like 3 |
| 7vg2.1.A | 24.34 | monomer | - | HHblits | EM | NA | 0.32 | 0.60 | Dicer-like 3 |
| 7xw3.1.A | 32.19 | monomer | - | BLAST | EM | NA | 0.37 | 0.43 | Endoribonuclease Dicer |
| 5zam.1.A | 32.19 | monomer | - | BLAST | EM | NA | 0.37 | 0.43 | Endoribonuclease Dicer |
| 5zak.1.A | 32.19 | monomer | - | BLAST | EM | NA | 0.37 | 0.43 | Endoribonuclease Dicer |
| 5zal.1.A | 32.19 | monomer | - | BLAST | EM | NA | 0.37 | 0.43 | Endoribonuclease Dicer |
| 6bu9.1.A | 28.31 | monomer | - | HHblits | EM | NA | 0.35 | 0.75 | Dicer-2, isoform A |
| 7xw2.1.A | 32.19 | monomer | - | BLAST | EM | NA | 0.37 | 0.43 | Endoribonuclease Dicer |
| 7zpj.1.A | 33.66 | monomer | - | BLAST | EM | NA | 0.37 | 0.29 | Endoribonuclease Dicer |
| 7yym.1.A | 33.66 | monomer | - | BLAST | EM | NA | 0.37 | 0.29 | Endoribonuclease Dicer |
| 7yz4.1.A | 33.66 | monomer | - | BLAST | EM | NA | 0.37 | 0.29 | Endoribonuclease Dicer |
| 7yyn.1.B | 33.66 | monomer | - | BLAST | EM | NA | 0.37 | 0.29 | Isoform 2 of Endoribonuclease Dicer |
| 7zpi.1.A | 33.66 | monomer | - | BLAST | EM | NA | 0.37 | 0.29 | Endoribonuclease Dicer |
| 8dfv.1.A | 41.34 | monomer | - | BLAST | EM | NA | 0.40 | 0.23 | Endoribonuclease Dcr-1 |
| 2qvw.3.A | 17.70 | monomer | - | HHblits | X-ray | 3.00Å | 0.28 | 0.31 | GLP\_546\_48378\_50642 |
| 2qvw.1.A | 17.70 | monomer | - | HHblits | X-ray | 3.00Å | 0.28 | 0.31 | GLP\_546\_48378\_50642 |

  
The table above shows the top 50 filtered templates. A further 1,650 templates were found which were considered to be less suitable for modelling than the filtered list.  
1a1v.1.A, 1c4o.1.A, 1cu1.1.A, 1cu1.1.B, 1d0e.1.C, 1d0e.1.D, 1d2m.1.A, 1d9x.1.A, 1d9z.1.A, 1di2.1.C, 1di2.1.D, 1eet.1.A, 1ekz.1.B, 1fko.1.A, 1fkp.1.A, 1fuk.1.A, 1fuu.1.B, 1gku.1.A, 1gl9.1.A, 1gm5.1.A, 1hei.1.A, 1hei.1.B, 1hv8.1.A, 1hv8.1.B, 1i4s.1.A, 1i4s.1.B, 1i6j.1.B, 1jfz.1.A, 1jfz.1.B, 1jfz.2.A, 1jfz.2.B, 1jr6.1.A, 1m6n.1.A, 1mml.1.B, 1mu2.1.A, 1mu2.1.B, 1nb4.1.A, 1nb4.2.A, 1nkt.1.A, 1nl3.1.B, 1nnd.1.A, 1o0w.1.A, 1onb.1.A, 1os5.1.A, 1oyw.1.A, 1q0u.1.A, 1q0u.2.A, 1qai.1.A, 1qai.1.B, 1qde.1.A, 1qu6.1.A, 1qva.1.A, 1rc7.1.E, 1rif.1.A, 1rif.2.A, 1rtd.1.C, 1s2m.1.A, 1stu.1.A, 1t4l.1.B, 1t4n.1.A, 1t4o.1.A, 1t4o.2.A, 1t5i.1.A, 1t5l.1.A, 1t5l.2.A, 1t6n.1.A, 1t6n.1.B, 1tf2.1.A, 1tp7.2.A, 1tp7.4.A, 1u61.1.A, 1uhz.1.A, 1uil.1.A, 1vec.1.A, 1vec.2.A, 1whn.1.A, 1whq.1.A, 1wne.1.A, 1wp9.1.A, 1wp9.2.A, 1wrb.1.A, 1wrb.2.A, 1x47.1.A, 1x48.1.A, 1x49.1.A, 1xr6.1.A, 1xr7.1.A, 1xti.1.A, 1xtj.1.A, 1xtk.1.A, 1yuy.1.A, 1yvf.2.A, 1yvx.1.A, 1yyk.1.E, 1yyk.1.F, 1yyo.1.E, 1yyo.1.F, 1yyw.1.E, 1yyw.1.F, 1yyw.2.E, 1yyw.2.F, 1yz9.1.E, 1yz9.1.F, 1z3i.1.A, 1z5z.1.A, 1z5z.2.A, 1z63.1.C, 1z6a.3.A, 1ztd.1.A, 1ztd.1.B, 2a11.1.A, 2b7t.1.A, 2b7v.1.A, 2bhr.1.A, 2bmf.1.A, 2bmf.2.A, 2ckw.1.A, 2cpn.1.A, 2d7d.1.A, 2d7s.1.A, 2db2.1.A, 2db3.1.B, 2dix.1.A, 2dmy.1.A, 2eb1.1.A, 2eb1.1.B, 2eyq.1.A, 2eyq.2.A, 2ez6.1.C, 2f55.1.B, 2f55.1.C, 2f55.2.A, 2fdc.1.B, 2fdc.2.B, 2fsh.1.A, 2fsh.1.B, 2fvp.1.B, 2fvr.1.D, 2fwr.1.A, 2fwr.2.A, 2fwr.3.A, 2fwr.4.A, 2fz4.1.A, 2fzl.1.A, 2g9n.1.A, 2g9n.2.A, 2gsl.1.A, 2gsl.2.A, 2gsl.2.B, 2gsl.3.A, 2gsl.3.B, 2gxq.1.A, 2gxs.1.A, 2gxs.1.B, 2hjv.1.A, 2hjv.2.A, 2hnd.1.A, 2hny.1.A, 2hxy.1.A, 2hxy.3.A, 2hyi.1.C, 2i4i.1.A, 2ibm.3.C, 2ibm.3.D, 2ipc.1.A, 2ipc.1.B, 2j0q.1.A, 2j0u.1.A, 2j0u.2.A, 2jgn.1.A, 2jgn.2.A, 2jlq.1.A, 2jlr.1.A, 2jls.1.A, 2jlu.1.A, 2jlv.2.A, 2jly.1.A, 2kbe.1.A, 2kbf.1.A, 2khx.1.A, 2kou.1.A, 2l2k.1.A, 2l2m.1.A, 2l2n.1.A, 2l33.1.A, 2l3c.1.A, 2l3j.1.A, 2l5c.1.A, 2l5d.1.A, 2lbs.1.B, 2ljh.1.A, 2lrs.1.A, 2ltr.1.A, 2lts.1.A, 2lup.1.B, 2luq.1.A, 2mdr.1.A, 2n3f.1.A, 2n3g.1.A, 2n3h.1.A, 2na2.1.A, 2nue.1.B, 2nue.1.C, 2nuf.1.C, 2nuf.1.D, 2nug.1.E, 2oca.1.A, 2opp.1.A, 2opq.1.A, 2opr.1.A, 2oxc.1.A, 2p6n.1.A, 2p6r.1.C, 2p6u.1.A, 2pl3.1.A, 2qeq.1.A, 2qeq.2.A, 2qvw.1.A, 2qvw.3.A, 2r2s.1.B, 2r2u.1.A, 2rb4.1.A, 2rs6.1.A, 2rs7.1.A, 2uut.1.A, 2v1x.1.A, 2v6i.1.A, 2v6j.1.A, 2v8o.1.A, 2va8.1.A, 2va8.2.A, 2vbc.1.A, 2vda.1.A, 2vl7.1.A, 2vsf.1.A, 2vso.1.A, 2w00.2.A, 2wax.1.A, 2wax.2.A, 2way.1.A, 2wcx.1.A, 2whx.1.A, 2wv9.1.A, 2wwy.1.B, 2wzq.1.A, 2xau.1.A, 2xb2.2.A, 2xfm.1.A, 2xgj.1.A, 2xgj.2.A, 2xhu.1.A, 2xhv.1.A, 2xhw.1.A, 2xwh.1.A, 2xxd.1.A, 2xym.1.A, 2yjt.1.D, 2ykg.1.A, 2ykm.1.A, 2ykn.1.A, 2yt4.1.A, 2z0m.1.A, 2z83.1.A, 2zj5.2.B, 2zj8.2.A, 2zjo.1.A, 2zu6.1.A, 2zu6.1.C, 2zu6.2.A, 2zu6.2.C, 3adg.1.A, 3adi.1.A, 3adi.2.A, 3adi.3.A, 3adj.1.A, 3adl.1.A, 3b6e.1.A, 3ber.1.A, 3bor.1.A, 3bxz.1.A, 3c4b.1.A, 3c4t.1.A, 3cdu.1.A, 3crv.1.A, 3crw.1.A, 3cso.2.A, 3din.1.A, 3dkp.1.A, 3dl8.1.A, 3du5.1.A, 3du5.2.A, 3du6.2.A, 3eaq.1.A, 3eaq.1.B, 3ear.1.A, 3eas.1.A, 3eas.1.B, 3eiq.1.A, 3eiq.1.C, 3ews.1.A, 3ews.2.A, 3ex7.2.C, 3fe2.1.A, 3fhc.1.B, 3fho.1.A, 3fht.1.A, 3fmo.1.B, 3frz.1.A, 3g0h.1.A, 3gfp.1.A, 3gnw.1.A, 3h1t.1.A, 3hgq.1.A, 3hvo.2.A, 3i32.1.A, 3i62.1.A, 3iqm.1.A, 3iqy.1.A, 3iuy.1.A, 3j6b.1.5, 3j6b.1.9, 3j9m.35.A, 3jcm.1.I, 3jux.1.A, 3jv2.1.A, 3jv2.2.A, 3klv.1.A, 3kmq.1.A, 3kms.1.A, 3kna.1.A, 3koa.1.A, 3kqh.1.A, 3kql.1.A, 3kqu.1.B, 3kx2.2.A, 3kyl.1.A, 3lkh.2.A, 3llh.1.A, 3llh.2.A, 3llm.1.A, 3llm.1.B, 3ly5.1.A, 3mwj.1.A, 3mwj.1.B, 3mww.2.A, 3mwy.1.A, 3n3w.1.A, 3n6l.1.A, 3n6n.1.A, 3nbf.1.A, 3nbf.2.A, 3nky.1.A, 3nl0.1.A, 3nma.1.A, 3o2r.1.A, 3o2r.2.B, 3o6e.1.C, 3o7v.1.A, 3o7x.1.A, 3o7x.2.B, 3o8b.1.A, 3o8b.2.A, 3o8c.1.A, 3o8d.1.B, 3o8r.1.B, 3oiy.1.A, 3oiy.2.A, 3p1x.1.A, 3p4x.1.A, 3p4x.2.A, 3p4y.1.A, 3peu.1.A, 3pew.1.A, 3pey.1.A, 3q0z.3.A, 3qgg.2.A, 3qir.1.A, 3qir.2.B, 3qir.3.A, 3rc3.1.A, 3rc8.1.A, 3rrm.1.A, 3rrn.1.A, 3rv0.1.A, 3rv0.1.B, 3rv0.2.A, 3rv0.2.B, 3rv1.1.A, 3rv1.1.B, 3rvb.1.A, 3ske.1.A, 3sqw.1.A, 3sqx.1.A, 3tbk.1.A, 3u4r.2.A, 3upi.1.A, 3uwx.1.B, 3v4r.1.A, 3v6d.1.A, 3v6d.2.A, 3v81.1.A, 3v81.2.A, 3vyx.1.A, 3vyy.1.A, 3vyy.2.A, 3zd6.1.A, 3zd7.1.A, 4a15.1.A, 4a2p.1.A, 4a2q.1.A, 4a2q.3.A, 4a2w.1.A, 4a2w.2.A, 4a36.2.A, 4a4d.1.A, 4a4z.1.A, 4a92.1.A, 4aep.1.A, 4ay2.1.A, 4b3p.1.A, 4b6e.1.A, 4b75.1.A, 4be7.1.A, 4beb.1.A, 4bec.1.A, 4bgd.1.A, 4bpb.1.A, 4bru.1.A, 4brw.1.A, 4buj.1.A, 4buj.2.A, 4c9b.1.A, 4cbg.1.A, 4cbg.3.A, 4cbh.4.A, 4cbi.2.A, 4cbi.4.A, 4cbl.1.A, 4cbl.3.A, 4cbm.1.A, 4cbm.3.A, 4cbm.4.A, 4cdg.1.A, 4ce4.1.3, 4cgz.1.A, 4crw.1.B, 4ct4.1.B, 4ct4.2.B, 4ct5.1.A, 4d25.1.A, 4db2.1.A, 4db4.1.A, 4ddt.1.A, 4ddu.1.A, 4ddv.1.A, 4ddw.1.A, 4ddx.1.A, 4dkk.1.A, 4dru.1.A, 4eaw.1.A, 4ern.1.A, 4f91.1.A, 4f92.1.A, 4f93.1.A, 4gl2.1.A, 4gl2.2.A, 4hkq.1.A, 4i1s.1.A, 4ih7.1.A, 4ika.1.A, 4iqx.1.A, 4j02.1.A, 4ju2.2.A, 4k50.4.A, 4kai.1.A, 4kbf.1.A, 4kbf.2.A, 4kbg.2.A, 4khm.1.A, 4khr.1.A, 4kit.1.A, 4ljy.1.A, 4lk2.1.A, 4lk2.2.A, 4m30.1.A, 4m30.1.B, 4mh8.1.A, 4mz4.2.A, 4ngb.1.B, 4ngf.4.A, 4ngf.4.B, 4ngg.1.B, 4nha.2.A, 4nho.1.A, 4nl4.1.A, 4nl8.2.B, 4nld.1.A, 4nlr.1.A, 4nls.1.A, 4nlu.1.A, 4nlv.1.A, 4nlw.1.A, 4nlx.1.A, 4nz0.1.A, 4nz0.2.A, 4nz0.3.A, 4nz0.5.A, 4nz0.6.A, 4o3m.1.A, 4ojq.1.A, 4ojq.1.B, 4ok3.1.A, 4ok3.1.B, 4oks.1.A, 4ol8.1.A, 4ol8.1.B, 4ol8.2.C, 4ol8.2.D, 4on9.1.A, 4on9.2.A, 4oog.1.C, 4oow.1.A, 4oow.2.A, 4oun.1.A, 4pqu.1.A, 4pqu.2.A, 4px9.1.A, 4pxa.1.A, 4q2c.1.A, 4q47.1.A, 4q47.2.A, 4q48.1.A, 4q48.2.A, 4qqw.6.A, 4qqx.1.A, 4qqx.4.A, 4qu4.1.A, 4ry4.1.A, 4ry4.2.A, 4ry5.1.A, 4ry5.2.A, 4ry6.1.A, 4ry7.1.A, 4ry7.2.A, 4s20.1.F, 4s20.2.F, 4tlr.1.A, 4tmu.1.A, 4tyn.1.A, 4tyw.1.A, 4tyy.1.A, 4tz0.1.A, 4u4c.1.A, 4u7d.1.A, 4uaq.1.A, 4v1a.1.H, 4w7s.1.A, 4w7s.1.B, 4wft.2.A, 4wfx.1.A, 4wfy.1.A, 4wxp.1.A, 4wxr.1.A, 4wyl.1.A, 4wyq.1.A, 4wyq.1.B, 4wyq.2.A, 4wyq.2.B, 4wyw.1.A, 4wzm.1.A, 4wzq.1.A, 4x2b.1.A, 4x8w.1.A, 4x8w.1.B, 4x8w.1.C, 4x8w.1.D, 4x8w.2.B, 4xgt.1.A, 4xjx.1.A, 4xjx.1.B, 4xqk.1.A, 4xqk.2.A, 4y2a.1.A, 4y2c.1.A, 4y3c.1.A, 4y3c.2.A, 4y3c.3.A, 4y3c.5.A, 4y3c.6.A, 4ys0.1.A, 4zcf.1.C, 4zp6.1.A, 4zp8.1.A, 4zp9.1.A, 4zpa.1.A, 4zpd.1.A, 5a9j.1.A, 5a9j.3.A, 5aga.1.A, 5anr.2.B, 5aor.1.A, 5aor.2.A, 5b16.1.A, 5b7i.1.A, 5c24.1.A, 5cff.1.C, 5cqg.3.A, 5cqg.3.B, 5czb.1.A, 5d0u.1.A, 5dca.1.A, 5dmq.1.A, 5dtu.1.A, 5dv7.1.C, 5dzr.1.A, 5e02.1.A, 5e3h.1.A, 5e4f.1.A, 5e4f.2.A, 5e7i.1.A, 5e7i.2.A, 5e7i.3.A, 5e7j.1.A, 5e7m.1.A, 5elx.1.A, 5eul.1.A, 5f3o.1.A, 5f3o.1.B, 5f3q.1.A, 5f8g.1.A, 5f98.1.C, 5f9f.1.A, 5f9f.1.E, 5f9h.1.E, 5ffj.2.A, 5ffm.1.A, 5fmf.1.0, 5fmf.1.A, 5g2x.1.C, 5gan.1.I, 5gao.1.I, 5gi4.1.A, 5gi4.1.B, 5gjb.1.A, 5gjc.1.A, 5gju.1.A, 5gm6.1.B, 5gm6.1.W, 5gn1.1.A, 5gn1.4.A, 5gqh.1.A, 5gvr.1.A, 5gvs.1.A, 5gvs.3.A, 5gvu.1.A, 5gvu.2.A, 5gvu.3.A, 5h1y.1.A, 5h1y.2.A, 5h8c.1.A, 5h8w.1.A, 5hhk.1.A, 5hhk.1.B, 5hhl.1.A, 5hzr.1.A, 5i61.1.A, 5i61.2.A, 5i62.1.A, 5i8q.1.A, 5irg.2.B, 5ivl.1.A, 5ivl.1.B, 5ivw.1.A, 5ivw.1.B, 5jaj.1.A, 5jb2.1.A, 5jbj.1.A, 5jc3.1.A, 5jc7.1.A, 5jmt.1.A, 5jps.1.A, 5jpt.1.A, 5jpt.2.A, 5jrz.1.A, 5jwh.1.A, 5jxr.1.A, 5jxs.1.A, 5jxt.1.A, 5jxt.1.B, 5jxt.1.E, 5jxt.1.F, 5jxt.1.L, 5jxt.1.O, 5jxt.1.R, 5jxt.1.S, 5k8l.1.A, 5k8t.1.A, 5k9t.1.A, 5lb3.1.A, 5lb3.2.A, 5lb5.1.A, 5lb8.1.A, 5lba.1.A, 5lba.2.A, 5lba.3.A, 5lj5.1.G, 5lj5.1.c, 5lst.1.A, 5lta.1.A, 5ltj.1.A, 5ltk.1.A, 5m52.1.A, 5m52.2.A, 5m59.1.B, 5m59.2.B, 5m59.3.B, 5m59.4.B, 5m5p.1.A, 5m5p.2.A, 5mc6.34.A, 5mfx.1.A, 5mq0.1.4, 5mrc.31.A, 5mrc.35.A, 5mz4.1.A, 5mz4.1.B, 5n8l.1.A, 5n8m.1.A, 5n8s.1.A, 5n8s.2.A, 5n8u.2.A, 5n8x.1.A, 5n90.2.A, 5n94.1.A, 5n95.1.A, 5n98.2.A, 5n9f.1.A, 5n9f.2.A, 5npa.1.A, 5npg.1.A, 5nt7.1.A, 5o9z.1.C, 5oc4.1.A, 5oc5.1.A, 5oc6.1.A, 5of4.1.A, 5of4.1.B, 5ooq.1.A, 5oqj.1.3, 5oqj.1.W, 5ovn.1.A, 5sup.1.A, 5sup.2.A, 5sup.3.A, 5suq.1.A, 5suq.1.C, 5sva.1.Y, 5sva.1.Z, 5t16.1.A, 5t16.1.B, 5tnu.1.A, 5tnu.2.A, 5twn.2.A, 5txg.1.A, 5txl.1.A, 5txl.2.A, 5txm.1.A, 5txo.1.A, 5uj2.1.A, 5urj.1.A, 5urm.1.A, 5urm.2.A, 5v9x.1.A, 5vbs.1.A, 5vha.1.A, 5vhc.1.A, 5vhe.1.A, 5vi7.1.A, 5vvr.1.M, 5wdx.1.A, 5wsg.1.h, 5wx1.1.A, 5x0x.1.K, 5x0y.1.K, 5xc6.1.A, 5xc7.1.A, 5xdr.1.A, 5xe0.1.A, 5xjc.1.Y, 5y4z.1.A, 5y6m.1.A, 5y6n.1.A, 5y6z.1.A, 5y88.1.W, 5ylz.1.W, 5yvj.1.A, 5yvu.1.A, 5yvv.1.A, 5yvw.1.A, 5yvy.1.B, 5yw1.1.A, 5yzg.1.0, 5yzg.1.5, 5yzg.1.D, 5z3g.1.Y, 5z3l.1.K, 5z3o.1.K, 5z3u.1.A, 5z3v.1.A, 5z56.1.v, 5zak.1.A, 5zak.1.B, 5zal.1.A, 5zal.1.B, 5zam.1.A, 5zam.1.B, 5zbz.1.A, 5zc9.1.A, 5zit.1.A, 5ztm.1.A, 5ztm.1.B, 5zwm.1.0, 6ac6.1.A, 6ac6.1.B, 6ac8.1.B, 6ac8.2.A, 6aca.1.A, 6acx.1.B, 6adw.1.A, 6ah0.1.s, 6ahd.58.A, 6aib.1.A, 6aic.1.A, 6ar1.1.A, 6ar1.2.A, 6b4i.1.C, 6b4j.2.C, 6b4k.1.A, 6b4k.2.A, 6bb8.1.A, 6bk8.1.S, 6bog.1.A, 6bog.2.A, 6bu9.1.A, 6bua.1.A, 6c0f.1.8, 6c66.1.A, 6c90.1.A, 6crm.1.A, 6cz5.1.A, 6d6r.1.M, 6d6v.1.A, 6dcr.1.A, 6dcr.2.A, 6dgd.1.A, 6dgd.2.A, 6e53.1.A, 6ei8.1.A, 6em3.1.0, 6eud.1.A, 6exn.1.T, 6f00.1.A, 6f4a.1.B, 6f9s.1.A, 6fa5.1.A, 6fa9.1.A, 6faa.1.A, 6fac.1.A, 6fml.1.G, 6fsz.1.N, 6ft6.1.t, 6ftx.1.M, 6fwr.1.A, 6fws.1.A, 6fws.1.C, 6g0l.1.K, 6g0l.1.L, 6g19.1.A, 6g1s.1.A, 6g1x.1.A, 6g7e.1.A, 6gaw.44.A, 6gej.1.L, 6gjz.1.A, 6gkh.1.A, 6gox.1.A, 6gpg.1.C, 6gvv.1.A, 6gvy.1.A, 6h57.1.A, 6h61.1.A, 6h66.1.A, 6hak.2.A, 6heg.1.A, 6hts.1.G, 6htu.1.C, 6htu.1.D, 6htu.1.E, 6hys.2.A, 6hyt.3.A, 6hyu.1.A, 6hyu.2.A, 6i3o.1.A, 6i3o.2.A, 6i3p.1.A, 6i3r.1.A, 6i9r.1.a, 6icz.1.b, 6id1.1.g, 6ieg.1.A, 6ieg.2.A, 6ieh.1.A, 6igm.1.H, 6iro.1.A, 6itc.1.A, 6iy2.1.K, 6iy3.1.K, 6jde.1.A, 6jde.2.A, 6jtz.1.A, 6jyl.1.K, 6k1p.1.K, 6kdj.1.A, 6kdj.2.A, 6kr6.1.A, 6kw3.1.1, 6kw4.1.1, 6kw5.1.1, 6kwq.1.A, 6kyv.1.B, 6l5l.1.A, 6l5m.2.A, 6l5m.3.A, 6l5m.4.A, 6l5n.1.A, 6l5n.2.A, 6l5o.1.A, 6l8o.1.A, 6lqs.75.A, 6lsg.1.A, 6ltj.1.I, 6lxd.1.A, 6lxe.1.A, 6m40.1.A, 6m6a.1.F, 6m6b.1.F, 6mec.1.C, 6mh3.1.A, 6mvk.1.A, 6mvo.1.A, 6mvp.1.A, 6mvq.1.A, 6ne3.1.K, 6nmi.1.A, 6nmi.1.B, 6nu2.35.A, 6o16.1.A, 6o16.2.A, 6o5f.1.A, 6o8e.1.A, 6o8e.2.A, 6o8f.1.A, 6o8g.1.A, 6o8g.2.A, 6o8g.3.A, 6o8h.1.A, 6o9l.1.2, 6o9l.1.V, 6o9m.1.A, 6o9m.1.H, 6p4f.1.A, 6p4o.1.A, 6p4o.2.A, 6p4o.3.A, 6p4w.1.A, 6p66.1.A, 6p66.2.A, 6pwf.1.K, 6qdv.1.0, 6qdv.1.D, 6qdv.1.H, 6qic.1.A, 6qic.2.A, 6qic.4.A, 6qid.1.A, 6qie.1.A, 6qv3.1.A, 6qv4.1.A, 6qw6.1.X, 6qws.1.A, 6qx9.23.A, 6qx9.64.A, 6rfl.1.N, 6rm8.1.A, 6rm9.1.A, 6rma.1.A, 6rmb.1.A, 6rmc.1.A, 6rmc.2.A, 6ro1.1.A, 6rwz.1.A, 6ryr.1.K, 6ryu.1.K, 6s0k.1.6, 6s2l.1.A, 6s8o.1.A, 6s8q.1.A, 6s8r.1.A, 6s8s.2.A, 6s9i.1.A, 6sdw.1.A, 6sdy.1.A, 6sh6.1.A, 6sh7.1.A, 6sxa.1.A, 6sxb.1.A, 6sxh.1.A, 6t4h.1.A, 6tda.1.S, 6tnn.1.B, 6tnn.1.C, 6up2.1.A, 6up3.1.A, 6up4.1.A, 6uso.1.A, 6usp.1.A, 6usq.1.A, 6usr.1.A, 6uv0.1.A, 6uv0.2.A, 6uv1.1.A, 6uv2.1.A, 6uv4.1.A, 6uxw.1.N, 6v5b.1.A, 6v5b.1.B, 6v5b.1.C, 6v5c.1.A, 6v5c.1.B, 6v5c.1.C, 6vff.1.B, 6vmi.72.A, 6vz4.1.K, 6x26.1.A, 6x2f.1.A, 6x2n.1.A, 6x43.1.A, 6x4w.1.A, 6x4y.1.A, 6x50.1.A, 6xeo.1.A, 6xki.1.A, 6y53.1.D, 6y5q.1.F, 6ydw.15.A, 6yhr.1.A, 6yvh.1.F, 6ywe.33.A, 6ywe.39.A, 6yxx.15.A, 6yxy.9.A, 6z6f.1.C, 6z6f.1.D, 6z6h.1.C, 6z6h.1.D, 6z6o.1.C, 6z6o.1.D, 6z6p.1.C, 6z6p.1.D, 6zbk.1.B, 6zd1.1.A, 6zd2.1.A, 6zd6.1.A, 6zdp.1.A, 6zdq.1.A, 6zdu.1.A, 6zdu.1.B, 6zdw.1.A, 6zdw.1.B, 6zm2.1.A, 6zm5.35.A, 6zmw.1.t, 6znp.1.A, 6znq.2.A, 6zns.1.A, 6zqd.37.A, 6zqf.1.O, 6zqg.1.8, 6zsa.65.A, 6zsc.65.A, 6zse.65.A, 6zww.1.A, 6zww.2.A, 6zww.3.A, 6zww.4.A, 6zwx.1.A, 7a5f.35.A, 7a5g.35.A, 7a5h.1.7, 7a5i.36.A, 7a5k.36.A, 7a5p.1.e, 7a8r.1.A, 7a8r.2.A, 7abg.1.H, 7ad8.1.C, 7ad8.1.E, 7ahx.1.A, 7ajt.79.A, 7aju.74.A, 7akp.1.A, 7am2.64.A, 7amv.1.M, 7aoh.1.M, 7aoi.59.A, 7apk.1.G, 7apx.1.F, 7ase.1.A, 7ask.1.A, 7auc.1.A, 7aud.1.A, 7aud.2.A, 7aud.3.A, 7aud.4.A, 7aud.6.A, 7b9v.1.E, 7b9v.1.T, 7bbb.1.A, 7bdi.1.A, 7bdj.1.A, 7bdk.1.A, 7bdl.1.A, 7bg9.1.A, 7bkp.1.A, 7bkq.1.A, 7blv.1.A, 7bm0.1.A, 7bst.1.A, 7bst.1.B, 7btp.1.B, 7btq.1.F, 7clg.1.A, 7clg.1.B, 7d0f.1.B, 7d0g.1.B, 7d1a.1.A, 7d4i.78.A, 7d5t.1.r, 7dco.1.D, 7dcp.1.A, 7dcq.1.A, 7dcr.1.A, 7dd3.1.A, 7ddx.1.B, 7dey.1.A, 7dey.1.B, 7dey.2.A, 7dey.2.B, 7dtj.1.A, 7dtk.1.A, 7dtk.1.B, 7e4v.1.A, 7egb.1.G, 7egc.1.G, 7egc.1.H, 7egp.1.H, 7eld.1.A, 7ele.1.A, 7enc.31.A, 7enc.32.A, 7enn.1.A, 7epu.1.B, 7evn.1.E, 7evo.1.L, 7fse.1.A, 7fsf.1.A, 7gqs.1.A, 7gqt.1.A, 7gqu.1.A, 7jl0.1.C, 7jl1.1.A, 7jno.1.A, 7k01.1.C, 7k9y.1.A, 7kft.1.D, 7kqm.1.A, 7kqn.1.A, 7kse.1.A, 7ksf.1.A, 7lbm.1.0, 7lbm.1.1, 7liu.1.A, 7lmb.1.D, 7luv.1.F, 7m2u.1.A, 7m2u.1.E, 7m8e.1.F, 7mkn.1.F, 7mkq.1.F, 7ml0.1.1, 7ml0.1.U, 7ml1.1.C, 7ml1.1.G, 7ml2.1.2, 7ml3.1.C, 7ml3.1.H, 7ml4.1.Q, 7ml4.1.U, 7mqa.43.A, 7mqj.1.A, 7nac.1.v, 7nga.1.C, 7nic.1.A, 7niq.1.A, 7nkx.1.U, 7nqh.37.A, 7nsh.1.K, 7nvv.1.C, 7o0g.1.A, 7o0h.1.A, 7o0h.1.B, 7o24.1.A, 7o24.1.B, 7o4i.1.H, 7o4j.1.H, 7o4l.1.H, 7o72.1.A, 7o72.1.H, 7o73.1.H, 7o75.1.H, 7o9k.40.A, 7o9m.36.A, 7ohp.1.F, 7ohr.1.G, 7ohs.1.J, 7ohv.1.G, 7ohw.1.J, 7ohx.1.I, 7oi6.1.e, 7oi7.1.7, 7oi9.1.7, 7oib.1.7, 7oic.1.a, 7oid.1.8, 7oie.1.8, 7oo3.1.Q, 7oob.1.M, 7oop.1.X, 7opc.1.X, 7oqb.1.T, 7os1.1.A, 7os2.1.A, 7otq.1.A, 7otz.2.A, 7pd3.1.b, 7pli.1.C, 7pli.2.A, 7pli.2.C, 7pmm.1.B, 7pmq.1.A, 7pmq.2.B, 7ppz.1.B, 7pq0.1.B, 7px3.1.A, 7qdr.1.A, 7qdz.1.A, 7qe0.1.A, 7qh6.1.4, 7qh7.1.5, 7qtt.1.K, 7qxa.1.A, 7qxb.1.A, 7r06.1.A, 7r08.1.A, 7r08.2.A, 7r08.2.C, 7r2k.1.A, 7r6q.1.B, 7r76.1.A, 7r77.1.A, 7r78.1.A, 7r7j.1.A, 7r7j.2.A, 7r97.1.A, 7s7b.1.A, 7s7c.1.A, 7s9v.1.A, 7s9w.1.A, 7sr6.1.A, 7sr6.1.B, 7ssg.1.A, 7t02.1.A, 7tbm.98.A, 7tn2.1.K, 7tnx.1.A, 7tny.1.A, 7tnz.1.A, 7to0.1.A, 7to1.1.A, 7to2.1.A, 7tr8.1.A, 7tr9.1.P, 7tra.1.A, 7trd.1.B, 7uim.1.B, 7uin.1.B, 7ujb.1.A, 7ux9.1.I, 7uy5.1.B, 7uy6.1.B, 7v2y.1.F, 7v2z.1.A, 7v4q.1.A, 7v4r.1.A, 7v6b.1.A, 7v6b.1.B, 7v6c.1.A, 7v6c.1.B, 7v99.1.A, 7v9u.1.A, 7v9u.1.B, 7v9x.1.F, 7vdt.1.A, 7vdv.1.J, 7vg2.1.A, 7vg3.1.A, 7w0a.1.A, 7w0b.1.A, 7w0c.1.A, 7w0d.1.D, 7w0d.1.E, 7w0e.1.C, 7w0f.1.A, 7w1r.1.A, 7w59.1.Y, 7w5a.1.Y, 7w5b.1.Y, 7wd4.1.A, 7x3t.1.T, 7xex.1.A, 7xex.2.A, 7xex.3.A, 7xf0.1.A, 7xf0.2.A, 7xf0.3.A, 7xf1.1.A, 7xg3.1.L, 7xha.1.A, 7xhb.1.A, 7xt0.1.A, 7xw2.1.A, 7xw3.1.A, 7xwy.1.A, 7xxe.1.A, 7xxe.2.A, 7xyf.1.G, 7xyg.1.G, 7y8r.1.J, 7yfx.1.A, 7yfy.1.A, 7ygn.1.A, 7ymf.1.A, 7ymf.1.B, 7yym.1.A, 7yyn.1.B, 7yz4.1.A, 7z0z.1.A, 7z4y.1.B, 7z4y.1.D, 7z52.1.A, 7z8s.1.D, 7zb5.1.D, 7zi4.1.G, 7zj1.1.A, 7zj1.1.B, 7zke.1.D, 7zlq.1.A, 7zmm.2.A, 7zmm.3.A, 7zmm.4.A, 7zmn.1.A, 7zmo.1.A, 7zmp.1.A, 7zmp.2.A, 7zmq.2.A, 7zmr.1.A, 7zms.1.A, 7zmt.1.A, 7zmv.2.A, 7znj.1.A, 7znk.1.G, 7zpi.1.A, 7zpj.1.A, 7zpj.1.C, 7zpk.1.C, 7zpq.75.A, 7zrs.75.A, 7zsa.1.3, 7zsb.1.3, 7zuw.75.A, 8alz.1.B, 8ark.1.A, 8ark.2.A, 8arp.1.A, 8arp.1.B, 8arp.1.C, 8arp.1.D, 8arp.1.E, 8arp.1.F, 8atf.1.A, 8av6.1.G, 8b02.1.A, 8b0a.1.A, 8b3d.1.R, 8b3f.1.R, 8b9g.1.A, 8b9i.1.A, 8b9j.1.A, 8b9k.1.A, 8b9l.1.A, 8bc8.1.A, 8bc9.1.A, 8bca.1.A, 8bcb.1.A, 8bcc.1.A, 8bcd.1.A, 8bce.1.A, 8bcf.1.A, 8bcg.1.A, 8bch.1.A, 8bgj.1.A, 8bgj.2.A, 8bgj.3.A, 8bvw.1.A, 8bvw.1.B, 8byq.1.A, 8byq.1.B, 8c1n.1.A, 8c1n.1.B, 8c2p.1.A, 8c6j.2.A, 8c6j.30.A, 8c6j.6.A, 8c8j.1.A, 8cen.1.H, 8ceo.1.A, 8ch6.1.9, 8ch6.1.U, 8cnt.1.A, 8dfv.1.A, 8dfv.1.C, 8dg5.1.A, 8dg5.1.C, 8dg7.1.A, 8dg7.1.D, 8dga.1.A, 8dga.1.D, 8dgi.1.A, 8dgi.1.B, 8dgj.1.A, 8dgj.1.B, 8dpe.1.A, 8dvs.1.A, 8dw1.1.A, 8dwm.1.A, 8e0f.1.B, 8e2w.1.A, 8ebs.1.B, 8ebt.1.A, 8ebt.1.B, 8ebu.1.A, 8ebu.1.B, 8ebw.1.A, 8ejm.1.A, 8enk.1.A, 8enk.1.B, 8esr.1.I, 8ets.1.A, 8etw.1.A, 8eu9.1.A, 8euf.1.A, 8eup.1.J, 8fak.1.D, 8fks.1.j, 8fkt.1.0, 8fli.1.B, 8flj.1.M, 8flj.1.N, 8g7t.1.A, 8g7t.1.C, 8g7u.1.A, 8g7u.1.C, 8g7v.1.A, 8g7v.1.C, 8g9u.1.A, 8gh6.1.A, 8gzq.1.B, 8gzr.1.B, 8h2h.1.C, 8h5y.2.A, 8h5z.1.A, 8h5z.2.A, 8h6e.1.M, 8h6j.1.5, 8h6j.1.M, 8h6l.1.E, 8he5.1.O, 8hf0.1.A, 8hf0.1.D, 8hf1.1.A, 8hf1.1.D, 8hf1.1.F, 8huj.1.A, 8i0r.1.R, 8i0t.1.W, 8i0v.1.V, 8i0w.1.0, 8i0w.1.5, 8i9j.1.B, 8i9j.1.C, 8i9p.1.F, 8i9r.1.F, 8i9t.1.G, 8i9t.1.Y, 8i9v.1.0, 8i9v.1.G, 8i9w.1.F, 8i9w.1.Z, 8i9x.1.0, 8i9y.1.G, 8i9z.1.G, 8i9z.1.Y, 8ia0.23.A, 8ia0.5.A, 8ibw.1.C, 8ibx.1.A, 8iby.1.B, 8ibz.1.A, 8igd.1.A, 8igd.2.A, 8iju.1.A, 8izn.1.A, 8j90.1.K, 8jix.1.A, 8k22.1.R, 8kca.1.A, 8kca.2.A, 8kcb.1.K, 8kcc.1.K, 8kfp.1.A, 8ofb.1.A, 8ohm.1.A, 8oo7.1.G, 8oop.1.G, 8oor.1.G, 8oz0.1.s, 8oz0.1.t, 8oz7.1.A, 8oz7.1.B, 8pfl.1.A, 8pfp.1.A, 8pjb.1.A, 8pjj.1.A, 8pk0.1.Z, 8pnk.1.A, 8po6.1.A, 8po7.1.A, 8po8.1.A, 8po8.1.B, 8q7w.1.F, 8q9t.1.A, 8qbk.1.C, 8qcf.1.L, 8qr1.1.A, 8qzs.2.A, 8r08.10.A, 8r08.26.A, 8r0a.1.M, 8r0b.1.B, 8r0s.1.A, 8rc0.1.E, 8rev.1.A, 8rm5.1.9, 8ro0.1.E, 8ro1.1.G, 8scz.1.A, 8sd0.1.A, 8so8.1.A, 8sp4.1.A, 8spg.1.A, 8ssw.1.A, 8sxt.1.C, 8sxu.1.A, 8szp.1.A, 8szp.2.A, 8szq.1.A, 8szr.1.A, 8t2r.1.A, 8t2s.1.A, 8t2t.1.B, 8t5s.1.A, 8tbx.1.A, 8tcj.1.A, 8tck.1.A, 8tcl.1.A, 8tvy.1.M, 8u6n.1.A, 8uw3.1.A, 8v44.1.A, 8v4y.1.K, 8v83.1.I, 8v84.1.C, 8v87.1.C, 8v87.1.J, 8vx9.1.B, 8vxa.1.C, 8vxc.1.B, 8vxy.1.D, 8w0a.1.A, 8wap.1.G, 8wap.1.H, 8wh5.1.K, 8wh8.1.K, 8wh9.1.K, 8wtk.1.A, 8wus.1.A, 8wut.1.B, 8x15.1.I, 8x19.1.I, 8x1c.1.I, 8x1z.1.A, 8xt1.1.a, 8xvg.1.I, 8xxn.1.a, 8y6o.1.E, 8y6o.1.I, 8yle.1.A, 8ynj.1.A, 9asj.1.A, 9ask.1.A, 9bh6.1.A, 9bh7.1.A, 9bh8.1.A, 9bh8.1.B, 9bh9.1.B, 9c57.1.H, 9c5q.1.B, 9c62.1.F, 9fmd.10.A, 9fmd.31.A

Swiss Institute of Bioinformatics
Contact Us
