# Supplementary material for: Molecular Ruler Variation in Insect Dicer-2 Suggests a Structural Basis for Species-Dependent siRNA Length and Antiviral Defense Diversity
Source: Viruses. 2026 Feb 27;18(3):285. doi: 10.3390/v18030285 (PMC13030778; doi:10.3390/v18030285)
Supplement: Supplementary file 1 [file viruses-18-00285-s001.zip › Thricoplusia_report_7w0e.html]

Thrice\_dicer2 | Report


Homology Modelling Report

## Model Building Report

This document lists the results for the homology modelling project "Thrice\_dicer2" submitted to SWISS-MODEL workspace
on Dec. 20, 2024, 5:01 p.m..The submitted primary amino acid sequence is given in Table T1.

If you use any results in your research, please cite the relevant publications:

- Waterhouse A, Bertoni M, Bienert S, Studer G, Tauriello G, Gumienny R, Heer FT, de Beer TAP, Rempfer C, Bordoli L, Lepore R, Schwede T

  SWISS-MODEL: homology modelling of protein structures and complexes.

  Nucleic Acids Res 46, W296-W303. (2018) 2978835510.1093/nar/gky427
- Bienert S, Waterhouse A, de Beer TAP, Tauriello G, Studer G, Bordoli L, Schwede T

  The SWISS-MODEL Repository - new features and functionality.

  Nucleic Acids Res 45, D313-D319. (2017) 2789967210.1093/nar/gkw1132
- Studer G, Tauriello G, Bienert S, Biasini M, Johner N, Schwede T

  ProMod3 - A versatile homology modelling toolbox.

  PLOS Comp Biol 17(1), e1008667. (2021) 3350798010.1371/journal.pcbi.1008667
- Studer G, Rempfer C, Waterhouse AM, Gumienny R, Haas J, Schwede T

  QMEANDisCo - distance constraints applied on model quality estimation.

  Bioinformatics 36, 1765-1771. (2020) 3169731210.1093/bioinformatics/btz828
- Bertoni M, Kiefer F, Biasini M, Bordoli L, Schwede T

  Modeling protein quaternary structure of homo- and hetero-oligomers beyond binary interactions by homology.

  Scientific Reports 7. (2017) 2887468910.1038/s41598-017-09654-8

## Results

The user uploaded a template structure to use for the modelling process.

## Models

The following model was built (see Materials and Methods "Model Building"):

| Model #01 | File | Built with | Oligo-State | Ligands | GMQE | QMEANDisCo Global |
| --- | --- | --- | --- | --- | --- | --- |
|  | PDB | ProMod3 3.4.1 | monomer | None | 0.34 | 0.51 ± 0.05 |

|  |  |  |
| --- | --- | --- |
|  |  |  |

| Template | Seq Identity | Oligo-state | QSQE | Found by | Method | Resolution | Seq Similarity | Range | Coverage | Description |
| --- | --- | --- | --- | --- | --- | --- | --- | --- | --- | --- |
| template\_upload.1.A | 29.07 | monomer | 0.00 | HHblits | Unknown | - | 0.35 | 461 - 2083 | 0.69 | Polypeptide |

  

### The template contained no ligands.

  

```
Target                   MKIGVTFKSKDVEKTIRCMSRGKSPGHDGLSIEHLRYAGAHMSRVLSMLYNICVG  
template_upload.1.A      -------------------------------------------------------  
  
Target                   HSYLPSDMMRTVVVPIVKNKTGDLADKDNYRPISLATVISKVLDSMLNTQLNKYL  
template_upload.1.A      -------------------------------------------------------  
  
Target                   CLYDNQFGFRAGLSTESAILGLKHAVKYYTQRATSVYACFLDLSRAFDMVSYDYL  
template_upload.1.A      -------------------------------------------------------  
  
Target                   WKKLQNIKMPTEIVNIFKYWYGNQINNVRWAGALSLPYRLECGVRQGGLSSPTLF  
template_upload.1.A      -------------------------------------------------------  
  
Target                   NLYVNELIGELSGTRVGCFIDGVCVNNISYADDMVLLSASICGLRKLVSLCEEYA  
template_upload.1.A      -------------------------------------------------------  
  
Target                   KSHGLVYNCKKSEIMVFETRGRTHDNIPPLILNGTALRRVFRFKYLGHVLTPSLK  
template_upload.1.A      -------------------------------------------------------  
  
Target                   DDEDIERERRALSVRANMIARRFARCSLKVKLTLFRAYCTNFYTCSLWAGYTQRT  
template_upload.1.A      -------------------------------------------------------  
  
Target                   YNALRVQYNNAFRVLVGLPRFCSASGMFADAQVDCFYATMRKRCASLVSRVRASS  
template_upload.1.A      -------------------------------------------------------  
  
Target                   NSILNMIASRLDCVYLGRCCAISHGLLWRTQIKNVIEKLCAVSGVGAYSSENGVD  
template_upload.1.A      --------------------FMCNTVELARQQAMAVRRCTNF-KVGFYVGEQGVD  
  
Target                   YWDKAKWDAELEKNQVIVMTSQILNDMLTHQYIRIEDINLLIFDECHHAVEDHPM  
template_upload.1.A      DWTRGMWSDEIKKNQVLVGTAQVFLDMVTQTYVALSSLSVVIIDECHHGTGHHPF  
  
Target                   RVIMKHFEGCPKHSQPRVLGLTATLLNANVKSRKVEDTLHDLEITFHATIATVDE  
template_upload.1.A      REFMRLFTIANQTKLPRVVGLTGVLIKGN-EITNVATKLKELEITYRGNIITVSD  
  
Target                   LG---KVLNYSTNPNEMVQFYRSSPPSA-VTNEVIKLLSVRQELIASVKLPRSST  
template_upload.1.A      TKEMENVMLYATKPTEVMVSFPHQEQVLTVTRLISAEIE---KFYVSLDLMNI-G  
  
Target                   KQTITLKQHQEDISNNPKKIVKAVKNMISSMILFLNELGMYGGSLGILAYIILLE  
template_upload.1.A      VQPIRRSKSLQCLR--DPSKKSFVKQLFNDFLYQMKEYGIYAASIAIISLIVEFD  
  
Target                   RLRRRASSKEEDILYQNVITCCIDARARLLKAMSDV---------HGYERIIKYS  
template_upload.1.A      IKRRQAETLSVKLMHRTALTLCEKIRHLLVQKLQDMTYDDDDDNVNTEEVIMNFS  
  
Target                   SEKVLLTLNILKEYNPAYQDTPGVLLKVNRSRKPLSAIIFTKQRFTAKVLYNLLK  
template_upload.1.A      TPKVQRFLMSLKVSFAD------------KDPKDICCLVFVERRYTCKCIYGLLL  
  
Target                   DVRDSNPAEFDFLKHDFVVGFNVNPLKSTREEYYIKKTGQQALLKFGNNDLNCLI  
template_upload.1.A      NYIQSTPELRNVLTPQFMVGRNNISP--DFESVLERKWQKSAIQQFRDGNANLMI  
  
Target                   STSVIEEGIDIPQCLLVLRYDQPLEYRSYIQSKGRARSSESSYVILVNREDEKKF  
template_upload.1.A      CSSVLEEGIDVQACNHVFILDPVKTFNMYVQSKGRARTTEAKFVLFTADKEREKT  
  
Target                   MTLYKEFQETEQLIQRILVGNTDDRDEPAQENIDKNLYQDEDVPPFISPYGGRLT  
template_upload.1.A      IQQIYQYRKAHNDIAEYLKDRVLEKTEPELYEIKG-HF-QDDIDPFTNENGAVLL  
  
Target                   ATSAISLLNRYCSMLPHDHFTIITPMWIKETVT---------NKHGFDCNLVTIV  
template_upload.1.A      PNNALAILHRYCQTIPTDAFGFVIPWFHVLQEDERDRIFGVSAKGKHVI---SIN  
  
Target                   LPIACPIKEEIKGMPMYNLKSAKRSAALNACVKLYEAGELDPLTMLPMRYTAVDF  
template_upload.1.A      MPVNCMLRDTIYSDPMDNVKTAKISAAFKACKVLYSLGELNER-FVPKTLKERVA  
  
Target                   DDADVQSCFLNWRNDDMKRVDDPDYPAPGTKGRVRKHRIQFPA-VLDSVPD-ESE  
template_upload.1.A      SIAD--VHFEHWNKYGDS---VTATVNKADKSKDRTYKTECPLEFYDALPRVGEI  
  
Target                   YYLHIIKTTTAFAEPKDTREKALYDLLHRPEGFGFMTQKPLPAICDFPMFMTVGE  
template_upload.1.A      CYAYEIFLEPQFESCE--YTEHMYLNLQTPRNYAILLRNKLPRLAEMPLFSNQGK  
  
Target                   VSTSLDVNY-AVIKLDAKLLQLVKQFHFFIFEQVLAIAKKFIVFEG--KVNCLYV  
template_upload.1.A      LHVRVANAPLEVIIQNSEQLELLHQFHGMVFRDILKIWHPFFVLDRRSKENSYLV  
  
Target                   VPVKEDN--GYNIDWDVMATHDQIQPVTPPPYEDR-INLKVTPENYKDCVVTPWY  
template_upload.1.A      VPLILGAGEQKCFDWELMTNFRRLPQSHGSNVQQREQQPAPRPEDFEGKIVTQWY  
  
Target                   RVLPDRYIVSRVLEFMTPQSHFDSD-SYVTFADYYADKYKLEI--IGDKSQALLE  
template_upload.1.A      ANYDKPMLVTKVHRELTPLSYMEKNQQDKTYYEFTMSKYGNRIGDVVHKDKFMIE  
  
Target                   VRNISSRMNCLLPRAATINSFTDKQKKLVSASQGDDKTNRGFAEVFVAEFCIKYD  
template_upload.1.A      VRDLTEQLTFYVHNRGKFN---A-------------KSKAKMKVILIPELCFNFN  
  
Target                   FPGVLWYKAIMLPSIVHRVFMLLVAHELLTEISESTKYGNPKRRKGEEWRPVSSN  
template_upload.1.A      FPGDLWLKLIFLPSILNRMYFLLHAEALRKRFNTYLNLHLLPF-NGTDYMP----  
  
Target                   MQIATLSLLAQVEEPTPITSVDRINNPTDDENPRRPNIMSIKQSLYQLQQKKLSK  
template_upload.1.A      -------------------------------------------------------  
  
Target                   DYPWDEKMEPIDIERNLSTVTVMDIECYDEFVSSPLVPIMSPTRVLSPPRVVVGS  
template_upload.1.A      --------RPLEIDYSLK---------------GKVKPL---------------L  
  
Target                   KISAAISAPPAKYNDKLNILKMTATGNGPELRDILTALTTIKSHDTFNLERVETL  
template_upload.1.A      ILQKTVS---------------KEHITPAEQGEFLAAITASSAADVFDMERLEIL  
  
Target                   GDAFLKFAASLYLFHKFPKFNEGQLTNIKGRLISNRNLYYAGERFNLAGRMKVEQ  
template_upload.1.A      GNSFLKLSATLYLASKYSDWNEGTLTEVKSKLVSNRNLLFCLIDADIPKTLNTIQ  
  
Target                   FSPRKDFMVPGYFAPPEVEKFIAEK-----KLRPTFLIGVYFPSSEA-FDGNLSK  
template_upload.1.A      FTPRYTWLPPGISLPHNVLALWRENPEFAKIIGPHNLRDLALGDEESLVKGNCSD  
  
Target                   ESMAMVRDRFADCDGTAETEPECRVQNAMQLYIHSQAVADKSVADCVEALIGTYL  
template_upload.1.A      INYNRFVEGCRA-NGQ-SFYAGADFSSEVNFCVGLVTIPNKVIADTLEALLGVIV  
  
Target                   LSGGVLGAIKVIEWMRIIPPQDN--FATYLHTRVS-TVLSEKRATESDINFLLSH  
template_upload.1.A      KNYGLQHAFKMLEYFKICRADIDKPLTQLLNLELGGKKMRA-NVNTTEIDGFL-I  
  
Target                   CRPDVEKILNYKFKDPSHLLEALSHPSYIRNRLTRSYERYEFLGDAILDFLITSH  
template_upload.1.A      NHYYLEKNLGYTFKDRRYLLQALTHPSYPTNRITGSYQELEFIGNAILDFLISAY  
  
Target                   VFENCGDLKPGEMTDLRSALVNNVTFASYVVKLGLHKFLCSELNPTLDKAVITFV  
template_upload.1.A      IFENNTKMNPGALTDLRSALVNNTTLACICVRHRLHFFILAE-NAKLSEIISKFV  
  
Target                   DHQVQREHQIVEDVLYLIDEEECHIAEYVEVPKVLSDIFEALVGAIFLDSGGDLQ  
template_upload.1.A      NFQESQGHR------------VTNMSTNVDVPKALGDVLEALIAAVYLDCR-DLQ  
  
Target                   TVWALVYRIMCKEIHAFSSRIPQQPVKVLYEKIHACPVFDKSVVIDPDIPKIKVG  
template_upload.1.A      RTWEVIFNLFEPELQEFTRKVPINHIRQLVEHKHAKPVFSSPIVEGE-TVMVSCQ  
  
Target                   VTITKNDWQHTVYGVGKNKSQAKRAAAKMALKVLGI  
template_upload.1.A      FTCME--KTIKVYGFGSNKDQAKLSAAKHALQQLS-
```

  


---

  

## Materials and Methods

## User Template Alignment

The user entered their own target sequence together with an uploaded a template structure file in PDB format.

## Model Building

Models are built based on the target-template alignment using ProMod3 (Studer et al.). Coordinates which are conserved between the target and the template are copied from the template to the model. Insertions and deletions are remodelled using a fragment library. Side chains are then rebuilt. Finally, the geometry of the resulting model is regularized by using a force field.

## Model Quality Estimation

The global and per-residue model quality has been assessed using the QMEAN scoring function (Studer et al.).

## Ligand Modelling

Ligands present in the template structure are transferred by homology to the model when the following criteria are met: (a) The ligands are annotated as biologically relevant in the template library, (b) the ligand is in contact with the model, (c) the ligand is not clashing with the protein, (d) the residues in contact with the ligand are conserved between the target and the template. If any of these four criteria is not satisfied, a certain ligand will not be included in the model. The model summary includes information on why and which ligand has not been included.

## Oligomeric State Conservation

The quaternary structure annotation of the template is used to model the target sequence in its oligomeric form. The method (Bertoni et al.) is based on a supervised machine learning algorithm, Support Vector Machines (SVM), which combines interface conservation, structural clustering, and other template features to provide a quaternary structure quality estimate (QSQE). The QSQE score is a number between 0 and 1, reflecting the expected accuracy of the interchain contacts for a model built based a given alignment and template. Higher numbers indicate higher reliability. This complements the GMQE score which estimates the accuracy of the tertiary structure of the resulting model.

## References

- Camacho C, Coulouris G, Avagyan V, Ma N, Papadopoulos J, Bealer K, Madden TL

  BLAST+: architecture and applications.

  BMC Bioinformatics, 10, 421-430. (2009) 2000350010.1186/1471-2105-10-421
- Steinegger M, Meier M, Mirdita M, Vöhringer H, Haunsberger SJ, Söding J

  HH-suite3 for fast remote homology detection and deep protein annotation.

  BMC Bioinformatics 20, 473. (2019) 3152111010.1186/s12859-019-3019-7

## Table T1:

Primary amino acid sequence for which templates were searched and models were built.

MKIGVTFKSKDVEKTIRCMSRGKSPGHDGLSIEHLRYAGAHMSRVLSMLYNICVGHSYLPSDMMRTVVVPIVKNKTGDLADKDNYRPISLATVISKVLDS  
MLNTQLNKYLCLYDNQFGFRAGLSTESAILGLKHAVKYYTQRATSVYACFLDLSRAFDMVSYDYLWKKLQNIKMPTEIVNIFKYWYGNQINNVRWAGALS  
LPYRLECGVRQGGLSSPTLFNLYVNELIGELSGTRVGCFIDGVCVNNISYADDMVLLSASICGLRKLVSLCEEYAKSHGLVYNCKKSEIMVFETRGRTHD  
NIPPLILNGTALRRVFRFKYLGHVLTPSLKDDEDIERERRALSVRANMIARRFARCSLKVKLTLFRAYCTNFYTCSLWAGYTQRTYNALRVQYNNAFRVL  
VGLPRFCSASGMFADAQVDCFYATMRKRCASLVSRVRASSNSILNMIASRLDCVYLGRCCAISHGLLWRTQIKNVIEKLCAVSGVGAYSSENGVDYWDKA  
KWDAELEKNQVIVMTSQILNDMLTHQYIRIEDINLLIFDECHHAVEDHPMRVIMKHFEGCPKHSQPRVLGLTATLLNANVKSRKVEDTLHDLEITFHATI  
ATVDELGKVLNYSTNPNEMVQFYRSSPPSAVTNEVIKLLSVRQELIASVKLPRSSTKQTITLKQHQEDISNNPKKIVKAVKNMISSMILFLNELGMYGGS  
LGILAYIILLERLRRRASSKEEDILYQNVITCCIDARARLLKAMSDVHGYERIIKYSSEKVLLTLNILKEYNPAYQDTPGVLLKVNRSRKPLSAIIFTKQ  
RFTAKVLYNLLKDVRDSNPAEFDFLKHDFVVGFNVNPLKSTREEYYIKKTGQQALLKFGNNDLNCLISTSVIEEGIDIPQCLLVLRYDQPLEYRSYIQSK  
GRARSSESSYVILVNREDEKKFMTLYKEFQETEQLIQRILVGNTDDRDEPAQENIDKNLYQDEDVPPFISPYGGRLTATSAISLLNRYCSMLPHDHFTII  
TPMWIKETVTNKHGFDCNLVTIVLPIACPIKEEIKGMPMYNLKSAKRSAALNACVKLYEAGELDPLTMLPMRYTAVDFDDADVQSCFLNWRNDDMKRVDD  
PDYPAPGTKGRVRKHRIQFPAVLDSVPDESEYYLHIIKTTTAFAEPKDTREKALYDLLHRPEGFGFMTQKPLPAICDFPMFMTVGEVSTSLDVNYAVIKL  
DAKLLQLVKQFHFFIFEQVLAIAKKFIVFEGKVNCLYVVPVKEDNGYNIDWDVMATHDQIQPVTPPPYEDRINLKVTPENYKDCVVTPWYRVLPDRYIVS  
RVLEFMTPQSHFDSDSYVTFADYYADKYKLEIIGDKSQALLEVRNISSRMNCLLPRAATINSFTDKQKKLVSASQGDDKTNRGFAEVFVAEFCIKYDFPG  
VLWYKAIMLPSIVHRVFMLLVAHELLTEISESTKYGNPKRRKGEEWRPVSSNMQIATLSLLAQVEEPTPITSVDRINNPTDDENPRRPNIMSIKQSLYQL  
QQKKLSKDYPWDEKMEPIDIERNLSTVTVMDIECYDEFVSSPLVPIMSPTRVLSPPRVVVGSKISAAISAPPAKYNDKLNILKMTATGNGPELRDILTAL  
TTIKSHDTFNLERVETLGDAFLKFAASLYLFHKFPKFNEGQLTNIKGRLISNRNLYYAGERFNLAGRMKVEQFSPRKDFMVPGYFAPPEVEKFIAEKKLR  
PTFLIGVYFPSSEAFDGNLSKESMAMVRDRFADCDGTAETEPECRVQNAMQLYIHSQAVADKSVADCVEALIGTYLLSGGVLGAIKVIEWMRIIPPQDNF  
ATYLHTRVSTVLSEKRATESDINFLLSHCRPDVEKILNYKFKDPSHLLEALSHPSYIRNRLTRSYERYEFLGDAILDFLITSHVFENCGDLKPGEMTDLR  
SALVNNVTFASYVVKLGLHKFLCSELNPTLDKAVITFVDHQVQREHQIVEDVLYLIDEEECHIAEYVEVPKVLSDIFEALVGAIFLDSGGDLQTVWALVY  
RIMCKEIHAFSSRIPQQPVKVLYEKIHACPVFDKSVVIDPDIPKIKVGVTITKNDWQHTVYGVGKNKSQAKRAAAKMALKVLGI

## Table T2:

| Template | Seq Identity | Oligo-state | QSQE | Found by | Method | Resolution | Seq Similarity | Coverage | Description |
| --- | --- | --- | --- | --- | --- | --- | --- | --- | --- |
| template\_upload.1.A | 29.07 | monomer | - | HHblits | Unknown | NA | 0.35 | 0.69 | Polypeptide |
| template\_upload.1.A | 30.71 | monomer | - | BLAST | Unknown | NA | 0.37 | 0.43 | Polypeptide |
| template\_upload.1.A | 37.66 | monomer | - | BLAST | Unknown | NA | 0.39 | 0.22 | Polypeptide |
| template\_upload.1.A | 30.86 | monomer | - | HHblits | Unknown | NA | 0.34 | 0.04 | Polypeptide |

  
The table above shows the top 4 filtered templates. A further 1 template was found which was considered to be less suitable for modelling than the filtered list.  
template\_upload.1.A

Swiss Institute of Bioinformatics
Contact Us
