# Supplementary material for: Molecular Ruler Variation in Insect Dicer-2 Suggests a Structural Basis for Species-Dependent siRNA Length and Antiviral Defense Diversity
Source: Viruses. 2026 Feb 27;18(3):285. doi: 10.3390/v18030285 (PMC13030778; doi:10.3390/v18030285)
Supplement: Supplementary file 1 [file viruses-18-00285-s001.zip › Tribolium_report_7V6C.html]

Tribolium\_dcr-2 | Report


Homology Modelling Report

## Model Building Report

This document lists the results for the homology modelling project "Tribolium\_dcr-2" submitted to SWISS-MODEL workspace
on Aug. 21, 2024, 4:57 p.m..The submitted primary amino acid sequence is given in Table T1.

If you use any results in your research, please cite the relevant publications:

- Waterhouse A, Bertoni M, Bienert S, Studer G, Tauriello G, Gumienny R, Heer FT, de Beer TAP, Rempfer C, Bordoli L, Lepore R, Schwede T

  SWISS-MODEL: homology modelling of protein structures and complexes.

  Nucleic Acids Res 46, W296-W303. (2018) 2978835510.1093/nar/gky427
- Bienert S, Waterhouse A, de Beer TAP, Tauriello G, Studer G, Bordoli L, Schwede T

  The SWISS-MODEL Repository - new features and functionality.

  Nucleic Acids Res 45, D313-D319. (2017) 2789967210.1093/nar/gkw1132
- Studer G, Tauriello G, Bienert S, Biasini M, Johner N, Schwede T

  ProMod3 - A versatile homology modelling toolbox.

  PLOS Comp Biol 17(1), e1008667. (2021) 3350798010.1371/journal.pcbi.1008667
- Studer G, Rempfer C, Waterhouse AM, Gumienny R, Haas J, Schwede T

  QMEANDisCo - distance constraints applied on model quality estimation.

  Bioinformatics 36, 1765-1771. (2020) 3169731210.1093/bioinformatics/btz828
- Bertoni M, Kiefer F, Biasini M, Bordoli L, Schwede T

  Modeling protein quaternary structure of homo- and hetero-oligomers beyond binary interactions by homology.

  Scientific Reports 7. (2017) 2887468910.1038/s41598-017-09654-8

## Results

The SWISS-MODEL template library (SMTL version 2024-08-14, PDB release 2024-08-09) was searched with
BLAST (Camacho et al.) and HHblits (Steinegger et al.)
for evolutionary related structures matching the target sequence in Table T1. For details on the template search, see Materials and Methods. Overall 8180 templates were found (Table T2).

## Models

The following model was built (see Materials and Methods "Model Building"):

| Model #01 | File | Built with | Oligo-State | Ligands | GMQE | QMEANDisCo Global |
| --- | --- | --- | --- | --- | --- | --- |
|  | PDB | ProMod3 3.4.1 | monomer | None | 0.60 | 0.57 ± 0.05 |

|  |  |  |
| --- | --- | --- |
|  |  |  |

| Template | Seq Identity | Oligo-state | QSQE | Found by | Method | Resolution | Seq Similarity | Range | Coverage | Description |
| --- | --- | --- | --- | --- | --- | --- | --- | --- | --- | --- |
| 7v6c.1.A | 29.72 | monomer | 0.00 | HHblits | EM | 3.30Å | 0.35 | 9 - 1622 | 0.98 | Dicer-2, isoform A |

  

### The template contained no ligands.

  

```
Target    MDEEDELKPRNYQVNLMEIAIRENTIIYLPTGSGKTFIAIMVLKQLCAPILRPYSDGGKISVILVNSVALVDQHGKYVRD  
7v6c.1.A  -MEDVEIKPRGYQLRLVDHLTKSNGIVYLPTGSGKTFVAILVLKRFSQDFDKPIESGGKRALFMCNTVELARQQAMAVRR  
  
Target    HATFSVGTYTGEMNVDFWSEAEWEQQFNKYQVVIMTSQIMVNLINNRFIDLGKVNLMIFDECHHGVEDQPMRQIMKHFHS  
7v6c.1.A  CTNFKVGFYVGEQGVDDWTRGMWSDEIKKNQVLVGTAQVFLDMVTQTYVALSSLSVVIIDECHHGTGHHPFREFMRLFTI  
  
Target    C--TDKPRVLGLTATLLNGNCKLSKVMDEIRSLEVTFHSKVATVEGL---DVVVGYSTNPQELFKVCQPGALSLDAKQ-V  
7v6c.1.A  ANQTKLPRVVGLTGVLIKGNE-ITNVATKLKELEITYRGNIITVSDTKELENVMLYATKPTEVMVSFPHQEQVLTVTRLI  
  
Target    LNNLRQLINDLEHINIKDEQNSVNLLQSETLKPLEPSDVLKSLRNLISDLMIHIEMLGAFGGHIACVAHMIQIERIKKHC  
7v6c.1.A  SAEIEKFYVSLDLMNIGVQ----PIRRSKSLQCLRDPSKKSFVKQLFNDFLYQMKEYGIYAASIAIISLIVEFDIKRRQA  
  
Target    QNHQLFIVLNYVMTIMGTTKLLLEETMA---------GYEPLEKIRKFSSDKVLKVFEILDEYKTK-SDEELCCLVFTKR  
7v6c.1.A  ETLSVKLMHRTALTLCEKIRHLLVQKLQDMTYDDDDDNVNTEEVIMNFSTPKVQRFLMSLKVSFADKDPKDICCLVFVER  
  
Target    RFTAKVLHHIIDKASQVDPKF-YHIKSNFVVGNKNNPYNDTRENLYITKKNREVLNSFVSKEINVLVSSNVLEEGVDIPK  
7v6c.1.A  RYTCKCIYGLLLNYIQSTPELRNVLTPQFMVGRNNISP--DFESVLERKWQKSAIQQFRDGNANLMICSSVLEEGIDVQA  
  
Target    CTLVIKFDKSEDYRSYIQSKGRARHIKSLYYTIVETTDVAKYDKKYSAFKEIENLVNDLLIGKNSERDHPNLSEIRNMYN  
7v6c.1.A  CNHVFILDPVKTFNMYVQSKGRARTTEAKFVLFTADKEREKTIQQIYQYRKAHNDIAEYLKDRVLEKTEPELYEIKGHFQ  
  
Target    EDKLEPYYVNGPNSAQVNMTSAVALLCRYCSNLASDKYTTYAPEWYYEEDS---------SSAKLRVVIFLPVVCPLIDP  
7v6c.1.A  -DDIDP-F-TNENGAVLLPNNALAILHRYCQTIPTDAFGFVIPWFHVLQEDERDRIFGVSAKGKHVISINMPVNCMLRDT  
  
Target    IVGPYMHNKKDAKRAAALVACIKLHQCGELDNNLLPWKKQLDE-ADVSYLFTHWPQEKES-----DAGNKKKKRLHDKEI  
7v6c.1.A  IYSDPMDNVKTAKISAAFKACKVLYSLGELNERFVPKTLKERVASIADVHFEHWNKYGDSVTATVNKADKSKDRTYKTEC  
  
Target    APSVKSAI-QPDRVLYLHTININPQYKRSDDLKNAVTIYDLYKTPLKFGLLSPKPLPDLCKFPLFDSNGTLEIEIRNNV-  
7v6c.1.A  PLEFYDALPRVGEICYAYEIFLEPQFESC---EYTEHMYLNLQTPRNYAILLRNKLPRLAEMPLFSNQGKLHVRVANAPL  
  
Target    REVEFAANEMKEMREFHFLVFNDLLEILKEFLIFDNTGMNSEMLLVVPVQDRC-GDVCVDFRVIRDNKNLKNKLEPAATE  
7v6c.1.A  EVIIQNSEQLELLHQFHGMVFRDILKIWHPFFVLDRRS-KENSYLVVPLILGAGEQKCFDWELMTNFRRLPQSHGSNVQQ  
  
Target    R-INLNVTEETYLHKIVSPWYRSPPKMYVVTKVCPDKSALSRFPNH-EYPNFVSYYSEKHSLS---ILDPSQPLLLVKGL  
7v6c.1.A  REQQPAPRPEDFEGKIVTQWYANYDKPMLVTKVHRELTPLSYMEKNQQDKTYYEFTMSKYGNRIGDVVHKDKFMIEVRDL  
  
Target    SERLNAFKPRGAGGKRKKEKMYEELEEYLIPELVIKQEFPSCLWIQARFLPSILSRLAYLLKLQQLQVDIARGIGAKAEY  
7v6c.1.A  TEQLTFYVHNRGKFNA---KSKAKMKVILIPELCFNFNFPGDLWLKLIFLPSILNRMYFLLHAEALRKRFNTYLNLHLLP  
  
Target    L--K--DCPPLELNLHLLHYEPNDPQLTQESDKSTPLIDNCLALECPKNLRTIQYNKDFAAKMLEAEYYWKTIEEPKDIE  
7v6c.1.A  FNGTDYMPRPLEIDYSLKRNVDPLGNVIPTEDIEEPK---SLLEPMPT--KSIEA-SVANLEITEFENPWQKYMEPVDLS  
  
Target    RNIN-VTVMDIEYYETFISHQPSKTGR-LL-------------K------NDSPV-KQQNVPAITYDCQ-F--EAKQLQI  
7v6c.1.A  RNLLSTYPVELDYYYHFSVGNVCEMNEMDFEDKEYWAKNQFHMPTGNIYGNRTPAKTNANVPALMPSKPTVRGKVKPLLI  
  
Target    LDVQFD---NQSPNLCQIYQALTAAEANDIVNLERLETLGDSFLKFVASLYIIFKFPTYNEGKSTTLKGKLVSNKNLYYL  
7v6c.1.A  LQKTVSKEHITPAEQGEFLAAITASSAADVFDMERLEILGDSFLKLSATLYLASKYSDWNEGTLTEVKSKLVSNRNLLFC  
  
Target    GVRKNLGGILKNSDLSPS-DWVPPCFCIPQTISKAIGNKE-----YSVVSLFNCCISPEEQ-VSGNLNRKTLSDMTTEEI  
7v6c.1.A  LIDADIPKTLNTIQFTPRYTWLPPGISLPHNVLALWRENPEFAKIIGPHNLRDLALGDEESLVKGNCSDINYNRFVEGCR  
  
Target    APD-----EENSYGNMCNFLNKQYVGDKSIADSVEALLGAYFLSGGIQGGIKFMEWIGILPLSEQ--IQRLIETTQVDPV  
7v6c.1.A  ANGQSFYAGADFSSEVNFCVGLVTIPNKVIADTLEALLGVIVKNYGLQHAFKMLEYFKICRADIDKPLTQLLNLELGGKK  
  
Target    LNKKSTKTDVDFHMPQWREIEQRLGYTFTNRAFLLQALTHSSYSPNRITLSYERLEFLGDAVLDFLITCYIFEHCGHLEP  
7v6c.1.A  MRANVNTTEIDGFLINHYYLEKNLGYTFKDRRYLLQALTHPSYPTNRITGSYQELEFIGDAILDFLISAYIFENNTKMNP  
  
Target    GQVTDLRSSLVNNNTFASLVVRCGFHKFLLMMNSNLQGHIDKFADYLASKNYVIDDEVLI--------------------  
7v6c.1.A  GALTDLRSALVNNTTLACICVRHRLHFFILAENAKLSEIISKFVNFQESQGHRVTNYVRILLEEADVQPTPLDLDDELDM  
  
Target    ------------LLE-----EDEMNIAEYVDVPKVLGDIFEALAGAIYLDSNKDLKTVWRVFYKIIWREIDLFSKNVPKN  
7v6c.1.A  TELPHANKCISQEAEKGVPPKGEFNMSTNVDVPKALGDVLEALIAAVYLDCR-DLQRTWEVIFNLFEPELQEFTRKVPIN  
  
Target    VIRRLYECHTVYPPQFSKALEVGNQKTMVSLDFMCEGRKKRVHGFGTNKILAKRAAAKIALRALKL  
7v6c.1.A  HIRQLVEHKHA-KPVFSSPIVEGE-TVMVSCQFTCMEKTIKVYGFGSNKDQAKLSAAKHALQQLS-
```

  


---

  

## Materials and Methods

## Template Search

Template search with BLAST and HHblits
has been performed against the SWISS-MODEL template library (SMTL, last update: 2024-08-14, last included PDB release: 2024-08-09).

The target sequence was searched with BLAST against the primary amino acid sequence contained in the SMTL.
A total of 217 templates were found.

An initial HHblits profile has been built using the procedure outlined in (Steinegger et al.), followed by 1 iteration of HHblits against Uniclust30 (Mirdita, von den Driesch et al.). The obtained profile has then be searched against all profiles of the SMTL. A total of 8249 templates were found.

## Model Building

Models are built based on the target-template alignment using ProMod3 (Studer et al.). Coordinates which are conserved between the target and the template are copied from the template to the model. Insertions and deletions are remodelled using a fragment library. Side chains are then rebuilt. Finally, the geometry of the resulting model is regularized by using a force field.

## Model Quality Estimation

The global and per-residue model quality has been assessed using the QMEAN scoring function (Studer et al.).

## Ligand Modelling

Ligands present in the template structure are transferred by homology to the model when the following criteria are met: (a) The ligands are annotated as biologically relevant in the template library, (b) the ligand is in contact with the model, (c) the ligand is not clashing with the protein, (d) the residues in contact with the ligand are conserved between the target and the template. If any of these four criteria is not satisfied, a certain ligand will not be included in the model. The model summary includes information on why and which ligand has not been included.

## Oligomeric State Conservation

The quaternary structure annotation of the template is used to model the target sequence in its oligomeric form. The method (Bertoni et al.) is based on a supervised machine learning algorithm, Support Vector Machines (SVM), which combines interface conservation, structural clustering, and other template features to provide a quaternary structure quality estimate (QSQE). The QSQE score is a number between 0 and 1, reflecting the expected accuracy of the interchain contacts for a model built based a given alignment and template. Higher numbers indicate higher reliability. This complements the GMQE score which estimates the accuracy of the tertiary structure of the resulting model.

## References

- Camacho C, Coulouris G, Avagyan V, Ma N, Papadopoulos J, Bealer K, Madden TL

  BLAST+: architecture and applications.

  BMC Bioinformatics, 10, 421-430. (2009) 2000350010.1186/1471-2105-10-421
- Steinegger M, Meier M, Mirdita M, Vöhringer H, Haunsberger SJ, Söding J

  HH-suite3 for fast remote homology detection and deep protein annotation.

  BMC Bioinformatics 20, 473. (2019) 3152111010.1186/s12859-019-3019-7
- Mirdita M, von den Driesch L, Galiez C, Martin MJ, Söding J, Steinegger M

  Uniclust databases of clustered and deeply annotated protein sequences and alignments.

  Nucleic Acids Res, 45, D170–D176. (2016) 2789957410.1093/nar/gkw1081

## Table T1:

Primary amino acid sequence for which templates were searched and models were built.

MDEEDELKPRNYQVNLMEIAIRENTIIYLPTGSGKTFIAIMVLKQLCAPILRPYSDGGKISVILVNSVALVDQHGKYVRDHATFSVGTYTGEMNVDFWSE  
AEWEQQFNKYQVVIMTSQIMVNLINNRFIDLGKVNLMIFDECHHGVEDQPMRQIMKHFHSCTDKPRVLGLTATLLNGNCKLSKVMDEIRSLEVTFHSKVA  
TVEGLDVVVGYSTNPQELFKVCQPGALSLDAKQVLNNLRQLINDLEHINIKDEQNSVNLLQSETLKPLEPSDVLKSLRNLISDLMIHIEMLGAFGGHIAC  
VAHMIQIERIKKHCQNHQLFIVLNYVMTIMGTTKLLLEETMAGYEPLEKIRKFSSDKVLKVFEILDEYKTKSDEELCCLVFTKRRFTAKVLHHIIDKASQ  
VDPKFYHIKSNFVVGNKNNPYNDTRENLYITKKNREVLNSFVSKEINVLVSSNVLEEGVDIPKCTLVIKFDKSEDYRSYIQSKGRARHIKSLYYTIVETT  
DVAKYDKKYSAFKEIENLVNDLLIGKNSERDHPNLSEIRNMYNEDKLEPYYVNGPNSAQVNMTSAVALLCRYCSNLASDKYTTYAPEWYYEEDSSSAKLR  
VVIFLPVVCPLIDPIVGPYMHNKKDAKRAAALVACIKLHQCGELDNNLLPWKKQLDEADVSYLFTHWPQEKESDAGNKKKKRLHDKEIAPSVKSAIQPDR  
VLYLHTININPQYKRSDDLKNAVTIYDLYKTPLKFGLLSPKPLPDLCKFPLFDSNGTLEIEIRNNVREVEFAANEMKEMREFHFLVFNDLLEILKEFLIF  
DNTGMNSEMLLVVPVQDRCGDVCVDFRVIRDNKNLKNKLEPAATERINLNVTEETYLHKIVSPWYRSPPKMYVVTKVCPDKSALSRFPNHEYPNFVSYYS  
EKHSLSILDPSQPLLLVKGLSERLNAFKPRGAGGKRKKEKMYEELEEYLIPELVIKQEFPSCLWIQARFLPSILSRLAYLLKLQQLQVDIARGIGAKAEY  
LKDCPPLELNLHLLHYEPNDPQLTQESDKSTPLIDNCLALECPKNLRTIQYNKDFAAKMLEAEYYWKTIEEPKDIERNINVTVMDIEYYETFISHQPSKT  
GRLLKNDSPVKQQNVPAITYDCQFEAKQLQILDVQFDNQSPNLCQIYQALTAAEANDIVNLERLETLGDSFLKFVASLYIIFKFPTYNEGKSTTLKGKLV  
SNKNLYYLGVRKNLGGILKNSDLSPSDWVPPCFCIPQTISKAIGNKEYSVVSLFNCCISPEEQVSGNLNRKTLSDMTTEEIAPDEENSYGNMCNFLNKQY  
VGDKSIADSVEALLGAYFLSGGIQGGIKFMEWIGILPLSEQIQRLIETTQVDPVLNKKSTKTDVDFHMPQWREIEQRLGYTFTNRAFLLQALTHSSYSPN  
RITLSYERLEFLGDAVLDFLITCYIFEHCGHLEPGQVTDLRSSLVNNNTFASLVVRCGFHKFLLMMNSNLQGHIDKFADYLASKNYVIDDEVLILLEEDE  
MNIAEYVDVPKVLGDIFEALAGAIYLDSNKDLKTVWRVFYKIIWREIDLFSKNVPKNVIRRLYECHTVYPPQFSKALEVGNQKTMVSLDFMCEGRKKRVH  
GFGTNKILAKRAAAKIALRALKL

## Table T2:

| Template | Seq Identity | Oligo-state | QSQE | Found by | Method | Resolution | Seq Similarity | Coverage | Description |
| --- | --- | --- | --- | --- | --- | --- | --- | --- | --- |
| 7v6c.1.A | 29.72 | monomer | - | HHblits | EM | 3.30Å | 0.35 | 0.98 | Dicer-2, isoform A |
| 8hf0.1.A | 29.49 | homo-dimer | 0.23 | HHblits | EM | NA | 0.35 | 0.98 | Dicer-2, isoform A |
| 7xw3.1.A | 32.86 | monomer | - | HHblits | EM | NA | 0.36 | 0.95 | Endoribonuclease Dicer |
| 8hf0.1.D | 29.49 | homo-dimer | 0.23 | HHblits | EM | NA | 0.35 | 0.98 | Dicer-2, isoform A |
| 8dga.1.A | 29.40 | monomer | - | HHblits | EM | NA | 0.34 | 0.98 | Endoribonuclease Dcr-1 |
| 7v6b.1.A | 29.67 | monomer | - | HHblits | EM | 3.30Å | 0.35 | 0.98 | Dicer-2, isoform A |
| 7w0a.1.A | 29.49 | homo-dimer | 0.15 | HHblits | EM | NA | 0.35 | 0.98 | Dicer-2, isoform A |
| 8dfv.1.A | 29.40 | monomer | - | HHblits | EM | NA | 0.34 | 0.98 | Endoribonuclease Dcr-1 |
| 7zpj.1.A | 32.32 | monomer | - | HHblits | EM | NA | 0.36 | 0.95 | Endoribonuclease Dicer |
| 8dg7.1.A | 29.40 | monomer | - | HHblits | EM | NA | 0.34 | 0.98 | Endoribonuclease Dcr-1 |
| 8dg5.1.A | 29.40 | monomer | - | HHblits | EM | NA | 0.34 | 0.98 | Endoribonuclease Dcr-1 |
| 7w0f.1.A | 29.67 | monomer | - | HHblits | EM | NA | 0.35 | 0.98 | Dicer-2, isoform A |
| 8dgj.1.A | 29.40 | monomer | - | HHblits | EM | NA | 0.34 | 0.98 | Endoribonuclease Dcr-1 |
| 8dgi.1.A | 29.40 | monomer | - | HHblits | EM | NA | 0.34 | 0.98 | Endoribonuclease Dcr-1 |
| 7v6c.1.A | 33.35 | monomer | - | BLAST | EM | 3.30Å | 0.37 | 0.96 | Dicer-2, isoform A |
| 7yym.1.A | 32.19 | monomer | - | HHblits | EM | NA | 0.36 | 0.95 | Endoribonuclease Dicer |
| 7yz4.1.A | 32.19 | monomer | - | HHblits | EM | NA | 0.36 | 0.95 | Endoribonuclease Dicer |
| 7v6b.1.A | 33.35 | monomer | - | BLAST | EM | 3.30Å | 0.37 | 0.96 | Dicer-2, isoform A |
| 5zak.1.A | 32.86 | monomer | - | HHblits | EM | NA | 0.36 | 0.95 | Endoribonuclease Dicer |
| 5zam.1.A | 32.86 | monomer | - | HHblits | EM | NA | 0.36 | 0.95 | Endoribonuclease Dicer |
| 5zal.1.A | 32.86 | monomer | - | HHblits | EM | NA | 0.36 | 0.95 | Endoribonuclease Dicer |
| 8hf1.1.D | 33.16 | homo-trimer | 0.15 | BLAST | EM | NA | 0.37 | 0.96 | Dicer-2, isoform A |
| 8hf1.1.A | 33.16 | homo-trimer | 0.15 | BLAST | EM | NA | 0.37 | 0.96 | Dicer-2, isoform A |
| 8hf0.1.D | 33.16 | homo-dimer | 0.14 | BLAST | EM | NA | 0.37 | 0.96 | Dicer-2, isoform A |
| 8hf1.1.F | 33.16 | homo-trimer | 0.15 | BLAST | EM | NA | 0.37 | 0.96 | Dicer-2, isoform A |
| 7w0a.1.A | 33.16 | homo-dimer | 0.08 | BLAST | EM | NA | 0.37 | 0.96 | Dicer-2, isoform A |
| 7w0f.1.A | 33.29 | monomer | - | BLAST | EM | NA | 0.37 | 0.96 | Dicer-2, isoform A |
| 7zpi.1.A | 32.32 | monomer | - | HHblits | EM | NA | 0.36 | 0.95 | Endoribonuclease Dicer |
| 7xw2.1.A | 32.86 | monomer | - | HHblits | EM | NA | 0.36 | 0.95 | Endoribonuclease Dicer |
| 7yyn.1.B | 30.53 | monomer | - | HHblits | EM | NA | 0.35 | 0.82 | Isoform 2 of Endoribonuclease Dicer |
| 7eld.1.A | 26.06 | monomer | - | HHblits | EM | NA | 0.33 | 0.85 | Endoribonuclease Dicer homolog 1 |
| 7ele.1.A | 26.06 | monomer | - | HHblits | EM | NA | 0.33 | 0.85 | Endoribonuclease Dicer homolog 1 |
| 6bua.1.A | 29.55 | monomer | - | HHblits | EM | NA | 0.35 | 0.98 | Dicer-2, isoform A |
| 7xw3.1.A | 34.35 | monomer | - | BLAST | EM | NA | 0.38 | 0.59 | Endoribonuclease Dicer |
| 7zpj.1.A | 33.58 | monomer | - | BLAST | EM | NA | 0.37 | 0.59 | Endoribonuclease Dicer |
| 7yym.1.A | 33.58 | monomer | - | BLAST | EM | NA | 0.37 | 0.59 | Endoribonuclease Dicer |
| 7yz4.1.A | 33.58 | monomer | - | BLAST | EM | NA | 0.37 | 0.59 | Endoribonuclease Dicer |
| 5zam.1.A | 34.35 | monomer | - | BLAST | EM | NA | 0.38 | 0.59 | Endoribonuclease Dicer |
| 5zak.1.A | 34.35 | monomer | - | BLAST | EM | NA | 0.38 | 0.59 | Endoribonuclease Dicer |
| 5zal.1.A | 34.35 | monomer | - | BLAST | EM | NA | 0.38 | 0.59 | Endoribonuclease Dicer |
| 7vg2.1.A | 24.92 | monomer | - | HHblits | EM | NA | 0.33 | 0.80 | Dicer-like 3 |
| 7vg3.1.A | 24.92 | monomer | - | HHblits | EM | NA | 0.33 | 0.80 | Dicer-like 3 |
| 7zpi.1.A | 33.58 | monomer | - | BLAST | EM | NA | 0.37 | 0.59 | Endoribonuclease Dicer |
| 7xw2.1.A | 34.35 | monomer | - | BLAST | EM | NA | 0.38 | 0.59 | Endoribonuclease Dicer |
| 7yyn.1.B | 31.91 | monomer | - | BLAST | EM | NA | 0.36 | 0.43 | Isoform 2 of Endoribonuclease Dicer |
| 2qvw.1.A | 17.97 | monomer | - | HHblits | X-ray | 3.00Å | 0.28 | 0.39 | GLP\_546\_48378\_50642 |
| 2qvw.3.A | 17.97 | monomer | - | HHblits | X-ray | 3.00Å | 0.28 | 0.39 | GLP\_546\_48378\_50642 |
| 6bu9.1.A | 29.55 | monomer | - | HHblits | EM | NA | 0.35 | 0.98 | Dicer-2, isoform A |
| 3zd7.1.A | 28.41 | monomer | - | BLAST | X-ray | 2.50Å | 0.35 | 0.33 | PROBABLE ATP-DEPENDENT RNA HELICASE DDX58 |
| 2ykg.1.A | 28.22 | monomer | - | BLAST | X-ray | 2.50Å | 0.35 | 0.33 | PROBABLE ATP-DEPENDENT RNA HELICASE DDX58 |

  
The table above shows the top 50 filtered templates. A further 6,653 templates were found which were considered to be less suitable for modelling than the filtered list.  
1a1v.1.A, 1a5t.1.A, 1b7t.1.A, 1c4o.1.A, 1cr0.1.A, 1cu1.1.A, 1cu1.1.B, 1d0x.1.A, 1d2m.1.A, 1d9x.1.A, 1d9z.1.A, 1dfk.1.A, 1dfl.1.A, 1di2.1.C, 1di2.1.D, 1do0.1.A, 1do0.1.B, 1do0.1.C, 1do0.1.F, 1do2.1.C, 1do2.3.B, 1e0j.1.A, 1e0j.1.B, 1e0j.2.A, 1e1q.1.A, 1e1r.1.A, 1e1r.1.B, 1e1r.1.C, 1e32.1.B, 1e6c.1.A, 1e79.1.B, 1e94.3.D, 1efr.1.C, 1ekz.1.B, 1ffh.1.A, 1fnn.1.A, 1fuk.1.A, 1fuu.1.A, 1g3i.1.V, 1g41.1.C, 1g4a.1.H, 1g4a.1.M, 1g4b.1.B, 1g5r.1.A, 1g64.1.A, 1g6o.1.A, 1g8p.1.A, 1g8y.1.D, 1gki.1.D, 1gku.1.A, 1gl6.1.A, 1gl7.1.C, 1gl9.1.A, 1gm5.1.A, 1h8e.1.E, 1hei.1.A, 1hei.1.B, 1hqc.1.A, 1hqc.2.A, 1hv8.1.A, 1hv8.1.B, 1i4s.1.A, 1i4s.1.B, 1im2.1.F, 1in4.1.A, 1in5.1.A, 1in6.1.A, 1in7.1.A, 1in8.1.A, 1iqp.1.A, 1iqp.1.B, 1iqp.1.C, 1iqp.1.D, 1iqp.1.E, 1iqp.1.F, 1ixr.1.C, 1ixs.1.B, 1ixz.1.A, 1iy2.1.A, 1j7k.1.A, 1j8m.1.A, 1jah.1.A, 1jb1.1.F, 1jbk.1.A, 1jfz.1.A, 1jfz.1.B, 1jfz.2.A, 1jfz.2.B, 1jpj.1.A, 1jpn.1.A, 1jpn.2.A, 1jr3.1.A, 1jr3.1.B, 1jr3.1.C, 1jr3.1.E, 1jr6.1.A, 1kag.1.A, 1kag.2.A, 1kao.1.A, 1kdo.1.A, 1kdo.2.A, 1kdp.2.A, 1kdr.1.A, 1kdr.2.A, 1kdt.1.A, 1kdt.2.A, 1ki9.2.A, 1ki9.2.E, 1ki9.2.F, 1kk7.1.A, 1kk8.1.A, 1kkl.1.C, 1kkl.1.H, 1ko4.1.A, 1ko4.1.B, 1ko5.1.B, 1ko8.1.A, 1ko8.1.B, 1kqm.1.A, 1ksf.1.A, 1kwo.1.A, 1kyi.1.B, 1kyi.1.D, 1kyi.1.U, 1l2o.1.A, 1l8q.1.A, 1ls1.1.A, 1lv7.1.A, 1m6n.1.A, 1m8q.1.J, 1mab.1.A, 1mmd.1.A, 1mmg.1.A, 1mmn.1.A, 1mnd.1.A, 1mvw.1.A, 1mvw.1.D, 1mvw.1.G, 1mvw.1.J, 1mvw.1.M, 1mvw.1.P, 1n0w.1.A, 1n25.1.A, 1nbm.1.D, 1nbm.1.E, 1njf.1.A, 1njf.2.A, 1njf.3.A, 1njf.4.A, 1njg.1.A, 1njg.2.A, 1nks.1.A, 1nks.1.B, 1nks.1.C, 1nkt.1.A, 1nl3.1.B, 1nlf.1.B, 1nlz.1.A, 1nlz.1.B, 1nlz.1.C, 1nlz.1.F, 1np6.1.A, 1np6.1.B, 1nsf.1.A, 1ny6.1.A, 1ny6.1.B, 1ny6.1.C, 1ny6.1.E, 1ny6.1.F, 1ny6.1.G, 1ny6.2.A, 1ny6.2.B, 1ny6.2.E, 1ny6.2.F, 1ny6.2.G, 1o0w.1.A, 1o18.1.K, 1o18.1.N, 1o19.1.M, 1o19.1.P, 1o1a.1.M, 1o1a.1.P, 1o1b.1.G, 1o1b.1.J, 1o1c.1.M, 1o1d.1.M, 1o1d.1.P, 1o1e.1.M, 1o1f.1.D, 1o1g.1.M, 1o1g.1.P, 1o87.1.A, 1o87.2.A, 1oe9.1.A, 1ofh.1.A, 1ofh.2.A, 1ofh.2.B, 1ofi.1.A, 1ohh.1.C, 1ojl.1.A, 1ojl.1.C, 1ojl.2.A, 1ojl.2.B, 1okk.1.A, 1olo.1.A, 1olo.1.D, 1onb.1.A, 1oyw.1.A, 1p9n.1.A, 1p9n.1.B, 1p9r.1.A, 1pv4.1.A, 1pv4.1.B, 1pv4.1.C, 1pv4.1.D, 1pv4.1.E, 1pv4.1.F, 1pvo.1.C, 1pzn.1.A, 1pzn.1.D, 1q0u.1.A, 1q0u.2.A, 1q3t.1.A, 1q57.1.A, 1q57.1.B, 1q57.1.C, 1q57.1.D, 1q57.1.E, 1q57.1.F, 1q57.1.G, 1qde.1.A, 1qhg.1.A, 1qhh.1.A, 1qu6.1.A, 1qva.1.A, 1qvi.1.A, 1qvr.1.A, 1qvr.1.B, 1qvr.1.C, 1r4k.1.A, 1r6b.1.A, 1r7r.1.A, 1rc7.1.E, 1rif.1.A, 1rif.2.A, 1rj9.1.B, 1rkb.1.A, 1rz3.1.A, 1s2m.1.A, 1s3s.1.A, 1s3s.1.B, 1s3s.1.C, 1s3s.1.D, 1s3s.1.E, 1s3s.1.F, 1s9h.1.A, 1s9h.2.A, 1s9h.3.A, 1si3.1.A, 1sky.1.B, 1sr6.1.A, 1stu.1.A, 1svl.1.A, 1svl.1.B, 1svl.1.F, 1svm.1.A, 1svm.1.B, 1svo.1.A, 1sxj.1.A, 1sxj.1.B, 1sxj.1.C, 1sxj.1.D, 1sxj.1.E, 1szp.1.A, 1szp.1.B, 1t0h.1.B, 1t2r.1.A, 1t2s.1.A, 1t4l.1.B, 1t4n.1.A, 1t4o.1.A, 1t4o.2.A, 1t5i.1.A, 1t5l.1.A, 1t5l.2.A, 1t6n.1.A, 1t6n.1.B, 1tf2.1.A, 1tf7.1.A, 1tf7.1.F, 1tue.1.A, 1tue.2.A, 1tue.5.A, 1u0j.1.A, 1u61.1.A, 1uaa.1.B, 1uaa.1.C, 1uei.1.A, 1ufq.1.A, 1ufq.1.B, 1uhz.1.A, 1uil.1.A, 1uj2.1.C, 1um8.1.A, 1v5w.1.A, 1vec.1.A, 1vec.2.A, 1vg1.1.A, 1vg9.2.B, 1vma.1.A, 1vom.1.A, 1w2h.1.A, 1w2h.1.B, 1w36.1.C, 1w4r.1.A, 1w4r.1.B, 1w5s.1.A, 1w5s.2.A, 1w5t.1.A, 1w5t.3.A, 1w7j.1.A, 1w8j.1.A, 1w8j.3.A, 1w8j.4.A, 1w9l.1.A, 1whn.1.A, 1whq.1.A, 1wp9.1.A, 1wp9.2.A, 1wrb.1.A, 1wrb.2.A, 1x47.1.A, 1x48.1.A, 1x49.1.A, 1xbt.1.A, 1xew.1.A, 1xex.1.C, 1xfa.1.A, 1xjc.1.A, 1xpo.1.A, 1xpo.2.A, 1xpo.3.A, 1xpo.4.A, 1xpo.5.A, 1xpo.6.A, 1xpr.1.A, 1xpr.2.A, 1xpr.3.A, 1xpr.4.A, 1xpr.5.A, 1xpr.6.A, 1xpu.1.A, 1xrj.1.A, 1xti.1.A, 1xtj.1.A, 1xtk.1.A, 1xu4.1.A, 1xwi.1.A, 1xx6.1.A, 1xxh.1.B, 1xxh.1.C, 1xxh.1.D, 1xxh.1.E, 1xxh.2.B, 1xxh.2.C, 1xxh.2.D, 1xxi.1.B, 1xxi.1.C, 1xxi.1.D, 1xxi.1.E, 1xxi.2.B, 1y63.1.A, 1ye8.1.A, 1yyf.1.B, 1yyf.1.U, 1yyk.1.E, 1yyk.1.F, 1yyo.1.E, 1yyo.1.F, 1yyw.1.E, 1yyw.1.F, 1yyw.2.E, 1yyw.2.F, 1yz9.1.E, 1yz9.1.F, 1yzk.1.A, 1z3i.1.A, 1z5z.1.A, 1z5z.2.A, 1z63.1.C, 1z6a.3.A, 1z6g.1.A, 1z6t.1.A, 1ztd.1.A, 1ztd.1.B, 1zu4.1.A, 1zu5.1.A, 2a11.1.A, 2a2z.1.A, 2b21.1.A, 2b7t.1.A, 2b7v.1.A, 2b8t.1.A, 2b8t.1.C, 2b8t.1.D, 2bhr.1.A, 2bjw.1.A, 2bkh.1.A, 2bki.1.A, 2bmf.1.A, 2bmf.2.A, 2c03.1.A, 2c03.2.A, 2c04.1.A, 2c04.2.A, 2c9c.1.A, 2c9o.1.D, 2c9o.2.A, 2c9o.2.F, 2ce7.1.A, 2ce7.1.B, 2ce7.1.C, 2ce7.2.A, 2ce7.2.B, 2ce7.2.C, 2chg.1.A, 2chq.1.A, 2chq.1.B, 2chq.1.C, 2chv.1.C, 2chv.1.E, 2ck3.1.B, 2ck3.1.C, 2cmk.1.A, 2cnw.1.A, 2cnw.3.A, 2cpn.1.A, 2cvf.1.A, 2cvf.2.A, 2cvh.1.A, 2cvh.2.A, 2d7c.1.A, 2d7c.2.A, 2d7d.1.A, 2db2.1.A, 2db3.1.B, 2dfl.1.A, 2dhr.1.A, 2dhr.1.B, 2dix.1.A, 2dmy.1.A, 2dr3.1.A, 2dr3.1.B, 2dr3.1.C, 2dr3.1.D, 2dr3.1.E, 2dr3.1.F, 2dyk.1.A, 2eb1.1.A, 2eb1.1.B, 2ec6.1.A, 2eww.1.A, 2eyq.1.A, 2eyq.2.A, 2eyu.1.A, 2eyu.2.A, 2ez6.1.C, 2f1h.1.A, 2f1i.1.A, 2f1r.1.A, 2f1r.1.B, 2f43.1.A, 2f43.1.F, 2f55.1.B, 2f55.1.C, 2f55.2.A, 2fdc.1.B, 2fdc.2.B, 2ffh.1.A, 2fna.1.A, 2fsh.1.A, 2fsh.1.B, 2fwr.1.A, 2fwr.2.A, 2fwr.3.A, 2fwr.4.A, 2fz4.1.A, 2fzl.1.A, 2g9n.1.A, 2g9n.2.A, 2ga8.1.A, 2gbl.1.B, 2gbl.1.E, 2gbl.1.F, 2gdj.1.A, 2gjk.1.A, 2gk6.1.A, 2gk7.1.A, 2gno.1.A, 2gsl.1.A, 2gsl.2.A, 2gsl.2.B, 2gsl.3.A, 2gsl.3.B, 2gsz.1.A, 2gsz.1.B, 2gxa.1.B, 2gxa.1.C, 2gxa.1.F, 2gxa.1.G, 2gxa.2.D, 2gxa.2.G, 2gxq.1.A, 2gxs.1.A, 2gxs.1.B, 2gza.1.A, 2gza.1.C, 2h1l.1.A, 2h1l.1.B, 2h1l.1.C, 2hcb.1.A, 2hcb.1.B, 2hcb.1.C, 2hcb.1.D, 2hf9.1.A, 2hjv.1.A, 2hjv.2.A, 2ht1.1.A, 2ht1.1.B, 2hxy.1.A, 2hxy.3.A, 2hyi.1.C, 2i3b.1.A, 2i4i.1.A, 2ibm.3.C, 2ibm.3.D, 2ipc.1.A, 2ipc.1.B, 2is1.1.C, 2is1.2.C, 2is2.1.C, 2is4.1.C, 2is4.1.D, 2is6.1.D, 2ius.1.A, 2iut.1.A, 2iuu.1.B, 2iyq.1.A, 2iyr.1.A, 2iyr.2.A, 2iys.1.A, 2iyv.1.A, 2iyx.1.A, 2j0q.1.A, 2j0u.1.A, 2j0u.2.A, 2j28.1.H, 2j37.1.G, 2j41.1.A, 2j41.1.B, 2j45.1.A, 2j45.2.A, 2j46.1.A, 2j46.2.A, 2j7p.2.A, 2j9r.1.A, 2ja1.1.A, 2jeo.1.A, 2jgn.1.A, 2jgn.2.A, 2jiz.1.A, 2jj1.1.B, 2jj2.2.C, 2jlq.1.A, 2jlr.1.A, 2jls.1.A, 2jlu.1.A, 2jlv.2.A, 2jly.1.A, 2kbe.1.A, 2kbf.1.A, 2khx.1.A, 2kjq.1.A, 2kou.1.A, 2l2k.1.A, 2l2m.1.A, 2l2n.1.A, 2l33.1.A, 2l3c.1.A, 2l3j.1.A, 2l5c.1.A, 2l5d.1.A, 2l8b.1.A, 2lbs.1.B, 2ljh.1.A, 2lrs.1.A, 2ltr.1.A, 2lts.1.A, 2lup.1.B, 2luq.1.A, 2mdr.1.A, 2mys.1.A, 2n3f.1.A, 2n3g.1.A, 2n3h.1.A, 2na2.1.A, 2ng1.1.A, 2nq2.1.B, 2nq2.1.D, 2nue.1.B, 2nue.1.C, 2nuf.1.C, 2nuf.1.D, 2nug.1.E, 2o0j.1.A, 2o0k.1.A, 2oap.1.A, 2oap.1.B, 2oca.1.A, 2og2.1.A, 2oxc.1.A, 2p65.1.A, 2p6n.1.A, 2p6r.1.C, 2p6u.1.A, 2pey.1.A, 2pey.1.B, 2pjr.1.A, 2pjr.1.C, 2pl3.1.A, 2pt7.1.B, 2pt7.1.D, 2q6t.1.A, 2q6t.2.A, 2q6t.3.A, 2q6t.4.A, 2qa5.1.A, 2qag.1.A, 2qag.1.C, 2qag.1.E, 2qby.1.C, 2qby.1.D, 2qe7.1.D, 2qeq.1.A, 2qeq.2.A, 2qgn.1.A, 2qgz.1.A, 2qnr.1.A, 2qor.1.A, 2qp9.1.A, 2qpa.1.A, 2qpa.2.A, 2qpa.3.A, 2qvw.1.A, 2qvw.3.A, 2qy9.1.A, 2qz4.1.A, 2r2a.1.A, 2r44.1.A, 2r62.1.A, 2r62.2.A, 2r65.1.A, 2r65.2.A, 2r65.3.A, 2r65.4.A, 2r65.5.A, 2r6a.1.A, 2r6a.1.B, 2r6c.1.A, 2r6c.1.B, 2r6c.1.C, 2r6c.1.D, 2r6c.1.E, 2r6c.1.F, 2r6d.1.A, 2r6d.1.B, 2r6d.1.C, 2r6d.1.D, 2r6d.1.E, 2r6d.1.F, 2r6e.1.A, 2r6e.1.F, 2r8r.1.A, 2r8r.2.A, 2rap.1.A, 2rb4.1.A, 2rko.1.A, 2rs6.1.A, 2rs7.1.A, 2shk.1.A, 2shk.1.B, 2uz3.1.A, 2v1u.1.A, 2v1x.1.A, 2v26.1.A, 2v3c.1.B, 2v3c.2.B, 2v6i.1.A, 2v6j.1.A, 2v7q.1.A, 2v7q.1.B, 2v7q.1.C, 2v7q.1.E, 2v8o.1.A, 2v9p.1.A, 2v9p.1.D, 2v9p.2.E, 2v9p.2.F, 2va8.1.A, 2va8.2.A, 2vas.1.A, 2vb6.1.A, 2vbc.1.A, 2vda.1.A, 2vii.1.A, 2vl7.1.A, 2vsf.1.A, 2vso.1.A, 2w00.2.A, 2w0m.1.A, 2w4a.3.A, 2w4g.1.C, 2w4h.1.C, 2w4t.1.A, 2w4v.1.A, 2w4w.1.A, 2w58.1.A, 2w58.1.B, 2w6e.1.A, 2w6e.1.B, 2w6e.1.C, 2w6e.1.D, 2w6e.1.E, 2w6j.1.A, 2w6j.1.B, 2w6j.1.C, 2w6j.1.F, 2wax.1.A, 2wax.2.A, 2way.1.A, 2whx.1.A, 2wjv.1.A, 2wjy.1.A, 2wpd.1.B, 2wsm.1.A, 2wss.1.A, 2wss.1.C, 2wss.2.B, 2wv9.1.A, 2wvj.1.A, 2www.2.B, 2wwy.1.B, 2wzq.1.A, 2x31.1.G, 2x31.1.H, 2x51.1.A, 2x8a.1.A, 2xau.1.A, 2xb2.2.A, 2xb4.1.A, 2xfm.1.A, 2xgj.1.A, 2xgj.2.A, 2xnd.1.A, 2xnd.1.B, 2xnd.1.C, 2xnd.1.D, 2xnd.1.E, 2xnd.1.F, 2xok.1.A, 2xok.1.B, 2xok.1.D, 2xok.1.E, 2xok.1.F, 2xsz.1.A, 2xsz.1.D, 2xxa.1.A, 2xxa.2.A, 2xzl.1.A, 2xzo.1.A, 2xzp.1.A, 2y0r.1.A, 2y8i.1.A, 2yjt.1.D, 2ykg.1.A, 2yt4.1.A, 2z0m.1.A, 2z43.1.A, 2z43.2.A, 2z4r.1.A, 2z83.1.A, 2zam.1.A, 2zan.1.A, 2zao.1.A, 2ze5.1.A, 2zj5.2.B, 2zj8.2.A, 2zjb.1.A, 2zjo.1.A, 2zm5.1.A, 2zm5.2.A, 2zpa.1.A, 2zpa.2.A, 2zts.1.A, 2zu6.1.A, 2zu6.1.C, 2zu6.2.A, 2zu6.2.C, 2zub.1.A, 2zub.1.B, 2zuc.1.A, 2zuc.1.B, 2zud.1.A, 2zud.1.B, 3a8t.1.A, 3adg.1.A, 3adi.1.A, 3adi.2.A, 3adi.3.A, 3adj.1.A, 3adk.1.A, 3adl.1.A, 3b6e.1.A, 3b85.1.A, 3b9p.1.A, 3b9q.1.A, 3be4.1.A, 3ber.1.A, 3bgw.1.A, 3bgw.1.B, 3bgw.1.C, 3bgw.1.D, 3bgw.1.E, 3bgw.1.F, 3bh0.1.A, 3bk7.1.A, 3bor.1.A, 3bos.1.A, 3bos.1.B, 3bs4.1.A, 3bxz.1.A, 3c4b.1.A, 3c4t.1.A, 3cbq.1.A, 3cf0.1.A, 3cf1.1.C, 3cf1.1.D, 3cf2.2.D, 3cf3.1.A, 3cf3.1.F, 3co5.1.A, 3cpe.1.A, 3cr7.1.A, 3cr7.1.B, 3crm.1.A, 3crv.1.A, 3crw.1.A, 3d3q.1.A, 3d3q.2.A, 3d6t.1.A, 3d8b.1.A, 3d8b.1.B, 3din.1.A, 3dkp.1.A, 3dl8.1.A, 3dm5.1.A, 3dm9.1.C, 3dmd.1.D, 3dmd.3.A, 3e1s.1.A, 3e2i.1.A, 3e70.1.A, 3eaq.1.A, 3eaq.1.B, 3ear.1.A, 3eas.1.A, 3eas.1.B, 3ec2.1.A, 3ecc.1.A, 3eie.1.A, 3eih.1.A, 3eih.2.A, 3eih.3.A, 3eiq.1.A, 3eiq.1.C, 3eph.1.A, 3epl.1.A, 3epl.1.C, 3etl.1.A, 3ews.1.A, 3ews.2.A, 3ex7.2.C, 3exa.1.A, 3exa.1.B, 3fe2.1.A, 3fhc.1.B, 3fho.1.A, 3fht.1.A, 3fks.1.C, 3fks.1.D, 3fks.1.E, 3fks.1.F, 3fks.2.B, 3fks.2.D, 3fks.3.A, 3fks.3.D, 3fmo.1.B, 3fmp.1.B, 3foz.1.B, 3ftq.1.A, 3ftq.2.A, 3ftq.3.A, 3ftq.4.A, 3g0h.1.A, 3gfp.1.A, 3glf.1.B, 3glf.1.C, 3glf.1.D, 3glg.1.B, 3glg.1.C, 3glg.1.D, 3glh.1.B, 3glh.1.C, 3glh.1.D, 3glh.2.B, 3glh.3.B, 3gli.1.B, 3gli.1.E, 3gp8.1.A, 3gpl.1.A, 3h1t.1.A, 3h4m.1.A, 3h4m.2.A, 3h4m.3.A, 3h86.1.A, 3h86.1.B, 3h86.1.C, 3h86.2.A, 3hgq.1.A, 3hte.1.A, 3hte.1.B, 3hte.1.C, 3hte.1.D, 3hu1.1.A, 3hu2.1.A, 3hu3.1.F, 3hws.1.C, 3hws.1.F, 3hws.2.B, 3hws.3.A, 3hws.3.C, 3i32.1.A, 3i5f.1.A, 3i5g.1.A, 3i62.1.A, 3ice.1.A, 3ice.1.B, 3ice.1.C, 3ice.1.D, 3ice.1.E, 3ice.1.F, 3iij.1.A, 3io5.1.A, 3iqm.1.A, 3iqy.1.A, 3iuy.1.A, 3j3r.1.G, 3j3r.1.H, 3j3r.1.I, 3j3r.1.J, 3j3r.1.K, 3j3r.1.L, 3j3s.1.G, 3j3s.1.H, 3j3s.1.I, 3j3s.1.J, 3j3s.1.K, 3j3s.1.L, 3j3t.1.G, 3j3t.1.H, 3j3t.1.I, 3j3t.1.J, 3j3t.1.K, 3j3t.1.L, 3j3u.1.G, 3j3u.1.H, 3j3u.1.I, 3j3u.1.J, 3j3u.1.K, 3j3u.1.L, 3j67.1.A, 3j6b.1.5, 3j6b.1.9, 3j94.1.A, 3j94.1.B, 3j94.1.C, 3j94.1.D, 3j94.1.E, 3j94.1.F, 3j95.1.A, 3j95.1.B, 3j95.1.C, 3j95.1.D, 3j95.1.E, 3j95.1.F, 3j96.1.A, 3j96.1.E, 3j96.1.F, 3j97.1.A, 3j97.1.B, 3j97.1.C, 3j97.1.D, 3j97.1.E, 3j97.1.F, 3j98.1.A, 3j98.1.B, 3j98.1.D, 3j98.1.E, 3j98.1.F, 3j99.1.A, 3j99.1.D, 3j99.1.E, 3j99.1.F, 3j9m.35.A, 3jaj.45.A, 3jan.45.A, 3jb9.1.5, 3jbl.1.A, 3jc8.36.A, 3jc8.37.A, 3jcm.1.I, 3jco.1.Q, 3jco.1.R, 3jco.1.S, 3jco.1.T, 3jco.1.U, 3jco.1.V, 3jcp.1.Q, 3jcp.1.R, 3jcp.1.S, 3jcp.1.T, 3jcp.1.U, 3jcp.1.V, 3jux.1.A, 3jv2.1.A, 3jv2.2.A, 3jvu.1.A, 3jvu.1.B, 3jvu.1.C, 3jvv.1.A, 3jvv.1.B, 3jvv.1.C, 3jzm.1.A, 3jzm.1.F, 3k09.1.A, 3k09.1.B, 3k09.1.E, 3k09.1.F, 3k0a.1.A, 3k0a.1.B, 3k0a.1.C, 3k0a.1.F, 3k0e.1.A, 3k0e.1.B, 3k0e.1.C, 3k0e.1.D, 3k0e.1.E, 3k0e.1.F, 3k0f.1.A, 3k0f.1.B, 3k0f.1.E, 3k0f.1.F, 3k1j.1.A, 3k1j.1.B, 3k70.1.A, 3k70.2.A, 3k70.2.C, 3kds.1.A, 3kqh.1.A, 3kql.1.A, 3kqu.1.B, 3kta.1.A, 3kta.2.A, 3kx2.2.A, 3l0o.1.A, 3l0o.1.B, 3l0p.1.A, 3l9i.1.A, 3ld9.1.A, 3lda.1.A, 3lfu.1.A, 3llh.1.A, 3llh.2.A, 3llm.1.A, 3llm.1.B, 3lnc.1.A, 3ly5.1.A, 3m0e.1.A, 3m6a.1.A, 3m6a.1.D, 3m6a.1.E, 3m6a.1.F, 3mj0.1.A, 3mkd.1.A, 3mwj.1.A, 3mwj.1.B, 3mwy.1.A, 3n3w.1.A, 3n70.1.A, 3n70.2.A, 3n70.2.B, 3n70.3.A, 3n70.4.A, 3n70.5.A, 3n70.5.B, 3n70.7.A, 3nbf.1.A, 3nbf.2.A, 3nbx.1.A, 3ndb.1.B, 3ney.1.A, 3ney.2.A, 3ng1.1.A, 3ntu.1.A, 3o2r.1.A, 3o2r.2.B, 3o6e.1.C, 3o7v.1.A, 3o7x.1.A, 3o7x.2.B, 3o8b.1.A, 3o8b.2.A, 3o8c.1.A, 3o8d.1.B, 3o8r.1.B, 3oaa.2.A, 3oaa.4.B, 3oaa.4.C, 3oe7.1.D, 3oee.1.A, 3oee.1.B, 3oee.1.C, 3oee.3.F, 3oeh.1.B, 3oeh.1.D, 3oeh.1.E, 3oeh.2.B, 3oeh.2.F, 3oeh.3.C, 3ofn.1.A, 3ofn.1.B, 3ofn.2.F, 3ofn.3.C, 3oiy.1.A, 3oiy.2.A, 3p1x.1.A, 3p4x.1.A, 3p4x.2.A, 3p4y.1.A, 3peu.1.A, 3pew.1.A, 3pey.1.A, 3pfi.1.A, 3pfi.1.B, 3pjr.1.C, 3pvs.1.A, 3pvs.1.B, 3pvs.1.C, 3pvs.1.D, 3pxg.1.B, 3pxg.1.D, 3pxg.1.F, 3pxg.1.J, 3pxi.1.B, 3pxi.1.D, 3pxi.1.F, 3qir.1.A, 3qir.2.B, 3qir.3.A, 3qkr.1.A, 3qks.1.A, 3r8f.1.A, 3rap.1.A, 3rap.1.B, 3rc3.1.A, 3rc8.1.A, 3rrm.1.A, 3rrn.1.A, 3rv0.1.A, 3rv0.1.B, 3rv0.2.A, 3rv0.2.B, 3rv1.1.A, 3rv1.1.B, 3rvb.1.A, 3sc3.1.A, 3sc3.1.B, 3sfz.1.A, 3sop.1.A, 3sqw.1.A, 3sqx.1.A, 3syk.1.A, 3syk.1.B, 3t15.1.A, 3t5d.1.A, 3tau.1.A, 3tau.1.B, 3tbk.1.A, 3te6.1.A, 3te6.2.A, 3tlx.1.A, 3tlx.3.A, 3tqf.1.A, 3trf.1.A, 3tw4.1.A, 3u44.1.A, 3u4q.1.B, 3u5z.1.A, 3u5z.1.D, 3u5z.2.A, 3u5z.2.B, 3u60.1.A, 3u60.1.B, 3u60.1.C, 3u60.1.D, 3u61.1.A, 3u61.1.B, 3u61.1.C, 3u61.1.D, 3ua2.1.A, 3ua2.1.B, 3ua2.1.C, 3ua2.1.D, 3ua2.1.E, 3ua2.1.F, 3uk6.1.A, 3upu.1.A, 3upu.3.A, 3uwx.1.B, 3v4r.1.A, 3v70.1.A, 3vfd.1.A, 3vkw.1.A, 3vyx.1.A, 3vyy.1.A, 3vyy.2.A, 3w8r.1.A, 3whk.1.A, 3zd6.1.A, 3zd7.1.A, 3zia.2.C, 3zuh.1.A, 3zw6.1.A, 3zw6.1.B, 3zw6.1.F, 4a15.1.A, 4a1f.1.A, 4a1f.1.B, 4a2p.1.A, 4a2q.1.A, 4a2q.3.A, 4a2w.1.A, 4a2w.2.A, 4a36.2.A, 4a4d.1.A, 4a4z.1.A, 4a7f.1.C, 4a7h.1.C, 4a7l.1.C, 4a92.1.A, 4ad8.1.A, 4ag5.2.B, 4ag6.1.A, 4ag6.1.B, 4ag6.2.B, 4ai6.1.A, 4ai6.1.B, 4ak9.1.A, 4akg.1.A, 4akg.1.B, 4akh.1.A, 4akh.2.A, 4aki.1.B, 4asu.1.A, 4asu.1.C, 4asu.1.D, 4asu.1.E, 4asu.1.F, 4ay2.1.A, 4b2i.1.A, 4b2p.1.A, 4b2q.1.2, 4b2q.1.B, 4b2q.1.C, 4b2q.1.D, 4b2q.1.E, 4b3c.1.A, 4b3d.2.A, 4b3f.1.A, 4b3g.1.A, 4b3g.2.A, 4b6e.1.A, 4b75.1.A, 4be7.1.A, 4beb.1.A, 4bec.1.A, 4bgd.1.A, 4bij.1.A, 4bil.1.A, 4bpb.1.A, 4bru.1.A, 4brw.1.A, 4bs1.1.B, 4buj.1.A, 4buj.2.A, 4byf.1.A, 4bzp.1.A, 4c0h.1.A, 4c2t.1.A, 4c2t.2.A, 4c2u.1.A, 4c30.1.A, 4c30.2.B, 4c7o.1.A, 4c7o.2.A, 4c9b.1.A, 4cbg.1.A, 4cbg.3.A, 4cbh.4.A, 4cbi.2.A, 4cbi.4.A, 4cbl.1.A, 4cbl.3.A, 4cbm.1.A, 4cbm.3.A, 4cbm.4.A, 4cdg.1.A, 4ce4.1.3, 4ceh.1.A, 4cei.1.A, 4cej.1.A, 4cej.1.B, 4cgz.1.A, 4ciu.1.A, 4cr2.1.O, 4cr2.1.P, 4cr2.1.Q, 4cr2.1.R, 4cr2.1.S, 4cr2.1.T, 4cr4.1.O, 4cr4.1.P, 4cr4.1.Q, 4cr4.1.R, 4cr4.1.S, 4cr4.1.T, 4crw.1.B, 4ct4.1.B, 4ct4.2.B, 4ct5.1.A, 4d25.1.A, 4d2i.1.A, 4d2i.1.B, 4d2q.1.A, 4d2u.1.A, 4d2x.1.C, 4d6p.1.A, 4d80.1.A, 4d80.1.B, 4d80.1.C, 4d80.1.D, 4d80.1.F, 4d81.1.A, 4d82.1.A, 4db1.1.A, 4db1.2.A, 4db2.1.A, 4db4.1.A, 4dbp.1.A, 4dbq.1.A, 4dbr.1.A, 4dc9.2.D, 4ddt.1.A, 4ddu.1.A, 4ddv.1.A, 4ddw.1.A, 4ddx.1.A, 4dkk.1.A, 4e2i.1.A, 4e7s.1.A, 4e7s.2.A, 4e7z.1.A, 4eiw.1.A, 4eiw.1.B, 4ern.1.A, 4esv.1.B, 4esv.1.C, 4esv.1.D, 4esv.1.E, 4esv.1.F, 4esv.1.G, 4esv.2.B, 4esv.2.C, 4esv.2.D, 4esv.2.E, 4esv.2.F, 4esv.2.G, 4eun.1.A, 4f3t.1.A, 4f91.1.A, 4f92.1.A, 4f93.1.A, 4fct.1.A, 4fcv.1.A, 4fcw.1.A, 4fcw.2.A, 4fcw.3.A, 4fd2.1.A, 4gdf.1.A, 4gdf.1.B, 4gl2.1.A, 4gl2.2.A, 4hse.1.A, 4hut.1.A, 4hut.1.B, 4hyy.1.A, 4i1s.1.A, 4i34.1.A, 4i34.1.B, 4i34.1.C, 4i34.1.D, 4i4l.1.D, 4i4l.1.E, 4i4l.1.F, 4i5o.1.A, 4i5o.1.C, 4i5o.1.E, 4i63.1.C, 4i63.1.F, 4i81.1.B, 4i81.1.C, 4i81.1.D, 4i81.1.E, 4i81.1.F, 4iee.1.A, 4ife.1.A, 4ihq.1.A, 4ihq.1.B, 4ihq.1.C, 4ii7.1.A, 4ii7.1.B, 4ii7.2.B, 4ijm.1.A, 4ijm.1.C, 4ijm.1.F, 4kbf.1.A, 4kbf.2.A, 4kbg.2.A, 4kfs.1.A, 4kfs.2.A, 4kft.3.A, 4kft.4.A, 4kit.1.A, 4kln.1.A, 4kod.1.A, 4kod.2.E, 4kre.1.A, 4ksr.1.A, 4ksr.1.B, 4ksr.1.C, 4kss.1.A, 4kss.1.B, 4kss.1.C, 4kss.1.D, 4kss.1.E, 4kss.1.F, 4kv9.1.A, 4kva.1.B, 4kxf.1.A, 4kxf.2.A, 4kxf.3.A, 4kxf.4.A, 4kxf.5.A, 4kxf.6.A, 4kxf.7.A, 4kxf.8.A, 4kxt.1.A, 4l15.1.A, 4l16.1.A, 4lcb.1.A, 4lgm.1.A, 4lj5.1.A, 4lj6.1.A, 4lj7.1.A, 4lj7.2.A, 4lj7.3.A, 4lj8.1.A, 4ljy.1.A, 4lk2.1.A, 4lk2.2.A, 4lps.1.A, 4lwz.1.A, 4lx0.1.A, 4ly6.1.A, 4ly6.1.B, 4ly6.1.C, 4ly6.1.D, 4ly6.1.E, 4ly6.1.F, 4ly6.2.F, 4lya.1.A, 4lzz.1.A, 4lzz.1.B, 4lzz.1.C, 4lzz.1.D, 4lzz.1.E, 4lzz.1.F, 4lzz.3.F, 4m30.1.A, 4m30.1.B, 4m4w.1.A, 4m4w.1.B, 4m4w.1.C, 4m4w.1.D, 4m4w.1.E, 4m4w.1.F, 4m4w.1.J, 4m4w.1.K, 4m4w.1.L, 4m4w.1.M, 4m4w.1.N, 4m4w.1.O, 4n0n.1.A, 4n0o.1.A, 4n0o.2.A, 4n0o.3.A, 4n0o.4.A, 4n1a.1.A, 4n1a.2.A, 4n1a.4.A, 4ngb.1.B, 4ngf.4.A, 4ngf.4.B, 4ngg.1.B, 4nha.2.A, 4nho.1.A, 4nkr.1.A, 4nkr.2.A, 4nkr.2.B, 4nkr.3.A, 4nkr.3.B, 4nl4.1.A, 4nl8.2.B, 4nmn.1.A, 4nmn.1.B, 4ntz.1.A, 4nu0.1.A, 4nu0.2.A, 4o3m.1.A, 4ojk.1.A, 4ojq.1.A, 4ojq.1.B, 4ok3.1.A, 4ok3.1.B, 4oks.1.A, 4olb.1.A, 4on9.1.A, 4on9.2.A, 4oog.1.C, 4oun.1.A, 4oyh.1.A, 4oyh.2.A, 4oyh.2.B, 4oyh.3.A, 4oyh.3.B, 4pfo.1.A, 4pht.1.B, 4pj3.1.A, 4pjj.1.A, 4pjk.1.A, 4pjl.1.A, 4pk4.1.A, 4px9.1.A, 4pxa.1.A, 4pzl.1.A, 4pzl.1.B, 4q2c.1.A, 4q47.1.A, 4q47.2.A, 4q48.1.A, 4q48.2.A, 4qnm.1.A, 4qqw.6.A, 4qqx.1.A, 4qqx.4.A, 4qrh.1.A, 4qrh.1.B, 4qu4.1.A, 4r2h.1.A, 4r7z.1.A, 4s20.1.F, 4s20.2.F, 4tl6.1.A, 4tl6.1.E, 4tl8.1.A, 4tl8.1.C, 4tl8.1.D, 4tl9.1.A, 4tl9.1.B, 4tl9.1.D, 4tl9.1.E, 4tla.1.C, 4tla.1.E, 4tla.1.F, 4tlb.1.A, 4tlb.1.B, 4tlb.1.D, 4tlb.1.E, 4tlc.1.A, 4tlc.1.B, 4tlc.1.D, 4tlc.1.E, 4tld.1.A, 4tld.1.C, 4tld.1.D, 4tle.1.A, 4tle.1.B, 4tle.1.D, 4tle.1.F, 4tmu.1.A, 4tsf.1.B, 4tsf.1.E, 4tt3.1.A, 4tt3.1.C, 4tyn.1.A, 4tyw.1.A, 4tyy.1.A, 4tz0.1.A, 4u4c.1.A, 4u7d.1.A, 4uaq.1.A, 4ue5.1.D, 4uj3.1.A, 4unn.1.A, 4unp.1.A, 4unq.1.A, 4unq.2.A, 4uxh.1.A, 4v1a.1.H, 4w5h.1.A, 4w5j.1.A, 4w5j.3.A, 4w5n.1.A, 4w5q.1.A, 4w5w.1.A, 4w7s.1.A, 4w7s.1.B, 4wft.2.A, 4wia.1.A, 4wvy.1.B, 4wvy.1.E, 4ww0.1.A, 4ww0.1.B, 4ww0.1.C, 4ww4.1.B, 4ww4.2.E, 4wxp.1.A, 4wxr.1.A, 4wyq.1.A, 4wyq.1.B, 4wyq.2.A, 4wyq.2.B, 4x8w.1.A, 4x8w.1.B, 4x8w.1.C, 4x8w.1.D, 4x8w.2.B, 4xd7.1.A, 4xd7.1.B, 4xd7.1.C, 4xd7.1.D, 4xd7.1.E, 4xd7.1.F, 4xgc.1.C, 4xgc.1.D, 4xgc.1.G, 4xgt.1.A, 4xgu.1.A, 4xgu.1.B, 4xgu.1.C, 4xgu.1.D, 4xgu.1.E, 4xgu.1.F, 4xjx.1.A, 4xjx.1.B, 4xqk.1.A, 4xqk.2.A, 4yds.1.A, 4ypl.1.A, 4ypl.1.B, 4ypl.1.C, 4ypl.1.D, 4ypl.1.E, 4ypl.1.F, 4ypn.1.A, 4ys0.1.A, 4yxw.1.A, 4yxw.1.B, 4yxw.1.D, 4yxw.1.E, 4z4h.1.A, 4z51.1.A, 4z54.1.A, 4z54.1.B, 4z8x.1.A, 4zc0.1.A, 4zc0.1.B, 4zcf.1.C, 4zlk.1.A, 4znj.1.A, 4znk.1.A, 4znl.2.A, 4znl.3.A, 4zpx.1.A, 5a5b.1.Q, 5a5b.1.R, 5a5b.1.S, 5a5b.1.T, 5a5b.1.U, 5a5b.1.V, 5a9j.1.A, 5a9j.3.A, 5aga.1.A, 5anr.2.B, 5aor.1.A, 5aor.2.A, 5ar1.1.A, 5ara.1.A, 5ara.1.C, 5ara.1.D, 5ara.1.F, 5are.1.A, 5are.1.B, 5are.1.C, 5are.1.D, 5are.1.E, 5arh.1.A, 5arh.1.C, 5arh.1.D, 5arh.1.E, 5arh.1.F, 5ari.1.A, 5ari.1.C, 5ari.1.D, 5ari.1.E, 5b16.1.A, 5b7i.1.A, 5bq5.1.A, 5c18.1.A, 5c18.1.B, 5c18.1.C, 5c18.1.D, 5c18.1.E, 5c18.1.F, 5c19.1.A, 5c19.1.B, 5c19.1.C, 5c19.1.D, 5c19.1.E, 5c19.1.F, 5c1a.1.A, 5c1a.1.B, 5c1a.1.C, 5c1a.1.D, 5c1a.1.E, 5c1a.1.F, 5c1a.2.A, 5c1a.2.B, 5c1a.2.C, 5c1a.2.D, 5c1a.2.E, 5c1a.2.F, 5c1b.1.A, 5c1b.1.B, 5c1b.1.C, 5c1b.1.E, 5c1b.1.F, 5c3c.1.A, 5c3c.1.B, 5cff.1.C, 5ck4.1.A, 5ck5.1.A, 5ck5.2.A, 5ck5.3.A, 5ck5.4.A, 5cyo.1.A, 5cyo.2.A, 5cyp.1.A, 5d0u.1.A, 5d4w.1.A, 5dca.1.A, 5dtu.1.A, 5dv7.1.C, 5dyg.1.E, 5dyi.1.A, 5dzr.1.A, 5e02.1.A, 5e3h.1.A, 5e4f.1.A, 5e4f.2.A, 5e7i.1.A, 5e7i.2.A, 5e7i.3.A, 5e7j.1.A, 5e7m.1.A, 5e7p.1.A, 5ean.1.A, 5eaw.1.A, 5eax.1.A, 5elx.1.A, 5ep0.1.A, 5ep2.1.A, 5ep4.1.A, 5eqt.1.A, 5eul.1.A, 5ext.1.D, 5exx.1.A, 5f3o.1.A, 5f3o.1.B, 5f3q.1.A, 5f4h.1.A, 5f4h.1.B, 5f4h.1.C, 5f4h.1.D, 5f4h.1.E, 5f4h.1.F, 5f98.1.C, 5f9f.1.A, 5f9f.1.E, 5f9h.1.E, 5ffj.1.A, 5ffj.2.A, 5ffm.1.A, 5fhd.2.B, 5fhe.1.A, 5fhf.1.A, 5fhg.2.A, 5fhh.1.A, 5fhh.2.A, 5fij.1.A, 5fij.1.B, 5fij.1.C, 5fij.1.E, 5fij.1.F, 5fik.1.A, 5fik.1.B, 5fik.1.D, 5fik.1.E, 5fik.1.F, 5fil.1.A, 5fil.1.C, 5fil.1.F, 5fl3.1.A, 5fl7.1.A, 5fl7.1.B, 5fl7.1.C, 5fl7.1.D, 5fl7.1.E, 5fl7.1.F, 5fm6.1.A, 5fm6.1.B, 5fmf.1.0, 5fmf.1.A, 5fox.1.A, 5ftb.1.A, 5ftc.1.A, 5ftd.1.A, 5fte.1.A, 5ftf.1.A, 5ftj.1.B, 5ftk.1.E, 5ftl.1.B, 5ftm.1.A, 5ftn.1.A, 5fuv.1.A, 5fuw.1.B, 5fuy.1.A, 5fv0.1.A, 5fv0.2.A, 5g4f.1.A, 5g4f.1.B, 5g4f.1.C, 5g4f.1.D, 5g4f.1.E, 5g4f.1.F, 5g4g.1.A, 5gad.1.7, 5gaf.1.7, 5gan.1.I, 5gao.1.I, 5gi4.1.A, 5gi4.1.B, 5gjb.1.A, 5gjc.1.A, 5gjq.1.N, 5gjq.1.P, 5gjq.1.T, 5gjr.1.A, 5gjr.3.A, 5gjr.4.A, 5gjr.47.A, 5gjr.49.A, 5gjr.6.A, 5gju.1.A, 5gm6.1.B, 5gm6.1.W, 5gn1.1.A, 5gn1.4.A, 5gq1.1.A, 5gq1.1.B, 5gq1.1.C, 5gq1.1.D, 5gq1.1.E, 5gq1.1.F, 5gqh.1.A, 5grb.1.A, 5grb.1.B, 5grb.1.C, 5grb.1.D, 5grb.2.A, 5guh.1.A, 5gvr.1.A, 5gvs.1.A, 5gvs.3.A, 5gvu.1.A, 5gvu.2.A, 5gvu.3.A, 5h1b.1.C, 5h1c.1.C, 5h1y.1.A, 5h1y.2.A, 5h53.1.A, 5h8c.1.A, 5h8w.1.A, 5hd9.1.A, 5he8.1.A, 5he8.1.B, 5he8.2.A, 5he8.2.B, 5he8.3.A, 5he8.3.B, 5he8.4.A, 5he8.4.B, 5he8.5.A, 5he8.5.B, 5he8.6.A, 5he8.6.B, 5he9.1.A, 5hkk.2.D, 5hkk.2.E, 5hzr.1.A, 5i8q.1.A, 5ifs.1.D, 5ifw.1.B, 5ik2.1.A, 5ik2.1.B, 5ik2.1.C, 5ikn.1.D, 5ikn.1.E, 5ikn.1.F, 5ikn.1.G, 5ikn.1.H, 5ikn.1.I, 5ikn.1.J, 5irr.1.A, 5it5.1.A, 5it5.1.B, 5it5.1.C, 5it5.1.D, 5it5.1.E, 5it5.1.F, 5ivl.1.A, 5ivl.1.B, 5ivw.1.A, 5ivw.1.B, 5j1s.1.A, 5j1t.1.A, 5jaj.1.A, 5jb2.1.A, 5jbj.1.A, 5jc3.1.A, 5jc7.1.A, 5jcs.1.7, 5jed.1.A, 5jee.1.A, 5ji2.3.A, 5ji2.3.B, 5ji3.1.R, 5jji.1.A, 5jji.1.B, 5jji.1.D, 5jji.1.E, 5jji.1.F, 5jjl.1.C, 5jmt.1.A, 5jpq.1.8, 5jps.1.A, 5jpt.1.A, 5jpt.2.A, 5jrz.1.A, 5js2.1.A, 5jwh.1.A, 5jwo.1.A, 5jwr.1.A, 5jwr.2.A, 5jxr.1.A, 5jxt.1.A, 5jxt.1.B, 5jxt.1.E, 5jxt.1.F, 5jxt.1.L, 5jxt.1.O, 5jxt.1.R, 5jxt.1.S, 5jzc.1.A, 5jzv.1.A, 5k8l.1.A, 5k8t.1.A, 5k9t.1.A, 5kdd.1.A, 5kiw.1.A, 5kiw.1.B, 5kiy.1.A, 5kne.1.A, 5kne.1.B, 5kne.1.C, 5kne.1.D, 5kne.1.E, 5kne.1.F, 5kwa.1.A, 5kzf.1.A, 5kzf.1.B, 5kzf.1.C, 5kzf.1.D, 5kzf.1.E, 5kzf.1.F, 5kzf.2.A, 5kzf.2.B, 5kzf.2.C, 5kzf.2.D, 5kzf.2.E, 5kzf.2.F, 5l3q.1.A, 5l3r.1.A, 5l3r.1.B, 5l3s.1.B, 5l3s.2.B, 5l3s.3.B, 5l3s.4.B, 5l4g.1.2, 5l4g.1.3, 5l4g.1.4, 5l4g.1.5, 5l4g.1.6, 5l4g.1.7, 5l8v.1.A, 5lb2.1.A, 5lb3.1.A, 5lb3.2.A, 5lb4.1.A, 5lb5.1.A, 5lb8.1.A, 5lba.1.A, 5lba.2.A, 5lba.3.A, 5lbi.1.A, 5ld2.1.A, 5ld2.1.C, 5lj5.1.G, 5lj5.1.c, 5ln3.1.P, 5ln3.1.Q, 5ln3.1.R, 5ln3.1.S, 5ln3.1.T, 5ln3.1.U, 5lqx.1.E, 5lqx.1.F, 5lqx.1.G, 5lqx.1.H, 5lqx.1.I, 5lqy.1.G, 5lqz.1.F, 5lst.1.A, 5lta.1.A, 5ltj.1.A, 5ltk.1.A, 5m05.1.A, 5m32.1.2, 5m32.1.3, 5m32.1.4, 5m32.1.5, 5m32.1.6, 5m32.1.7, 5m52.1.A, 5m52.2.A, 5m59.1.B, 5m59.2.B, 5m59.3.B, 5m59.4.B, 5m5p.1.A, 5m5p.2.A, 5mbv.1.A, 5mbv.1.C, 5mc6.34.A, 5mfx.1.A, 5mp9.1.2, 5mp9.1.3, 5mp9.1.4, 5mp9.1.5, 5mp9.1.6, 5mp9.1.7, 5mpa.1.2, 5mpa.1.3, 5mpa.1.4, 5mpa.1.5, 5mpa.1.6, 5mpa.1.7, 5mpb.1.2, 5mpb.1.3, 5mpb.1.4, 5mpb.1.5, 5mpb.1.6, 5mpb.1.7, 5mpc.1.2, 5mpc.1.3, 5mpc.1.4, 5mpc.1.5, 5mpc.1.6, 5mpc.1.7, 5mq0.1.4, 5mrc.31.A, 5mrc.35.A, 5mrc.62.A, 5mvr.1.A, 5mz4.1.A, 5mz4.1.B, 5mzn.1.A, 5n69.1.A, 5n69.1.B, 5n6a.1.A, 5n8l.1.A, 5n8m.1.A, 5n8o.1.A, 5n8s.1.A, 5n8s.2.A, 5n8u.2.A, 5n8y.1.B, 5n90.2.A, 5n94.1.A, 5n98.2.A, 5n9f.1.A, 5n9f.2.A, 5nco.1.9, 5niy.1.A, 5np7.1.D, 5np9.1.A, 5npa.1.A, 5npg.1.A, 5nq5.1.A, 5nrn.1.A, 5nrn.1.B, 5nss.1.F, 5nss.1.G, 5nss.1.H, 5nss.1.I, 5nss.1.K, 5nt7.1.A, 5nwl.1.A, 5nwl.1.G, 5o2l.1.A, 5o6b.1.A, 5o6b.1.B, 5o6d.1.A, 5o6d.2.A, 5o6e.1.A, 5o6e.2.A, 5o9z.1.C, 5oaf.1.A, 5oaf.1.B, 5oaf.1.C, 5oaf.1.D, 5oaf.1.E, 5oaf.1.F, 5oc4.1.A, 5oc5.1.A, 5oc6.1.A, 5oe8.1.A, 5oe8.2.A, 5oe8.3.A, 5oea.1.A, 5oea.4.A, 5oea.6.A, 5of4.1.A, 5of4.1.B, 5ofo.1.A, 5ofo.1.B, 5ofo.1.C, 5ofo.1.D, 5ofo.1.E, 5ofo.1.F, 5og1.1.A, 5og1.1.B, 5og1.1.C, 5og1.1.D, 5og1.1.E, 5og1.1.F, 5oiu.1.A, 5oiu.1.C, 5oiu.1.E, 5ooq.1.A, 5oqj.1.3, 5oqj.1.W, 5oql.1.9, 5qub.1.A, 5qul.1.A, 5qum.1.A, 5qum.2.A, 5qun.1.A, 5rob.1.A, 5rob.2.A, 5sup.1.A, 5sup.2.A, 5sup.3.A, 5suq.1.A, 5suq.1.C, 5sva.1.Y, 5sva.1.Z, 5swj.1.A, 5syp.1.A, 5syp.2.A, 5syr.1.A, 5t0c.13.A, 5t0c.48.A, 5t0c.50.A, 5t0g.1.O, 5t0g.1.P, 5t0g.1.Q, 5t0g.1.R, 5t0g.1.S, 5t0g.1.T, 5t0h.1.A, 5t0h.1.B, 5t0h.1.C, 5t0h.1.D, 5t0h.1.E, 5t0h.1.F, 5t0i.1.A, 5t0i.1.B, 5t0i.1.C, 5t0i.1.D, 5t0i.1.E, 5t0i.1.F, 5t0j.1.P, 5t0j.1.Q, 5t0j.1.R, 5t0j.1.S, 5t0j.1.T, 5t0j.1.U, 5t16.1.A, 5t16.1.B, 5t7b.1.B, 5tnu.1.A, 5tnu.2.A, 5tsg.1.A, 5tsg.1.C, 5tsh.1.A, 5tsh.1.B, 5tsh.1.E, 5txg.1.A, 5txv.3.D, 5u32.1.A, 5ubv.1.A, 5ubv.2.A, 5uie.1.A, 5uie.1.B, 5uie.1.D, 5uie.1.E, 5uie.1.F, 5uj7.1.A, 5uj7.1.B, 5uj7.1.C, 5uj7.2.A, 5uj7.2.B, 5uj7.2.C, 5ujm.1.A, 5ujm.1.E, 5urj.1.A, 5urm.1.A, 5urm.2.A, 5v7x.1.A, 5v8f.1.H, 5v8f.1.I, 5v8f.1.L, 5v8f.1.M, 5v9x.1.A, 5vc7.1.A, 5vca.1.A, 5vca.1.B, 5vca.1.C, 5vca.1.D, 5vca.1.E, 5vca.1.F, 5vfp.1.d, 5vfp.1.e, 5vfp.1.f, 5vfp.1.g, 5vfp.1.h, 5vfp.1.i, 5vfq.1.d, 5vfq.1.e, 5vfq.1.f, 5vfq.1.g, 5vfq.1.h, 5vfq.1.i, 5vfr.1.d, 5vfr.1.e, 5vfr.1.f, 5vfr.1.g, 5vfr.1.h, 5vfr.1.i, 5vfs.1.d, 5vfs.1.e, 5vfs.1.f, 5vfs.1.g, 5vfs.1.h, 5vfs.1.i, 5vft.1.L, 5vft.1.M, 5vft.1.N, 5vft.1.O, 5vft.1.P, 5vft.1.Q, 5vfu.1.L, 5vfu.1.M, 5vfu.1.N, 5vfu.1.O, 5vfu.1.P, 5vfu.1.Q, 5vh9.1.A, 5vha.1.A, 5vhc.1.A, 5vhe.1.A, 5vhf.1.B, 5vhf.1.C, 5vhf.1.D, 5vhf.1.E, 5vhf.1.F, 5vhf.1.G, 5vhi.1.A, 5vhi.1.B, 5vhi.1.C, 5vhi.1.D, 5vhi.1.E, 5vhi.1.F, 5vhj.1.B, 5vhj.1.C, 5vhj.1.D, 5vhj.1.E, 5vhj.1.F, 5vhj.1.G, 5vhm.1.B, 5vhm.1.C, 5vhm.1.D, 5vhm.1.E, 5vhm.1.F, 5vhm.1.G, 5vhn.1.B, 5vhn.1.C, 5vhn.1.D, 5vhn.1.E, 5vhn.1.F, 5vhn.1.G, 5vho.1.B, 5vho.1.C, 5vho.1.D, 5vho.1.E, 5vho.1.F, 5vho.1.G, 5vhp.1.B, 5vhp.1.C, 5vhp.1.D, 5vhp.1.E, 5vhp.1.F, 5vhp.1.G, 5vhq.1.B, 5vhq.1.C, 5vhq.1.D, 5vhq.1.E, 5vhq.1.F, 5vhq.1.G, 5vhr.1.B, 5vhr.1.C, 5vhr.1.D, 5vhr.1.E, 5vhr.1.F, 5vhr.1.G, 5vhs.1.A, 5vhs.1.B, 5vhs.1.C, 5vhs.1.D, 5vhs.1.E, 5vhs.1.F, 5vi7.1.A, 5vjh.1.A, 5vjh.1.B, 5vjh.1.C, 5vjh.1.D, 5vjh.1.E, 5vjh.1.F, 5vlj.1.A, 5vm9.1.A, 5vm9.2.A, 5vqa.1.A, 5vvr.1.M, 5vy8.1.A, 5vy8.1.B, 5vy8.1.C, 5vy8.1.D, 5vy8.1.E, 5vy8.1.F, 5vy9.1.A, 5vy9.1.B, 5vy9.1.C, 5vy9.1.D, 5vy9.1.E, 5vy9.1.F, 5vya.1.A, 5vya.1.B, 5vya.1.C, 5vya.1.D, 5vya.1.E, 5vya.1.F, 5w0t.1.A, 5w1a.1.A, 5w1a.2.A, 5w6v.1.A, 5wbw.1.A, 5wbw.2.A, 5wbw.3.A, 5wc0.1.A, 5wc0.1.D, 5wc0.1.E, 5wc0.1.F, 5wc1.1.A, 5wc2.1.A, 5wcb.1.A, 5wcb.1.B, 5wcb.1.C, 5wcb.1.D, 5wcb.1.E, 5wcb.1.F, 5wdx.1.A, 5wea.1.A, 5wsg.1.h, 5wvi.1.A, 5wvi.1.B, 5wvi.1.P, 5wvi.1.Q, 5wvi.1.R, 5wvi.1.W, 5wvk.1.2, 5wvk.1.3, 5wvk.1.4, 5wvk.1.5, 5wvk.1.6, 5wvk.1.7, 5wwp.1.A, 5wwp.2.A, 5wx1.1.A, 5x06.1.E, 5x06.1.G, 5x0x.1.K, 5x0y.1.K, 5xc6.1.A, 5xc7.1.A, 5xdr.1.A, 5xjc.1.Y, 5xmi.1.A, 5xmi.1.B, 5xmi.1.C, 5xmi.1.D, 5xmi.1.E, 5xmi.1.F, 5xmk.1.A, 5xmk.1.B, 5xmk.1.C, 5xmk.1.D, 5xmk.1.E, 5xmk.1.F, 5y4z.1.A, 5y6m.1.A, 5y6n.1.A, 5y88.1.W, 5ylz.1.W, 5yvj.1.A, 5yvu.1.A, 5yvv.1.A, 5yvw.1.A, 5yvy.1.B, 5yw1.1.A, 5yww.1.A, 5yz8.1.A, 5yzg.1.0, 5yzg.1.5, 5yzg.1.D, 5z2m.1.B, 5z2m.2.A, 5z3g.1.Y, 5z3l.1.K, 5z3o.1.K, 5z3q.1.A, 5z3q.1.B, 5z3q.1.C, 5z3q.1.D, 5z3q.2.A, 5z3u.1.A, 5z3v.1.A, 5z56.1.v, 5z57.1.g, 5z6q.1.A, 5z6r.1.A, 5zak.1.A, 5zak.1.B, 5zal.1.A, 5zal.1.B, 5zam.1.A, 5zam.1.B, 5zbz.1.A, 5zc9.1.A, 5zfq.1.A, 5zfr.1.A, 5zfr.1.B, 5zfr.1.C, 5zql.1.A, 5zql.2.A, 5zqm.1.A, 5zr1.1.A, 5zr1.1.D, 5zr1.1.E, 5ztm.1.A, 5ztm.1.B, 5zui.1.D, 5zwm.1.0, 6ac6.1.A, 6ac6.1.B, 6ac8.1.B, 6ac8.2.A, 6aca.1.A, 6acx.1.B, 6adw.1.A, 6ah0.1.s, 6ahd.58.A, 6ahf.1.A, 6ahf.1.B, 6ahf.1.C, 6ahf.1.D, 6ahf.1.E, 6ahf.1.F, 6aib.1.A, 6aic.1.A, 6amn.1.A, 6ap1.1.A, 6ap1.1.B, 6ap1.1.E, 6ap1.1.G, 6az0.1.A, 6az0.1.B, 6az0.1.C, 6az0.1.D, 6az0.1.E, 6az0.1.F, 6azy.1.A, 6b4i.1.C, 6b4j.2.C, 6b4k.1.A, 6b4k.2.A, 6b5b.1.C, 6b5c.1.A, 6b5d.1.A, 6b8h.1.T, 6b8h.1.U, 6b8h.1.V, 6b8h.1.W, 6b8h.1.r, 6b8h.1.s, 6bb8.1.A, 6bbm.1.A, 6bbm.1.B, 6bbm.1.C, 6bbm.1.D, 6bbm.1.E, 6bbm.1.F, 6bge.1.A, 6bih.1.A, 6bk8.1.S, 6blb.1.A, 6bmf.1.A, 6bmf.1.B, 6bmf.1.E, 6bnp.1.A, 6bnp.1.B, 6bnp.1.C, 6bnp.1.D, 6bnp.1.E, 6bnp.1.F, 6bnq.1.A, 6bnq.1.B, 6bnq.1.C, 6bnq.1.D, 6bnq.1.E, 6bnq.1.F, 6bnv.1.A, 6bnw.1.A, 6bog.1.A, 6bog.2.A, 6bu9.1.A, 6bua.1.A, 6c0f.1.8, 6c1d.1.F, 6c1g.1.A, 6c1h.1.F, 6c2u.1.A, 6c2v.1.A, 6c66.1.A, 6c90.1.A, 6chs.1.B, 6cp3.1.M, 6cp3.1.N, 6cp6.1.N, 6crm.1.A, 6cy1.1.A, 6cy1.1.B, 6cy5.1.A, 6cz5.1.A, 6d00.1.A, 6d00.1.B, 6d00.1.C, 6d00.1.D, 6d00.1.E, 6d00.1.F, 6d6r.1.M, 6dcr.1.A, 6dcr.2.A, 6dgd.1.A, 6dgd.2.A, 6dju.1.A, 6dju.1.C, 6dju.1.F, 6dju.1.G, 6djv.1.B, 6djv.1.D, 6djv.1.F, 6djv.1.G, 6due.1.A, 6duq.2.C, 6e10.1.A, 6e10.1.B, 6e10.1.C, 6e10.1.D, 6e10.1.E, 6e10.1.F, 6e11.1.D, 6e11.1.E, 6e11.1.F, 6e11.1.G, 6e11.1.H, 6e11.1.J, 6ed3.1.C, 6ed3.1.E, 6ed3.1.F, 6ef0.1.H, 6ef0.1.I, 6ef0.1.J, 6ef0.1.K, 6ef0.1.L, 6ef0.1.M, 6ef1.1.H, 6ef1.1.I, 6ef1.1.J, 6ef1.1.K, 6ef1.1.L, 6ef1.1.M, 6ef2.1.H, 6ef2.1.I, 6ef2.1.J, 6ef2.1.K, 6ef2.1.L, 6ef2.1.M, 6ef3.1.O, 6ef3.1.P, 6ef3.1.Q, 6ef3.1.R, 6ef3.1.S, 6ef3.1.T, 6eg2.1.A, 6eg3.1.A, 6ei8.1.A, 6ej5.1.A, 6ejf.1.M, 6ejf.1.N, 6ejf.1.O, 6ejf.1.P, 6ejf.1.Q, 6ejf.1.R, 6em3.1.0, 6em8.1.A, 6em8.1.B, 6em8.1.C, 6em8.1.D, 6em8.1.E, 6em8.1.F, 6em8.1.G, 6em8.1.H, 6em8.1.I, 6em8.1.J, 6em9.1.A, 6em9.1.B, 6em9.1.C, 6em9.1.D, 6em9.1.E, 6em9.1.F, 6em9.1.G, 6em9.1.H, 6em9.1.I, 6em9.1.J, 6emw.1.B, 6emw.1.E, 6epc.1.0, 6epc.1.1, 6epc.1.2, 6epc.1.3, 6epc.1.4, 6epc.1.5, 6epd.1.0, 6epd.1.1, 6epd.1.2, 6epd.1.3, 6epd.1.4, 6epd.1.5, 6epe.1.0, 6epe.1.1, 6epe.1.2, 6epe.1.3, 6epe.1.4, 6epe.1.5, 6epf.1.0, 6epf.1.1, 6epf.1.2, 6epf.1.3, 6epf.1.4, 6epf.1.5, 6eud.1.A, 6exn.1.T, 6f00.1.A, 6f0x.1.A, 6f0x.1.B, 6f0x.1.C, 6f0x.1.D, 6f0x.1.E, 6f0x.1.F, 6f4a.1.B, 6f8l.1.M, 6f8l.1.N, 6f8l.1.O, 6f8l.1.P, 6f8l.1.Q, 6f8l.1.R, 6f9s.1.A, 6fa5.1.A, 6fa9.1.A, 6faa.1.A, 6fac.1.A, 6fml.1.A, 6fml.1.B, 6fml.1.C, 6fml.1.D, 6fml.1.E, 6fml.1.F, 6fml.1.G, 6fo1.1.A, 6fo1.1.D, 6fo1.1.E, 6foc.1.A, 6foc.1.B, 6foc.1.C, 6foc.1.E, 6foc.1.F, 6frk.1.t, 6fsz.1.N, 6ft6.1.t, 6ftx.1.M, 6fvt.1.f, 6fvt.1.g, 6fvt.1.h, 6fvt.1.i, 6fvt.1.j, 6fvt.1.k, 6fvu.1.f, 6fvu.1.g, 6fvu.1.h, 6fvu.1.i, 6fvu.1.j, 6fvv.1.f, 6fvv.1.g, 6fvv.1.h, 6fvv.1.i, 6fvv.1.j, 6fvv.1.k, 6fvw.1.f, 6fvw.1.g, 6fvw.1.h, 6fvw.1.i, 6fvw.1.j, 6fvw.1.k, 6fvx.1.f, 6fvx.1.g, 6fvx.1.h, 6fvx.1.i, 6fvx.1.j, 6fvx.1.k, 6fvy.1.f, 6fvy.1.g, 6fvy.1.h, 6fvy.1.i, 6fvy.1.j, 6fvy.1.k, 6fwr.1.A, 6fws.1.A, 6fws.1.C, 6g0l.1.K, 6g0l.1.L, 6g19.1.A, 6g1s.1.A, 6g1x.1.A, 6g2v.1.A, 6g2w.1.A, 6g2x.1.A, 6g2y.1.A, 6g2z.1.A, 6g30.1.A, 6g7e.1.A, 6gaw.44.A, 6gcn.1.B, 6gcn.2.C, 6gco.1.C, 6gco.1.F, 6geb.1.A, 6geb.1.B, 6geb.1.C, 6geb.1.D, 6geb.1.E, 6geb.1.F, 6gef.1.A, 6gef.1.B, 6gef.1.C, 6gef.1.D, 6gef.1.E, 6gef.1.F, 6gej.1.L, 6gej.1.O, 6gej.1.P, 6gej.1.Q, 6gej.1.R, 6gej.1.S, 6gej.1.T, 6gen.1.P, 6gen.1.Q, 6gjz.1.A, 6gkh.1.A, 6gox.1.A, 6gpg.1.C, 6h57.1.A, 6h61.1.A, 6h66.1.A, 6h7x.1.A, 6hd0.1.G, 6hd3.1.A, 6he4.1.A, 6he4.1.B, 6he4.1.C, 6he4.1.D, 6he4.1.E, 6he4.1.F, 6he8.1.2, 6he8.1.3, 6he8.1.4, 6he8.1.5, 6he8.1.6, 6he8.1.7, 6he9.1.2, 6he9.1.3, 6he9.1.4, 6he9.1.5, 6he9.1.6, 6he9.1.7, 6hea.1.2, 6hea.1.3, 6hea.1.4, 6hea.1.5, 6hea.1.6, 6hea.1.7, 6hec.1.2, 6hec.1.3, 6hec.1.4, 6hec.1.5, 6hec.1.6, 6hec.1.7, 6hed.1.2, 6hed.1.3, 6hed.1.4, 6hed.1.5, 6hed.1.6, 6hed.1.7, 6heg.1.A, 6hf7.1.A, 6hiv.47.A, 6hph.1.A, 6hpt.1.A, 6hpu.2.A, 6hqu.2.A, 6hqu.3.A, 6hqu.4.A, 6hqu.6.A, 6hqu.8.A, 6hts.1.A, 6hts.1.B, 6hts.1.C, 6hts.1.D, 6hts.1.E, 6hts.1.F, 6hts.1.G, 6htu.1.C, 6htu.1.D, 6htu.1.E, 6hyp.1.A, 6hys.2.A, 6hyt.3.A, 6hyu.1.A, 6hyu.2.A, 6hz4.1.A, 6hz4.1.B, 6hz4.1.C, 6hz4.1.D, 6hz4.1.E, 6hz4.1.F, 6i26.1.A, 6i27.1.A, 6i3o.1.A, 6i3o.2.A, 6i3p.1.A, 6i3r.1.A, 6i9r.1.a, 6icz.1.b, 6id1.1.g, 6ieg.1.A, 6ieg.2.A, 6ieh.1.A, 6igm.1.A, 6igm.1.B, 6igm.1.C, 6igm.1.D, 6igm.1.E, 6igm.1.F, 6igm.1.H, 6ip2.1.A, 6ip2.1.B, 6ip2.1.C, 6ip2.1.D, 6ip2.1.E, 6ip2.1.F, 6iro.1.A, 6itc.1.A, 6iy1.4.A, 6iy2.1.K, 6iy3.1.K, 6iy8.1.A, 6iy8.1.B, 6iy8.1.C, 6iy8.1.D, 6j17.1.A, 6j18.1.A, 6j19.1.A, 6j2c.1.2, 6j2c.1.3, 6j2c.1.4, 6j2c.1.5, 6j2c.1.6, 6j2c.1.7, 6j2n.1.2, 6j2n.1.3, 6j2n.1.4, 6j2n.1.5, 6j2n.1.6, 6j2n.1.7, 6j2q.1.2, 6j2q.1.3, 6j2q.1.4, 6j2q.1.5, 6j2q.1.6, 6j2q.1.7, 6j2x.1.3, 6j2x.1.4, 6j2x.1.5, 6j2x.1.6, 6j2x.1.7, 6j30.1.3, 6j30.1.4, 6j30.1.5, 6j30.1.6, 6j30.1.7, 6j5i.1.A, 6j5i.1.B, 6j5i.1.C, 6j5i.1.D, 6j5i.1.E, 6j5i.1.F, 6j5j.1.A, 6j5j.1.C, 6j5j.1.D, 6j5j.1.E, 6j5j.1.F, 6j5k.65.A, 6j7e.1.A, 6jd4.1.A, 6jd5.1.A, 6jde.1.A, 6jde.2.A, 6jdi.1.A, 6jdl.1.A, 6jds.1.A, 6jim.1.A, 6jpq.1.A, 6jpu.1.A, 6jq0.1.A, 6jq0.1.B, 6jq0.1.C, 6jq0.1.D, 6jq0.1.E, 6jq0.1.F, 6jtz.1.A, 6jyl.1.K, 6jyt.1.A, 6jyt.2.A, 6k0r.1.A, 6k0r.1.D, 6k0r.1.F, 6k1p.1.K, 6kr6.1.A, 6kw3.1.1, 6kw4.1.1, 6kw5.1.1, 6kww.1.A, 6kww.11.A, 6kww.13.A, 6kww.16.A, 6kww.2.A, 6kww.24.A, 6kww.5.A, 6kww.6.A, 6kww.7.A, 6kww.8.A, 6kww.9.A, 6kyv.1.B, 6kza.1.A, 6kza.2.A, 6l1q.1.A, 6l3g.1.E, 6l5l.1.A, 6l5m.2.A, 6l5m.3.A, 6l5m.4.A, 6l5n.1.A, 6l5n.2.A, 6l5o.1.A, 6l8d.1.A, 6l8d.1.D, 6l8d.1.E, 6l8o.1.A, 6lk0.1.A, 6ln3.1.A, 6lqp.59.A, 6lqq.58.A, 6lqs.75.A, 6lsy.1.A, 6lt4.1.A, 6ltj.1.I, 6lua.1.A, 6lxd.1.A, 6lxe.1.A, 6m40.1.A, 6m5r.1.A, 6m5s.1.A, 6m5u.1.A, 6m5v.1.A, 6m6a.1.F, 6m6b.1.F, 6mck.1.A, 6mdm.1.A, 6mdm.1.B, 6mdm.1.C, 6mdm.1.D, 6mdm.1.E, 6mdm.1.F, 6mdn.1.A, 6mdn.1.B, 6mdn.1.C, 6mdn.1.D, 6mdn.1.E, 6mdn.1.F, 6mdp.1.A, 6mdp.1.B, 6mdp.1.C, 6mdp.1.D, 6mdp.1.E, 6mdp.1.F, 6mfn.1.A, 6mfr.1.A, 6mfr.2.A, 6mfu.1.A, 6mh3.1.A, 6mq9.1.A, 6mq9.2.A, 6mq9.3.A, 6mqb.1.A, 6mqk.4.A, 6mql.1.A, 6msb.1.M, 6msb.1.N, 6msb.1.O, 6msb.1.P, 6msb.1.Q, 6msb.1.R, 6msd.1.O, 6msd.1.P, 6msd.1.Q, 6msd.1.R, 6mse.1.M, 6mse.1.N, 6mse.1.O, 6mse.1.P, 6mse.1.Q, 6mse.1.R, 6msg.1.M, 6msg.1.N, 6msg.1.O, 6msg.1.P, 6msg.1.Q, 6msg.1.R, 6msh.1.M, 6msh.1.O, 6msh.1.P, 6msh.1.Q, 6msh.1.R, 6msj.1.M, 6msj.1.N, 6msj.1.O, 6msj.1.P, 6msj.1.Q, 6msj.1.R, 6msk.1.M, 6msk.1.N, 6msk.1.O, 6msk.1.P, 6msk.1.Q, 6msk.1.R, 6n2i.1.A, 6n2z.1.O, 6n2z.1.P, 6n30.1.N, 6n30.1.O, 6n30.1.P, 6n30.1.Q, 6n30.1.R, 6n30.1.S, 6n4o.1.A, 6n53.1.A, 6n53.1.B, 6n55.1.A, 6n55.1.B, 6n55.2.A, 6n6l.1.A, 6n6l.2.A, 6n6m.1.A, 6n6m.2.A, 6n6z.1.A, 6n6z.2.A, 6n70.1.A, 6n70.2.A, 6n71.1.A, 6n71.2.A, 6n72.1.A, 6n72.2.A, 6n73.1.A, 6n73.2.A, 6n75.1.A, 6n75.2.A, 6n7s.1.A, 6n7s.1.B, 6n7s.1.C, 6n7s.1.D, 6n7s.1.E, 6n7s.1.F, 6n7t.1.A, 6n7t.1.F, 6n7v.1.F, 6n8t.1.A, 6n8t.1.B, 6n8t.1.C, 6n8t.1.D, 6n8t.1.E, 6n8t.1.F, 6n8v.1.A, 6n8v.1.B, 6n8v.1.C, 6n8v.1.D, 6n8v.1.E, 6n8v.1.F, 6n8z.1.A, 6n8z.1.B, 6n8z.1.C, 6n8z.1.D, 6n8z.1.E, 6n8z.1.F, 6n9v.1.A, 6n9v.1.C, 6n9v.1.D, 6n9v.1.F, 6ne3.1.K, 6nmi.1.A, 6nmi.1.B, 6nu2.35.A, 6nui.1.A, 6nyv.1.A, 6nyw.1.A, 6nyy.1.A, 6nyy.1.B, 6nyy.1.C, 6nyy.1.D, 6nyy.1.E, 6nyy.1.F, 6o16.1.A, 6o16.2.A, 6o1w.1.A, 6o1w.2.A, 6o1x.1.A, 6o1x.2.A, 6o1y.1.A, 6o1y.2.A, 6o1z.1.A, 6o5f.1.A, 6o8e.1.A, 6o8e.2.A, 6o8f.1.A, 6o8g.1.A, 6o8g.2.A, 6o8g.3.A, 6o8h.1.A, 6o9l.1.2, 6o9l.1.V, 6o9m.1.A, 6o9m.1.H, 6oa9.1.A, 6oa9.1.B, 6oa9.1.C, 6oa9.1.D, 6oa9.1.E, 6oa9.1.F, 6oaa.1.A, 6oaa.1.B, 6oaa.1.C, 6oaa.1.D, 6oab.1.A, 6oab.1.B, 6oab.1.C, 6oab.1.E, 6oax.1.A, 6oax.1.B, 6oax.1.C, 6oax.1.D, 6oax.1.E, 6oax.1.F, 6oay.1.A, 6oay.1.B, 6oay.1.C, 6oay.1.D, 6oay.1.E, 6oay.1.F, 6oif.1.A, 6ojx.1.F, 6ojy.1.A, 6ojy.1.E, 6ojy.1.F, 6ojz.1.D, 6ok2.1.A, 6ok2.1.B, 6ok2.1.C, 6ok2.1.E, 6ok2.1.F, 6okv.1.B, 6olj.1.A, 6olj.1.B, 6olj.1.C, 6olj.1.E, 6olk.1.D, 6oll.1.B, 6oll.1.D, 6olm.1.D, 6omb.1.A, 6omb.1.B, 6omb.1.C, 6omb.1.D, 6omb.1.E, 6on2.1.A, 6on2.1.B, 6on2.1.C, 6on2.1.D, 6on2.1.E, 6on2.1.F, 6oo2.1.A, 6oo2.1.D, 6oo2.1.E, 6oo2.1.G, 6oon.1.A, 6opc.1.F, 6oqr.1.C, 6oqr.1.D, 6oqt.1.B, 6oqu.1.C, 6oqv.1.B, 6oqv.1.C, 6oqv.1.D, 6oqw.1.B, 6oqw.1.C, 6oqw.1.D, 6or5.1.A, 6orb.1.A, 6p07.1.A, 6p07.1.B, 6p07.1.C, 6p07.1.E, 6p07.1.F, 6p11.1.A, 6p12.1.A, 6p14.1.A, 6p4f.1.A, 6p4o.1.A, 6p4o.2.A, 6p4o.3.A, 6p4w.1.A, 6p66.1.A, 6p66.2.A, 6p8v.1.A, 6p8v.1.B, 6p8v.1.C, 6p8v.1.D, 6p8v.1.E, 6p8v.1.F, 6pb3.1.A, 6pdw.1.A, 6pdw.1.B, 6pdw.1.C, 6pdw.1.D, 6pdw.1.E, 6pdy.1.A, 6pdy.1.B, 6pdy.1.C, 6pdy.1.D, 6pdy.1.E, 6pdy.1.F, 6pe0.1.A, 6pe0.1.B, 6pe0.1.C, 6pe0.1.D, 6pe0.1.E, 6pe0.1.F, 6pen.1.A, 6pen.1.B, 6pen.1.C, 6pen.1.D, 6pen.1.E, 6pen.1.G, 6pjw.1.A, 6pjw.1.C, 6pk5.1.A, 6pk5.1.B, 6pk5.1.C, 6pk5.2.B, 6po1.1.A, 6po1.1.B, 6po1.1.C, 6po3.1.A, 6po3.1.D, 6po3.1.E, 6pod.1.E, 6pod.1.F, 6pos.1.A, 6pos.1.B, 6pos.1.C, 6pos.1.D, 6pos.1.E, 6pos.1.F, 6pp5.1.D, 6pp5.1.E, 6pp5.1.F, 6pp6.1.B, 6pp6.1.C, 6pp6.1.F, 6pp7.1.A, 6pp7.1.C, 6pp7.1.D, 6ppj.1.A, 6ppj.1.B, 6ppr.1.A, 6ppr.1.B, 6ppu.1.A, 6psp.1.A, 6pwf.1.K, 6pwz.1.D, 6pwz.2.A, 6pwz.2.D, 6pxi.1.E, 6pxi.1.F, 6pxk.1.A, 6pxk.1.B, 6pxk.1.C, 6pxk.1.D, 6pxk.1.E, 6pxk.1.F, 6pxk.2.F, 6pxl.1.B, 6pxl.1.F, 6pxl.2.A, 6q7l.1.U, 6q7l.1.V, 6q7l.1.W, 6q7l.1.X, 6q7l.1.Y, 6q7l.1.Z, 6q7m.1.U, 6q7m.1.W, 6q7m.1.X, 6q7m.1.Y, 6qdj.1.A, 6qdv.1.0, 6qdv.1.D, 6qdv.1.H, 6qel.1.A, 6qel.1.B, 6qel.1.C, 6qel.1.D, 6qel.1.E, 6qel.1.F, 6qel.1.G, 6qel.1.H, 6qel.1.I, 6qel.1.J, 6qel.1.L, 6qem.1.A, 6qem.1.B, 6qem.1.C, 6qem.1.D, 6qem.1.E, 6qem.1.F, 6qem.1.G, 6qem.1.H, 6qem.1.J, 6qem.1.K, 6qem.1.L, 6qi8.1.A, 6qi8.1.B, 6qi8.1.C, 6qi8.1.D, 6qi8.1.E, 6qi8.1.F, 6qi9.1.A, 6qi9.1.B, 6qi9.1.C, 6qi9.1.D, 6qi9.1.E, 6qi9.1.F, 6qic.1.A, 6qic.2.A, 6qic.4.A, 6qid.1.A, 6qie.1.A, 6qp0.1.A, 6qpw.1.A, 6qs4.1.A, 6qs4.1.B, 6qs4.1.C, 6qs4.1.E, 6qs4.1.F, 6qs6.1.A, 6qs6.1.E, 6qs6.1.F, 6qs7.1.A, 6qs7.1.D, 6qs7.1.E, 6qs7.1.F, 6qs8.1.F, 6qv3.1.A, 6qv4.1.A, 6qw6.1.X, 6qws.1.A, 6qx9.23.A, 6qx9.64.A, 6r6g.79.A, 6ra4.1.A, 6rad.1.A, 6rae.1.A, 6rd4.1.2, 6rd4.1.3, 6rd4.1.4, 6rdc.1.2, 6rdc.1.3, 6rdc.1.4, 6rdg.1.R, 6rdg.1.S, 6rdg.1.T, 6rdh.1.4, 6rdk.1.3, 6rdk.1.4, 6rdq.1.2, 6rdq.1.3, 6rdq.1.4, 6rdw.1.3, 6rdw.1.4, 6re8.1.2, 6re8.1.4, 6res.1.2, 6res.1.3, 6res.1.4, 6rfl.1.N, 6rm8.1.A, 6rm9.1.A, 6rma.1.A, 6rmb.1.A, 6rmc.1.A, 6rmc.2.A, 6rn2.1.A, 6rn2.1.B, 6rn2.1.C, 6rn2.1.D, 6rn2.1.E, 6rn2.1.F, 6rn3.1.A, 6rn3.1.B, 6rn3.1.C, 6rn3.1.D, 6rn3.1.E, 6rn3.1.F, 6rn4.1.A, 6rn4.1.E, 6rn4.1.F, 6ro1.1.A, 6rqc.1.A, 6rqc.1.D, 6rqc.1.E, 6rwz.1.A, 6rxu.36.A, 6rxv.36.A, 6rxy.1.8, 6rxz.35.A, 6ryr.1.K, 6ryu.1.K, 6s0k.1.6, 6s3a.1.A, 6s3e.1.A, 6s3e.2.A, 6s3h.1.A, 6s3h.2.A, 6s3i.1.A, 6s3m.1.A, 6s3m.2.A, 6s3n.2.A, 6s3o.1.A, 6s3p.1.A, 6s8o.1.A, 6s8q.1.A, 6s8r.1.A, 6s8s.2.A, 6s9i.1.A, 6sdw.1.A, 6sdx.1.A, 6sdy.1.A, 6sfw.1.A, 6sfw.1.B, 6sfw.1.C, 6sfw.1.D, 6sfw.1.E, 6sfw.1.F, 6sh3.1.A, 6sh4.1.A, 6sh5.1.A, 6sh6.1.A, 6sh7.1.A, 6sjb.1.A, 6sje.1.A, 6sjg.1.C, 6sxa.1.A, 6sxb.1.A, 6sxh.1.A, 6sz9.1.A, 6sza.1.A, 6sza.1.C, 6sza.1.D, 6szb.1.C, 6szb.1.F, 6t2u.1.A, 6t4h.1.A, 6t66.1.A, 6t66.1.C, 6t66.1.D, 6t66.1.F, 6t8b.1.A, 6t8b.1.B, 6t8b.1.C, 6t8b.1.D, 6t8b.1.E, 6t8b.1.F, 6t8g.1.A, 6t8g.1.B, 6t8g.1.C, 6t8g.1.D, 6t8g.1.E, 6t8g.1.F, 6t8o.1.A, 6t8o.1.B, 6t8o.1.D, 6t8o.1.F, 6tda.1.S, 6tdy.1.D, 6tdy.1.E, 6tdy.1.F, 6tnn.1.B, 6tnn.1.C, 6tt7.1.A, 6tt7.1.B, 6tt7.1.C, 6tuu.1.A, 6tuu.4.A, 6tw4.1.A, 6tw9.1.A, 6u5z.1.A, 6ugd.1.A, 6ugd.1.B, 6ugd.1.C, 6ugd.1.E, 6ugd.1.F, 6uge.1.A, 6ugf.1.A, 6ugf.1.B, 6ugf.1.D, 6ugf.1.F, 6ui4.1.A, 6uko.1.A, 6uko.1.B, 6uko.1.C, 6uko.1.D, 6uko.1.E, 6uko.1.F, 6uko.1.G, 6ukp.1.F, 6uks.1.A, 6up2.1.A, 6up3.1.A, 6up4.1.A, 6upa.1.A, 6upa.1.B, 6upq.1.A, 6upq.1.B, 6upr.1.A, 6upr.1.B, 6uqe.1.A, 6uqe.1.B, 6uqe.1.C, 6uqe.1.D, 6uqe.1.E, 6uqe.1.F, 6uqo.1.A, 6uqo.1.B, 6uqo.1.C, 6uqo.1.D, 6uqo.1.E, 6uqo.1.F, 6uqq.1.B, 6ut3.1.A, 6ut3.1.B, 6ut3.1.C, 6ut3.1.D, 6ut3.1.E, 6ut3.1.F, 6ut4.1.A, 6ut4.1.B, 6ut4.1.C, 6ut4.1.E, 6ut4.1.F, 6ut5.1.A, 6ut5.1.B, 6ut5.1.C, 6ut5.1.D, 6ut5.1.E, 6ut5.1.F, 6ut6.1.A, 6ut6.1.B, 6ut6.1.C, 6ut6.1.D, 6ut6.1.E, 6ut6.1.F, 6ut7.1.A, 6ut7.1.B, 6ut7.1.C, 6ut7.1.D, 6ut7.1.E, 6ut7.1.F, 6uv0.1.A, 6uv0.2.A, 6uv1.1.A, 6uv2.1.A, 6uv4.1.A, 6uxw.1.N, 6v11.1.A, 6v11.1.B, 6v11.1.C, 6v11.1.D, 6v11.1.E, 6v11.1.F, 6v5b.1.A, 6v5b.1.B, 6v5b.1.C, 6v5c.1.A, 6v5c.1.B, 6v5c.1.C, 6vff.1.B, 6vfs.1.A, 6vfs.1.B, 6vfs.1.C, 6vfs.1.D, 6vfs.1.E, 6vfs.1.F, 6vfx.1.A, 6vfx.1.B, 6vfx.1.C, 6vfx.1.D, 6vfx.1.F, 6vmi.72.A, 6vvo.1.A, 6vvo.1.B, 6vvo.1.C, 6vvo.1.D, 6vvo.1.E, 6vz4.1.K, 6w1z.1.O, 6w1z.1.P, 6w1z.1.Q, 6w1z.1.R, 6w1z.1.S, 6w1z.1.T, 6w20.1.O, 6w20.1.P, 6w20.1.Q, 6w20.1.R, 6w20.1.S, 6w20.1.T, 6w21.1.A, 6w21.1.B, 6w21.1.C, 6w21.1.D, 6w21.1.E, 6w21.1.F, 6w6e.1.C, 6w6g.1.A, 6w6g.1.D, 6w6g.1.E, 6w6g.1.F, 6w6h.1.A, 6w6h.1.B, 6w6h.1.C, 6w6h.1.D, 6w6h.1.E, 6w6h.1.F, 6w6i.1.E, 6w6j.1.F, 6wa8.1.A, 6wa8.1.B, 6wa8.1.C, 6wa8.1.D, 6wa8.1.E, 6wa8.1.F, 6wct.1.A, 6wct.1.B, 6wct.2.B, 6wg3.1.A, 6wgc.1.A, 6wgc.1.E, 6wgg.1.C, 6wgi.1.H, 6wjd.1.M, 6wjd.1.N, 6wjd.1.O, 6wjd.1.P, 6wjd.1.Q, 6wjd.1.R, 6wjn.1.d, 6wjn.1.e, 6wjn.1.f, 6wjn.1.g, 6wjn.1.h, 6wjn.1.i, 6wqh.1.A, 6wqh.1.B, 6wqh.1.C, 6wqh.1.D, 6wqh.1.E, 6wqh.1.F, 6wrf.1.A, 6wrf.1.B, 6wrf.1.C, 6wrf.1.D, 6wrf.1.E, 6wrf.1.N, 6wsg.1.A, 6wsg.1.B, 6wsg.1.C, 6wsg.1.D, 6wsg.1.E, 6wsg.1.F, 6wvk.1.G, 6x26.1.A, 6x2f.1.A, 6x2n.1.A, 6x43.1.A, 6x4w.1.A, 6x4y.1.A, 6x50.1.A, 6x5z.1.C, 6x61.1.A, 6x61.2.A, 6x61.3.A, 6x61.4.A, 6x61.6.A, 6xaf.1.A, 6xaf.1.B, 6xas.1.L, 6xeo.1.A, 6xez.1.E, 6xez.1.F, 6xki.1.A, 6xtw.1.A, 6xtw.2.A, 6xyw.50.A, 6xzt.1.A, 6xzt.2.A, 6y2z.1.A, 6y2z.2.A, 6y30.1.A, 6y30.2.A, 6y31.1.A, 6y31.2.A, 6y32.1.A, 6y53.1.D, 6y5q.1.F, 6ybh.1.A, 6ydw.15.A, 6yhr.1.A, 6ylh.1.t, 6yny.44.A, 6yny.46.A, 6yny.48.A, 6yt1.1.B, 6yvh.1.F, 6ywe.33.A, 6ywe.39.A, 6yxu.1.F, 6yxx.15.A, 6yxy.9.A, 6yys.1.F, 6z1e.1.B, 6z1e.1.E, 6z1f.1.A, 6z1f.1.B, 6z1f.1.C, 6z1f.1.D, 6z1f.1.F, 6z1r.1.B, 6z1r.1.D, 6z1r.1.E, 6z1u.1.B, 6z1u.1.E, 6z6f.1.D, 6z6h.1.C, 6z6o.1.C, 6z6p.1.D, 6z9p.1.D, 6z9q.1.G, 6z9r.1.E, 6z9s.1.E, 6z9t.1.A, 6zbk.1.B, 6zca.1.G, 6zdw.1.A, 6zdw.1.B, 6zfb.1.N, 6zm2.1.A, 6zm5.35.A, 6zmw.1.t, 6znp.1.A, 6znq.2.A, 6zns.1.A, 6zpo.1.B, 6zpo.1.C, 6zpo.1.D, 6zpo.1.F, 6zpo.1.G, 6zqb.38.A, 6zqb.39.A, 6zqc.3.A, 6zqd.37.A, 6zqf.1.O, 6zqg.1.8, 6zqm.1.A, 6zqm.1.C, 6zqm.1.F, 6zqn.1.A, 6zqn.1.C, 6zsa.65.A, 6zsc.65.A, 6zse.65.A, 6zsl.1.A, 6zsl.2.A, 6zww.1.A, 6zww.2.A, 6zww.3.A, 6zww.4.A, 6zwx.1.A, 6zz6.1.B, 7a5f.35.A, 7a5g.35.A, 7a5h.1.7, 7a5i.36.A, 7a5k.36.A, 7a5p.1.T, 7a5p.1.e, 7a8r.1.A, 7a8r.2.A, 7abg.1.H, 7abi.1.G, 7abr.1.A, 7abr.1.B, 7abr.1.C, 7abr.1.D, 7abr.1.E, 7abr.1.F, 7ad8.1.C, 7ad8.1.E, 7ada.1.A, 7ada.2.A, 7adb.1.F, 7adc.1.B, 7aho.1.A, 7aho.1.B, 7aho.1.C, 7aho.1.D, 7aho.1.E, 7aho.1.F, 7ajc.1.G, 7ajd.1.C, 7aje.1.F, 7ajf.1.2, 7ajf.1.3, 7ajf.1.4, 7ajf.1.5, 7ajf.1.6, 7ajf.1.7, 7ajf.1.A, 7ajf.1.B, 7ajf.1.C, 7ajf.1.D, 7ajf.1.E, 7ajf.1.F, 7ajg.1.B, 7aji.1.5, 7ajj.1.6, 7ajt.3.A, 7ajt.6.A, 7ajt.79.A, 7aju.74.A, 7akp.1.A, 7alv.1.A, 7am2.64.A, 7amv.1.M, 7aoh.1.M, 7aoi.59.A, 7apd.1.B, 7apd.1.C, 7apd.1.D, 7apd.1.E, 7apd.1.F, 7apd.1.G, 7apk.1.G, 7apx.1.F, 7ase.1.A, 7ask.1.A, 7auc.1.A, 7aud.1.A, 7aud.2.A, 7aud.3.A, 7aud.4.A, 7aud.6.A, 7b9v.1.E, 7b9v.1.T, 7bbb.1.A, 7bdi.1.A, 7bdj.1.A, 7bdk.1.A, 7bdl.1.A, 7bil.1.A, 7bkp.1.A, 7bkq.1.A, 7blv.1.A, 7bm0.1.A, 7bp8.1.B, 7bp9.1.C, 7bpa.1.A, 7bpb.1.A, 7bst.1.A, 7bst.1.B, 7bto.1.F, 7btp.1.B, 7btq.1.F, 7btr.1.B, 7c98.1.C, 7c99.1.A, 7c9a.1.A, 7c9c.1.A, 7cg3.1.A, 7cg3.1.B, 7cg3.1.C, 7cg3.1.D, 7cg3.1.E, 7cg3.1.F, 7cgy.1.A, 7clg.1.A, 7clg.1.B, 7cte.1.C, 7ctf.1.D, 7ctf.1.E, 7cxm.1.G, 7cxm.1.H, 7cxn.1.G, 7cxn.1.H, 7cyq.1.G, 7d4i.78.A, 7d5t.1.r, 7dco.1.D, 7dcp.1.A, 7dcq.1.A, 7dcr.1.A, 7dd3.1.A, 7ddx.1.B, 7dey.1.A, 7dey.1.B, 7dey.2.A, 7dey.2.B, 7dhw.1.A, 7dtj.1.A, 7dtk.1.A, 7dtk.1.B, 7dxq.1.D, 7dye.1.A, 7dyi.1.A, 7dyj.1.A, 7dyk.1.A, 7e4v.1.A, 7e6v.1.A, 7e6v.2.A, 7egb.1.G, 7egc.1.G, 7egc.1.H, 7egp.1.H, 7egq.1.E, 7egq.1.M, 7egq.1.Q, 7egq.1.R, 7egt.1.A, 7eiz.1.J, 7eiz.1.K, 7ej6.1.A, 7ej7.1.A, 7ejc.1.C, 7ejw.1.C, 7ejw.1.D, 7eld.1.A, 7ele.1.A, 7enc.31.A, 7enc.32.A, 7enn.1.A, 7epk.1.A, 7epu.1.B, 7es4.1.A, 7evn.1.E, 7evo.1.L, 7f6j.1.A, 7f6j.1.B, 7fd4.1.A, 7fd4.1.B, 7fd4.1.C, 7fd4.1.D, 7fd4.1.E, 7fd4.1.F, 7fd5.1.A, 7fd5.1.B, 7fd5.1.C, 7fd5.1.D, 7fd5.1.E, 7fd5.1.F, 7fdv.1.A, 7fid.1.A, 7fid.1.B, 7fid.1.C, 7fid.1.D, 7fid.1.E, 7fid.1.F, 7fie.1.A, 7fie.1.B, 7fie.1.C, 7fie.1.D, 7fie.1.E, 7fie.1.F, 7fiz.1.A, 7fiz.1.B, 7fiz.1.C, 7fiz.1.D, 7fiz.1.E, 7fiz.1.F, 7fse.1.A, 7fsf.1.A, 7gqs.1.A, 7gqt.1.A, 7gqu.1.A, 7jg5.1.A, 7jg5.1.B, 7jg5.1.C, 7jg5.1.F, 7jg6.1.A, 7jg6.1.B, 7jg6.1.C, 7jg7.1.A, 7jg7.1.B, 7jg7.1.C, 7jg8.1.B, 7jg8.1.C, 7jg9.1.C, 7jg9.1.F, 7jga.1.A, 7jga.1.B, 7jga.1.C, 7jga.1.F, 7jgr.1.C, 7jgr.1.F, 7jgr.1.G, 7jk2.1.D, 7jk3.1.D, 7jk3.1.G, 7jk5.1.C, 7jk5.1.D, 7jk5.1.F, 7jk6.1.B, 7jk6.1.C, 7jl0.1.C, 7jl1.1.A, 7jlv.1.A, 7jno.1.A, 7jpo.1.A, 7jpo.1.D, 7jpo.1.E, 7jpp.1.D, 7jpp.1.E, 7jpq.1.C, 7jpq.1.D, 7jpr.1.A, 7jpr.1.D, 7jpr.1.E, 7jps.1.A, 7jps.1.D, 7jps.1.E, 7jsh.1.A, 7jsh.1.B, 7jsh.1.C, 7jsh.1.D, 7jsh.1.E, 7jsh.1.F, 7jsh.1.G, 7jy5.1.B, 7k01.1.C, 7k56.1.F, 7k57.1.A, 7kch.1.B, 7ki3.1.A, 7ki3.2.A, 7krn.1.E, 7kro.1.E, 7krz.1.A, 7krz.1.B, 7krz.1.C, 7krz.1.D, 7krz.1.E, 7krz.1.F, 7ks4.1.A, 7ksl.1.A, 7ksl.1.B, 7ksl.1.C, 7ksl.1.D, 7ksl.1.E, 7ksm.1.A, 7ksm.1.B, 7ksm.1.C, 7ksm.1.D, 7ksm.1.E, 7ksm.1.F, 7kx7.1.A, 7kx9.1.A, 7l1q.1.A, 7l1q.1.B, 7l1q.1.C, 7l1q.1.D, 7l1q.1.E, 7l1q.1.F, 7l1r.1.A, 7l1r.1.B, 7l1r.1.D, 7l1r.1.E, 7l1r.1.F, 7l1s.1.C, 7l5w.1.L, 7l5x.1.D, 7l6n.1.B, 7l6n.1.F, 7l9p.1.A, 7l9p.1.B, 7l9p.1.C, 7l9p.1.D, 7l9p.1.E, 7l9p.1.F, 7l9x.1.A, 7lar.1.A, 7lar.1.B, 7lar.1.C, 7lar.1.D, 7lar.1.E, 7lar.1.F, 7lbm.1.0, 7lbm.1.1, 7lcc.1.A, 7liu.1.A, 7ljf.1.A, 7ljf.1.B, 7ljf.1.C, 7ljf.1.F, 7lmy.1.A, 7lmz.1.A, 7lmz.1.B, 7lmz.1.C, 7lmz.1.D, 7lmz.1.E, 7lmz.1.F, 7ln0.1.A, 7ln0.1.B, 7ln0.1.F, 7ln1.1.A, 7ln1.1.B, 7ln1.1.C, 7ln1.1.D, 7ln1.1.E, 7ln1.1.F, 7ln2.1.A, 7ln2.1.B, 7ln2.1.C, 7ln2.1.D, 7ln2.1.E, 7ln2.1.F, 7ln3.1.A, 7ln3.1.B, 7ln3.1.F, 7ln4.1.A, 7ln4.1.B, 7ln4.1.C, 7ln4.1.D, 7ln4.1.E, 7ln4.1.F, 7ln5.1.A, 7ln5.1.B, 7ln5.1.C, 7ln5.1.E, 7ln5.1.F, 7ln6.1.A, 7ln6.1.B, 7ln6.1.C, 7ln6.1.D, 7ln6.1.E, 7ln6.1.F, 7luv.1.F, 7luy.1.A, 7luy.1.B, 7luy.2.B, 7m2u.1.A, 7m2u.1.E, 7m6j.1.A, 7m6j.1.B, 7m6j.1.C, 7m6j.1.D, 7m6j.1.E, 7m6j.1.F, 7m8e.1.F, 7m99.1.E, 7m9a.1.C, 7m9a.1.L, 7m9b.1.A, 7m9b.1.D, 7m9b.1.K, 7m9c.1.B, 7m9c.1.E, 7m9c.1.F, 7m9c.1.G, 7m9c.1.H, 7m9c.1.I, 7m9c.1.J, 7mbw.1.A, 7mbw.1.B, 7mbw.2.A, 7mbw.2.B, 7mca.1.A, 7mca.1.D, 7mca.1.E, 7mca.1.I, 7mcs.1.A, 7mcs.1.B, 7mcs.1.C, 7mcs.1.D, 7mcs.1.E, 7mcs.1.F, 7mcs.1.G, 7md2.1.C, 7md2.1.E, 7md3.1.A, 7md3.1.B, 7md3.1.D, 7md3.1.F, 7mdm.1.A, 7mdm.1.B, 7mdm.1.C, 7mdm.1.D, 7mdm.1.E, 7mdm.1.F, 7mdo.1.A, 7mhb.1.A, 7mhs.1.A, 7mhs.1.B, 7mhs.1.C, 7mhs.1.D, 7mhs.1.E, 7mkn.1.F, 7mkq.1.F, 7ml0.1.1, 7ml0.1.U, 7ml1.1.C, 7ml1.1.G, 7ml2.1.2, 7ml3.1.C, 7ml3.1.H, 7ml4.1.Q, 7ml4.1.U, 7mq8.38.A, 7mq8.39.A, 7mq9.33.A, 7mqa.43.A, 7mqj.1.A, 7mr0.1.A, 7mr1.1.A, 7mr1.1.C, 7mr2.1.A, 7mr3.1.A, 7mr3.1.C, 7mr4.1.A, 7nac.1.v, 7nep.1.P, 7nep.1.S, 7nfx.1.q, 7nfy.1.A, 7nfy.1.B, 7nfy.1.C, 7nfy.1.D, 7nfy.1.E, 7nfy.1.F, 7ng4.1.A, 7ng4.1.B, 7ng4.1.C, 7ng4.1.D, 7ng4.1.E, 7ng4.1.F, 7ng5.1.A, 7ng5.1.B, 7ng5.1.C, 7ng5.1.D, 7ng5.1.E, 7ng5.1.F, 7nga.1.C, 7ngc.1.A, 7ngc.1.B, 7ngc.1.C, 7ngc.1.D, 7ngc.1.E, 7ngc.1.F, 7ngf.1.A, 7ngf.1.B, 7ngf.1.C, 7ngf.1.D, 7ngf.1.E, 7ngf.1.F, 7ngl.1.A, 7ngl.1.B, 7ngl.1.C, 7ngl.1.D, 7ngl.1.E, 7ngl.1.F, 7ngp.1.A, 7ngp.1.C, 7ngp.1.D, 7ngp.1.E, 7ngp.1.F, 7ngq.1.A, 7ngq.1.B, 7ngq.1.C, 7ngq.1.D, 7ngq.1.E, 7ngq.1.F, 7nic.1.A, 7nio.1.A, 7nio.1.B, 7niq.1.A, 7njl.1.A, 7njl.1.B, 7njl.1.C, 7njm.1.C, 7njn.1.C, 7njo.1.C, 7njq.1.C, 7nku.1.A, 7nkx.1.U, 7nn0.1.A, 7nn0.2.A, 7nn0.3.A, 7nn0.4.A, 7nqh.37.A, 7nsh.1.K, 7nvv.1.C, 7o41.1.C, 7o41.1.Z, 7o42.1.A, 7o42.1.B, 7o42.1.C, 7o42.1.D, 7o42.1.E, 7o42.1.F, 7o43.1.A, 7o43.1.B, 7o4i.1.H, 7o4j.1.H, 7o4l.1.H, 7o5b.1.C, 7o72.1.A, 7o72.1.H, 7o73.1.H, 7o75.1.H, 7o9f.1.A, 7o9f.2.A, 7o9f.3.A, 7o9g.1.A, 7o9h.1.A, 7o9h.1.B, 7o9i.1.A, 7o9k.40.A, 7o9m.36.A, 7oar.1.A, 7oar.1.B, 7oat.1.B, 7obq.1.F, 7obr.1.p, 7ohp.1.F, 7ohr.1.G, 7ohs.1.J, 7ohv.1.G, 7ohw.1.J, 7ohx.1.I, 7oi6.1.e, 7oi7.1.7, 7oi9.1.7, 7oib.1.7, 7oic.1.a, 7oid.1.8, 7oie.1.8, 7ole.1.A, 7ole.1.B, 7ole.1.C, 7ole.1.D, 7ole.1.E, 7ole.1.F, 7oo3.1.Q, 7oob.1.M, 7oop.1.X, 7opc.1.X, 7oqb.1.T, 7oqh.1.A, 7oqh.1.B, 7oqh.1.C, 7oqh.1.D, 7oqh.1.E, 7oqh.1.F, 7os1.1.A, 7os2.1.A, 7otj.1.A, 7otj.2.A, 7otq.1.A, 7ovb.1.A, 7owe.1.A, 7owe.1.B, 7owe.1.C, 7owe.2.A, 7owe.2.B, 7owe.2.C, 7owh.1.A, 7owh.1.B, 7owh.1.C, 7owh.2.A, 7owh.2.C, 7owj.1.B, 7owj.1.C, 7owj.2.B, 7owk.1.A, 7owk.1.B, 7owk.1.C, 7owk.2.A, 7owk.2.B, 7owk.2.C, 7owl.1.C, 7owl.2.A, 7oxo.1.A, 7oxo.1.B, 7oxo.1.C, 7oxo.1.D, 7oxo.1.E, 7oxo.1.F, 7p09.1.A, 7p09.1.B, 7p09.1.C, 7p09.1.D, 7p09.1.E, 7p09.1.F, 7p0b.1.A, 7p0b.1.B, 7p0b.1.C, 7p0b.1.D, 7p0b.1.E, 7p0b.1.F, 7p0m.1.A, 7p0m.1.B, 7p0m.1.C, 7p0m.1.D, 7p0m.1.E, 7p0m.1.F, 7p2y.1.D, 7p2y.1.E, 7p2y.1.F, 7p3n.1.D, 7p3n.1.E, 7p3n.1.F, 7p3w.1.D, 7p3w.1.E, 7p3w.1.F, 7p6u.1.A, 7p6u.1.B, 7p6u.1.C, 7p6u.1.D, 7p6u.1.E, 7p6u.1.F, 7p6x.1.A, 7p6x.1.B, 7p6x.1.C, 7p6x.1.D, 7p6x.1.E, 7p6x.1.F, 7pbl.1.A, 7pbl.1.B, 7pbl.1.C, 7pbl.1.D, 7pbl.1.E, 7pbl.1.F, 7pbm.1.E, 7pbo.1.D, 7pbo.1.E, 7pbp.1.D, 7pbp.1.F, 7pbq.1.D, 7pbq.1.E, 7pbr.1.D, 7pbt.1.C, 7pbt.1.E, 7pd3.1.b, 7plh.1.A, 7pli.1.C, 7pli.2.A, 7pli.2.C, 7plt.1.A, 7plw.1.B, 7plx.1.B, 7pm0.1.B, 7pm1.1.B, 7pm2.1.B, 7pm5.1.B, 7pm7.1.B, 7pm8.1.A, 7pm9.1.B, 7pma.1.B, 7pmb.1.B, 7pmc.1.B, 7pmf.1.B, 7pmg.1.A, 7pmi.1.A, 7pmj.1.B, 7pml.1.B, 7pmm.1.B, 7pmq.1.A, 7pmq.2.B, 7ppj.1.A, 7ppz.1.B, 7pq0.1.B, 7pua.19.A, 7pub.19.A, 7pux.1.F, 7px3.1.A, 7px9.1.A, 7px9.1.C, 7px9.1.D, 7px9.1.E, 7px9.1.F, 7pxc.1.G, 7pxc.1.H, 7pxc.1.I, 7pxc.1.K, 7qdr.1.A, 7qdz.1.A, 7qe0.1.A, 7qh6.1.4, 7qh7.1.5, 7qin.1.L, 7qo4.1.B, 7qo4.1.C, 7qo4.1.D, 7qo4.1.E, 7qo4.1.F, 7qo4.1.G, 7qo5.1.f, 7qo5.1.g, 7qo5.1.h, 7qo5.1.i, 7qo5.1.j, 7qo5.1.k, 7qo6.1.f, 7qo6.1.g, 7qo6.1.h, 7qo6.1.i, 7qo6.1.j, 7qo6.1.k, 7qtt.1.K, 7qv8.1.A, 7qv9.1.H, 7qv9.1.I, 7qv9.1.J, 7qv9.1.K, 7qv9.1.L, 7qv9.1.M, 7qwq.1.F, 7qxm.1.A, 7qxm.1.B, 7qxm.1.E, 7qxm.1.F, 7qxp.1.N, 7qxu.1.N, 7qxu.1.O, 7qxw.1.L, 7qxw.1.M, 7qxw.1.N, 7qy7.1.M, 7qy7.1.N, 7qy7.1.O, 7qy7.1.P, 7qy7.1.Q, 7qy7.1.R, 7qya.1.M, 7qya.1.N, 7qya.1.O, 7qya.1.P, 7qya.1.Q, 7qya.1.R, 7r2k.1.A, 7r6q.1.B, 7r76.1.A, 7r77.1.A, 7r78.1.A, 7r7j.1.A, 7r7j.2.A, 7r7s.1.A, 7r7s.1.B, 7r7s.1.C, 7r7s.1.D, 7r7s.1.E, 7r7s.1.F, 7r7t.1.A, 7r7t.1.B, 7r7t.1.C, 7r7t.1.D, 7r7t.1.E, 7r7t.1.F, 7r7u.1.A, 7r7u.1.B, 7r7u.1.C, 7r7u.1.D, 7r7u.1.E, 7r7u.1.F, 7r8d.1.A, 7r8e.1.A, 7r91.1.D, 7r97.1.A, 7rav.1.A, 7rb8.1.B, 7rb9.1.B, 7rdx.1.E, 7rdx.1.F, 7rdy.1.E, 7rdy.1.F, 7rdz.1.E, 7rdz.1.F, 7re0.1.E, 7re0.1.F, 7re1.1.E, 7re1.1.F, 7re2.1.E, 7re3.1.E, 7re3.1.N, 7rl6.1.A, 7rl7.1.A, 7rl9.1.A, 7rla.1.A, 7rlb.1.A, 7rlc.1.A, 7rld.1.A, 7rlf.1.A, 7rlg.1.A, 7rlh.1.A, 7rli.1.A, 7rlj.1.A, 7rzy.1.A, 7s65.1.A, 7s65.1.B, 7s65.1.C, 7s66.1.A, 7s7b.1.A, 7s7c.1.A, 7s9v.1.A, 7s9w.1.A, 7sgz.1.A, 7sgz.1.B, 7sgz.1.C, 7sgz.1.D, 7sgz.1.E, 7sh2.1.A, 7sh2.1.B, 7sh2.1.C, 7sh2.1.E, 7sjr.1.A, 7sjr.1.B, 7sql.1.A, 7sql.1.B, 7sql.1.C, 7sql.1.D, 7ssg.1.A, 7stb.1.B, 7stb.1.C, 7stb.1.D, 7stb.1.E, 7ste.1.A, 7ste.1.B, 7ste.1.C, 7ste.1.D, 7suk.62.A, 7suk.63.A, 7sva.1.A, 7svu.1.R, 7svv.1.K, 7swf.1.A, 7swl.1.A, 7swl.1.B, 7swl.1.D, 7swl.1.E, 7swl.1.F, 7swq.1.A, 7t02.1.A, 7t20.1.A, 7t20.1.B, 7t20.1.C, 7t20.1.D, 7t20.1.E, 7t20.1.F, 7t21.1.A, 7t21.1.B, 7t21.1.C, 7t21.1.D, 7t21.1.E, 7t21.1.F, 7t22.1.A, 7t22.1.B, 7t22.1.C, 7t22.1.D, 7t22.1.E, 7t22.1.F, 7t23.1.A, 7t23.1.B, 7t23.1.D, 7t23.1.E, 7t23.1.F, 7t3i.1.A, 7t3i.1.B, 7t3i.1.C, 7t3i.1.D, 7t3i.1.E, 7t3i.1.F, 7tbm.98.A, 7tdo.1.A, 7tfh.1.A, 7tfh.1.B, 7tfh.1.C, 7tfh.1.D, 7tfh.1.E, 7tfi.1.A, 7tfi.1.B, 7tfi.1.D, 7tfi.1.E, 7tfj.1.A, 7tfj.1.C, 7tfj.1.E, 7tfk.1.A, 7tfk.1.B, 7tfk.1.C, 7tfk.1.D, 7tfk.1.E, 7tfl.1.A, 7tfl.1.B, 7tfl.1.C, 7tfl.1.D, 7tfl.1.E, 7thj.1.A, 7thj.1.C, 7thj.1.D, 7thj.1.E, 7thv.1.A, 7thv.1.C, 7thv.1.D, 7thv.1.E, 7ti8.1.A, 7ti8.1.E, 7tib.1.A, 7tib.1.C, 7tib.1.E, 7tic.1.A, 7tic.1.B, 7tic.1.C, 7tic.1.D, 7tic.1.E, 7tid.1.A, 7tid.1.B, 7tjf.1.A, 7tjf.1.D, 7tjh.1.A, 7tjh.1.E, 7tjh.1.I, 7tji.1.E, 7tji.1.I, 7tjj.1.I, 7tju.1.A, 7tju.1.B, 7tju.1.D, 7tjv.1.E, 7tjw.1.C, 7tjw.1.E, 7tjx.1.B, 7tjx.1.E, 7tk0.1.K, 7tk1.1.K, 7tk1.1.N, 7tk1.1.O, 7tk2.1.K, 7tk2.1.L, 7tk5.1.K, 7tk5.1.L, 7tk5.1.M, 7tk5.1.N, 7tk5.1.O, 7tk5.1.P, 7tk7.1.N, 7tk7.1.O, 7tk9.1.L, 7tka.1.N, 7tkb.1.K, 7tkb.1.M, 7tkc.1.N, 7tkd.1.K, 7tkd.1.L, 7tkd.1.M, 7tkd.1.N, 7tkd.1.O, 7tkd.1.P, 7tke.1.L, 7tke.1.N, 7tke.1.O, 7tkf.1.K, 7tkf.1.M, 7tkg.1.K, 7tki.1.K, 7tki.1.L, 7tki.1.M, 7tki.1.O, 7tki.1.P, 7tkj.1.K, 7tkj.1.L, 7tkj.1.M, 7tkj.1.N, 7tkj.1.O, 7tkj.1.P, 7tkk.1.K, 7tkk.1.L, 7tkk.1.M, 7tkk.1.N, 7tkk.1.O, 7tkk.1.P, 7tkl.1.L, 7tkl.1.M, 7tkm.1.P, 7tkn.1.K, 7tkn.1.L, 7tkn.1.M, 7tkn.1.N, 7tkn.1.O, 7tkn.1.P, 7tkp.1.K, 7tkr.1.K, 7tkr.1.L, 7tkr.1.N, 7tkr.1.O, 7tkr.1.P, 7tks.1.K, 7tks.1.L, 7tks.1.M, 7tks.1.N, 7tks.1.O, 7tks.1.P, 7tku.1.A, 7tku.1.B, 7tku.1.C, 7tku.1.E, 7tle.1.A, 7tlg.1.A, 7tn2.1.K, 7tnx.1.A, 7tny.1.A, 7tnz.1.A, 7to0.1.A, 7to1.1.A, 7to2.1.A, 7tr8.1.A, 7tr9.1.P, 7tra.1.A, 7ttr.1.A, 7ttr.1.B, 7ttr.1.C, 7ttr.1.D, 7ttr.1.E, 7ttr.1.F, 7u19.1.A, 7u19.1.D, 7u19.1.E, 7u1a.1.A, 7u1a.1.C, 7u1a.1.D, 7u1a.1.E, 7u1p.1.A, 7u1p.1.B, 7u1p.1.C, 7udt.1.E, 7udu.1.D, 7ufi.1.C, 7ufm.1.A, 7uiv.1.A, 7uiv.1.B, 7uiv.1.C, 7uiv.1.D, 7uiv.1.E, 7uiv.1.F, 7uiw.1.A, 7uiw.1.B, 7uiw.1.C, 7uiw.1.D, 7uiw.1.E, 7uiw.1.F, 7uix.1.A, 7uix.1.B, 7uix.1.C, 7uix.1.D, 7uix.1.E, 7uix.1.F, 7uiy.1.A, 7uiy.1.B, 7uiy.1.C, 7uiy.1.D, 7uiy.1.E, 7uiy.1.F, 7uiz.1.A, 7uiz.1.B, 7uiz.1.C, 7uiz.1.D, 7uiz.1.E, 7uiz.1.F, 7uj0.1.A, 7uj0.1.B, 7uj0.1.C, 7uj0.1.D, 7uj0.1.E, 7uj0.1.F, 7ujb.1.A, 7upr.1.A, 7upr.1.B, 7upr.1.C, 7upr.1.E, 7upr.1.F, 7upt.1.A, 7upt.1.B, 7upt.1.C, 7upt.1.D, 7upt.1.E, 7upt.1.F, 7uqi.1.A, 7uqi.1.B, 7uqi.1.C, 7uqi.1.D, 7uqi.1.E, 7uqi.1.F, 7uqj.1.A, 7uqj.1.B, 7uqj.1.C, 7uqj.1.D, 7uqj.1.E, 7uqj.1.F, 7us2.1.A, 7us2.1.F, 7ux9.1.I, 7v2b.1.A, 7v2b.1.B, 7v2v.1.A, 7v2v.1.B, 7v2y.1.F, 7v2z.1.A, 7v3w.1.A, 7v3w.1.B, 7v4e.1.A, 7v4e.1.B, 7v4q.1.A, 7v4r.1.A, 7v6b.1.A, 7v6b.1.B, 7v6c.1.A, 7v6c.1.B, 7vcs.1.A, 7vct.1.A, 7vcu.1.A, 7vcv.1.A, 7vcx.1.A, 7vdt.1.A, 7vdv.1.J, 7vg2.1.A, 7vg3.1.A, 7vsr.1.A, 7vsr.1.B, 7vsr.1.C, 7vsr.1.D, 7vsr.1.E, 7vsr.1.F, 7w0a.1.A, 7w0b.1.A, 7w0c.1.A, 7w0d.1.D, 7w0d.1.E, 7w0e.1.C, 7w0f.1.A, 7w1r.1.A, 7w37.1.A, 7w37.1.D, 7w38.1.B, 7w38.1.C, 7w38.1.D, 7w38.1.j, 7w39.1.A, 7w39.1.B, 7w39.1.C, 7w39.1.D, 7w39.1.E, 7w39.1.F, 7w3a.1.A, 7w3a.1.B, 7w3a.1.C, 7w3a.1.D, 7w3a.1.E, 7w3a.1.F, 7w3b.1.A, 7w3b.1.B, 7w3b.1.C, 7w3b.1.D, 7w3b.1.E, 7w3b.1.F, 7w3c.1.A, 7w3c.1.B, 7w3c.1.C, 7w3c.1.D, 7w3c.1.E, 7w3c.1.F, 7w3f.1.A, 7w3f.1.B, 7w3f.1.C, 7w3f.1.D, 7w3f.1.E, 7w3f.1.F, 7w3g.1.A, 7w3g.1.B, 7w3g.1.C, 7w3g.1.D, 7w3g.1.E, 7w3g.1.F, 7w3h.1.A, 7w3h.1.B, 7w3h.1.C, 7w3h.1.D, 7w3h.1.E, 7w3h.1.F, 7w3i.1.B, 7w3i.1.C, 7w3i.1.D, 7w3i.1.E, 7w3i.1.F, 7w3i.1.G, 7w3j.1.6, 7w3j.1.7, 7w3j.1.8, 7w3j.1.9, 7w3j.1.A, 7w3j.1.B, 7w3k.1.8, 7w3k.1.9, 7w3k.1.A, 7w3k.1.B, 7w3k.1.C, 7w3k.1.D, 7w3m.1.A, 7w3m.1.B, 7w3m.1.C, 7w3m.1.D, 7w3m.1.E, 7w3m.1.F, 7w59.1.Y, 7w5a.1.Y, 7w5b.1.Y, 7wbb.1.A, 7wbb.1.B, 7wbb.1.C, 7wbb.1.D, 7wbb.1.E, 7wbb.1.G, 7wbt.1.A, 7wbt.1.B, 7wd3.1.A, 7wd3.1.B, 7wd3.1.C, 7wd3.1.E, 7wd3.1.F, 7wd4.1.A, 7wdc.1.A, 7wi4.1.A, 7wi4.1.B, 7wi4.1.C, 7wi4.1.D, 7wi4.1.E, 7wi4.1.F, 7wub.1.B, 7wub.1.C, 7wub.1.D, 7wub.1.J, 7x1y.1.A, 7x1z.1.A, 7x2r.1.A, 7x2r.1.B, 7x2r.1.C, 7x2r.1.D, 7x2r.1.E, 7x2r.1.F, 7x3t.1.T, 7x5b.1.A, 7x7p.1.C, 7x7p.1.E, 7x7p.1.G, 7x7p.1.I, 7x7q.1.I, 7x7q.1.J, 7x7q.1.K, 7x7q.1.L, 7x7s.1.A, 7xex.1.A, 7xex.2.A, 7xex.3.A, 7xf0.1.A, 7xf0.2.A, 7xf0.3.A, 7xf1.1.A, 7xg3.1.L, 7xha.1.A, 7xhb.1.A, 7xkh.1.A, 7xkh.1.B, 7xkh.1.C, 7xkh.1.D, 7xkh.1.E, 7xkh.1.F, 7xko.1.B, 7xko.1.C, 7xko.1.D, 7xko.1.E, 7xko.1.F, 7xkp.1.A, 7xkp.1.B, 7xkp.1.C, 7xkp.1.D, 7xkp.1.E, 7xkq.1.A, 7xkq.1.B, 7xkq.1.C, 7xkq.1.D, 7xkq.1.E, 7xkr.1.A, 7xkr.1.C, 7xkr.1.D, 7xt0.1.A, 7xt3.1.A, 7xw2.1.A, 7xw3.1.A, 7xwy.1.A, 7xxe.1.A, 7xxe.2.A, 7xyf.1.G, 7xyg.1.G, 7y4w.1.E, 7y4w.1.F, 7y4w.1.G, 7y4w.1.H, 7y4w.1.I, 7y4w.1.J, 7y53.1.E, 7y53.1.F, 7y53.1.G, 7y53.1.H, 7y53.1.I, 7y53.1.J, 7y59.1.E, 7y59.1.F, 7y59.1.G, 7y59.1.H, 7y59.1.I, 7y59.1.J, 7y5a.1.A, 7y5a.1.B, 7y5a.1.C, 7y5a.1.D, 7y5a.1.E, 7y5a.1.F, 7y5b.1.A, 7y5b.1.B, 7y5b.1.C, 7y5b.1.D, 7y5b.1.E, 7y5b.1.F, 7y5c.1.A, 7y5c.1.B, 7y5c.1.C, 7y5d.1.M, 7y5d.1.N, 7y5d.1.O, 7y8r.1.J, 7yfq.1.C, 7yfx.1.A, 7yfy.1.A, 7yg6.1.B, 7ygn.1.A, 7ykk.1.A, 7ykk.1.B, 7ykk.1.C, 7ykk.1.D, 7ykk.1.E, 7ykk.1.F, 7ykl.1.A, 7ykl.1.B, 7ykl.1.C, 7ykl.1.D, 7ykl.1.E, 7ykl.1.F, 7ykt.1.A, 7ykt.1.B, 7ykt.1.C, 7ykt.1.D, 7ykt.1.E, 7ykt.1.F, 7ykz.1.A, 7ykz.1.B, 7ykz.1.C, 7ykz.1.D, 7ykz.1.E, 7ykz.1.F, 7ymf.1.A, 7ymf.1.B, 7yph.1.A, 7yph.1.B, 7yph.1.C, 7yph.1.D, 7yph.1.E, 7ypi.1.A, 7ypi.1.B, 7ypi.1.C, 7ypi.1.D, 7ypi.1.E, 7ypi.1.F, 7ypj.1.A, 7ypj.1.B, 7ypj.1.C, 7ypj.1.D, 7ypj.1.E, 7ypk.1.B, 7ypk.1.C, 7ypk.1.D, 7ypk.1.E, 7ypk.1.F, 7ypk.1.G, 7yuh.1.A, 7yuh.1.B, 7yuh.1.C, 7yum.1.A, 7yum.1.B, 7yum.1.C, 7yum.1.D, 7yup.1.A, 7yup.1.B, 7yup.1.C, 7yup.1.D, 7yup.1.E, 7yut.1.A, 7yut.1.B, 7yut.1.C, 7yut.1.D, 7yut.1.E, 7yut.1.F, 7yuu.1.A, 7yuu.1.B, 7yuu.1.C, 7yuv.1.A, 7yuv.1.B, 7yuv.1.C, 7yuv.1.D, 7yuw.1.A, 7yuw.1.B, 7yuw.1.C, 7yuw.1.D, 7yuw.1.E, 7yux.1.A, 7yux.1.B, 7yux.1.C, 7yux.1.D, 7yux.1.E, 7yux.1.F, 7yym.1.A, 7yyn.1.B, 7yz4.1.A, 7z11.1.A, 7z11.1.B, 7z11.1.C, 7z11.1.D, 7z11.1.E, 7z11.1.F, 7z34.1.A, 7z34.1.h, 7z34.1.i, 7z34.1.n, 7z34.1.q, 7z34.1.r, 7z4y.1.B, 7z4y.1.D, 7z52.1.A, 7z6h.1.D, 7z6h.1.E, 7z6h.1.F, 7z6h.1.G, 7z6h.1.H, 7z7n.1.D, 7z8s.1.D, 7zb5.1.D, 7zbh.1.A, 7zel.1.A, 7zep.1.A, 7zi4.1.A, 7zi4.1.B, 7zi4.1.C, 7zi4.1.D, 7zi4.1.E, 7zi4.1.F, 7zi4.1.G, 7zj1.1.A, 7zj1.1.B, 7zke.1.D, 7zlq.1.A, 7zmm.2.A, 7zmm.3.A, 7zmm.4.A, 7zmn.1.A, 7zmo.1.A, 7zmp.1.A, 7zmp.2.A, 7zmq.2.A, 7zmr.1.A, 7zms.1.A, 7zmt.1.A, 7zmv.2.A, 7znj.1.A, 7znk.1.G, 7zpi.1.A, 7zpj.1.A, 7zpj.1.C, 7zpk.1.C, 7zpq.75.A, 7zrs.75.A, 7zsa.1.3, 7zsb.1.3, 7zuw.75.A, 8a22.73.A, 8a3v.1.A, 8a3v.1.H, 8a8u.1.A, 8a8u.1.B, 8a8u.1.C, 8a8u.1.D, 8a8u.1.E, 8a8u.1.F, 8a8v.1.A, 8a8v.1.B, 8a8v.1.C, 8a8v.1.D, 8a8v.1.E, 8a8v.1.F, 8a8w.1.A, 8a8w.1.B, 8a8w.1.C, 8a8w.1.D, 8a8w.1.E, 8a8w.1.F, 8act.1.A, 8act.1.B, 8alz.1.B, 8amz.1.A, 8amz.1.B, 8amz.1.C, 8amz.1.D, 8amz.1.E, 8amz.1.F, 8apo.73.A, 8ark.1.A, 8ark.2.A, 8ark.3.A, 8arp.1.A, 8arp.1.B, 8arp.1.C, 8arp.1.D, 8arp.1.E, 8arp.1.F, 8atf.1.A, 8av6.1.G, 8b02.1.A, 8b0a.1.A, 8b1r.1.A, 8b1r.1.C, 8b1t.1.A, 8b1t.1.C, 8b3d.1.R, 8b3f.1.R, 8b5r.1.A, 8b5r.1.B, 8b5r.1.C, 8b5r.1.D, 8b5r.1.E, 8b5r.1.F, 8b9g.1.A, 8b9i.1.A, 8b9j.1.A, 8b9k.1.A, 8b9l.1.A, 8bc8.1.A, 8bc9.1.A, 8bca.1.A, 8bcb.1.A, 8bcc.1.A, 8bcd.1.A, 8bce.1.A, 8bcf.1.A, 8bcg.1.A, 8bch.1.A, 8bd4.1.B, 8bd5.1.E, 8bd5.1.F, 8bd5.1.G, 8bd5.1.H, 8bd5.1.I, 8bd5.1.J, 8bns.1.A, 8bns.1.B, 8bns.2.A, 8bns.2.B, 8bnv.1.A, 8bnx.1.A, 8bnx.2.A, 8bob.1.C, 8bq2.1.H, 8br9.1.A, 8bsc.1.G, 8btg.1.A, 8btg.1.B, 8btg.1.C, 8btg.1.D, 8btg.1.E, 8btg.1.F, 8btg.1.G, 8bv3.1.A, 8bvw.1.A, 8bvw.1.B, 8byq.1.A, 8byq.1.B, 8c0v.1.A, 8c0v.1.B, 8c0v.1.C, 8c0v.1.D, 8c0v.1.E, 8c0v.1.F, 8c0w.1.A, 8c0w.1.B, 8c0w.1.C, 8c0w.1.D, 8c0w.1.E, 8c0w.1.F, 8c3j.2.A, 8c3n.1.A, 8c6j.2.A, 8c6j.30.A, 8c6j.6.A, 8c6j.9.A, 8cen.1.H, 8ceo.1.A, 8ch6.1.9, 8ch6.1.U, 8ch6.1.v, 8cnt.1.A, 8cvt.1.A, 8cvt.1.B, 8cvt.1.C, 8cvt.1.D, 8cvt.1.E, 8cvt.1.F, 8d6j.1.A, 8d6x.1.A, 8d6x.1.B, 8d6x.1.C, 8d6x.1.D, 8d6x.1.E, 8d6x.1.F, 8d6y.1.A, 8d6y.1.B, 8d6y.1.C, 8d6y.1.D, 8d6y.1.E, 8d6y.1.F, 8d71.1.A, 8d8k.1.O, 8das.1.A, 8das.1.B, 8das.1.C, 8das.1.D, 8das.1.E, 8das.1.F, 8dau.1.A, 8dau.1.B, 8dau.1.C, 8dau.1.D, 8dau.1.E, 8dau.1.F, 8db3.1.A, 8db3.1.B, 8db3.1.C, 8dba.1.A, 8dba.1.B, 8dba.1.C, 8dba.1.D, 8dba.1.E, 8dba.1.F, 8dba.1.G, 8dba.1.H, 8dba.1.I, 8dba.1.J, 8dba.1.K, 8dba.1.L, 8dbw.1.A, 8dbw.1.B, 8dbw.1.C, 8dfv.1.A, 8dfv.1.C, 8dg5.1.A, 8dg5.1.C, 8dg7.1.A, 8dg7.1.D, 8dga.1.A, 8dga.1.D, 8dgc.1.E, 8dgi.1.A, 8dgi.1.B, 8dgj.1.A, 8dgj.1.B, 8dkt.1.A, 8dol.1.A, 8dol.1.B, 8dol.1.C, 8dol.1.D, 8dol.1.E, 8dol.1.F, 8dpe.1.A, 8dqw.1.A, 8dqw.1.B, 8dqw.1.D, 8dqx.1.A, 8dqx.1.B, 8dqx.1.E, 8dqz.1.A, 8dqz.1.B, 8dr0.1.A, 8dr0.1.C, 8dr0.1.D, 8dr1.1.A, 8dr1.1.B, 8dr3.1.B, 8dr3.1.C, 8dr4.1.A, 8dr4.1.B, 8dr4.1.C, 8dr6.1.E, 8dr7.1.A, 8dr7.1.B, 8dr7.1.E, 8dtp.1.A, 8dtp.1.C, 8dtp.1.D, 8dtp.1.E, 8dtp.1.F, 8dtp.1.G, 8due.1.A, 8due.1.B, 8due.1.C, 8due.1.D, 8due.1.E, 8due.1.F, 8duo.1.A, 8duo.1.B, 8duo.1.C, 8duo.1.D, 8duo.1.E, 8duo.1.F, 8dvs.1.A, 8dze.1.A, 8dzf.1.A, 8dzg.1.A, 8e0f.1.B, 8e2l.1.A, 8e2l.1.C, 8e2l.1.G, 8e2w.1.A, 8e7v.1.C, 8e7v.1.E, 8e7v.1.F, 8e8q.1.A, 8e8q.1.C, 8e91.1.A, 8e91.1.B, 8e91.1.C, 8e91.1.D, 8e91.1.E, 8e91.1.F, 8ea4.1.C, 8ea4.1.I, 8ea4.1.J, 8ea4.1.M, 8ebs.1.B, 8ebt.1.A, 8ebt.1.B, 8ebu.1.A, 8ebu.1.B, 8ebw.1.A, 8efd.1.A, 8efe.1.A, 8efh.1.A, 8efv.1.A, 8efv.1.F, 8efy.1.A, 8efy.1.B, 8efy.1.C, 8efy.1.D, 8efy.1.E, 8efy.1.F, 8ejm.1.A, 8emc.1.A, 8emc.1.H, 8emc.1.I, 8emc.1.J, 8emc.1.K, 8emc.1.L, 8emc.1.M, 8emc.1.N, 8emh.1.A, 8emh.1.B, 8emh.1.C, 8emh.1.D, 8emh.1.E, 8emh.1.F, 8emh.1.G, 8emh.1.H, 8emh.1.I, 8emh.1.J, 8emh.1.K, 8emh.1.L, 8enk.1.A, 8enk.1.B, 8esr.1.I, 8et3.1.A, 8et3.1.B, 8et3.1.C, 8et3.1.D, 8et3.1.E, 8et3.1.F, 8etr.1.A, 8ets.1.A, 8ets.1.D, 8ets.1.E, 8ets.1.F, 8ets.1.G, 8ets.1.H, 8ets.1.I, 8etw.1.A, 8eu9.1.A, 8eu9.1.D, 8eu9.1.E, 8eu9.1.F, 8eu9.1.G, 8eu9.1.H, 8eu9.1.I, 8euf.1.A, 8euf.1.D, 8euf.1.E, 8euf.1.F, 8euf.1.G, 8euf.1.H, 8euf.1.I, 8eup.1.J, 8exw.1.A, 8f29.1.0, 8f29.1.V, 8f29.1.W, 8f29.1.X, 8f29.1.Y, 8f29.1.Z, 8f2k.1.A, 8f2k.1.C, 8f2k.1.D, 8f2k.1.E, 8f39.1.T, 8f39.1.U, 8f39.1.V, 8f39.1.W, 8f39.1.X, 8f39.1.Y, 8fak.1.D, 8faz.1.B, 8faz.1.C, 8faz.1.D, 8fcl.1.A, 8fcl.1.B, 8fcl.1.C, 8fcl.1.D, 8fcl.1.E, 8fcm.1.D, 8fcm.1.E, 8fcm.1.G, 8fcn.1.L, 8fco.1.D, 8fco.1.E, 8fco.1.G, 8fcp.1.A, 8fcp.1.C, 8fcp.1.F, 8fcq.1.B, 8fcq.1.C, 8fcr.1.D, 8fcr.1.E, 8fct.1.A, 8fct.1.B, 8fct.1.D, 8fct.1.F, 8fcu.1.Q, 8fcv.1.E, 8fcv.1.F, 8fcv.1.G, 8fcv.1.I, 8fcv.1.J, 8fcw.1.D, 8fkj.1.B, 8fkj.1.C, 8fkj.1.D, 8fkj.1.E, 8fkj.1.F, 8fks.1.j, 8fkt.1.0, 8fl8.1.D, 8fl8.1.E, 8fl8.1.F, 8fl8.1.G, 8fl8.1.H, 8fl8.1.I, 8flj.1.M, 8flj.1.N, 8fml.1.A, 8fs3.1.A, 8fs3.1.B, 8fs3.1.E, 8fs4.1.A, 8fs4.1.C, 8fs4.1.E, 8fs5.1.A, 8fs6.1.A, 8fs6.1.B, 8fs6.1.E, 8fs7.1.A, 8fs7.1.C, 8fs7.1.E, 8fs8.1.B, 8fs8.1.C, 8fs8.1.D, 8fs8.1.E, 8fth.1.A, 8ftk.1.A, 8ftm.1.A, 8ftm.2.A, 8fvu.1.A, 8fvu.1.B, 8fw2.1.A, 8fw9.1.A, 8fw9.1.B, 8fw9.1.C, 8g09.1.J, 8g0z.1.A, 8g0z.1.B, 8g0z.1.C, 8g0z.1.D, 8g0z.1.E, 8g0z.1.F, 8g7t.1.A, 8g7t.1.C, 8g7u.1.A, 8g7u.1.C, 8g7v.1.A, 8g7v.1.C, 8g9u.1.A, 8gbj.1.B, 8gbj.1.C, 8gbj.1.D, 8giy.1.B, 8giy.1.C, 8giy.1.D, 8giy.1.E, 8giz.1.B, 8giz.1.C, 8giz.1.D, 8giz.1.E, 8gj0.1.B, 8gj0.1.C, 8gj0.1.D, 8gj0.1.E, 8gj1.1.B, 8gj1.1.C, 8gj1.1.D, 8gj1.1.E, 8gj2.1.B, 8gj2.1.C, 8gj2.1.D, 8gj2.1.E, 8gj3.1.B, 8gj3.1.C, 8gj3.1.D, 8gj3.1.E, 8gj8.1.A, 8gja.1.A, 8gja.1.B, 8gja.2.B, 8gja.3.A, 8gja.3.B, 8gju.1.B, 8gju.2.B, 8gju.2.C, 8glu.1.A, 8glu.1.C, 8glu.1.D, 8glv.468.A, 8glv.844.A, 8glv.902.A, 8glv.974.A, 8glw.1.A, 8glw.1.B, 8glw.1.C, 8glw.1.E, 8glw.1.F, 8glw.1.G, 8glw.1.H, 8glx.1.A, 8glx.1.E, 8glx.1.F, 8glx.1.G, 8glx.1.I, 8glx.1.J, 8gme.1.B, 8gme.2.B, 8gw1.1.G, 8gw1.1.H, 8gwb.1.E, 8gwb.1.F, 8gwe.1.E, 8gwe.1.F, 8gwg.1.G, 8gwg.1.H, 8gwk.1.G, 8gwk.1.H, 8gwm.1.E, 8gwm.1.F, 8gwn.1.G, 8gwn.1.H, 8gwo.1.G, 8gwo.1.H, 8gyk.1.A, 8gyk.1.B, 8gyk.1.C, 8gyk.1.E, 8gyk.1.F, 8gyk.1.H, 8gzq.1.B, 8gzr.1.B, 8h3h.1.A, 8h3h.1.B, 8h3h.1.C, 8h3h.1.D, 8h3h.1.E, 8h3h.1.F, 8h5y.2.A, 8h5z.1.A, 8h5z.2.A, 8h6e.1.M, 8h6j.1.5, 8h6j.1.M, 8h6l.1.E, 8h9i.1.C, 8h9p.1.A, 8h9u.1.C, 8h9v.1.Y, 8he5.1.O, 8hf0.1.A, 8hf0.1.D, 8hf1.1.A, 8hf1.1.D, 8hf1.1.F, 8hh1.1.A, 8hh3.1.D, 8hh4.1.A, 8hh4.1.B, 8hh4.1.C, 8hh5.1.D, 8hh5.1.E, 8hh8.1.F, 8hh9.1.D, 8hh9.1.E, 8hhc.1.D, 8hl7.1.B, 8hmy.1.F, 8hrz.1.A, 8hrz.1.L, 8huj.1.A, 8i0r.1.R, 8i0s.1.P, 8i0t.1.W, 8i0v.1.V, 8i0w.1.0, 8i0w.1.5, 8i9j.1.B, 8i9j.1.C, 8i9p.1.F, 8i9r.1.F, 8i9t.1.G, 8i9t.1.Y, 8i9v.1.0, 8i9v.1.G, 8i9w.1.F, 8i9w.1.Z, 8i9x.1.0, 8i9y.1.G, 8i9z.1.G, 8i9z.1.Y, 8ia0.23.A, 8ia0.5.A, 8igd.1.A, 8igd.2.A, 8iju.1.A, 8iud.1.A, 8izn.1.A, 8j07.4.A, 8j07.844.A, 8j07.848.A, 8j07.849.A, 8j4t.1.G, 8j4u.1.M, 8j4u.1.N, 8j4u.1.O, 8j4u.1.P, 8j4u.1.Q, 8j4u.1.R, 8j90.1.K, 8jix.1.A, 8jne.1.K, 8jqb.1.E, 8jqc.1.E, 8jr0.1.A, 8jr0.1.B, 8jr0.1.C, 8jr0.1.D, 8jr0.1.E, 8juw.1.A, 8juw.1.B, 8juw.1.C, 8juw.1.D, 8juw.1.E, 8juw.1.F, 8juy.1.A, 8juy.1.B, 8juy.1.C, 8juy.1.D, 8juy.1.E, 8juy.1.F, 8juz.1.A, 8juz.1.E, 8juz.1.F, 8jx6.1.A, 8jx6.2.A, 8k22.1.R, 8k3y.1.A, 8k3y.1.B, 8k3y.1.C, 8k3y.1.D, 8k3y.1.E, 8k3y.1.F, 8kca.1.A, 8kca.2.A, 8kcb.1.K, 8kcc.1.K, 8kfp.1.A, 8kg2.1.A, 8ki3.1.A, 8ki3.1.B, 8ki3.1.C, 8ki3.1.D, 8ki3.1.E, 8ki3.1.F, 8ofb.1.A, 8ohm.1.A, 8ojl.1.A, 8ojl.1.B, 8ojl.1.C, 8ojl.1.D, 8ojl.1.E, 8ojl.1.F, 8oka.1.A, 8oka.1.B, 8oka.1.C, 8oka.1.D, 8oka.1.E, 8oka.1.F, 8om7.1.A, 8om7.1.B, 8om7.1.C, 8om7.1.D, 8om7.1.E, 8om7.1.F, 8oo7.1.C, 8oo7.1.E, 8oo7.1.G, 8ooi.1.C, 8oop.1.A, 8oop.1.G, 8oor.1.B, 8oor.1.D, 8oor.1.F, 8oor.1.G, 8osf.1.A, 8osf.1.B, 8osf.1.C, 8osf.1.D, 8osf.1.E, 8osf.1.F, 8osg.1.A, 8osg.1.B, 8osg.1.C, 8osg.1.D, 8osg.1.E, 8osg.1.F, 8osh.1.A, 8osh.1.B, 8osh.1.C, 8osh.1.D, 8osh.1.E, 8ouy.1.B, 8ouy.1.C, 8ouy.1.D, 8ouz.1.B, 8ouz.1.C, 8ouz.1.D, 8ovf.1.A, 8ovf.1.B, 8ovf.1.C, 8ovf.1.D, 8ovf.1.E, 8ovf.1.F, 8ovg.1.A, 8ovg.1.B, 8ovg.1.C, 8ovg.1.D, 8ovg.1.E, 8ovg.1.F, 8oz0.1.s, 8oz0.1.t, 8pb9.1.A, 8pb9.1.D, 8pb9.1.E, 8pbd.1.F, 8peu.1.A, 8peu.1.B, 8peu.1.C, 8peu.1.D, 8peu.1.E, 8peu.1.F, 8peu.1.G, 8peu.1.H, 8peu.1.I, 8peu.1.J, 8peu.1.K, 8peu.1.L, 8pew.1.A, 8pew.1.B, 8pew.1.C, 8pew.1.D, 8pew.1.E, 8pew.1.F, 8pew.1.G, 8pew.1.H, 8pew.1.I, 8pew.1.J, 8pew.1.K, 8pew.1.L, 8pew.1.M, 8pew.1.N, 8pew.1.O, 8pew.1.P, 8pew.1.Q, 8pew.1.R, 8pex.1.A, 8pex.1.B, 8pex.1.C, 8pex.1.E, 8pex.1.F, 8pex.1.G, 8pex.1.H, 8pex.1.I, 8pex.1.J, 8pex.1.K, 8pex.1.L, 8pey.1.A, 8pey.1.B, 8pey.1.C, 8pey.1.E, 8pey.1.F, 8pey.1.G, 8pey.1.H, 8pey.1.J, 8pey.1.L, 8pey.1.M, 8pfl.1.A, 8pfp.1.A, 8pjb.1.A, 8pjj.1.A, 8pk0.1.Z, 8pnk.1.A, 8po6.1.A, 8po7.1.A, 8po8.1.A, 8po8.1.B, 8pqx.1.A, 8pqx.1.B, 8ptg.1.A, 8ptg.1.B, 8ptg.1.C, 8ptg.1.E, 8ptg.1.F, 8ptm.1.A, 8ptm.1.E, 8ptm.1.F, 8ptn.1.A, 8ptn.1.B, 8ptn.1.C, 8ptn.1.D, 8ptn.1.E, 8ptn.1.F, 8pto.1.A, 8pto.1.E, 8ptp.1.A, 8ptp.1.E, 8pts.1.A, 8pts.1.B, 8q3i.1.G, 8q3n.1.A, 8q3n.1.E, 8q3n.1.F, 8q3o.1.A, 8q3o.1.B, 8q3o.1.C, 8q3o.1.D, 8q3o.1.E, 8q3o.1.F, 8q3p.1.A, 8q3q.1.A, 8q3w.1.A, 8q3w.1.B, 8q4d.1.E, 8q4d.1.F, 8q4d.1.G, 8q4d.1.H, 8q4d.1.M, 8q4d.1.N, 8q7w.1.F, 8q9t.1.A, 8qcf.1.L, 8qqe.1.A, 8qr1.1.A, 8qr1.1.H, 8qr1.1.I, 8qr1.1.J, 8qr1.1.K, 8qr1.1.L, 8qr1.1.M, 8qyp.1.A, 8qyq.1.A, 8qyq.2.A, 8qyr.1.A, 8qyu.1.A, 8qzs.2.A, 8r08.10.A, 8r0a.1.M, 8r0b.1.B, 8r0e.1.A, 8r2g.1.B, 8r3z.1.A, 8r9r.1.A, 8rc0.1.E, 8rd3.1.A, 8rdu.1.K, 8rdu.1.L, 8rdu.1.M, 8rdu.1.U, 8rdu.1.W, 8rdu.1.X, 8rev.1.A, 8rhn.1.A, 8rhn.1.B, 8rhn.1.C, 8rhn.1.D, 8rhn.1.E, 8rhn.1.F, 8ri2.1.A, 8rm5.1.9, 8rm5.1.k, 8ro0.1.E, 8ro0.1.R, 8ro1.1.G, 8rs9.1.A, 8rsb.1.A, 8rsb.1.B, 8rsb.1.C, 8rsc.1.A, 8rsc.1.B, 8rsc.1.F, 8rtb.1.C, 8rtd.1.5, 8s9i.1.A, 8scz.1.A, 8sd0.1.A, 8sgd.1.A, 8sm3.1.B, 8so8.1.A, 8sp4.1.A, 8spg.1.A, 8spv.1.A, 8spv.1.D, 8spw.1.A, 8spw.1.B, 8spw.1.C, 8spw.1.D, 8spw.1.E, 8spw.1.F, 8spx.1.A, 8spx.1.C, 8spx.1.D, 8spx.1.F, 8ssw.1.A, 8stn.1.A, 8su9.1.M, 8su9.1.P, 8su9.1.Q, 8su9.1.R, 8sub.1.N, 8sub.1.O, 8sub.1.P, 8suw.1.M, 8suw.1.N, 8suw.1.O, 8suw.1.P, 8szp.1.A, 8szp.2.A, 8szq.1.A, 8szr.1.A, 8t14.1.E, 8t5s.1.A, 8t5u.1.A, 8tbj.1.A, 8tbn.1.A, 8tbx.1.A, 8tby.1.D, 8th8.1.L, 8thb.1.A, 8thb.1.B, 8thb.1.C, 8thb.1.D, 8thc.1.A, 8thc.1.B, 8thc.1.C, 8thc.1.D, 8thc.1.E, 8thd.1.A, 8thd.1.E, 8ti0.1.D, 8tid.1.L, 8tjy.1.F, 8tpl.1.A, 8tvy.1.M, 8u0u.1.A, 8u0v.1.A, 8u0v.1.B, 8u0v.1.C, 8u0v.1.D, 8u0v.1.E, 8u0v.1.F, 8u0w.1.A, 8u3k.1.A, 8u7i.1.G, 8u7i.1.H, 8uae.1.M, 8uae.1.N, 8uae.1.O, 8uae.1.P, 8uae.1.Q, 8uae.1.R, 8uaf.1.M, 8uaf.1.N, 8uaf.1.O, 8uaf.1.P, 8uaf.1.Q, 8uaf.1.R, 8uh7.1.E, 8ui7.1.B, 8ui7.1.C, 8ui7.1.D, 8ui7.1.E, 8ui8.1.C, 8ui8.1.D, 8ui8.1.E, 8ui9.1.B, 8ui9.1.C, 8ui9.1.D, 8ui9.1.E, 8uii.1.B, 8uii.1.C, 8uii.1.D, 8uii.1.E, 8uk9.1.B, 8uk9.1.C, 8uk9.1.D, 8uk9.1.E, 8uk9.2.A, 8uk9.2.B, 8uk9.2.C, 8uk9.2.D, 8umt.1.A, 8umt.1.B, 8umt.1.C, 8umt.1.D, 8umt.1.E, 8umu.1.A, 8umu.1.B, 8umu.1.C, 8umu.1.D, 8umu.1.E, 8umv.1.A, 8umv.1.C, 8umv.1.D, 8umv.1.E, 8un0.1.A, 8un0.1.B, 8unf.1.F, 8unf.1.G, 8unf.1.H, 8unf.1.I, 8unh.1.A, 8unh.1.B, 8unj.1.B, 8unj.1.C, 8unj.1.D, 8unj.1.E, 8v44.1.A, 8v4y.1.K, 8v83.1.I, 8v85.1.A, 8v87.1.J, 8val.1.B, 8val.1.C, 8val.1.D, 8val.1.E, 8vam.1.B, 8vam.1.C, 8vam.1.D, 8vam.1.E, 8van.1.B, 8van.1.C, 8van.1.D, 8van.1.E, 8vap.1.B, 8vap.1.C, 8vap.1.D, 8vap.1.E, 8vaq.1.B, 8vaq.1.C, 8vaq.1.D, 8vaq.1.E, 8var.1.B, 8var.1.C, 8var.1.D, 8var.1.E, 8vas.1.B, 8vas.1.C, 8vas.1.D, 8vas.1.E, 8vat.1.B, 8vat.1.C, 8vat.1.D, 8vat.1.E, 8vcj.1.A, 8vcj.1.B, 8vcj.1.C, 8vcj.1.E, 8vcj.1.F, 8vcj.1.G, 8vcj.1.H, 8vct.1.A, 8vct.1.E, 8vct.1.F, 8vct.1.G, 8vct.1.H, 8vct.1.J, 8vku.1.C, 8vls.1.I, 8vov.1.A, 8vx9.1.B, 8vxa.1.C, 8vxc.1.B, 8vxy.1.D, 8w0a.1.A, 8w8d.1.A, 8w8d.1.B, 8w8d.1.D, 8w8d.1.E, 8w8d.1.F, 8wap.1.G, 8wap.1.H, 8wh5.1.K, 8wh8.1.K, 8wh9.1.K, 8wsm.1.A, 8wtk.1.A, 8wv8.1.A, 8wve.1.A, 8x15.1.I, 8x15.1.M, 8x15.1.O, 8x15.1.P, 8x15.1.Q, 8x19.1.I, 8x19.1.O, 8x19.1.Q, 8x19.1.R, 8x1c.1.I, 8x1c.1.M, 8x1c.1.N, 8x1c.1.P, 8x1c.1.Q, 8x1c.1.R, 8x5n.1.B, 8x5n.1.C, 8x5n.1.D, 8x5n.1.E, 8xak.1.A, 8xak.2.A, 8xau.1.A, 8xau.1.B, 8xau.1.D, 8xau.1.E, 8xau.1.F, 8xav.1.A, 8xav.1.C, 8xav.1.E, 8xaw.1.A, 8xaw.1.B, 8xaw.1.C, 8xaw.1.D, 8xaw.1.E, 8xaw.1.F, 8xax.1.A, 8xax.1.B, 8xax.1.D, 8xax.1.E, 8xbt.1.K, 8xbt.1.L, 8xbt.1.M, 8xbt.1.N, 8xbt.1.O, 8xbt.1.P, 8xbt.1.Q, 8xbt.1.R, 8xbx.1.C, 8xms.1.A, 8xon.1.A, 8xon.1.B, 8xon.1.C, 8xon.1.D, 8xon.1.E, 8xon.1.F, 8xoo.1.O, 8xoo.1.Q, 8xoo.1.R, 8xoo.1.S, 8xoo.1.T, 8xt1.1.a, 8xvb.1.B, 8xvc.1.A, 8xvg.1.B, 8xvg.1.C, 8xvg.1.D, 8xvg.1.F, 8xvg.1.G, 8xvg.1.I, 8xvt.1.D, 8xxn.1.a, 8y6o.1.E, 8y6o.1.I, 8yle.1.A, 8ynj.1.A, 9asj.1.A, 9ask.1.A, 9bh6.1.A, 9bh7.1.A, 9bh8.1.A, 9bh8.1.B, 9bh9.1.B, 9bht.1.C, 9bht.1.D, 9bhw.1.A, 9bhw.1.D, 9bqv.1.A, 9c5q.1.B, 9cc0.1.A, 9cc0.1.B, 9cc0.1.C, 9cc0.1.D, 9cc0.1.E, 9cc0.1.F, 9cc3.1.B, 9cc3.1.C, 9cc3.1.D, 9cc3.1.E, 9cc3.1.F, 9cc3.1.G, 9ema.1.A, 9ema.1.C, 9ema.1.D, 9emc.1.E, 9ezx.1.A, 9fmd.10.A, 9fmd.25.A, 9fmd.31.A, A9QW21.1.A

Swiss Institute of Bioinformatics
Contact Us
