# Supplementary material for: Molecular Ruler Variation in Insect Dicer-2 Suggests a Structural Basis for Species-Dependent siRNA Length and Antiviral Defense Diversity
Source: Viruses. 2026 Feb 27;18(3):285. doi: 10.3390/v18030285 (PMC13030778; doi:10.3390/v18030285)
Supplement: Supplementary file 1 [file viruses-18-00285-s001.zip › Tribolium_report_7w0e.html]

Triboli\_dicer2 | Report


Homology Modelling Report

## Model Building Report

This document lists the results for the homology modelling project "Triboli\_dicer2" submitted to SWISS-MODEL workspace
on Dec. 20, 2024, 2:33 p.m..The submitted primary amino acid sequence is given in Table T1.

If you use any results in your research, please cite the relevant publications:

- Waterhouse A, Bertoni M, Bienert S, Studer G, Tauriello G, Gumienny R, Heer FT, de Beer TAP, Rempfer C, Bordoli L, Lepore R, Schwede T

  SWISS-MODEL: homology modelling of protein structures and complexes.

  Nucleic Acids Res 46, W296-W303. (2018) 2978835510.1093/nar/gky427
- Bienert S, Waterhouse A, de Beer TAP, Tauriello G, Studer G, Bordoli L, Schwede T

  The SWISS-MODEL Repository - new features and functionality.

  Nucleic Acids Res 45, D313-D319. (2017) 2789967210.1093/nar/gkw1132
- Studer G, Tauriello G, Bienert S, Biasini M, Johner N, Schwede T

  ProMod3 - A versatile homology modelling toolbox.

  PLOS Comp Biol 17(1), e1008667. (2021) 3350798010.1371/journal.pcbi.1008667
- Studer G, Rempfer C, Waterhouse AM, Gumienny R, Haas J, Schwede T

  QMEANDisCo - distance constraints applied on model quality estimation.

  Bioinformatics 36, 1765-1771. (2020) 3169731210.1093/bioinformatics/btz828
- Bertoni M, Kiefer F, Biasini M, Bordoli L, Schwede T

  Modeling protein quaternary structure of homo- and hetero-oligomers beyond binary interactions by homology.

  Scientific Reports 7. (2017) 2887468910.1038/s41598-017-09654-8

## Results

The user uploaded a template structure to use for the modelling process.

## Models

The following model was built (see Materials and Methods "Model Building"):

| Model #01 | File | Built with | Oligo-State | Ligands | GMQE | QMEANDisCo Global |
| --- | --- | --- | --- | --- | --- | --- |
|  | PDB | ProMod3 3.4.1 | monomer | None | 0.55 | 0.54 ± 0.05 |

|  |  |  |
| --- | --- | --- |
|  |  |  |

| Template | Seq Identity | Oligo-state | QSQE | Found by | Method | Resolution | Seq Similarity | Range | Coverage | Description |
| --- | --- | --- | --- | --- | --- | --- | --- | --- | --- | --- |
| template\_upload.1.A | 30.09 | monomer | 0.00 | HHblits | Unknown | - | 0.36 | 4 - 1622 | 0.91 | Polypeptide |

  

### The template contained no ligands.

  

```
Target                   MDEEDELKPRNYQVNLMEIAIRENTIIYLPTGSGKTFIAIMVLKQLCAPILRPYS  
template_upload.1.A      ---DVEIKPRGYQLRLVDHLTKSNGIVYLPTGSGKTFVAILVLKRFSQDFDKPIE  
  
Target                   DGGKISVILVNSVALVDQHGKYVRDHATFSVGTYTGEMNVDFWSEAEWEQQFNKY  
template_upload.1.A      SGGKRALFMCNTVELARQQAMAVRRCTNFKVGFYVGEQGVDDWTRGMWSDEIKKN  
  
Target                   QVVIMTSQIMVNLINNRFIDLGKVNLMIFDECHHGVEDQPMRQIMKHFHSC--TD  
template_upload.1.A      QVLVGTAQVFLDMVTQTYVALSSLSVVIIDECHHGTGHHPFREFMRLFTIANQTK  
  
Target                   KPRVLGLTATLLNGNCKLSKVMDEIRSLEVTFHSKVATVEGL---DVVVGYSTNP  
template_upload.1.A      LPRVVGLTGVLIKGNE-ITNVATKLKELEITYRGNIITVSDTKEMENVMLYATKP  
  
Target                   QELFKVCQPGALSLDAKQV-LNNLRQLINDLEHINIKDEQNSVNLLQSETLKPLE  
template_upload.1.A      TEVMVSFPHQEQVLTVTRLISAEIEKFYVSLDLMNIGVQ----PIRRSKSLQCLR  
  
Target                   PSDVLKSLRNLISDLMIHIEMLGAFGGHIACVAHMIQIERIKKHCQNHQLFIVLN  
template_upload.1.A      DPSKKSFVKQLFNDFLYQMKEYGIYAASIAIISLIVEFDIKRRQAETLSVKLMHR  
  
Target                   YVMTIMGTTKLLLEETMA---------GYEPLEKIRKFSSDKVLKVFEILDEYKT  
template_upload.1.A      TALTLCEKIRHLLVQKLQDMTYDDDDDNVNTEEVIMNFSTPKVQRFLMSLKVSFA  
  
Target                   K-SDEELCCLVFTKRRFTAKVLHHIIDKASQVDPKF-YHIKSNFVVGNKNNPYND  
template_upload.1.A      DKDPKDICCLVFVERRYTCKCIYGLLLNYIQSTPELRNVLTPQFMVGRNNISP--  
  
Target                   TRENLYITKKNREVLNSFVSKEINVLVSSNVLEEGVDIPKCTLVIKFDKSEDYRS  
template_upload.1.A      DFESVLERKWQKSAIQQFRDGNANLMICSSVLEEGIDVQACNHVFILDPVKTFNM  
  
Target                   YIQSKGRARHIKSLYYTIVETTDVAKYDKKYSAFKEIENLVNDLLIGKNSERDHP  
template_upload.1.A      YVQSKGRARTTEAKFVLFTADKEREKTIQQIYQYRKAHNDIAEYLKDRVLEKTEP  
  
Target                   NLSEIRNMYNEDKLEPYYVNGPNSAQVNMTSAVALLCRYCSNLASDKYTTYAPEW  
template_upload.1.A      ELYEIKGHFQ-DDIDP-F-TNENGAVLLPNNALAILHRYCQTIPTDAFGFVIPWF  
  
Target                   YYEEDS---------SSAKLRVVIFLPVVCPLIDPIVGPYMHNKKDAKRAAALVA  
template_upload.1.A      HVLQEDERDRIFGVSAKGKHVISINMPVNCMLRDTIYSDPMDNVKTAKISAAFKA  
  
Target                   CIKLHQCGELDNNLLPWKKQLDEA-DVSYLFTHWPQEKES-----DAGNKKKKRL  
template_upload.1.A      CKVLYSLGELNERFVPKTLKERVASIADVHFEHWNKYGDSVTATVNKADKSKDRT  
  
Target                   HDKEIAPSVKSAI-QPDRVLYLHTININPQYKRSDDLKNAVTIYDLYKTPLKFGL  
template_upload.1.A      YKTECPLEFYDALPRVGEICYAYEIFLEPQFESC---EYTEHMYLNLQTPRNYAI  
  
Target                   LSPKPLPDLCKFPLFDSNGTLEIEIRNNV-REVEFAANEMKEMREFHFLVFNDLL  
template_upload.1.A      LLRNKLPRLAEMPLFSNQGKLHVRVANAPLEVIIQNSEQLELLHQFHGMVFRDIL  
  
Target                   EILKEFLIFDNTGMNSEMLLVVPVQDRC-GDVCVDFRVIRDNKNLKNKLEPAATE  
template_upload.1.A      KIWHPFFVLDRRS-KENSYLVVPLILGAGEQKCFDWELMTNFRRLPQSHGSNVQQ  
  
Target                   R-INLNVTEETYLHKIVSPWYRSPPKMYVVTKVCPDKSALSRFPNH-EYPNFVSY  
template_upload.1.A      REQQPAPRPEDFEGKIVTQWYANYDKPMLVTKVHRELTPLSYMEKNQQDKTYYEF  
  
Target                   YSEKHSLS---ILDPSQPLLLVKGLSERLNAFKPRGAGGKRKKEKMYEELEEYLI  
template_upload.1.A      TMSKYGNRIGDVVHKDKFMIEVRDLTEQLTFYVHNRGKFN---AKSKAKMKVILI  
  
Target                   PELVIKQEFPSCLWIQARFLPSILSRLAYLLKLQQLQVDIARGIGAKAEYLKDCP  
template_upload.1.A      PELCFNFNFPGDLWLKLIFLPSILNRMYFLLHAEALRKRFNTYLNLHLL------  
  
Target                   PLELNLHLLHYEPNDPQLTQESDKSTPLIDNCLALECPKNLRTIQYNKDFAAKML  
template_upload.1.A      P--FNGT-------D---------------------YM-----------------  
  
Target                   EAEYYWKTIEEPKDIERNINVTVMDIEYYETFISHQPSKTGRLLKNDSPVKQQNV  
template_upload.1.A      ---------PRPLEIDYSLK--------------GK------------------V  
  
Target                   PAITYDCQFEAKQLQILDVQF---DNQSPNLCQIYQALTAAEANDIVNLERLETL  
template_upload.1.A      KPL-----------LILQKTVSKEHITPAEQGEFLAAITASSAADVFDMERLEIL  
  
Target                   GDSFLKFVASLYIIFKFPTYNEGKSTTLKGKLVSNKNLYYLGVRKNLGGILKNSD  
template_upload.1.A      GNSFLKLSATLYLASKYSDWNEGTLTEVKSKLVSNRNLLFCLIDADIPKTLNTIQ  
  
Target                   LSPS-DWVPPCFCIPQTISKAIGNKE-----YSVVSLFNCCISPEEQ-VSGNLNR  
template_upload.1.A      FTPRYTWLPPGISLPHNVLALWRENPEFAKIIGPHNLRDLALGDEESLVKGNCSD  
  
Target                   KTLSDMTTEEIAPD-----EENSYGNMCNFLNKQYVGDKSIADSVEALLGAYFLS  
template_upload.1.A      INYNRFVEGCRANGQSFYAGADFSSEVNFCVGLVTIPNKVIADTLEALLGVIVKN  
  
Target                   GGIQGGIKFMEWIGILPLSEQ--IQRLIETTQVDPVLNKKSTKTDVDFHMPQWRE  
template_upload.1.A      YGLQHAFKMLEYFKICRADIDKPLTQLLNLELGGKKMRANVNTTEIDGFLINHYY  
  
Target                   IEQRLGYTFTNRAFLLQALTHSSYSPNRITLSYERLEFLGDAVLDFLITCYIFEH  
template_upload.1.A      LEKNLGYTFKDRRYLLQALTHPSYPTNRITGSYQELEFIGNAILDFLISAYIFEN  
  
Target                   CGHLEPGQVTDLRSSLVNNNTFASLVVRCGFHKFLLMMNSNLQGHIDKFADYLAS  
template_upload.1.A      NTKMNPGALTDLRSALVNNTTLACICVRHRLHFFILAENAKLSEIISKFVNFQES  
  
Target                   KNYVIDDEVLILLEEDEMNIAEYVDVPKVLGDIFEALAGAIYLDSNKDLKTVWRV  
template_upload.1.A      QGHRV------------TNMSTNVDVPKALGDVLEALIAAVYLDCR-DLQRTWEV  
  
Target                   FYKIIWREIDLFSKNVPKNVIRRLYECHTVYPPQFSKALEVGNQKTMVSLDFMCE  
template_upload.1.A      IFNLFEPELQEFTRKVPINHIRQLVEHKHA-KPVFSSPIVEGE-TVMVSCQFTCM  
  
Target                   GRKKRVHGFGTNKILAKRAAAKIALRALKL  
template_upload.1.A      EKTIKVYGFGSNKDQAKLSAAKHALQQLS-
```

  


---

  

## Materials and Methods

## User Template Alignment

The user entered their own target sequence together with an uploaded a template structure file in PDB format.

## Model Building

Models are built based on the target-template alignment using ProMod3 (Studer et al.). Coordinates which are conserved between the target and the template are copied from the template to the model. Insertions and deletions are remodelled using a fragment library. Side chains are then rebuilt. Finally, the geometry of the resulting model is regularized by using a force field.

## Model Quality Estimation

The global and per-residue model quality has been assessed using the QMEAN scoring function (Studer et al.).

## Ligand Modelling

Ligands present in the template structure are transferred by homology to the model when the following criteria are met: (a) The ligands are annotated as biologically relevant in the template library, (b) the ligand is in contact with the model, (c) the ligand is not clashing with the protein, (d) the residues in contact with the ligand are conserved between the target and the template. If any of these four criteria is not satisfied, a certain ligand will not be included in the model. The model summary includes information on why and which ligand has not been included.

## Oligomeric State Conservation

The quaternary structure annotation of the template is used to model the target sequence in its oligomeric form. The method (Bertoni et al.) is based on a supervised machine learning algorithm, Support Vector Machines (SVM), which combines interface conservation, structural clustering, and other template features to provide a quaternary structure quality estimate (QSQE). The QSQE score is a number between 0 and 1, reflecting the expected accuracy of the interchain contacts for a model built based a given alignment and template. Higher numbers indicate higher reliability. This complements the GMQE score which estimates the accuracy of the tertiary structure of the resulting model.

## References

- Camacho C, Coulouris G, Avagyan V, Ma N, Papadopoulos J, Bealer K, Madden TL

  BLAST+: architecture and applications.

  BMC Bioinformatics, 10, 421-430. (2009) 2000350010.1186/1471-2105-10-421
- Steinegger M, Meier M, Mirdita M, Vöhringer H, Haunsberger SJ, Söding J

  HH-suite3 for fast remote homology detection and deep protein annotation.

  BMC Bioinformatics 20, 473. (2019) 3152111010.1186/s12859-019-3019-7

## Table T1:

Primary amino acid sequence for which templates were searched and models were built.

MDEEDELKPRNYQVNLMEIAIRENTIIYLPTGSGKTFIAIMVLKQLCAPILRPYSDGGKISVILVNSVALVDQHGKYVRDHATFSVGTYTGEMNVDFWSE  
AEWEQQFNKYQVVIMTSQIMVNLINNRFIDLGKVNLMIFDECHHGVEDQPMRQIMKHFHSCTDKPRVLGLTATLLNGNCKLSKVMDEIRSLEVTFHSKVA  
TVEGLDVVVGYSTNPQELFKVCQPGALSLDAKQVLNNLRQLINDLEHINIKDEQNSVNLLQSETLKPLEPSDVLKSLRNLISDLMIHIEMLGAFGGHIAC  
VAHMIQIERIKKHCQNHQLFIVLNYVMTIMGTTKLLLEETMAGYEPLEKIRKFSSDKVLKVFEILDEYKTKSDEELCCLVFTKRRFTAKVLHHIIDKASQ  
VDPKFYHIKSNFVVGNKNNPYNDTRENLYITKKNREVLNSFVSKEINVLVSSNVLEEGVDIPKCTLVIKFDKSEDYRSYIQSKGRARHIKSLYYTIVETT  
DVAKYDKKYSAFKEIENLVNDLLIGKNSERDHPNLSEIRNMYNEDKLEPYYVNGPNSAQVNMTSAVALLCRYCSNLASDKYTTYAPEWYYEEDSSSAKLR  
VVIFLPVVCPLIDPIVGPYMHNKKDAKRAAALVACIKLHQCGELDNNLLPWKKQLDEADVSYLFTHWPQEKESDAGNKKKKRLHDKEIAPSVKSAIQPDR  
VLYLHTININPQYKRSDDLKNAVTIYDLYKTPLKFGLLSPKPLPDLCKFPLFDSNGTLEIEIRNNVREVEFAANEMKEMREFHFLVFNDLLEILKEFLIF  
DNTGMNSEMLLVVPVQDRCGDVCVDFRVIRDNKNLKNKLEPAATERINLNVTEETYLHKIVSPWYRSPPKMYVVTKVCPDKSALSRFPNHEYPNFVSYYS  
EKHSLSILDPSQPLLLVKGLSERLNAFKPRGAGGKRKKEKMYEELEEYLIPELVIKQEFPSCLWIQARFLPSILSRLAYLLKLQQLQVDIARGIGAKAEY  
LKDCPPLELNLHLLHYEPNDPQLTQESDKSTPLIDNCLALECPKNLRTIQYNKDFAAKMLEAEYYWKTIEEPKDIERNINVTVMDIEYYETFISHQPSKT  
GRLLKNDSPVKQQNVPAITYDCQFEAKQLQILDVQFDNQSPNLCQIYQALTAAEANDIVNLERLETLGDSFLKFVASLYIIFKFPTYNEGKSTTLKGKLV  
SNKNLYYLGVRKNLGGILKNSDLSPSDWVPPCFCIPQTISKAIGNKEYSVVSLFNCCISPEEQVSGNLNRKTLSDMTTEEIAPDEENSYGNMCNFLNKQY  
VGDKSIADSVEALLGAYFLSGGIQGGIKFMEWIGILPLSEQIQRLIETTQVDPVLNKKSTKTDVDFHMPQWREIEQRLGYTFTNRAFLLQALTHSSYSPN  
RITLSYERLEFLGDAVLDFLITCYIFEHCGHLEPGQVTDLRSSLVNNNTFASLVVRCGFHKFLLMMNSNLQGHIDKFADYLASKNYVIDDEVLILLEEDE  
MNIAEYVDVPKVLGDIFEALAGAIYLDSNKDLKTVWRVFYKIIWREIDLFSKNVPKNVIRRLYECHTVYPPQFSKALEVGNQKTMVSLDFMCEGRKKRVH  
GFGTNKILAKRAAAKIALRALKL

## Table T2:

| Template | Seq Identity | Oligo-state | QSQE | Found by | Method | Resolution | Seq Similarity | Coverage | Description |
| --- | --- | --- | --- | --- | --- | --- | --- | --- | --- |
| template\_upload.1.A | 30.09 | monomer | - | HHblits | Unknown | NA | 0.36 | 0.91 | Polypeptide |
| template\_upload.1.A | 30.94 | monomer | - | BLAST | Unknown | NA | 0.37 | 0.60 | Polypeptide |
| template\_upload.1.A | 38.95 | monomer | - | BLAST | Unknown | NA | 0.39 | 0.27 | Polypeptide |
| template\_upload.1.A | 27.50 | monomer | - | HHblits | Unknown | NA | 0.34 | 0.05 | Polypeptide |
| template\_upload.1.A | 28.05 | monomer | - | HHblits | Unknown | NA | 0.32 | 0.05 | Polypeptide |

  
The table above shows the top 5 filtered templates. A further 1 template was found which was considered to be less suitable for modelling than the filtered list.  
template\_upload.1.A

Swiss Institute of Bioinformatics
Contact Us
