# Supplementary figures and images for: Molecular Ruler Variation in Insect Dicer-2 Suggests a Structural Basis for Species-Dependent siRNA Length and Antiviral Defense Diversity
Source: Viruses. 2026 Feb 27;18(3):285. doi: 10.3390/v18030285 (PMC13030778; doi:10.3390/v18030285)

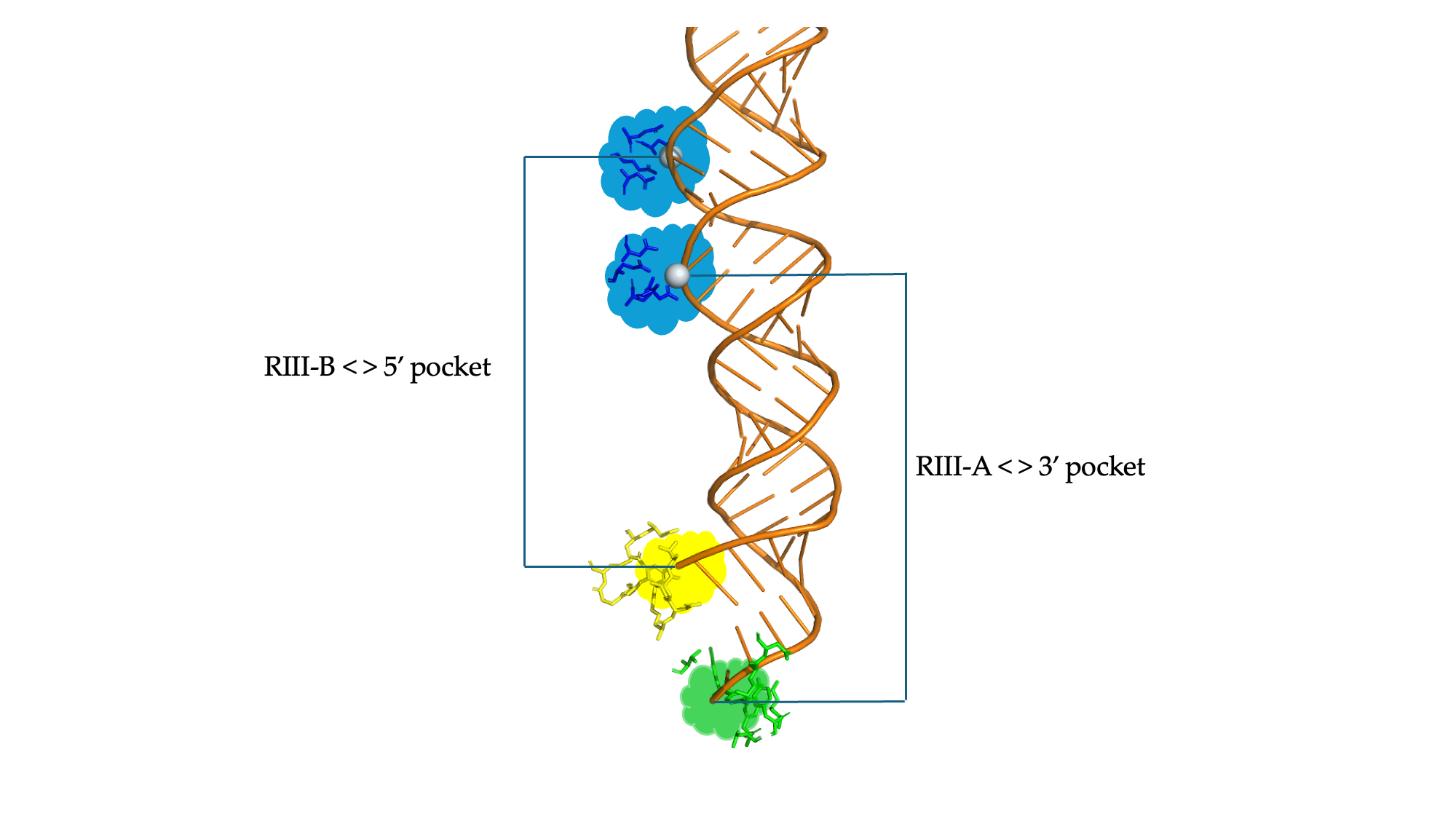

Supplement: Supplementary file 1 [file viruses-18-00285-s001.zip › R1-Supplementary Figure 1.png]
